# Atrial fibrillation and comorbidities: Clinical characteristics and antithrombotic treatment in GLORIA-AF

**DOI:** 10.1371/journal.pone.0249524

**Published:** 2021-04-14

**Authors:** Monika Kozieł, Christine Teutsch, Jonathan L. Halperin, Kenneth J. Rothman, Hans-Christoph Diener, Chang-Sheng Ma, Sabrina Marler, Shihai Lu, Venkatesh K. Gurusamy, Menno V. Huisman, Gregory Y. H. Lip, on behalf of the GLORIA-AF Investigators

**Affiliations:** 1 Liverpool Centre for Cardiovascular Science, University of Liverpool and Liverpool Heart & Chest Hospital, Liverpool, United Kingdom; 2 1st Department of Cardiology and Angiology, Silesian Centre for Heart Diseases, Zabrze, Poland; 3 Department of Clinical Development and Medical Affairs, Therapeutic Area Cardiometabolism, Boehringer Ingelheim International GmbH, Ingelheim, Germany; 4 Icahn School of Medicine at Mount Sinai, New York, New York, United States of America; 5 RTI Health Solutions, Research Triangle Park, North Carolina, United States of America; 6 Institute for Medical Informatics, Biometry and Epidemiology, University of Duisburg-Essen, Essen, Germany; 7 Cardiology Department, Atrial Fibrillation Center, Beijing AnZhen Hospital, Capital Medical University, Beijing, China; 8 Biostatistics and Data Sciences, Boehringer Ingelheim Pharmaceuticals, Inc., Ridgefield, Connecticut, United States of America; 9 Global Epidemiology, Boehringer Ingelheim International GmbH, Ingelheim, Germany; 10 Department of Thrombosis and Hemostasis, Leiden University Medical Center, Leiden, the Netherlands; 11 Aalborg Thrombosis Research Unit, Department of Clinical Medicine, Aalborg University, Aalborg, Denmark; Institut d’Investigacions Biomediques de Barcelona, SPAIN

## Abstract

**Background:**

Patients with AF often have multimorbidity (the presence of ≥2 concomitant chronic conditions).

**Objective:**

To describe baseline characteristics, patterns of antithrombotic therapy, and factors associated with oral anticoagulant (OAC) prescription in patients with AF and ≥2 concomitant, chronic, comorbid conditions.

**Methods:**

Phase III of the GLORIA-AF Registry enrolled consecutive patients from January 2014 through December 2016 with recently diagnosed AF and CHA_2_DS_2_-VASc score ≥1 to assess the safety and effectiveness of antithrombotic treatment.

**Results:**

Of 21,241 eligible patients, 15,119 (71.2%) had ≥2 concomitant, chronic, comorbid conditions. The proportions of patients with multimorbidity receiving non-vitamin K antagonist oral anticoagulants (NOACs) and vitamin K antagonists (VKA) were 60.2% and 23.6%, respectively. The proportion with paroxysmal AF was 57.0% in the NOAC group and 45.4% in the VKA group. Multivariable log-binomial regression analysis found the following factors were associated with no OAC prescription: pattern of AF (paroxysmal, persistent, or permanent), coronary artery disease, myocardial infarction, prior bleeding, smoking status, and region (Asia, North America, or Europe). Factors associated with OAC prescriptions were age, body mass index, renal function, hypertension, history of cerebral ischemic symptoms, and AF ablation.

**Conclusion:**

Multimorbid AF patients prescribed NOACs have fewer comorbidities than those prescribed VKAs. Age, AF pattern, comorbidities, and renal function are associated with OAC prescription.

## Introduction

Atrial fibrillation (AF) affects approximately 3% of adults and its prevalence and incidence are rising [1] with the aging of the population [2]. Older patients with AF often have other chronic conditions that affect their clinical course [3]. Multimorbidity (the presence of ≥2 concomitant chronic conditions) demands a holistic and integrated approach to patient care [4] since these patients face higher risks of stroke and bleeding than those without comorbidities [5, 6]. The interplay between comorbidity, AF, and optimal thromboprophylaxis has both medical and economic implications [7]

The aim of this analysis of the GLORIA-AF dataset is to describe baseline characteristics and antithrombotic therapy prescription patterns in patients with AF and multimorbidity and to identify factors associated with the selection of an oral anticoagulant (OAC) type for these complex patients.

## Materials and methods

The design of the GLORIA-AF registry (https://clinicaltrials.gov/ct2/home; trial registration numbers NCT01468701, NCT01671007, NCT01937377) has been reported [8]. The study protocol is concordant with the ethical guidelines of the 1975 Declaration of Helsinki, and informed consent was obtained from each patient before enrollment.

The registry collected routine clinical practice data regarding patients with newly diagnosed AF to evaluate patient characteristics influencing the selection, safety, and effectiveness of antithrombotic therapy. Phase I was conducted before non-vitamin K antagonist oral anticoagulants (NOACs) were available for stroke prevention in AF. Phase II began when dabigatran was approved in countries with participating clinical centers. Baseline characteristics were collected and those prescribed dabigatran were followed up for 2 years in Phase II. Phase III, which started when dabigatran had been more widely adopted, gathered data for up to 3 years, regardless of antithrombotic management [8].

Consecutive patients from 38 countries were enrolled between 2014 and 2016. Adult patients with recently diagnosed nonvalvular AF (<3 months before the baseline visit; Latin America <4.5 months) at risk of stroke (CHA_2_DS_2_-VASc score ≥1) achieved by any of the following: heart failure or left ventricular systolic dysfunction, hypertension, diabetes, prior stroke, transient ischemic attack (TIA) or systemic embolism, myocardial infarction (MI), peripheral artery disease, age ≥65 years, or female sex, were enrolled [9]. The risks of stroke and bleeding were assessed using the CHA_2_DS_2_-VASc and HAS-BLED (1 point is achieved by any of the following: hypertension, abnormal renal or hepatic function, prior stroke, bleeding or predisposition, labile International Normalised Ratio, elderly [>65 years], or concomitant use of alcohol or anti-inflammatory medications) [10]. Antithrombotic therapy was prescribed by the treating physicians according to local standards. This report is focused on baseline data obtained from patients in Phase III, collected using electronic case report forms.

### Statistical analysis

Baseline characteristics are summarized descriptively. Categorical variables are reported as absolute frequencies and percentages, and continuous variables are summarized by median (Quartile 1, Quartile 3). Baseline characteristics included stratification of patients with AF and multimorbidity according to stroke prevention strategies (OAC vs antiplatelet vs no antithrombotic therapy, NOAC vs vitamin K antagonists [VKAs], and NOACs once daily [QD] vs twice daily [BID]). Standardized differences were used to compare baseline characteristics across various stroke prevention strategies, focusing on variables with the highest standardized differences; differences ≤10% in absolute value were considered as balanced between groups [11].

Factors associated with antithrombotic treatment choice were analyzed by log-binomial, multivariable regression models, providing relative probability ratios for prescription (OAC vs no OAC use, NOAC vs VKA; and by region). Missing data were handled using multiple imputation, replacing missing data with multiple simulated values based on regression models to provide comparatively unbiased estimates under the missing-at-random assumption. The procedure introduces random error to compensate for the added, imputed information. The imputation regression models used 56 predictors to impute the missing data, and were repeated 20 times to give 20 datasets with imputed data [12].

Confidence intervals were calculated based on likelihood ratios and Rubin’s method to combine results across imputations. Both univariate and multivariable log-binomial regression analyses were performed to evaluate crude as well as the adjusted probability ratios together with 95% confidence intervals. The term “probability ratio” was used rather than “risk ratio”, as our measure describes treatment selections rather than adverse outcomes.

All data were calculated using SAS version 9.4 (SAS Institute, Inc., Cary, NC).

## Results

Of 21,241 eligible patients in this subanalysis, 15,119 (71.2%) had ≥2 concomitant, chronic conditions (Table 1).

**Table 1:** Proportion of AF patients according to number of comorbid diseases^a^.

Number of Comorbid Diseases	Number of Patients (n = 21,241)
0	1434 (6.8)
1	4688 (22.1)
2	5559 (26.2)
3	4286 (20.2)
4	2664 (12.5)
5	1463 (6.9)
6	695 (3.3)
7	332 (1.6)
8	88 (0.4)
9	22 (0.1)
10	8 (0.0)
11	2 (0.0)

^a^AF = atrial fibrillation.

### Baseline characteristics of AF multimorbid patients

Baseline characteristics of patients are summarized based on antithrombotic therapy (Table 2). Among multimorbid AF patients, 83.8% were prescribed OACs, 11.0% were prescribed antiplatelet therapy, and 5.2% were prescribed no antithrombotic therapy. The median (66.0, 79.0) age was 73.0 years in the OAC group, 71.0 (63.0–79.0) years in the antiplatelet therapy group, and 72.0 (64.0–80.0) years in the no antithrombotic therapy group. The proportions of females in these groups were 44.5%, 41.7%, and 45.5%, respectively. The median CHA_2_DS_2_-VASc and HAS-BLED scores were similar across the 3 groups.

**Table 2:** Baseline characteristics of AF multimorbid patients prescribed OAC or antiplatelets or no antithrombotic therapy^a^.

	OAC (n = 12,677)	Antiplatelets (n = 1658)	No Antithrombotic Therapy (n = 784)
Age (y), median (Q1, Q3)	73.0 (66.0–79.0)	71.0 (63.0–79.0)	72.0 (64.0–80.0)
Females, n (%)	5645 (44.5)	691 (41.7)	357 (45.5)
BMI (kg/m^2^), median (Q1, Q3)	28.0 (24.8–32.0)	26.1 (23.5–30.0)	26.1 (23.4–29.6)
Missing	123 (1.0)	17 (1.0)	8 (1.0)
Current smoker	1145 (9.0)	223 (13.4)	100 (12.8)
Alcohol abuse, ≥8 units/ week	866 (6.8)	85 (5.1)	54 (6.9)
Type of AF, n (%)			
Paroxysmal	6810 (53.7)	1166 (70.3)	496 (63.3)
Persistent	4478 (35.3)	401 (24.2)	242 (30.9)
Permanent	1389 (11.0)	91 (5.5)	46 (5.9)
Categorization of AF, n (%)			
EHRA I	4686 (37.0)	550 (33.2)	273 (34.8)
EHRA II	4025 (31.8)	563 (34.0)	270 (34.4)
EHRA III	3063 (24.2)	431 (26.0)	183 (23.3)
EHRA IV	903 (7.1)	114 (6.9)	58 (7.4)
Creatinine clearance (mL/min) (measured), median (Q1, Q3)	70.6 (52.5–95.3)	69.5 (50.9–92.4)	67.8 (49.7–90.3)
Creatinine clearance (mL/min), n (%)			
<15	100 (0.8)	18 (1.1)	10 (1.3)
15–29	305 (2.4)	62 (3.7)	23 (2.9)
30–49	1848 (14.6)	252 (15.2)	136 (17.3)
50–79	4152 (32.8)	526 (31.7)	253 (32.3)
≥80	4080 (32.2)	520 (31.4)	243 (31.0)
Missing	2192 (17.3)	280 (16.9)	119 (15.2)
CHA_2_DS_2_-VASc score, median (Q1, Q3)	4.0 (3.0–5.0)	4.0 (2.0–5.0)	3.0 (2.0–4.0)
HAS-BLED score, median (Q1, Q3)	1.0 (1.0–2.0)	2.0 (2.0–3.0)	1.0 (1.0–2.0)
Missing (HAS-BLED), n (%)	1234 (9.7)	134 (8.1)	69 (8.8)
Medical history, n (%)			
Congestive heart failure	3509 (27.7)	487 (29.4)	215 (27.4)
Hypertension	10,989 (86.7)	1370 (82.6)	638 (81.4)
Diabetes mellitus	4021 (31.7)	510 (30.8)	226 (28.8)
Previous stroke or TIA	2347 (18.5)	336 (20.3)	159 (20.3)
Myocardial infarction	1580 (12.5)	384 (23.2)	58 (7.4)
Coronary artery disease	3017 (23.8)	745 (44.9)	149 (19.0)
Peripheral artery disease	503 (4.0)	79 (4.8)	21 (2.7)
Cancer	1671 (13.2)	167 (10.1)	115 (14.7)
Dementia	101 (0.8)	18 (1.1)	1 (0.1)
Gastric ulcer	145 (1.1)	20 (1.2)	13 (1.7)
Gastritis or duodenitis	455 (3.6)	70 (4.2)	50 (6.4)
Chronic kidney disease	3881 (30.6)	526 (31.7)	271 (34.6)
COPD	1045 (8.2)	120 (7.2)	59 (7.5)
Bleeding (after diagnosis of AF), n (%)	182 (1.4)	32 (1.9)	33 (4.2)
Bleeding on OAC, n (%)	159 (87.4)	27 (84.4)	18 (54.5)
Location of bleeding (after diagnosis of AF), n (%)*			
Intracranial hemorrhage	12 (6.6)	6 (18.8)	8 (24.2)
Upper GI bleed	12 (6.6)	4 (12.5)	3 (9.1)
Lower GI bleed	25 (13.7)	6 (18.8)	5 (15.2)
GI bleed not further specified	11 (6.0)	4 (12.5)	4 (12.1)
Urogenital hemorrhage	31 (17.0)	3 (9.4)	3 (9.1)
Bleeding at other location	81 (44.5)	7 (21.9)	8 (24.2)
Bleeding with unknown location	10 (5.5)	2 (6.3)	2 (6.1)
Region, n (%)			
Asia	1739 (13.7)	719 (43.4)	325 (41.5)
Europe	6514 (51.4)	443 (26.7)	266 (33.9)
North America	3429 (27.0)	415 (25.0)	144 (18.4)
Latin America	995 (7.8)	81 (4.9)	49 (6.3)
Type of site, n (%)			
GP/primary care	686 (5.4)	171 (10.3)	77 (9.8)
Specialist office	3902 (30.8)	512 (30.9)	191 (24.4)
Community hospital	3757 (29.6)	350 (21.1)	175 (22.3)
University hospital	3878 (30.6)	543 (32.8)	326 (41.6)
Outpatient health care centre	222 (1.8)	51 (3.1)	6 (0.8)
Anticoagulation clinics	82 (0.6)	6 (0.4)	4 (0.5)
Other	150 (1.2)	25 (1.5)	5 (0.6)

^a^AF = atrial fibrillation; BMI = body mass index; CHA_2_DS_2_-VASc = congestive heart failure/left ventricular dysfunction, hypertension, age ≥75 years, diabetes, stroke/transient ischemic attack/systemic embolism, vascular disease, age 65–74 years, sex category (female); COPD = chronic obstructive pulmonary disease; EHRA = European Heart Rhythm Association; GI = gastrointestinal; GP = general practitioner; HAS-BLED = hypertension, abnormal renal /liver function, stroke, bleeding history or predisposition, labile International Normalised Ratio, elderly (>65 years), drugs or alcohol concomitantly; OAC = oral anticoagulant; Q = quartile; TIA = transient ischemic attack; y = years.

*Proportion calculated out of Bleeding (after diagnosis of AF).

Baseline characteristics of patients prescribed NOACs or VKAs are shown in Table 3. The median age was 73.0 (66.0–79.0) years, and the proportion of females was 44% in both treatment groups. There were no differences in CHA_2_DS_2_-VASc and HAS-BLED scores between these 2 groups. The prevalence of paroxysmal AF in patients with multimorbidity on NOACs and VKAs was 57.0% and 45.4%, respectively. Among patients on NOACs, 38.4% had a European Heart Rhythm Association symptom score of I, compared with 33.3% for patients on VKAs. A lower proportion (1.6%) of patients on NOACs had a glomerular filtration rate of 15–29 mL/min, compared with 4.4% of those on VKAs.

**Table 3:** Baseline characteristics of AF multimorbid patients prescribed NOACs or VKAs^a^.

	NOAC (n = 9105)	VKA (n = 3572)	Standardized Difference
Age (y), median (Q1, Q3)	73.0 (66.0–79.0)	73.0 (66.0–79.0)	0.005
Females, n (%)	4072 (44.7)	1573 (44.0)	–0.014
BMI (kg/m^2)^, median (Q1, Q3)	28.0 (24.8–32.2)	27.8 (24.6–31.6)	–0.066
Missing	37 (1.2)	60 (1.0)	0.020
Current smoker	812 (8.9)	333 (9.3)	0.014
Alcohol abuse, ≥8 units/ week	651 (7.1)	215 (6.0)	–0.046
Type of AF, n (%)			
Paroxysmal	5187 (57.0)	1623 (45.4)	–0.232
Persistent	3052 (33.5)	1426 (39.9)	0.133
Permanent	866 (9.5)	523 (14.6)	0.158
Categorization of AF, n (%)			
EHRA I	3496 (38.4)	1190 (33.3)	–0.106
EHRA II	2886 (31.7)	1139 (31.9)	0.004
EHRA III	2131 (23.4)	932 (26.1)	0.062
EHRA IV	592 (6.5)	311 (8.7)	0.083
Creatinine clearance (mL/min) (measured), median (Q1, Q3)	72.1 (53.7–97.0)	66.8 (48.9–91.0)	–0.078
Creatinine clearance (mL/min) n (%)			
<15	50 (0.5)	50 (1.4)	0.087
15–29	148 (1.6)	157 (4.4)	0.163
30–49	1280 (14.1)	568 (15.9)	0.052
50–79	3046 (33.5)	1106 (31.0)	–0.053
≥80	3053 (33.5)	1027 (28.8)	–0.103
Missing	1528 (16.8)	664 (18.6)	0.047
CHA_2_DS_2_-VASc score, median (Q1, Q3)	4.0 (3.0–5.0)	4.0 (3.0–5.0)	0.080
HAS-BLED score, median (Q1, Q3)	1.0 (1.0–2.0)	1.0 (1.0–2.0)	0.016
Missing (HAS-BLED), n (%)	858 (9.4)	376 (10.5)	0.037
Medical history, n (%)			
Congestive heart failure	2232 (24.5)	1277 (35.8)	0.247
Hypertension	7907 (86.8)	3082 (86.3)	–0.016
Diabetes mellitus	2839 (31.2)	1182 (33.1)	0.041
Previous stroke or TIA	1741 (19.1)	606 (17.0)	–0.056
Myocardial infarction	1039 (11.4)	541 (15.1)	0.110
Coronary artery disease	2104 (23.1)	913 (25.6)	0.057
Peripheral artery disease	355 (3.9)	148 (4.1)	0.012
Cancer	1223 (13.4)	448 (12.5)	–0.027
Dementia	76 (0.8)	25 (0.7)	–0.016
Gastric ulcer	111 (1.2)	34 (1.0)	–0.026
Gastritis or duodenitis	317 (3.5)	138 (3.9)	0.020
Chronic kidney disease	2663 (29.2)	1218 (34.1)	0.104
COPD	743 (8.2)	302 (8.5)	0.011
Bleeding (after diagnosis of AF), n (%)	130 (1.4)	52 (1.5)	0.002
Bleeding on OAC, n (%)	112 (86.2)	47 (90.4)	0.132
Location of bleeding (after diagnosis of AF), n (%)*			
Intracranial hemorrhage	11 (8.5)	1 (1.9)	–0.298
Upper GI bleed	8 (6.2)	4 (7.7)	0.061
Lower GI bleed	20 (15.4)	5 (9.6)	–0.175
GI bleed not further specified	9 (6.9)	2 (3.8)	–0.137
Urogenital hemorrhage	20 (15.4)	11 (21.2)	0.150
Bleeding at other location	56 (43.1)	25 (48.1)	0.101
Bleeding with unknown location	6 (4.6)	4 (7.7)	0.128
AF cardioversion	1814 (19.9)	521 (14.6)	–0.142
Region, n (%)			
Asia	1222 (13.4)	517 (14.5)	0.030
Europe	4498 (49.4)	2016 (56.4)	0.141
North America	2808 (30.8)	621 (17.4)	–0.319
Latin America	577 (6.3)	418 (11.7)	0.188
Type of site, n (%)			
GP/primary care	502 (5.5)	184 (5.2)	–0.016
Specialist office	3053 (33.5)	849 (23.8)	–0.217
Community hospital	2880 (31.6)	877 (24.6)	–0.158
University hospital	2454 (27.0)	1424 (39.9)	0.276
Outpatient health care centre	72 (0.8)	150 (4.2)	0.220
Anticoagulation clinics	37 (0.4)	45 (1.3)	0.094
Other	107 (1.2)	43 (1.2)	0.003

^a^AF = atrial fibrillation; BMI = body mass index; CHA_2_DS_2_-VASc = congestive heart failure/left ventricular dysfunction, hypertension, age ≥75 years, diabetes, stroke/transient ischemic attack/systemic embolism, vascular disease, age 65–74 years, sex category (female); COPD = chronic obstructive pulmonary disease; EHRA = European Heart Rhythm Association; GI = gastrointestinal; GP = general practitioner; HAS-BLED = hypertension, abnormal renal /liver function, stroke, bleeding history or predisposition, labile International Normalised Ratio, elderly (>65 years), drugs or alcohol concomitantly; NOAC = nonvitamin K antagonist oral anticoagulants; OAC = oral anticoagulant; Q = quartile; TIA = transient ischemic attack; VKA = vitamin K antagonists; y = years.

*Proportion calculated out of Bleeding (after diagnosis of AF).

Cardioversion was performed in 19.9% of patients on NOACs vs 14.6% of those on VKAs. Treatment in specialist offices was more prevalent for patients on NOACs (33.5% vs 23.8% in the VKA group), while comorbidities such as heart failure (HF) and MI were less prevalent among patients given NOACs.

Patient demographics, cardiovascular risk factors, comorbid diseases, AF categorization, stroke and bleeding risks, and concomitant treatments of patients on NOACs QD vs BID are summarized in Table 4 There were generally small differences between patients taking NOACs QD vs BID. Previous TIA or stroke were present in 14.9% of the patients on NOACs QD vs 21.3% of the patients on NOACs BID (Table 4).

**Table 4:** Baseline characteristics of AF multimorbid patients prescribed NOACs QD or NOACs BID.

	NOAC QD (n = 3071)	NOAC BID (n = 6034)	Standardized Difference
Age (y), median (Q1, Q3)	72.0 (65.0–79.0)	73.0 (66.0–79.0)	–0.098
Females, n (%)	1306 (42.5)	2766 (45.8)	–0.067
BMI (kg/m^2^), median (Q1, Q3)	28.3 (25.0–32.8)	27.9 (24.8–32.0)	0.089
Current smoker	250 (8.1)	562 (9.3)	–0.042
Alcohol abuse, ≥8 units/ week	242 (7.9)	409 (6.8)	0.042
Type of AF, n (%)			
Paroxysmal	1767 (57.5)	3420 (56.7)	0.017
Persistent	1045 (34.0)	2007 (33.3)	0.016
Permanent	259 (8.4)	607 (10.1)	–0.056
Categorization of AF, n (%)			
EHRA I	1138 (37.1)	2358 (39.1)	–0.042
EHRA II	983 (32.0)	1903 (31.5)	0.010
EHRA III	775 (25.2)	1356 (22.5)	0.065
EHRA IV	175 (5.7)	417 (6.9)	–0.050
Creatinine clearance (mL/min), (measured), median (Q1, Q3)	74.4 (55.3–101.8)	70.5 (53.1–94.3)	0.041
Creatinine clearance, n (%)			
<15	18 (0.6)	32 (0.5)	0.008
15–29	40 (1.3)	108 (1.8)	–0.040
30–49	401 (13.1)	879 (14.6)	–0.044
50–79	1018 (33.1)	2028 (33.6)	–0.010
≥80	1125 (36.6)	1928 (32.0)	0.099
Missing	469 (15.3)	1059 (17.6)	–0.062
CHA_2_DS_2_-VASc score, median (Q1, Q3)	3.0 (2.0–4.0)	4.0 (3.0–5.0)	–0.127
HAS-BLED score, median (Q1, Q3)	1.0 (1.0–2.0)	1.0 (1.0–2.0)	–0.066
Missing (HAS-BLED), n (%)	302 (9.8)	556 (9.2)	0.021
Medical history, n (%)			
Congestive heart failure	772 (25.1)	1460 (24.2)	0.022
Hypertension	2672 (87.0)	5235 (86.8)	0.007
Diabetes mellitus	1021 (33.2)	1818 (30.1)	0.067
Previous stroke or TIA	457 (14.9)	1284 (21.3)	–0.167
Myocardial infarction	366 (11.9)	673 (11.2)	0.024
Coronary artery disease	746 (24.3)	1358 (22.5)	0.042
Peripheral artery disease	119 (3.9)	236 (3.9)	–0.002
Cancer	407 (13.3)	816 (13.5)	–0.008
Dementia	24 (0.8)	52 (0.9)	–0.009
Gastric ulcer	40 (1.3)	71 (1.2)	0.011
Gastritis or duodenitis	116 (3.8)	201 (3.3)	0.024
Chronic kidney disease	839 (27.3)	1824 (30.2)	–0.064
COPD	258 (8.4)	485 (8.0)	0.013
Bleeding (after diagnosis of AF), n (%)	57 (1.9)	73 (1.2)	0.053
Bleeding on OAC, n (%)	52 (91.2)	60 (82.2)	0.269
Location of bleeding (after diagnosis of AF), n (%)			
Intracranial hemorrhage	2 (3.5)	9 (12.3)	–0.331
Upper GI bleed	4 (7.0)	4 (5.5)	0.064
Lower GI bleed	10 (17.5)	10 (13.7)	0.106
GI bleed not further specified	5 (8.8)	4 (5.5)	0.128
Urogenital hemorrhage	6 (10.5)	14 (19.2)	–0.245
Bleeding at other location	24 (42.1)	32 (43.8)	–0.035
Bleeding with unknown location	6 (10.5)	0 (0.0)	0.438
AF cardioversion	710 (23.1)	1104 (18.3)	0.119
Region, n (%)			
Asia	356 (11.6)	866 (14.4)	–0.082
Europe	1465 (47.7)	3033 (50.3)	–0.051
North America	1056 (34.4)	1752 (29.0)	0.115
Latin America	194 (6.3)	383 (6.3)	–0.001
Type of site, n (%)			
GP/primary care	184 (6.0)	318 (5.3)	0.031
Specialist office	1110 (36.1)	1943 (32.2)	0.083
Community hospital	921 (30.0)	1959 (32.5)	–0.053
University hospital	773 (25.2)	1681 (27.9)	–0.061
Outpatient health care center	19 (0.6)	53 (0.9)	–0.030
Anticoagulation clinics	18 (0.6)	19 (0.3)	0.041
Other	46 (1.5)	61 (1.0)	0.044

^a^AF = atrial fibrillation; BID = twice daily; BMI = body mass index; CHA_2_DS_2_-VASc = congestive heart failure/left ventricular dysfunction, hypertension, age ≥75 years, diabetes, stroke/transient ischemic attack/systemic embolism, vascular disease, age 65–74 years, sex category (female); COPD = chronic obstructive pulmonary disease; EHRA = European Heart Rhythm Association; GI = gastrointestinal; GP = general practitioner; HAS-BLED = hypertension, abnormal renal /liver function, stroke, bleeding history or predisposition, labile International Normalised Ratio, elderly (>65 years), drugs or alcohol concomitantly; NOAC = nonvitamin K antagonist oral anticoagulants; OAC = oral anticoagulant; Q = quartile; QD = once daily; TIA = transient ischemic attack.

### Factors associated with OAC non-prescription in multimorbid AF patients globally

Results from univariate analyses are presented in the S1 File. In the multivariable log-binomial regression analysis, factors associated with prescriptions for no OAC use in multimorbid AF patients were: type of AF (paroxysmal/persistent vs permanent), coronary artery disease (CAD), MI, history of bleeding, smoking status (current vs nonsmoker), and region (Asia, North America vs Europe). Factors associated with increased OAC use were: age 65–74 vs ≥75 years, body mass index (BMI) class (≥25 vs 18.5–24 kg/m^2^), creatinine clearance (30–59 vs ≥80 mL/min), hypertension, prior TIA or stroke, and AF ablation (Table 5).

**Table 5:** Multivariable log-binomial analysis for factors associated with prescription of OAC therapy (no OAC vs OAC)^a^^,^^b^.

Factor	Relative Risk (95% CI) For Prescription of No OAC Globally
Age	
<65	1.05 (0.95–1.16)
65–74	0.90 (0.83–0.99)
≥75	1.0 (ref)
BMI class	
<18.5	0.98 (0.77–1.24)
18.5–24	1.0 (ref)
25–29	0.85 (0.79–0.91)
30–34	0.77 (0.69–0.87)
≥35	0.70 (0.60–0.81)
Gender	
Male	1.0 (ref)
Female	1.05 (0.97–1.13)
Current smoker	1.14 (1.03–1.25)
Past smoker	0.91 (0.84–0.99)
Categorization of AF	
EHRA I	1.0 (ref)
EHRA II	1.04 (0.96–1.12)
EHRA III	0.99 (0.91–1.07)
EHRA IV	1.07 (0.95–1.20)
Type of AF	
Paroxysmal	1.67 (1.42–1.97)
Persistent	1.20 (1.02–1.43)
Permanent	1.0 (ref)
Hypertension	0.89 (0.83–0.97)
Coronary artery disease	1.42 (1.31–1.53)
Myocardial infarction	1.18 (1.08–1.28)
Congestive heart failure	1.01 (0.94–1.08)
Diabetes mellitus	0.95 (0.88–1.02)
Previous TIA or stroke	0.81 (0.68–0.97)
Bleeding after diagnosis of AF	1.60 (1.42–1.79)
Peripheral artery disease	1.13 (0.96–1.34)
Cancer	1.00 (0.90–1.12)
Functional dyspepsia	0.85 (0.56–1.27)
Gastric ulcer	0.91 (0.69–1.21)
Gastritis or duodenitis	0.95 (0.82–1.10)
COPD	1.03 (0.90–1.19)
Hyperthyroidism	0.96 (0.79–1.17)
Hepatic disease	1.05 (0.87–1.27)
Dementia	1.09 (0.76–1.56)
AF cardioversion	0.96 (0.89–1.04)
Creatinine clearance (mL/min)	
<30	1.09 (0.94–1.26)
30–59	0.88 (0.79–0.97)
60–79	0.91 (0.83–1.00)
≥80	1.0 (ref)
AF ablation	0.30 (0.20–0.45)
Region	
Asia	3.17 (2.88–3.49)
Europe	1.0 (ref)
North America	1.24 (1.11–1.39)
Latin America	1.14 (0.96–1.37)
Medical treatment reimbursed by	
Self-pay/no coverage	0.82 (0.69–0.96)
Not self-pay	1.0 (ref)
Type of site	
Specialist office	1.26 (1.14–1.39)
Community hospital	1.0 (ref)
University hospital	1.28 (1.17–1.40)

^a^A few other variables (alcohol abuse, psychosocial factors, biological heart valve implant, valve repair, and peptic ulcer) are included in the multivariable log-binomial regression analysis model and are presented in the S1 File.

^b^AF = atrial fibrillation; BMI = body mass index; CI = confidence interval; COPD = chronic obstructive pulmonary disease; EHRA = European Heart Rhythm Association; OAC = oral anticoagulant; ref = reference; TIA = transient ischemic attack.

### Factors associated with OACs non-prescription in multimorbid AF patients in Asia, Europe, and North America

Factors associated with prescriptions for no OAC use in multimorbid AF patients in Asia, Europe, and North America are presented in **S1 Table in** S2 File. Factors associated with increased OAC use are included in **S1 Table in** S2 File.

### Factors associated with type of OAC use in multimorbid AF patients globally

Factors associated with prescriptions for VKA use globally in multimorbid AF patients were: age <75 vs ≥75 years, MI, congestive HF, diabetes mellitus, creatinine clearance (<60 vs ≥80 mL/min), **S2 Table in** S2 File.

Factors associated with decreased VKA use globally were: type of AF (paroxysmal/persistent vs permanent), previous TIA or stroke, medical treatment reimbursement (self-pay/no coverage vs not self-pay), **S2 Table in** S2 File.

### Factors associated with OAC use in multimorbid AF patients in Asia, Europe, North America, and Latin America

Factors associated with prescriptions for VKA use in multimorbid AF patients in Asia, Europe, North America, and Latin America are presented in **S3 Table in** S2 File. Factors associated with decreased prescriptions for VKA use in multimorbid AF patients in Asia, Europe, North America, and Latin America are presented in **S3 Table in** S2 File.

## Discussion

There are still knowledge gaps in how OACs are used in clinical practice in patients with AF and multiple comorbidities and which factors influence OAC prescription in such patients. Our study shows that, despite a median CHA_2_DS_2_-VASc score >3, approximately 16% of patients with multimorbidity and AF are not anticoagulated. The baseline characteristics in these complex patients differ in relation to antithrombotic therapy selection, suggesting that comorbidities may influence antithrombotic therapy prescription patterns for patients with AF. For example, prescription of OACs globally in patients with AF and multimorbidity was associated with age, BMI, cardiovascular risk factors (smoking status), AF pattern, concomitant diseases (ie, hypertension, CAD, MI, previous TIA or stroke), history of bleeding, renal function, rhythm control strategy (AF ablation and AF cardioversion), and region (Asia and North America). Prescriptions patterns were also subject to regional differences in clinical practice.

### Patient characteristics according to antithrombotic therapy use

The results suggest that patients with AF and multimorbidity prescribed NOACs are more likely to have paroxysmal AF, and have fewer comorbidities than those prescribed VKAs, consistent with other reports [13–15]. Declining renal function may influence the choice of VKA in those with chronic kidney disease. Healthcare system-related factors (such as center type) also influence treatment strategies. Patients with AF and multimorbidity treated in specialist offices and community hospitals are more often prescribed NOACs than VKAs.

The patients in this cohort prescribed antiplatelet agents had a higher risk of bleeding according to HAS-BLED score than those who were prescribed OACs. They also more often had paroxysmal AF compared to those prescribed OACs. Patients with AF and CAD were more often prescribed antiplatelets than OACs despite the fact that antiplatelet therapy does not prevent stroke or reduce mortality, elevates the risk of bleeding, and is not recommended for prevention of AF-related thromboembolism [16]. Unfortunately, antiplatelet monotherapy is still a frequent choice of prescribing physicians based on several European reports [17, 18].

### Factors associated with OAC prescription in multimorbid AF patients globally

The majority of multimorbid AF patients had a high risk of stroke (CHA_2_DS_2_-VASc score ≥2) and oral anticoagulation therapy is recommended for these patients [19]. Hypertension and HF were the most prevalent risk factors for thromboembolic complications [20] and these factors and previous stroke or TIA are associated with a greater frequency of OAC prescription. Prescription of OACs was inversely associated with comorbidities that are strongly associated with elevated thromboembolic risk (eg, MI, CAD), just as conditions associated with an increased risk of bleeding (eg, previous hemorrhagic events) were associated with less frequent prescription of OACs. This is also consistent with prior reports [13] although current clinical practice guidelines recommend that patients with AF at a high risk of bleeding should generally continue anticoagulation with frequent visits and close monitoring [21]. A history of AF ablation in multimorbid AF patients was associated with more frequent OAC prescription as per guidelines [21] and consistent with other studies [22].

Younger age (≤75 years) was associated with greater OAC prescription and more frequent selection of VKAs compared to practice patterns for older patients. Several studies have suggested that increasing age is a barrier to implementing OAC use [23, 24]. Importantly, stroke risk increases with age, and the absolute benefit of OACs is clearly increased for older patients with AF [25]. In one report, when adjusted for comorbidity, age was not an important determinant of anticoagulation [26].

Multimorbid AF patients with paroxysmal or persistent AF were less often prescribed OACs in particular VKAs than those with permanent AF. NOACs should be preferred in patients with multimorbidity and polypharmacy given their lower number of drug–drug interactions compared with VKAs [27]. Ischemic stroke may occur as frequently in paroxysmal AF as in permanent AF, especially with multiple risk factors [28]. Moreover, the use of OACs should be based on stroke risk assessment according to the CHA_2_DS_2_-VASc risk score [21]. The pattern of AF seems to be related to patient profiles characterized by age, concomitant diseases, symptoms, and risk factors for stroke and bleeding [13]. Patients with higher European Heart Rhythm Association symptom scores were more often prescribed VKAs than those who were asymptomatic.

Multimorbid AF patients with a history of cardioversion were less often prescribed VKAs than those without prior cardioversion. NOACs were preferred in multimorbid AF patients after cardioversion. A similar pattern was found in another study where rhythm control strategy was associated with selection of NOAC [14].

### OAC prescription in multimorbid AF patients regionally

In this study, multimorbidity influenced ATT use within particular regions. In Europe, younger patients (age <65 years) were less likely to be prescribed OACs than older patients (age ≥75 years). Multimorbid AF patients with congestive HF were more likely to be anticoagulated due to an increased risk of thromboembolism. In Europe, bleeding risk of a patient as perceived by physicians may be the reason for decreased use of anticoagulation. Patients with gastritis or duodenitis or hepatic disease are less likely to be prescribed OACs, probably because of the elevated risk of bleeding. This association has been previously noted [26]. In Asia, younger patients (age <75 years) were more likely to be prescribed OACs than older patients (age ≥75 years). Interestingly, patients with gastritis or duodenitis or a history of cancer were more likely to receive OAC than those without those diseases. In North America, younger multimorbid AF patients (age <65 years) were less likely to be prescribed OACs than older patients (age ≥75 years). Multimorbid AF patients with diabetes were more likely to receive OACs, due to their association with higher thromboembolic risk, as well as higher all-cause, cardiovascular, and noncardiovascular mortality [29]. AF patients with multimorbidity and cancer in North America were less likely to receive OAC.

Asia and North America were associated with decreased OAC prescription. In Asia, OACs are less commonly prescribed in nonvalvular AF patients than in Europe, possibly because of suspicion of the risk of bleeding during treatment [30]. Also, NOACs are not reimbursed in some Asian countries.

### Strengths

It is one the largest prospective global cohort of consecutive AF patients receiving different antithrombotic treatments. Initiation of Phase III was region-specific, once relevant baseline characteristics of patients initiating dabigatran and VKA therapy in Phase II overlapped based on propensity score comparisons. After the baseline visit, all patients in this Phase III were managed according to local clinical practice and were followed for 3 years, regardless of prescribed antithrombotic therapy. This study had regular follow-up with physicians, alongside on-site monitoring, multiple standards for data quality assurance and review.

### Limitations

Although the GLORIA-AF study was designed to capture all outcome events, this analysis did not consider follow-up data. The following limitations exist in our study: we have no data on patient and prescriber treatment preferences; similarly, reasons for OAC nonprescription were not reported. Furthermore, this study reflects single, initial-treatment decisions during a period when prescribing patterns may have been changing, and the analysis was based on prescription pattern shortly after entry into the registry (baseline). Neither have we accounted for quality of anticoagulation or changes in clinical practice patterns over time.

## Conclusion

AF patients with multimorbidity who were prescribed NOACs were relatively healthier, more likely to have paroxysmal AF, and had fewer prevalent comorbidities than AF multimorbid patients on VKAs. Multimorbidity may determine the antithrombotic therapy prescription pattern within AF patients. Several factors are related to increased OAC prescription in multimorbid AF patients, including younger age, hypertension, prior TIA or stroke, and AF ablation. Pattern of AF (paroxysmal and persistent AF), CAD, MI, history of bleeding, and region (Asia, North America) were inversely associated with OAC prescription.

## Supporting information

S1 File (PDF)

S2 File (ODT)
