# Supplementary material for: Atrial fibrillation and comorbidities: Clinical characteristics and antithrombotic treatment in GLORIA-AF
Source: PLoS One. 2021 Apr 14;16(4):e0249524. doi: 10.1371/journal.pone.0249524 (PMC8046191; doi:10.1371/journal.pone.0249524)
Supplement: S1 File — (PDF) [file pone.0249524.s001.pdf]

ARGENTINA				
IRB or IEC (name/address)	IRB or IEC Chairperson	Centre number (5 digit number) / Investigator(s)	Protocol and/or Amendment number(s)	Date of Final Approval (DD/MMM/YYYY)
Comité de Ética en Investigación Instituto Cardiovascular de Buenos Aires / Av. Libertador 6302, C1428DCO, CABA, Buenos Aires Argentina	Alejandro Meretta	21008 / Giniger, Alberto	Protocol version 4.0 7/JUN/2013<br><br>Local amendment version 1.0 15/OCT/2014	11/NOV/2013<br><br>15/DEC/2014
Comité de Ética de Protocolos de Investigación del Hospital Italiano de Buenos Aires / J.D Perón 4190-4192 (1181) Capital Federal, Argentina	Gustavo Izbizky	21009 / Maid, Gustavo	Protocol version 4.0 7/JUN/2013<br><br>Local amendment version 1.0 15/OCT/2014	21/NOV/2013<br><br>07/MAY/2015
Comité Ética de la Investigacion Instituto de Investigaciones Clinicas de Mar del Plata / Av. Colón 3364, B7600FZN Mar del Plata, Buenos	Jesus Vazquez	21012 / Caccavo, Alberto	Protocol version 4.0 7/JUN/2013<br><br>Local amendment version 1.0 15/OCT/2014	24/JAN/2014<br><br>13/MAR/2015

ARGENTINA				
IRB or IEC (name/address)	IRB or IEC Chairperson	Centre number (5 digit number) / Investigator(s)	Protocol and/or Amendment number(s)	Date of Final Approval (DD/MMM/YYYY)
Aires, Argentina				
Comité Independiente de Ética para Ensayos en Farmacología Clinica / Pres. José Evaristo Uriburu 774, C1027 AAP, Buenos Aires, Argentina	Luis Zieher	21013 / Aguinaga, Luis	Protocol version 4.0 7/JUN/2013<br><br>Local amendment version 1.0 15/OCT/2014	22/APR/2014<br><br>02/FEB/2015
Comité de Ética de CER Investigaciones Clínicas / Av. Vicente López 1441 (CP1878), Quilmes Oeste, Bs. As. Argentina.	Patricia Iemma	21014 / Ranieri, Maria Fernanda	Protocol version 4.0 7/JUN/2013 and Local amendment version 1.0 15/OCT/2014	6/MAY/2015
Comité de Ética en Investigación Grupo Gamma / Catamarca 1351, Rosario, Santa Fe, Argentina	Silvia Luján	21015 / Fedele, Jose Luis	Protocol version 4.0 7/JUN/2013 and Local amendment version 1.0 15/OCT/2014	8/JUN/2015
Comité de Ética en Investigación Clinica y Maternidad Suizo	Patricia Saidón	21017 / Ferro, Hugo	Protocol version 4.0 7/JUN/2013 and Local amendment version 1.0	22/MAY/2015

ARGENTINA				
IRB or IEC (name/address)	IRB or IEC Chairperson	Centre number (5 digit number) / Investigator(s)	Protocol and/or Amendment number(s)	Date of Final Approval (DD/MMM/YYYY)
Argentina / Av. Pueyrredón 1486 2° piso (1118) CABA, Argentina			15/OCT/2014	
Comité de Ética San Isidro / Moreno 2950, C1209ABD CABA, Argentina	Mariana Vazquez Durand	21021 / Paz, Analia	Protocol version 4.0 7/JUN/2013 and Local amendment version 1.0 15/OCT/2014	17/SEP/2015
Comité de Ética en Investigación DIM Clinica Privada / Belgrano 136, Ramos Mejia, Buenos Aires, Argentina	Silvia Morales	21023 / Montaña, Oscar Romano	Protocol version 4.0 7/JUN/2013 and Local amendment version 1.0 15/OCT/2014	27/FEB/2016
Comité independiente de ética médica del Noroeste Argentino / Las Piedras 496 (Colegio Médico), 4to Piso. S.M. Tucumán, Argentina	Oscar Gallardo	21024 / Carreño, Susana	Protocol version 4.0 7/JUN/2013 and Local amendment version 1.0 15/OCT/2014	2/FEB/2016
Comité de Ética San Isidro / Av. Libertador 16958, B1643CRO, San Isidro	Mariana Vazquez Durand	21025 / Aiub, Jorge Roberto	Protocol version 4.0 7/JUN/2013 and Local amendment version 1.0 15/OCT/2014	4/FEB/2016

ARGENTINA				
IRB or IEC (name/address)	IRB or IEC Chairperson	Centre number (5 digit number) / Investigator(s)	Protocol and/or Amendment number(s)	Date of Final Approval (DD/MMM/YYYY)
Consejo Institucional de Revision de Estudios de Investigación (CIREI) / Córdoba 4545 - 3º piso - of. Docencia e Investigación, Mar del Plata - República Argentina	Sergio Gonorazky	21026 / Snitman, Marcelo Javier	Protocol version 4.0 7/JUN/2013 and Local amendment version 1.0 15/OCT/2014	20/APR/2016
Comité de Bioética del Instituto de Investigaciones Clínicas Provincia de Rosario / Paraguay 160, Rosario, Argentina	Beatriz Martinelli	21027 / Abud, Atilio Marcelo	Protocol version 4.0 7/JUN/2013 and Local amendment version 1.0 15/OCT/2014	1/JUN/2016
Comité Independiente de Ética para Ensayos en Farmacología Clinica / Pte. J. E. Uriburu 774 1º Piso, Ciudad Autónoma de Buenos Aires (C1027AAP), Argentina	Luis Zieher	21028 / Villamil, Alejandro Mario	Protocol version 4.0 7/JUN/2013 and Local amendment version 1.0 15/OCT/2014	12/APR/2016

AUSTRIA				
IRB or IEC (name/address)	IRB or IEC Chairperson	Centre number (5 digit number) / Investigator(s)	Protocol and/or Amendment number(s)	Date of Final Approval (DD/MMM/YYYY)
Ethics Committee of the Medical University of Vienna<br>Borschkegasse 8b/E06<br>1090 Wien	Univ.- Prof. Dr. Ernst Singer	34001, 34002, 34003, 34004, 34005, 34006, 34007, 34008*	Final Protocol	30 Jan 2013
Ethics Committee of the Medical University of Vienna<br>Borschkegasse 8b/E06<br>1090 Wien	Univ.- Prof. Dr. Ernst Singer	34001, 34003, 34004, 34006, 34007, 34008, 34011	Global Amendment 1	22 Jan 2015
Medizinische Universität Wien, Klinische Abteilung für Hämatologie und Hämostase	Frau Univ.-Prof. Dr. Sabine Eichinger-Hasenauer	34001, 34003, 34006, 34008, 34011	Global Amendment 1	22 Jan 2015

*EC statement that for a NIS: EC does not need to be notified in case of new centers or PI changes.

BELGIUM				
IRB or IEC (name/address)	IRB or IEC Chairperson	Centre number (5 digit number) / Investigator(s)	Protocol and/or Amendment number(s)	Date of Approval (DD/MMM/YYYY)
Comité d'Éthique<br>Route de Lennik 808<br>1070 Brussels	Christian Melot, Prof	58001 / Goethals, Dr. Peter<br>58002 / Hoffer, Dr. Etienne<br>58003 / Vincent, Dr. Marc<br>58004 / van de Borne, Prof. Philippe<br>58005 / Maqueda, Dr. Vicky<br>58006 / De Wolf, Dr. Luc<br>58007 / Rutgers, Dr. Matthieu<br>58008 / Pieters, Dr. Denis<br>58009 / Nuyens, Dr. Dieter	Protocol version 3.0 23 March 2012<br>Protocol version 4.0 22 Oct 2014	18/MAR/2014<br>16/DEC/2014

BRAZIL				
IRB or IEC (name/address)	IRB or IEC Chairperson	Centre number (5 digit number) / Investigator(s)	Protocol and/or Amendment number(s)	Date of Final Approval (DD/MMM/YYYY)
Comitê de Ética em Pesquisa do Instituto de Moléstias Cardiovasculares (IMC) / Rua Castelo D'Água, 3030 - Redentora - São José do Rio Preto - SP - CEP 15015-210	Dr. Adelino Parro Junior	22001 / Menezes Lorga Filho, Adalberto	Protocol version 3.0 19/JUL/2011	14/FEB/2012
			Protocol version 4.0 7/JUN/2013	30/OCT/2013
			Local amendment version 1.0 15/OCT/2014	15/JUL/2015
Comitê de Ética em Pesquisa da Universiade Federal de São Paulo - UNIFESP/EPM / Rua Botucatu, 572 Conj 14 -	Dr. Alberto Duarte	22003 / Vincenzo de Paola, Ângelo Amato	Protocol version 3.0 19/JUL/2011	24/AUG/2012

BRAZIL				
IRB or IEC (name/address)	IRB or IEC Chairperson	Centre number (5 digit number) / Investigator(s)	Protocol and/or Amendment number(s)	Date of Final Approval (DD/MMM/YYYY)
Vila Clementino - São Paulo - SP - CEP 04023-06			Protocol version 4.0 7/JUN/2013	2/JUL/2015
Comitê de Ética em Pesquisa da Universiade Federal de São Paulo - UNIFESP/EPM / Rua Botucatu, 572 Conj 14 - Vila Clementino - São Paulo - SP - CEP 04023-06 Comitê de Ética em Pesquisa do Instituto de Cardiologia - ICDF / Estrada Parque Contorno do Bosque, s/n, Cruzeiro Novo	Dr. Alberto Duarte Dr. Guilherme Urpia Monte	22003 / Vincenzo de Paola, Ângelo Amato	Local amendment version 1.0 15/OCT/2014	3/SEP/2015
			Protocol version 3.0 19/JUL/2011	19/DEC/2012

BRAZIL				
IRB or IEC (name/address)	IRB or IEC Chairperson	Centre number (5 digit number) / Investigator(s)	Protocol and/or Amendment number(s)	Date of Final Approval (DD/MMM/YYYY)
- Brasília - DF - CEP 70658-700			Protocol version 4.0 7/JUN/2013	6/MAY/2014
Comitê de Ética em Pesquisa do Instituto Dante Pazzanese de Cardiologia / Avenida Dr. Dante Pazzanese, 500 - Ibirapuera - São Paulo - SP - CEP 04012-909<br>Comitê de Ética em Pesquisa Hospital Vera Cruz / Rua Timbiras, 3156 - 9º andar - Sala 902 - Bairro Preto - Belo Horizonte - MG - CEP 30140-062	Abílio Augusto Fragata Filho<br>Dr. Carlos Ernesto Ferreira Starling	22009 / Ribeiro Moreira, Dalmo Antonio<br>22011 / Carvalho Neuenschwander, Fernando	Local amendment version 1.0 15/OCT/2014 and Local amendment version 1.0 15/OCT/2014	17/MAR/2015
			Protocol version 3.0 19/JUL/2011	9/JAN/2011

BRAZIL

IRB or IEC (name/address)	IRB or IEC Chairperson	Centre number (5 digit number) / Investigator(s)	Protocol and/or Amendment number(s)	Date of Final Approval (DD/MMM/YYYY)
			Protocol version 4.0 7/JUN/2013	18/DEC/2013
Comitê de Ética em Pesquisa Hospital Vera Cruz / Rua Timbiras, 3156 - 9º andar - Sala 902 - Bairro Preto - Belo Horizonte - MG - CEP 30140-062<br>Comitê de Ética em Pesquisa da PUC do Paraná / Rua Imaculada Conceição, 1155 - Bairro Prado Velho - Prédio Administrativo - 6º andar - Curitiba - PR - CEP 80215-901	Dr. Carlos Ernesto Ferreira Starling<br>Prof. MSc. Naim Akel Filho	22011 / Carvalho Neuenschwander, Fernando<br>22016 / Moura, Jorge José Carlos	Local amendment version 1.0 15/OCT/2014	12/AUG/2015
			Protocol version 3.0 19/JUL/2011	27/JUN/2012
Comitê de Ética em Pesquisa do Instituto Dante Pazzanese de Cardiologia / Avenida Dr. Dante Pazzanese, 500 - Ibirapuera	Abílio Augusto Fragata Filho<br>Prof. MSc. Naim Akel Filho<br>Prof. Dr. David Bianchini	22016 / Moura, Jorge José Carlos<br>22017 / Saraiva, José Francisco Kerr	Protocol version 4.0 7/JUN/2013	11/MAR/2015

BRAZIL				
IRB or IEC (name/address)	IRB or IEC Chairperson	Centre number (5 digit number) / Investigator(s)	Protocol and/or Amendment number(s)	Date of Final Approval (DD/MMM/YYYY)
- São Paulo - SP - CEP 04012-909<br>Comitê de Ética em Pesquisa da PUC do Paraná / Rua Imaculada Conceição, 1155 - Bairro Prado Velho - Prédio Administrativo - 6º andar - Curitiba - PR - CEP 80215-901<br>Comitê de Ética em Pesquisa PUC de Campinas / Avenida John Boyd Dunlop, s/n - Campinas - SP - CEP 13059-740			Local amendment version 1.0 15/OCT/2014	14/OCT/2015
Comitê de Ética em Pesquisa do Instituto Dante Pazzanese de Cardiologia / Avenida Dr. Dante Pazzanese, 500 - Ibirapuera	Abílio Augusto Fragata Filho<br>Dr. Carlos Ernesto Ferreira Starling	22011 / Carvalho Neuenschwander, Fernando<br>22017 / Saraiva, José	Protocol version 3.0 19/JUL/2011	3/SEP/2012

BRAZIL				
IRB or IEC (name/address)	IRB or IEC Chairperson	Centre number (5 digit number) / Investigator(s)	Protocol and/or Amendment number(s)	Date of Final Approval (DD/MMM/YYYY)
- São Paulo - SP - CEP 04012-909<br>Comitê de Ética em Pesquisa Hospital Vera Cruz / Rua Timbiras, 3156 - 9º andar - Sala 902 - Bairro Preto - Belo Horizonte - MG - CEP 30140-062<br>Comitê de Ética em Pesquisa PUC de Campinas / Avenida John Boyd Dunlop, s/n - Campinas - SP - CEP 13059-740<br>Comitê de Ética em Pesquisa da Casa de Saúde Santa Marcelina - Itaquera - SP / Rua Santa Marcelina, 177 - Itaquera - São Paulo - SP - CEP 08270-070	Prof. Dr. David Bianchini<br>Dr. Osmar José Moraes	Francisco Kerr	Protocol version 4.0 7/JUN/2013	12/DEC/2013
			Local amendment version 1.0 15/OCT/2014	8/OCT/2015

BRAZIL				
IRB or IEC (name/address)	IRB or IEC Chairperson	Centre number (5 digit number) / Investigator(s)	Protocol and/or Amendment number(s)	Date of Final Approval (DD/MMM/YYYY)
Comitê de Ética em Pesquisa Hospital Vera Cruz / Rua Timbiras, 3156 - 9º andar - Sala 902 - Bairro Preto - Belo Horizonte - MG - CEP 30140-062<br>Comitê de Ética em Pesquisa da PUC do Paraná / Rua Imaculada Conceição, 1155 - Bairro Prado Velho - Prédio Administrativo - 6º andar - Curitiba - PR - CEP 80215-901<br>Comitê de Ética em Pesquisa da Universidade de Passo Fundo / Universidade de Passo Fundo - CAMPUS I - Km 171 - BR 285 - Passo Fundo - RS	Dr. Carlos Ernesto Ferreira Starling<br>Prof. MSc. Naim Akel Filho<br>Prof. Nadir Antonio Pichler	22011 / Carvalho Neuenschwander, Fernando<br>22016 / Moura, Jorge José Carlos<br>22021 / Backes, Luciano Marcelo	Protocol version 3.0 19/JUL/2011	30/JUL/2012
			Protocol version 4.0 7/JUN/2013	26/FEB/2014
			Protocol version 3.0 19/JUL/2011	28/JUN/2012

BRAZIL				
IRB or IEC (name/address)	IRB or IEC Chairperson	Centre number (5 digit number) / Investigator(s)	Protocol and/or Amendment number(s)	Date of Final Approval (DD/MMM/YYYY)
Comitê de Ética em Pesquisa da PUC do Paraná / Rua Imaculada Conceição, 1155 - Bairro Prado Velho - Prédio Administrativo - 6º andar - Curitiba - PR - CEP 80215-901<br>Comitê de Ética em Pesquisa PUC de Campinas / Avenida John Boyd Dunlop, s/n - Campinas - SP - CEP 13059-740<br>Comitê de Ética em Pesquisa da Universidade de Passo Fundo / Universidade de Passo Fundo - CAMPUS I - Km 171 - BR 285 - Passo Fundo - RS<br>Comitê de Ética em Pesquisa do IC/DF / Estrada Parque Contorno do	Prof. MSc. Naim Akel Filho<br>Prof. Dr. David Bianchini<br>Prof. Nadir Antonio Pichler<br>Dr. Guilherme Urpia Monte	22016 / Moura, Jorge José Carlos<br>22017 / Saraiva, José Francisco Kerr<br>22021 / Backes, Luciano Marcelo<br>22022 / Gome Ferreira, Luis Gustavo	Protocol version 4.0 7/JUN/2013	26/NOV/2013
			Local amendment version 1.0 15/OCT/2014	25/SEP/2015
			Protocol version 3.0 19/JUL/2011	21/MAR/2013

BRAZIL				
IRB or IEC (name/address)	IRB or IEC Chairperson	Centre number (5 digit number) / Investigator(s)	Protocol and/or Amendment number(s)	Date of Final Approval (DD/MMM/YYYY)
Bosque, s/n - Cruzeiro Novo - Brasília - DF - CEP 70658-90				
Comitê de Ética em Pesquisa PUC de Campinas / Avenida John Boyd Dunlop, s/n - Campinas - SP - CEP 13059-740<br>Comitê de Ética em Pesquisa da Casa de Saúde Santa Marcelina - Itaquera - SP / Rua Santa Marcelina, 177 - Itaquera - São Paulo - SP - CEP 08270-070<br>Comitê de Ética em Pesquisa do IC/DF / Estrada Parque Contorno do Bosque, s/n - Cruzeiro Novo - Brasília - DF - CEP 70658-90<br>Comitê de Ética em	Prof. Dr. David Bianchini<br>Dr. Osmar José Moraes<br>Dr. Guilherme Urpia Monte<br>Bruno da Costa Rocha	22017 / Saraiva, José Francisco Kerr<br>22022 / Gome Ferreira, Luis Gustavo<br>22023 / Fonteles Ritt, Luiz Eduardo	Protocol version 4.0 7/JUN/2013	24/JUL/2014
			Protocol version 4.0 7/JUN/2013	26/APR/2015
			Local amendment version 1.0 15/OCT/2014	21/NOV/2015

BRAZIL				
IRB or IEC (name/address)	IRB or IEC Chairperson	Centre number (5 digit number) / Investigator(s)	Protocol and/or Amendment number(s)	Date of Final Approval (DD/MMM/YYYY)
Pesquisa Prof. Dr. Celso Figueirôa - Hospital Santa Izabel / Praça Conselheiro Almeida Couto, 500 - Nazaré - Salvador - BA - CEP 40050-410				
Comitê de Ética em Pesquisa da Universidade de Passo Fundo / Universidade de Passo Fundo - CAMPUS I - Km 171 - BR 285 - Passo Fundo - RS	Prof. Nadir Antonio Pichler	22021 / Backes, Luciano Marcelo	Protocol version 3.0 19/JUL/2011	20/NOV/2012
			Protocol version 4.0 7/JUN/2013	12/MAR/2015
Comitê de Ética em Pesquisa Prof. Dr. Celso Figueirôa - Hospital Santa Izabel / Praça Conselheiro Almeida Couto, 500 -	Bruno da Costa Rocha<br>Alice Cristina Oliveira Alves	22023 / Fonteles Ritt, Luiz Eduardo	Local amendment version 1.0 15/OCT/2014	5/JUL/2015

BRAZIL				
IRB or IEC (name/address)	IRB or IEC Chairperson	Centre number (5 digit number) / Investigator(s)	Protocol and/or Amendment number(s)	Date of Final Approval (DD/MMM/YYYY)
Nazaré - Salvador - BA - CEP 40050-410<br>Comitê de Ética em Pesquisa e Ensino do Centro Universitário CESMAC - COEPE / Rua Cônego Machado, 918 - Farol - Maceio - Alagoas - CEP 57051-160			Protocol version 3.0 19/JUL/2011	12/DEC/2012
			Protocol version 4.0 7/JUN/2013	19/AUG/2015
Comitê de Ética em Pesquisa da UNIFEV - Centro Universitário de Votuporanga / Rua Pernambuco, 4196 - Centro - Votuporanga - SP - CEP	Prof. Dr. Rogério Rocha Matarucco<br>Prof. Dr. Rogério Rocha Matarucco<br>Dr. Leonardo Pires	22025 / Esteves Hernandes, Mauro<br>22025 / Esteves Hernandes, Mauro	Protocol version 3.0 19/JUL/2011	14/FEB/2013

BRAZIL				
IRB or IEC (name/address)	IRB or IEC Chairperson	Centre number (5 digit number) / Investigator(s)	Protocol and/or Amendment number(s)	Date of Final Approval (DD/MMM/YYYY)
15500-006<br>Comitê de Ética em Pesquisa da UNIFEV - Centro Universitário de Votuporanga / Rua Pernambuco, 4196 - Centro - Votuporanga - SP - CEP 15500-006<br>Comitê de Ética em Pesquisa do Instituto de Cardiologia do RS - IC-FUC / Avenida Princesa Isabel, 395 - Bairro Santa - Porto Alegre - RS - CEP 90620-000			Protocol version 4.0 7/JUN/2013	7/JAN/2015
			Local amendment version 1.0 15/OCT/2014	16/JUL/15
			Protocol version 3.0 19/JUL/2011	23/MAR/2012
Comitê de Ética em Pesquisa Prof. Dr. Celso Figueirôa - Hospital Santa	Bruno da Costa Rocha<br>Alice Cristina Oliveira Alves	22023 / Fonteles Ritt, Luiz Eduardo<br>22029 / Zimmermann,	Protocol version 4.0 7/JUN/2013	16/OCT/2014

<table>
<tr><th colspan="5">BRAZIL</th></tr>
<tr><th>IRB or IEC (name/address)</th><th>IRB or IEC Chairperson</th><th>Centre number (5 digit number) / Investigator(s)</th><th>Protocol and/or Amendment number(s)</th><th>Date of Final Approval (DD/MMM/YYYY)</th></tr>
<tr><td rowspan="2">Izabel / Praça Conselheiro Almeida Couto, 500 - Nazaré - Salvador - BA - CEP 40050-410
Comitê de Ética em Pesquisa e Ensino do Centro Universitário CESMAC - COEPE / Rua Cônego Machado, 918 - Farol - Maceio - Alagoas - CEP 57051-160
Comitê de Ética em Pesquisa em Seres Humanos da Universidade Regional de Blumenau / Campus I - Central - Rua Antônio da Veiga, 140 - Victor Konder - Blumenau - SC - CEP 89012-900</td><td rowspan="2">Profa. Dra. Mercedes Gabriela Ratto Reiter</td><td rowspan="2">Sérgio</td><td>Protocol version 3.0 19/JUL/2011</td><td>12/JUN/2012</td></tr>
<tr><td>Protocol version 4.0 7/JUN/2013</td><td>13/DEC/2013</td></tr>
</table>

BRAZIL				
IRB or IEC (name/address)	IRB or IEC Chairperson	Centre number (5 digit number) / Investigator(s)	Protocol and/or Amendment number(s)	Date of Final Approval (DD/MMM/YYYY)
Comitê de Ética em Pesquisa em Seres Humanos da Universidade Regional de Blumenau / Campus I - Central - Rua Antônio da Veiga, 140 - Victor Konder - Blumenau - SC - CEP 89012-900<br>Comitê de Ética em Pesquisa da FMABC / Av. Príncipe de Gales, 821 - Santo André - SP - CEP 09060-650	Profa. Dra. Mercedes Gabriela Ratto Reiter<br>Prof. Dra. Marcia Tamosauskas	22029 / Zimmermann, Sérgio<br>22032 / Saporito, Wladmir Faustino	Protocol version 4.0 7/JUN/2013	18/DEC/2014
			Local amendment version 1.0 15/OCT/2014	23/JUL/2015
Comitê de Ética em Pesquisa da UNIFEV - Centro Universitário de Votuporanga / Rua Pernambuco, 4196 - Centro	Prof. Dr. Rogério Rocha Matarucco<br>Dr. Leonardo Pires<br>Prof. Dra. Marcia	22025 / Esteves Hernandes, Mauro<br>22032 / Saporito, Wladmir Faustino	Protocol version 3.0 19/JUL/2011	13/SEP/2012

BRAZIL				
IRB or IEC (name/address)	IRB or IEC Chairperson	Centre number (5 digit number) / Investigator(s)	Protocol and/or Amendment number(s)	Date of Final Approval (DD/MMM/YYYY)
- Votuporanga - SP - CEP 15500-006<br>Comitê de Ética em Pesquisa do Instituto de Cardiologia do RS - IC-FUC / Avenida Princesa Isabel, 395 - Bairro Santa - Porto Alegre - RS - CEP 90620-000<br>Comitê de Ética em Pesquisa da FMABC / Av. Príncipe de Gales, 821 - Santo André - SP - CEP 09060-650<br>Comitê de Ética em Pesquisa Hospital Copa D´or / Rua Figueiredo Magalães, 875 - Rio de Janeiro - RJ - CEP 22031-010	Tamosauskas<br>Denilson Campos de Albuquerque	22033/ Ferreira de Souza, Olga	Corection letter regarding IRB Approval letter dated of 13-Sep-12	21/NOV/2012
			Local amendment version 1.0 15/OCT/2014	21/AUG/2015

BRAZIL				
IRB or IEC (name/address)	IRB or IEC Chairperson	Centre number (5 digit number) / Investigator(s)	Protocol and/or Amendment number(s)	Date of Final Approval (DD/MMM/YYYY)
Comitê de Ética em Pesquisa em Seres Humanos da Universidade Regional de Blumenau / Campus I - Central - Rua Antônio da Veiga, 140 - Victor Konder - Blumenau - SC - CEP 89012-900	Profa. Dra. Mercedes Gabriela Ratto Reiter	22029 / Zimmermann, Sérgio	Protocol version 3.0 19/JUL/2011	18/DEC/2012
			Protocol version 4.0 7/JUN/2013	17/JUN/2015

BRAZIL				
IRB or IEC (name/address)	IRB or IEC Chairperson	Centre number (5 digit number) / Investigator(s)	Protocol and/or Amendment number(s)	Date of Final Approval (DD/MMM/YYYY)
Comitê de Ética em Pesquisa Hospital Copa D´or / Rua Figueiredo Magalães, 875 - Rio de Janeiro - RJ - CEP 22031-010<br>Comitê de Ética em Pesquisa do Instituto Nacional de Cardiologia / Rua das Laranjeiras, 374 - Rio de Janeiro - RJ - CEP 22240-00<br>Comitê de Ética em Pesquisa do Instituto Nacional de Cardiologia / Rua das Laranjeiras, 374 - Rio de Janeiro - RJ - CEP 22240-00	Denilson Campos de Albuquerque<br>Carla de Almeida<br>Carla de Almeida<br>Patrick Josué Mezzomo	22033/ Ferreira de Souza, Olga<br>22035 / Scherr, Carlos<br>22035 / Scherr, Carlos<br>22039 / Horbach, Stevie Jorge	Local amendment version 1.0 15/OCT/2014	1/SEP/2015
			Protocol version 3.0 19/JUL/2011	6/FEB/2013
			Protocol version 4.0 7/JUN/2013	30/OCT/2013

BRAZIL				
IRB or IEC (name/address)	IRB or IEC Chairperson	Centre number (5 digit number) / Investigator(s)	Protocol and/or Amendment number(s)	Date of Final Approval (DD/MMM/YYYY)
Comitê de Ética em Pesquisa do Hospital Pompéia / Avenida Júlio de Castilhos, 2163 - Centro - Caxias do Sul - RS - CEP 95010-005			Local amendment version 1.0 15/OCT/2014	8/JUL/2015
Comitê de Ética em Pesquisa da FMABC / Av. Príncipe de Gales, 821 - Santo André - SP - CEP 09060-650<br>Comitê de Ética em Pesquisa Hospital Copa D´or / Rua Figueiredo Magalães, 875 - Rio de Janeiro - RJ - CEP 22031-010<br>Comitê de Ética em Pesquisa Grupo Investiga - Instituto de Pesquisa / Avenida Romeu Tortima, 739 - Campinas - SP - CEP	Prof. Dra. Marcia Tamosauskas<br>Denilson Campos de Albuquerque<br>Cristiana Madjarof<br>Juarez Andrade	22032 / Saporito, Wladmir Faustino<br>22033/ Ferreira de Souza, Olga<br>22041 / Rossi dos Santos, Fábio<br>22042 / Deway Andrade Dracoulakis, Marianna	Protocol version 4.0 7/JUN/2013	22/DEC/2014
			Local amendment version 1.0 15/OCT/2014	28/AUG/2015
			Protocol version 4.0 7/JUN/2013 and Local amendment version 1.0 15/OCT/2014	3/MAR/2016

BRAZIL				
IRB or IEC (name/address)	IRB or IEC Chairperson	Centre number (5 digit number) / Investigator(s)	Protocol and/or Amendment number(s)	Date of Final Approval (DD/MMM/YYYY)
13084-791<br>Comitê de Ética em Pesquisa do Hospital da Bahia / Avenida Prof. Magalhães Neto, 1541 - Pituba - Salvador - BA - CEP - 41820-011				
Comitê de Ética em Pesquisa Hospital Copa D´or / Rua Figueiredo Magalães, 875 - Rio de Janeiro - RJ - CEP 22031-010<br>Comitê de Ética em Pesquisa do Instituto Nacional de Cardiologia / Rua das Laranjeiras, 374 - Rio de Janeiro - RJ - CEP 22240-00<br>Comitê de Ética em	Denilson Campos de Albuquerque<br>Carla de Almeida<br>Dr. José Carlos Costa Baptista Silva<br>Wanessa Miranda	22033/ Ferreira de Souza, Olga<br>22035 / Scherr, Carlos<br>22043 / Alves da Costa, Fernando Augusto<br>22044 / Noronha Campos, Rodrigo	Protocol version 4.0 7/JUN/2013 and #Local Amendment, version 1.0 dated 15Oct14	19/MAY/2016
			#Protocol version 4.0 dated 07Jun13 and Local amendment version 1.0 15/OCT/2014	29/JUN/2016

BRAZIL				
IRB or IEC (name/address)	IRB or IEC Chairperson	Centre number (5 digit number) / Investigator(s)	Protocol and/or Amendment number(s)	Date of Final Approval (DD/MMM/YYYY)
Pesquisa do Hospital Samaritano / Rua Conselheiro Brotero, 1486 - Higienópolis - São Paulo - SP - CEP 01232-010<br>Comitê de Ética em Pesquisa do Hospital Beneficiência Portuguesa de São Paulo / Rua Maestro Cardim, 769 - Térreo - Bloco 5 - Bela Vista - São Paulo - SP - CEP 01323-90			Protocol version 4.0 7/JUN/2013 and Local amendment version 1.0 15/OCT/2014	30/MAY/2016
Comitê de Ética em Pesquisa em Seres Humanos - Complexo Hospitalar HUOC/PROCAPE / Rua Arnóbio Marques, 310 - Santo Amaro - Recife - PE -	Magaly Bushatsky<br>Dr. André Márcio Murad<br>Ilóide de Fátima dos Santos Bittencourt	22045 / Sobral Filho, Dário Celestino<br>22046 / Lanna Figueiredo, Estevão<br>22047 / Bertolim Precoma, Dalton	Protocol version 4.0 7/JUN/2013 and Local amendment version 1.0 15/OCT/2014	4/JUL/2016

BRAZIL				
IRB or IEC (name/address)	IRB or IEC Chairperson	Centre number (5 digit number) / Investigator(s)	Protocol and/or Amendment number(s)	Date of Final Approval (DD/MMM/YYYY)
CEP 50100-130<br>Comitê de Ética em Pesquisa do Hospital Lifecenter / Avenida do contorno, 4747 - 20º andar - Serra - Belo Horizonte - MG - CEP 30110-090<br>Comitê de Ética em Pesquisa Sociedade Hospitalar Angelina Caron - Rodovia do Caqui 1150 - Recanto Verde - Campina Grande do Sul - PR - CEP 83430-000			Protocol version 4.0 7/JUN/2013 and Local amendment version 1.0 15/OCT/2014	2/JUN/2016
			Protocol version 4.0 7/JUN/2013 and Local amendment version 1.0 15/OCT/2014	27/MAY/2016
Comitê de Ética em Pesquisa do Incor - HCFMUSP / Avenida Dr. Arnaldo, 251 - 21º andar - Sala 36 - Cerqueira César - São Paulo - SP - CEP 01246-000	Prof. Dr. José Eduardo Krieger<br>José Mário Coelho Moares	22048 / da Costa Darrieux, Francisco Carlos<br>22049 / Sebba de Souza, Weimar Kunz	Protocol version 4.0 7/JUN/2013 Local amendment version 1.0 15/OCT/2014	15/AUG/2016

BRAZIL				
IRB or IEC (name/address)	IRB or IEC Chairperson	Centre number (5 digit number) / Investigator(s)	Protocol and/or Amendment number(s)	Date of Final Approval (DD/MMM/YYYY)
Comitê de Ética em Pesquisa Humana do Hospital das Clínicas da Universidade Federal de Goiás / 1ª Avenida s/n - Setor Leste Universitário - Goiânia - GO - CEP 74605-050			Protocol version 4.0 7/JUN/2013<br>Local amendment version 1.0 15/OCT/2014	16/JUN/2016

BULGARIA				
IRB or IEC (name/address)	IRB or IEC Chairperson	Centre number (5 digit number) / Investigator(s)	Protocol and/or Amendment number(s)	Date of Final Approval (DD/MMM/YYYY)
Ethichs Commmittee for Multicentre Trials<br>5, Sveta Nedelya Square<br>1000 Sofia, Bulgaria	Prof. Todor Popov, MD	36001 Lachezar Smilov, MD, PI<br>36002 Sidiqullah Rahimi, MD, PI<br>36003 Stefan Naydenov, MD, PI<br>36004 Ivaneta Ayryanova, MD, PI<br>36005 Penka Kamenova, MD, PI<br>36006 Margarita Mihneva, MD, PI<br>36007 Assoc. Prof. Elena Kinova, PI<br>36008 Dimo Vasilev, MD, PI<br>36009 Diana Smilkova, MD, PI<br>36011 Prof. Dimitar Raev, MD, PI<br>36012 Anastas Stoikov, MD PI	Protocol version 3.0 23/Mar/2012	01/Mar/2013

BULGARIA				
IRB or IEC (name/address)	IRB or IEC Chairperson	Centre number (5 digit number) / Investigator(s)	Protocol and/or Amendment number(s)	Date of Final Approval (DD/MMM/YYYY)
		36013 Silviya Syulemezova, MD, PI<br>36014 Assoc. Prof. Elina Trendafilova, MD, PI<br>36015 Assoc. Prof. Yoto Yotov, MD, PI		
Ethichs Commmittee for Multicentre Trials<br>5, Sveta Nedelya Square<br>1000 Sofia, Bulgaria	Prof. Todor Popov, MD	36001 Lachezar Smilov, MD, PI<br>36002 Sidiqullah Rahimi, MD, PI<br>36003 Stefan Naydenov, MD, PI<br>36004 Ivaneta Ayryanova, MD, PI<br>36005 Penka Kamenova, MD, PI<br>36006 Margarita Mihneva, MD, PI<br>36007 Assoc. Prof. Elena Kinova, PI<br>36008 Dimo Vasilev, MD, PI	Protocol version 4.0 22/Oct/2014	01/Apr/2015

BULGARIA				
IRB or IEC (name/address)	IRB or IEC Chairperson	Centre number (5 digit number) / Investigator(s)	Protocol and/or Amendment number(s)	Date of Final Approval (DD/MMM/YYYY)
		36009 Diana Smilkova, MD, PI<br>36011 Prof. Dimitar Raev, MD, PI<br>36012 Anastas Stoikov, MD PI<br>36013 Silviya Syulemezova, MD, PI<br>36014 Assoc. Prof. Elina Trendafilova, MD, PI<br>36015 Assoc. Prof. Yoto Yotov, MD, PI		
LEC at Multiprofile Hospital for Active Treatment "Akta Medika", Sevlievo<br>60, Nikola Petkov St., 5400 Sevlievo, Bulgaria	Dr. Stanka Kalcheva	36001 Lachezar Smilov, MD, PI	Protocol version 3.0 23/Mar/2012	NA (Only notification required)
LEC at Multiprofile Hospital for Active Treatment "Akta	Dr. Stanka Kalcheva	36001 Angel Angelov, MD, PI	Protocol version 4.0 22/Oct/2014	NA (Only notification required)

BULGARIA				
IRB or IEC (name/address)	IRB or IEC Chairperson	Centre number (5 digit number) / Investigator(s)	Protocol and/or Amendment number(s)	Date of Final Approval (DD/MMM/YYYY)
Medika", Sevlievo<br>60, Nikola Petkov St., 5400 Sevlievo, Bulgaria				
LEC at Multiprofile Hospital for Active Treatment "Dr. Hristo Stambolski", Kazanlak<br>16, Starozagorska St., 6100 Kazanlak, Bulgaria	Dr. Tanya Arabadjieva	36002 Sidiqullah Rahimi, MD, PI	Protocol version 3.0 23/Mar/2012	NA (Only notification required)
LEC at Multiprofile Hospital for Active Treatment "Dr. Hristo Stambolski", Kazanlak<br>16, Starozagorska St., 6100 Kazanlak, Bulgaria	Dr. Tanya Arabadjieva	36002 Sidiqullah Rahimi, MD, PI	Protocol version 4.0 22/Oct/2014	NA (Only notification required)
LEC at University Multiprofile Hospital for Active Treatment "Aleksandrovska", Sofia<br>1, Georgi Sofiisky Str.., 1431 Sofia, Bulgaria	Assoc. Prof. Rumyana Tarnnovska	36003 Stefan Naydenov, MD, PI	Protocol version 3.0 23/Mar/2012	NA (Only notification required)

BULGARIA				
IRB or IEC (name/address)	IRB or IEC Chairperson	Centre number (5 digit number) / Investigator(s)	Protocol and/or Amendment number(s)	Date of Final Approval (DD/MMM/YYYY)
LEC at University Multiprofile Hospital for Active Treatment "Aleksandrovska", Sofia<br>1, Georgi Sofiisky Str.., , 1431 Sofia, Bulgaria	Assoc. Prof. Rumyana Tarnnovska	36003 Stefan Naydenov, MD, PI	Protocol version 4.0 22/Oct/2014	NA (Only notification required)
LEC at Multiprofile Hospital for Active Treatment "Deva Maria", Burgas<br>Vetren district, Alexander Stamboliyski St., 5800 Burgas, Bulgaria	Dr. Sneja Zgurova	36004 Ivaneta Ayryanova, MD, PI	Protocol version 3.0 23/Mar/2012	NA (Only notification required)
LEC at Multiprofile Hospital for Active Treatment "Deva Maria", Burgas<br>Vetren district, Alexander Stamboliyski St., 5800 Burgas, Bulgaria	Dr. Sneja Zgurova	36004 Ivaneta Ayryanova MD, PI	Protocol version 4.0 22/Oct/2014	NA (Only notification required)
LEC at Multiprofile	Dr. Grigoriy Lefterov	36005 Penka Kamenova,	Protocol version 3.0	NA (Only notification

BULGARIA				
IRB or IEC (name/address)	IRB or IEC Chairperson	Centre number (5 digit number) / Investigator(s)	Protocol and/or Amendment number(s)	Date of Final Approval (DD/MMM/YYYY)
Hospital for Active Treatment, Ruse<br>2, Nezavisimost St., 7002 Ruse, Bulgaria		MD, PI	23/Mar/2012	required)
LEC at Multiprofile Hospital for Active Treatment, Ruse<br>2, Nezavisimost St., 7002 Ruse, Bulgaria	Dr. Grigoriy Lefterov	36005 Penka Kamenova, MD, PI	Protocol version 4.0 22/Oct/2014	NA (Only notification required)
LEC at Specialized Hospital for Active Treatment in Cardiology, Yambol<br>69, Dimitar Blagoev St., 8600 Yambol, Bulgaria	Dr. Bistra Pesheva-Musievska	36006 Margarita Mihneva, MD, PI	Protocol version 3.0 23/Mar/2012	NA (Only notification required)
LEC at Specialized Hospital for Active Treatment in Cardiology, Yambol<br>69, Dimitar Blagoev St.,	Dr. Bistra Pesheva-Musievska	36006 Margarita Mihneva, MD, PI	Protocol version 4.0 22/Oct/2014	NA (Only notification required)

BULGARIA				
IRB or IEC (name/address)	IRB or IEC Chairperson	Centre number (5 digit number) / Investigator(s)	Protocol and/or Amendment number(s)	Date of Final Approval (DD/MMM/YYYY)
8600 Yambol, Bulgaria				
LEC at University Multiprofile Hospital for Active Treatment "Tsaritsa Yoanna – ISUL", Sofia<br>8, Byalo More St., 1527 Sofia, Bulgaria	Assoc. Prof. Borislav Dimitrov	36007 Assoc. Prof. Elena Kinova, PI	Protocol version 3.0 23/Mar/2012	NA (Only notification required)
LEC at University Multiprofile Hospital for Active Treatment "Tsaritsa Yoanna – ISUL", Sofia<br>8, Byalo More St., 1527 Sofia, Bulgaria	Assoc. Prof. Borislav Dimitrov	36007 Assoc. Prof. Elena Kinova, PI	Protocol version 4.0 22/Oct/2014	NA (Only notification required)
LEC at University Multiprofile Hospital for Active Treatment "Sveti Georgi", 15A Vasil Aprilov Blvd.,., 4002 Plovdiv, Bulgaria	Assoc. Prof. Janet Grudeva	36008 Dimo Vasilev, MD, PI	Protocol version 3.0 23/Mar/2012	NA (Only notification required)

BULGARIA				
IRB or IEC (name/address)	IRB or IEC Chairperson	Centre number (5 digit number) / Investigator(s)	Protocol and/or Amendment number(s)	Date of Final Approval (DD/MMM/YYYY)
LEC at University Multiprofile Hospital for Active Treatment “Sveti Georgi”, 15A Vasil Aprilov Blvd., 4002 Plovdiv, Bulgaria	Assoc. Prof. Janet Grudeva	36008 Dimo Vasilev, MD, PI	Protocol version 4.0 22/Oct/2014	NA (Only notification required)
LEC at University Multiprofile Hospital of Active Treatment "Prof. Stoyan Kirkovich" Stara Zagora 11, Armeiska St., 6003 Stara Zagora, Bulgaria	Dr. Valkova	36009 Diana Smilkova, MD, PI	Protocol version 3.0 23/Mar/2012	NA (Only notification required)
LEC at University Multiprofile Hospital of Active Treatment "Prof. Stoyan Kirkovich" Stara Zagora 11, Armeiska St., 6003 Stara Zagora, Bulgaria	Dr. Valkova	36009 Diana Smilkova, MD, PI	Protocol version 4.0 22/Oct/2014	NA (Only notification required)
LEC at Diagnostic and Consultative Center	Valentina Dechkova	36011 Prof. Dimitar Raev, MD, PI	Protocol version 3.0 23/Mar/2012	NA (Only notification required)

BULGARIA				
IRB or IEC (name/address)	IRB or IEC Chairperson	Centre number (5 digit number) / Investigator(s)	Protocol and/or Amendment number(s)	Date of Final Approval (DD/MMM/YYYY)
"Ascendent", Sofia<br>47, Bacho Kiro St., 1202 Sofia, Bulgaria				
LEC at Diagnostic and Consultative Center "Ascendent", Sofia<br>47, Bacho Kiro St., 1202 Sofia, Bulgaria	Valentina Dechkova	36011 Prof. Dimitar Raev, MD, PI	Protocol version 4.0 22/Oct/2014	NA (Only notification required)
LEC at Multiprofile Hospital for Active Treatment "Vita", Sofia<br>9, Dragovitsa St., 1505 Sofia, Bulgaria	Dr. Ana Savcheva	36012 Anastas Stoikov, MD PI	Protocol version 3.0 23/Mar/2012	NA (Only notification required)
LEC at Multiprofile Hospital for Active Treatment "Vita", Sofia<br>9, Dragovitsa St., 1505 Sofia, Bulgaria	Dr. Ana Savcheva	36012 Anastas Stoikov, MD PI	Protocol version 4.0 22/Oct/2014	NA (Only notification required)

BULGARIA				
IRB or IEC (name/address)	IRB or IEC Chairperson	Centre number (5 digit number) / Investigator(s)	Protocol and/or Amendment number(s)	Date of Final Approval (DD/MMM/YYYY)
LEC at Multiprofile Hospital for Active Treatment "Dr. Ivan Seliminski", Sliven<br>1, Hristo Botev St., 8800 Sliven, Bulgaria	Dr. Bogdan Sotirov	36013 Silviya Syulemezova, MD, PI	Protocol version 3.0 23/Mar/2012	NA (Only notification required)
LEC at Multiprofile Hospital for Active Treatment "Dr. Ivan Seliminski", Sliven<br>1, Hristo Botev St., 8800 Sliven, Bulgaria	Dr. Bogdan Sotirov	36013 Silviya Syulemezova, MD, PI	Final Protocol<br>Global Amendment 1<br>(CTP 4.0/22 Oct 2014)	NA (Only notification required)
LEC at Multiprofile Hospital for Active Treatment "National Cardiology Hospital", Sofia<br>65, Konyovitsa St., 1309 Sofia, Bulgaria	Dr. Vera Baicheva	36014 Assoc. Prof. Elina Trendafilova, MD, PI	Protocol version 3.0 23/Mar/2012	NA (Only notification required)
LEC at Multiprofile Hospital for Active	Dr. Vera Baicheva	36014 Assoc. Prof. Elina Trendafilova, MD, PI	Final Protocol<br>Global Amendment 1	NA (Only notification required)

BULGARIA				
IRB or IEC (name/address)	IRB or IEC Chairperson	Centre number (5 digit number) / Investigator(s)	Protocol and/or Amendment number(s)	Date of Final Approval (DD/MMM/YYYY)
Treatment "National Cardiology Hospital", Sofia<br>65, Konyovitsa St., 1309 Sofia, Bulgaria			(CTP 4.0/22 Oct 2014)	
LEC at Multiprofile Hospital for Active Treatment "Sveta Marina", Varna<br>1, Hristo Smirnenski St., 9010 Varna, Bulgaria	Prof. Kiril Hristozov, MD	36015 Assoc. Prof. Yoto Yotov, MD, PI	Protocol version 3.0 23/Mar/2012	NA (Only notification required)
LEC at Multiprofile Hospital for Active Treatment "Sveta Marina", Varna<br>1, Hristo Smirnenski St., 9010 Varna, Bulgaria	Prof. Kiril Hristozov, MD	36015 Assoc. Prof. Yoto Yotov, MD, PI	Final Protocol<br>Global Amendment 1<br>(CTP 4.0/22 Oct 2014)	NA (Only notification required)

Canada				
IRB or IEC (name/address)	IRB or IEC Chairperson	Centre number (5 digit number) / Investigator(s)	Protocol and/or Amendment number(s)	Date of Final Approval (DD/MMM/YYYY)
IRB Services<br>372 Hollandview Trail, Suite 300<br>Aurora, ON, L4G 0A5	Dr. Fernand Laurendeau	06003/Bell	Protocol version 3.0 23/MAR/2012<br>Protocol version 4.0 7/JUN/2013	12 SEP 2011<br>18 JUL 2013
Health Research Ethics Board – Biomedical Panel<br>306 Campus Tower<br>Edmonton, Alberta<br>T6G 2R3	Dr. Stephen Bamforth	06004/Buthcher	Protocol version 3.0 23/MAR/2012<br>Protocol version 4.0 7/JUN/2013	3 OCT 2011<br>25 FEB 2014
IRB Services<br>372 Hollandview Trail, Suite 300<br>Aurora, ON, L4G 0A5	Dr. Fernand Laurendeau	06006/Chouinard	Protocol version 3.0 23/MAR/2012<br>Protocol version 4.0 7/JUN/2013	12 SEP 2011<br>18 JUL 2013
Comite d 'ethique de la recherche<br>Edifice Cooper<br>3981, boulevard St-Laurent, Mezz 2<br>Montreal, Quebec	Isabelle Duclos	06009/Coutu	Protocol version 3.0 23/MAR/2012<br>Protocol version 4.0 7/JUN/2013	16 NOV 2011<br>21 AUG 2013

Canada				
IRB or IEC (name/address)	IRB or IEC Chairperson	Centre number (5 digit number) / Investigator(s)	Protocol and/or Amendment number(s)	Date of Final Approval (DD/MMM/YYYY)
H2W 1Y5				
IRB Services<br>372 Hollandview Trail, Suite 300<br>Aurora, ON, L4G 0A5	Dr. Fernand Laurendeau	06014/Grondin	Protocol version 3.0 23/MAR/2012<br>Protocol version 4.0 7/JUN/2013	12 SEP 2011<br>18 JUL 2013
Hamilton Health Sciences / McMaster Faculty of Health Sciences Research Ethics Board<br>293 Wellington Street North, Suite 102<br>Hamilton, Ontario<br>L8L 8E7	Suzette Salama PhD	06016/Healey	Protocol version 3.0 23/MAR/2012<br>Protocol version 4.0 7/JUN/2013	22 AUG 2013

Canada				
IRB or IEC (name/address)	IRB or IEC Chairperson	Centre number (5 digit number) / Investigator(s)	Protocol and/or Amendment number(s)	Date of Final Approval (DD/MMM/YYYY)
IRB Services<br>372 Hollandview Trail, Suite 300<br>Aurora, ON, L4G 0A5	Dr. Fernand Laurendeau	06017/Heffernan	Protocol version 3.0 23/MAR/2012<br>Protocol version 4.0 7/JUN/2013	12 SEP 2011<br>18 JUL 2013
Faculty of Medicine<br>University of Calgary<br>Office of Medical Bioethics<br>3330 Hospital Dr NW<br>Heritage Medical Research Bldg,Suite 93<br>Calgary, Alberta<br>T2N 4N1	Stacey Page, PhD	06018/Hill	Protocol version 3.0 23/MAR/2012<br>Protocol version 4.0 7/JUN/2013	14 NOV 2011<br>23 SEP 2013
IRB Services<br>372 Hollandview Trail, Suite 300<br>Aurora, ON, L4G 0A5	Dr. Fernand Laurendeau	06020/Lam	Protocol version 3.0 23/MAR/2012<br>Protocol version 4.0 7/JUN/2013	12 SEP 2011<br>18 JUL 2013
Clinical Research Ethics Board (CRE B)<br>1952 Bay Street	Dr. D.S. Daly	06021/Leather	Protocol version 3.0 23/MAR/2012<br>Protocol version 4.0	28 OCT 2011

Canada				
IRB or IEC (name/address)	IRB or IEC Chairperson	Centre number (5 digit number) / Investigator(s)	Protocol and/or Amendment number(s)	Date of Final Approval (DD/MMM/YYYY)
3rd Floor - Kenning Wing, Memorial Pavilion Royal Jubilee Hospital<br>Victoria, BC VSR lJS			7/JUN/2013	03 SEP 2013
IUCPQ Research Ethics Committee<br>2725, Chemin Sainte-Foy<br>Quebec, PQ<br>G1V 4G5	DR. Franck Molin	06022/O'Hara	Protocol version 3.0 23/MAR/2012<br>Protocol version 4.0 7/JUN/2013	10 JAN 2012<br>19 SEP 2013
IRB Services<br>372 Hollandview Trail, Suite 300<br>Aurora, ON, L4G 0A5	Dr. Fernand Laurendeau	06023/Pandey	Protocol version 3.0 23/MAR/2012<br>Protocol version 4.0 7/JUN/2013	12 SEP 2011<br>18 JUL 2013
St. Micheal's Research Ethics Office<br>30 Bond Street<br>Toront, ON<br>M5B 1W8	Dr. Brenda McDowell	06026/Pinter	Protocol version 3.0 23/MAR/2012<br>Protocol version 4.0 7/JUN/2013	07 DEC 2011<br>27 JAN 2014

Canada				
IRB or IEC (name/address)	IRB or IEC Chairperson	Centre number (5 digit number) / Investigator(s)	Protocol and/or Amendment number(s)	Date of Final Approval (DD/MMM/YYYY)
University Health Network Research Ethics Board 10th Floor, Room 1056 700 University Ave<br>Toronto, Ontario, MSG 125	Anna Gagliardi, PhD<br>Alan Barolet , MD PhD	06031/Silver	Protocol version 3.0 23/MAR/2012<br>Protocol version 4.0 7/JUN/2013	03 OCT 2011<br>4 SEP 2013
IRB Services<br>372 Hollandview Trail, Suite 300<br>Aurora, ON, L4G 0A5	Dr. Fernand Laurendeau	06032/Tytus	Protocol version 3.0 23/MAR/2012<br>Protocol version 4.0 7/JUN/2013	12 SEP 2011<br>18 JUL 2013
University Health Network Research Ethics Board 10th Floor, Room 1056 700 University Ave<br>Toronto, Ontario, MSG 125	Alan Barolet , MD PhD	06036/Ha	Protocol version 3.0 23/MAR/2012<br>Protocol version 4.0 7/JUN/2013	13 MAY 2014

Canada				
IRB or IEC (name/address)	IRB or IEC Chairperson	Centre number (5 digit number) / Investigator(s)	Protocol and/or Amendment number(s)	Date of Final Approval (DD/MMM/YYYY)
IRB Services<br>372 Hollandview Trail, Suite 300<br>Aurora, ON, L4G 0A5	Dr. Fernand Laurendeau	06037/Henein	Protocol version 3.0 23/MAR/2012<br>Protocol version 4.0 7/JUN/2013	16 OCT 2013
Western University Health Science Research Ethics Board<br>Suppoty Services Bldg<br>Rm. 5150<br>London, Ontario, Canada<br>N6A 3K7	Dr. Joseph Gilbert	06038/Leong-Sit	Protocol version 3.0 23/MAR/2012<br>Protocol version 4.0 7/JUN/2013	30 JUN 2014
Human Health Research Ethics Commitee – Centre hospitalier universitaire de Sherbrooke<br>3001, 12e Avenue Nord, Z5-3014<br>Sherbrooke, QC<br>J1H 5N4	Annabelle Cumyn	06039/Roux	Protocol version 3.0 23/MAR/2012<br>Protocol version 4.0 7/JUN/2013	07 May 2014

Canada				
IRB or IEC (name/address)	IRB or IEC Chairperson	Centre number (5 digit number) / Investigator(s)	Protocol and/or Amendment number(s)	Date of Final Approval (DD/MMM/YYYY)
IRB Services<br>372 Hollandview Trail, Suite 300<br>Aurora, ON, L4G 0A5	Dr. Fernand Laurendeau	06040/Searles	Protocol version 3.0 23/MAR/2012<br>Protocol version 4.0 7/JUN/2013	11 FEB 2014
Ottawa Health Science Network Research Ethics Board<br>Civic Box 411 725 Parkdae Avenue<br>Ottawa, Ontario, Canada<br>K1Y 4E9	Dr. Raphael Saginur	06041/Steill	Protocol version 3.0 23/MAR/2012<br>Protocol version 4.0 7/JUN/2013	22 OCT 2014

Canada				
IRB or IEC (name/address)	IRB or IEC Chairperson	Centre number (5 digit number) / Investigator(s)	Protocol and/or Amendment number(s)	Date of Final Approval (DD/MMM/YYYY)
IRB Services<br>372 Hollandview Trail, Suite 300<br>Aurora, ON, L4G 0A5	Dr. Fernand Laurendeau	06042/Datta	Protocol version 3.0 23/MAR/2012<br>Protocol version 4.0 7/JUN/2013	31 MAR 2014
IRB Services<br>372 Hollandview Trail, Suite 300<br>Aurora, ON, L4G 0A5	Dr. Fernand Laurendeau	06043/Zizzo	Protocol version 3.0 23/MAR/2012<br>Protocol version 4.0 7/JUN/2013	31 MAR 2014
IRB Services<br>372 Hollandview Trail, Suite 300<br>Aurora, ON, L4G 0A5	Dr. Fernand Laurendeau	06044/Lowe	Protocol version 3.0 23/MAR/2012<br>Protocol version 4.0 7/JUN/2013	31 MAR 2014

CHILE				
IRB or IEC (name/address)	IRB or IEC Chairperson	Centre number (5 digit number) / Investigator(s)	Protocol and/or Amendment number(s)	Date of Final Approval (DD/MMM/YYYY)
Comité Ético Científico Servicio Salud Araucania Sur / Arturo Prat 969 - La Araucanía, Temuco, Chile	Patricio Valdes	56003 / Lanas Zanetti, Fernando	Protocol - version 4.0 - dated 07-Jun-2013<br>Local Amendment - version 1.0 - dated 15-Oct-2014	22/Oct/2013<br>20/Jan/2015
Comité Ética Científica HCFA / Av. las Condes 8631, Las Condes, Región Metropolitana, Chile	Tatiana Reyes	56006 / De Horta Campodonico, José	Protocol - version 4.0 - dated 07-Jun-2013<br>Local Amendment - version 1.0 - dated 15-Oct-2014	03/Nov/2015
Comité Ético Científico Servicio de Salud Metropolitana Sur / Av. Santa Rosa No 3453, San Miguel, Santiago, Chile	Veronica Rivera S	56007 / Olivares, Claudia	Protocol - version 4.0 - dated 07-Jun-2013<br>Local Amendment - version 1.0 - dated 15-Oct-2014	30/Oct/2015
Comité Ético Científico Servicio Salud Metropolitano Oriente / Av. Salvador 364, Providencia, Región Metropolitana, Chile	Sara Chernilo	56009 / Raffo Grado, Carlos	Protocol - version 4.0 - dated 07-Jun-2013<br>Local Amendment - version 1.0 - dated 15-Oct-2014	19/Apr/2016
Comité Ético Científico Servicio de Salud	Veronica Rivera S	56010 / Vega Miño, Mario	Protocol - version 4.0 - dated 07-Jun-2013	20/Jun/2016

CHILE				
IRB or IEC (name/address)	IRB or IEC Chairperson	Centre number (5 digit number) / Investigator(s)	Protocol and/or Amendment number(s)	Date of Final Approval (DD/MMM/YYYY)
Metropolitana Sur / Av. Santa Rosa No 3453, San Miguel, Santiago, Chile			Local Amendment - version 1.0 - dated 15-Oct-2014	

China				
IRB or IEC (name/address)	IRB or IEC Chairperson	Centre number (5 digit number) / Investigator(s)	Protocol and/or Amendment number(s)	Date of Final Approval (DD/MMM/YYYY)
Research Ethics Committee, Guangdong General Hospital, Guangdong Academy of Medical Sciences/ No.106 Zhongshan Er Road, Guangzhou	Yu, Xiyong	08001/ Shulin Wu	Protocol Amendment 4.0	04/DEC/2013
Ethics Committee of The Second Xiangya Hospital of Central South University/ No.139 Middle Renmin Road, Changsha	Yang, Lianyue	08007/ Shui Ping Zhao	Protocol Amendment 4.0	31/JUL/2013
Ethics Committee of Hangzhou First People's Hospital/Huansha Road No.261,Hangzhou	Huang Jinyu	08011/ Ningfu Wang	Protocol Amendment 4.0	30/AUG/2013
Ethics Committee of the Huadong hospital/ No.221 Yan'an Road (West), Jing'an District, Shanghai	Chen, jie	08012/ Zhaohui Qiu	Protocol Amendment 4.0	30/DEC/2013
Ethics Committee of Beijing Anzhen Hospital, Capital Medical University/	Sun，Yanqing	08017/ Changsheng Ma	Protocol Amendment 4.0	11/JUL/2013

China				
IRB or IEC (name/address)	IRB or IEC Chairperson	Centre number (5 digit number) / Investigator(s)	Protocol and/or Amendment number(s)	Date of Final Approval (DD/MMM/YYYY)
No.2,Anzhen Road,Chaoyang District,Beijing City,China				
Medical ethics committee of Beijing Shijingshan Hospital/ No.24 Shi Jing Shan Road, Beijing	Fu Jingliang	08018/ Mingsheng Wang	Protocol Amendment 4.0	26/JUL/2013
Ethics Committee of FuWai Hospital Chinese Academy of Medical Sciences /No.167,Beilishi Road,Xicheng District,Beijing	Chen, Xianshen	08019/ Yanmin Yang	Protocol Amendment 4.0	29/OCT/2013
Ethics review committee of Peking Union Medical College Hospital /No.1,Shuaifuyuan,Dongcheng District,Beijing	Bai, Hua	08023/ Quan Fang	Protocol Amendment 4.0	26/JUL/2013
Ethics Committee of Peking university first hospital /No 8，Xishenku Street，Xicheng District，Beijing City，China	Yimo Yang	08024/ Jing Zhou	Protocol Amendment 4.0	30 /AUG/2013
Ethics Committee of Zhongda Hospital Southeast University	Baoan Chen	08026/ Genshan Ma	Protocol Amendment 4.0	08/DEC/2014

China				
IRB or IEC (name/address)	IRB or IEC Chairperson	Centre number (5 digit number) / Investigator(s)	Protocol and/or Amendment number(s)	Date of Final Approval (DD/MMM/YYYY)
/No.87 Dingjia Bridge, Nanjing				
Ethics Committee of Shanghai East Hospital/ No.150 Jimo Road, Pudong New Area, Shanghai	Han Lei	08029/ Xuebo Liu	Protocol Amendment 4.0	16/DEC/2013
Ethics Committee of the Huashan hospital/ No.12 Urumqi Zhong Road	Zou hejian	08031/ Qiang Dong	Protocol Amendment 4.0	21/NOV/2013
Ethics Committee of Shanghai Tongji Hospital / No.389 Xincun Road , Putuo District,Shanghai	Luo, Ming	08032/ Ming Luo	Protocol Amendment 4.0	08/AUG/2013
Ethics Committee of Renji Hospital to Medical College of Shanghai Jiaotong University / No. 1630 Dong Fang Road, Pudong District, Shanghai	Kong Xianming	08036/ Xinhua Wang	Protocol Amendment 4.0	24/OCT/2014
Ethics Committee of The First Affiliated Hospital of Jilin University / No.71 Xinmin	Yuquan Tan	08039/ Yang Zheng	Protocol Amendment 4.0	05/SEP/2013

China				
IRB or IEC (name/address)	IRB or IEC Chairperson	Centre number (5 digit number) / Investigator(s)	Protocol and/or Amendment number(s)	Date of Final Approval (DD/MMM/YYYY)
Street, Changchun,Jilin ,China				
Ethics Committee of Chinese Traditional Medicine Hospital of Sichuna Province / No.41, Shierqiao Road, Chengdu	Zhang, zhongyuan	08049/ Yongjun Yin	Protocol Amendment 4.0	22/AUG/2013
Ethics Committee of Jinan Central Hospital/ No. 105 Jiefang Road, Jinan	Su, Guohai	08050/ Guohai Su	Protocol Amendment 4.0	06Sep2013
Ethics Committee of the Central Hospital of Tai'an/No.29 Longtan Road, Tai'an	Liu, Lun	08051/ Huayni Zhang	Protocol Amendment 4.0	25/JUN/2014
Ethics Committee of Chinese People's Liberation Army 401 Hospital	Xin, Suning	08054/ Nan Li	Protocol Amendment 4.0	30/DEC/2013
Ethics Committee of SuZhou Kowloon Hospital/No.118 Wansheng Street, Suzhou	Zhu, Xuming	08063/ Feng Liu	Protocol Amendment 4.0	01/AUG/2013
Ethics Committee of The Second Affiliated Hospital to Nanchang University/No.1	Ge, Xiaozhen	08065/ Kui Hong	Protocol Amendment 4.0	16/AUG/2013

China				
IRB or IEC (name/address)	IRB or IEC Chairperson	Centre number (5 digit number) / Investigator(s)	Protocol and/or Amendment number(s)	Date of Final Approval (DD/MMM/YYYY)
Minde Road, Nanchang				
Ethics Committee of Wuhan Asia Heart Hospital/No.753, Jinghan Avenue, Wuhan	Liu, Chengwei	08075/ Xi Su	Protocol Amendment 4.0	04/SEP/2013
Ethics Committee of Xiangya Hospital Central South University/ No.87 Xiangya Road, Changsha	Tang, Beisha	08076/ Tianlun Yang	Protocol Amendment 4.0	29/OCT/2013
Ethics Committee of Fujian Provicial Hospital / No.134 East Street, Fuzhou	Zhu, pengli	08078/ Wei Chen	Protocol Amendment 4.0	09/JUL/2014
Ethics Committee of The People's Hospital of Liaoning Province /No 33,Wenyi Road, Shenhe District, Shenyang City, Liaoning province China	Xilian,Jiang	08080/ Zhanquan Li	Protocol Amendment 4.0	01/AUG/2013
Ethics Committee of Shengjing Hospital of China Medical University/ No.36, Sanhao Street, Heping district, Shenyang city, Liaoning province, China	Danan Wang	08081/ Xiaodong Li	Protocol Amendment 4.0	30/AUG/2013

China				
IRB or IEC (name/address)	IRB or IEC Chairperson	Centre number (5 digit number) / Investigator(s)	Protocol and/or Amendment number(s)	Date of Final Approval (DD/MMM/YYYY)
Ethics Committee of Dalian Municipal Central Hospital/No.826 Xinan Road, Dalian	Jiang Xilian	08083/ Hailong Lin	Protocol Amendment 4.0	01/AUG/2013
Ethics Committee of The affiliated hospital of medicalcollege qingdao university/No.16 Jiangsu Road, Shinan District, Qingdao	Liang, Jun	08092/ Shanglang Cai	Protocol Amendment 4.0	29/SEP/2013
Ethics Committee of The First Affiliated Hospital of Xiamen University / No.55, Zhenhai Road, Xiamen, China	Zhang, zhiming	08100/ Weihua Li	Protocol Amendment 4.0	10/JAN/2014
Ethics Committee of The Forth People's Hospital of Shenzhen/ No 3025, Shennan road, Shenzhen, China	Ren, Fugui	08105/ Zhang Donghui	Protocol Amendment 4.0	11/JUN/2014
Ethics Committee of Jiading District Central Hospital/No 1, Chengbei Road, Jiading District, Shanghai - 201800	Zhang Yanhua	08106/ Xiangdong Xu	Protocol Amendment 4.0	24/MAR/2014

China				
IRB or IEC (name/address)	IRB or IEC Chairperson	Centre number (5 digit number) / Investigator(s)	Protocol and/or Amendment number(s)	Date of Final Approval (DD/MMM/YYYY)
Medical ethics committee of The Forth Hospital of Hebei Medical University/ No.169,Tianshanda Street,Dongkaifa District,Shijiazhuang	Wang, Faqi	08107/ Yalin Liu	Protocol Amendment 4.0	26/FEB/2014
Ethics Committee of Hangzhou Second People's Hospital/No 126, Wenzhou Road, Hangzhou - 310000	Wang Weiming	08109/ Xingwei Zhang	Protocol Amendment 4.0	28/APR/2014
Ethics Committee of Beijing Tsinghua Changgung Hospital/ No 168Llitang road, Changping district, Beijing Ctiy	Qinping Liao	08110/ Ping Zhang	Protocol Amendment 4.0	05/NOV/2015
Ethics Committee of Zhengzhou People's Hospital/ No.33, Huanghe Road,Zhengzhou, 450003	Liu, Caigao	08111/ Hengliang Liu	Protocol Amendment 4.0	24/SEP/2015
Ethics Committee of Zhengzhou No.7 People's Hospital/ N0.17, The Fifth	Sun, Didi	08112/ Yujie Zhao	Protocol Amendment 4.0	08/JAN/2016

China				
IRB or IEC (name/address)	IRB or IEC Chairperson	Centre number (5 digit number) / Investigator(s)	Protocol and/or Amendment number(s)	Date of Final Approval (DD/MMM/YYYY)
Jingnan Road, Zhengzhou , 450006				
Ethics Committee of Subei People's Hospital of Jiangsu Province/No.98, Nantong West Road, Yangzhou City, China	Wang, Jingcheng	08113/ Xiang Gu	Protocol Amendment 4.0	07/DEC/2017
Ethics Committee of Qianfoshan Hospital of Shandong Province/ No.16766 Jingshi Road	Jiang, Zhongmin	08114/ Yinglong Hou	Protocol Amendment 4.0	01/DEC/2015
Ethics Committee of Hubei Province Zhongshan Hospita/No. 26, Zhongshan Road, Wuhan, China	Si, Yuanren	08115/ Wenxia Zong	Protocol Amendment 4.0	09/DEC/2017
Ethics Committee of Shanxi Cardiovascular Hospital/ No.18,Yi fen Street,Taiyuan City	Wang, Gang	08116/ Xiaoming Li	Protocol Amendment 4.0	15/DEC/2015
Ethics Committee of Yankuang Group General Hospital/No.560 Kuangjian	Zhang Chuanjun	08117/ Jun Zhang	Protocol Amendment 4.0	13/JAN/2017

China				
IRB or IEC (name/address)	IRB or IEC Chairperson	Centre number (5 digit number) / Investigator(s)	Protocol and/or Amendment number(s)	Date of Final Approval (DD/MMM/YYYY)
east, Zoucheng, Shandong				
Ethics Committee of Xuzhou Central Hospital/ No .199.South Jiefang Road,Xuzhou,China	Mingwei Jiang	08119/ Bing Han	Protocol Amendment 4.0	04/NOV/2015
Ethics Committee of Shandong Jiaotong Hospital/ No.12, Middle Wuyingshan Road, Jinan City	Zhang, Huayi	08120/ Tian Wang	Protocol Amendment 4.0	02/FEB/2016
Ethics Committee of Zhengzhou Central Hospital/ No.195, The middle of Tongbo road, Zhengzhou, Henan	Lian, Hongkai	08121/ Zhichen Zhao	Protocol Amendment 4.0	15/JAN/2016
Ethics Committee of Jining people's hospital/No.17 the fifth Jingnan Road, Zhengzhou 450017,China	Sun Shuyin	08122/ Xiaofei Sun	Protocol Amendment 4.0	27/APR/2016
Ethics Committee of The First Afiliated Hospital, Sun Yet-sen University/ No.58 Zhongshan Er	CEC	08005/ Yugang Dong	Protocol Amendment 4.0	12/AUG/2013

China				
IRB or IEC (name/address)	IRB or IEC Chairperson	Centre number (5 digit number) / Investigator(s)	Protocol and/or Amendment number(s)	Date of Final Approval (DD/MMM/YYYY)
Road,Guangzhou				
Ethics Committee of Renji Hospital of Shanghai Second Medical University/ No.1630 Dongfang Road, Shanghai	Kong, Xianming	08014/ Ningyuan Fang	Protocol Amendment 4.0	24/OCT/2013
Ethics Committee of Songjiang Hospital/ NO.746 Zhongshan middle road, Shanghai	CEC	08027/ Jiang Heng	Protocol Amendment 4.0	24/FEB/2014
Ethics Committee of Minhang District Central Hospital/ No.170 Xin Song Road, Minhang District, Shanghai	CEC	08035/ Dadong Zhang	Protocol Amendment 4.0	19/JUL/2013
Ethics Committee of Renji Hospital of Shanghai Second Medical University/ No.1630 Dongfang Road, Shanghai	Kong, Xianming	08037/ Yansheng Li	Protocol Amendment 4.0	24/OCT/2013
Ethics Committee of Wu Jing Yi Xue Yuan Fu Shu Yi Yuan/ No.220 Chenglin Road, Tianjin	CEC	08043/ Tiemin Jiang	Protocol Amendment 4.0	26/SEP/2013

China				
IRB or IEC (name/address)	IRB or IEC Chairperson	Centre number (5 digit number) / Investigator(s)	Protocol and/or Amendment number(s)	Date of Final Approval (DD/MMM/YYYY)
Ethics Committee of Shangdong Provincial Hospital/ No.324 Jingwuweiqi Road, Jinan	Qin, Chengyong	08047/ Lianqun Cui	Protocol Amendment 4.0	29/NOV/2013
Ethics Committee of QingDao Municipal Hospital/ No.1 Jiaozhou Road, Qingdao	CEC	08052/ Quansan Zhang	Protocol Amendment 4.0	12/SEP/2013
Ethics Committee of Qingdao Central Hospital/ No.127 Si liu nan Road, Qingdao	CEC	08053/ Li Sun	Protocol Amendment 4.0	09/SEP/2013
Ethics Committee of The First Affiliated Hospital of Jinan University/ No. 613 West Huangpu Avenue, Guangzhou	Cha Zhengang	08060/ Zicheng Li	Protocol Amendment 4.0	25/OCT/2013
Ethics Committee of The Second Hospital of Hebei Medical University/ No.215 West Heping Road, Shijiazhuang	CEC	08066/ Xianghua Fu	Protocol Amendment 4.0	20/AUG/2013
Ethics Committee of Xiamen Heart Center/ No.201 South	CEC	08077/ Wenhui Liu	Protocol Amendment 4.0	16/SEP/2013

China				
IRB or IEC (name/address)	IRB or IEC Chairperson	Centre number (5 digit number) / Investigator(s)	Protocol and/or Amendment number(s)	Date of Final Approval (DD/MMM/YYYY)
Hubin Road, Xiamen				
Ethics Committee of The General Hospital of Shenyang Military Command/ No.83 Wenhua Road, Shenyang	Hou, Mingxiao	08091/ Zulu Wang	Protocol Amendment 4.0	28/AUG/2013
Ethics Committee of Chinese People's Liberation Army 401 Hospital/ No.22 Minjiang Road, Shi nan District, Qingdao	CEC	08095/ Zhitao Liu	Protocol Amendment 4.0	14/NOV/2013
Ethics Committee of Qingdao Fuwai Hospital/ No 201, Nanjing Road, Qingdao - 266034	CEC	08096/ Xianyan Jiang	Protocol Amendment 4.0	19/NOV/2013
Ethics Committee of QingDao Municipal Hospital/ No.1 Jiaozhou Road, Qingdao	CEC	08098/ Xinwen Zhao	Protocol Amendment 4.0	26/DEC/2013
Ethics Committee of QingDao Municipal Hospital/ No.1 Jiaozhou Road, Qingdao	CEC	08099/ Hong Ma	Protocol Amendment 4.0	26/DEC/2013
Ethics Committee of Foshan	CEC	08102/ Jianqiu Liang	Protocol Amendment 4.0	26/DEC/2013

China				
IRB or IEC (name/address)	IRB or IEC Chairperson	Centre number (5 digit number) / Investigator(s)	Protocol and/or Amendment number(s)	Date of Final Approval (DD/MMM/YYYY)
Hospital of Southern Medical University/ No.78, Weiguo Road, Foshan				
Ethics Committee of Wu Jing Zong Dui Hospital of Guangdong Province/ No. 268, Yanling Road	CEC	08103/ Zhaohui Wu	Protocol Amendment 4.0	21/JAN/2014

COLOMBIA				
IRB or IEC (name/address)	IRB or IEC Chairperson	Centre number (5 digit number) / Investigator(s)	Protocol and/or Amendment number(s)	Date of Final Approval (DD/MMM/YYYY)
Comité de Ética en Investigación Biomédica Fundación Valle del Lili / Av. Simón Bolívar<br>Cra 98 No. 18 – 49<br>PBX: (57) (2) 331 9090<br>Cali – Colombia	Luis Angel Betancur Franco	24002 / Esteban Gomez, Juan	Protocol - version 4.0 - dated 07-Jun-2013<br>Local Amendment - version 1.0 - dated 15-Oct-2014	16/Jan/2014<br>09/Feb/2015
Comité de Ética en investigación CAIMED / Cra 42 A # 17-50, Bogotá – Colombia	Fernando Suarez	24005 / Sanchez, Gregorio	Protocol - version 4.0 - dated 07-Jun-2013<br>Local Amendment - version 1.0 - dated 15-Oct-2014	03/Jul/2015
Comité de Ética en Investigación del Centro de Diagnóstico Cardiologico para la Investigación Biomédica	Carlos García del Rio	24007 / Manzur, Fernando	Protocol - version 4.0 - dated 07-Jun-2013<br>Local Amendment - version 1.0 - dated 15-Oct-2014	29/Nov/2013<br>10/Mar/2015

COLOMBIA				
IRB or IEC (name/address)	IRB or IEC Chairperson	Centre number (5 digit number) / Investigator(s)	Protocol and/or Amendment number(s)	Date of Final Approval (DD/MMM/YYYY)
Comité de Ética en Investigación Biomédica IPS centro médico Julian Coronel / Carrera 59 No. 1 E-21, Cali - Colombia	Diana Milena Martinez Buitrago	24014 / Coronel, Julian	Protocol - version 4.0 - dated 07-Jun-2013<br>Local Amendment - version 1.0 - dated 15-Oct-2014	10/Dec/2013<br>07/Feb/2015
Comité de Ética de la investigación del CEMDE / Cl. 33a #70a 175, Medellín, Antioquia, Colombia	Marta Elena Arias de Lotero	24015 / Nicolas, Jaramillo	Protocol - version 4.0 - dated 07-Jun-2013<br>Local Amendment - version 1.0 - dated 15-Oct-2014	15/May/2015
Comité de Ética en la Investigación CAIMED Carrera 42 A N° 17 – 50, Bogota, Colombia	Miguel Antonio Tolosa	24015 / Nicolas, Jaramillo	Protocol - version 4.0 - dated 07-Jun-2013	27/MAR/2018
Comité Institucional de Ética de investigación en Humanos Universidad CES / Calle 10A #22-04, Medellín, Antioquia, Colombia	Jorge Julian Osorio	24016 / Duque, Mauricio	Protocol - version 4.0 - dated 07-Jun-2013<br>Local Amendment - version 1.0 - dated 15-Oct-2014	25/Aug/2015

COLOMBIA				
IRB or IEC (name/address)	IRB or IEC Chairperson	Centre number (5 digit number) / Investigator(s)	Protocol and/or Amendment number(s)	Date of Final Approval (DD/MMM/YYYY)
Comité de Ética en Investigaciones de la Fundación Cardiovascular / Calle 155A # 23-58 Urb. El Bosque, Sector E1<br>Floridablanca - Santander	Evaristo José Vega Fernandez	24017 / Luengas, Carlos Alberto	Protocol - version 4.0 - dated 07-Jun-2013<br>Local Amendment - version 1.0 - dated 15-Oct-2014	06/Jul/2016
Comité de Ética en Investigación con Seres Humanos de la fundación hospital infantil Universitario de San Jose / Carrera 52 No. 67A -71 Bogotá D.C., Colombia	Olga Lucia Pedraza	24019 / Cardenas Rizo, Tatiana	Protocol - version 4.0 - dated 07-Jun-2013<br>Local Amendment - version 1.0 - dated 15-Oct-2014	03/Jun/2016<br>27/Jul/2016

CROATIA				
IRB or IEC (name/address)	IRB or IEC Chairperson	Centre number (5 digit number) / Investigator(s)	Protocol and/or Amendment number(s)	Date of Final Approval (DD/MMM/YYYY)
Agency for Medicinal Products and Medicla Devices – Central Ethics Committee – Ksaverska cesta 4, 10000 Zagreb, Croatia	Prof. Dinko Vitezić, MD, PhD	CEC / All sites	Initial approval<br><br>Protocol v.3.0 with Local Amendment 1 and Local Amendment 2	05JUN2013
Agency for Medicinal Products and Medicla Devices – Central Ethics Committee – Ksaverska cesta 4, 10000 Zagreb, Croatia	Prof. Dinko Vitezić, MD, PhD	CEC / All sites	Global Protocol Amendment 1, Protocol v.4.0<br>Local Amendment v.3.0	08APR2015
Drug Committee<br>Thalassotherpaia Opatija<br>Special Hospital for medical rehabilitation of heart and lung diseases and rheumatisim	Bojan Miletić, MD	04001/ PI (Prof. Viktor Peršić, MD	Initial approval<br><br>Protocol v.3.0 with Local Amendment 1 and Local Amendment 2	15MAR2013
Drug Committee<br>Thalassotherpaia Opatija<br>Special Hospital for	Notification by MD, Peršić	04001/ PI (Prof. Viktor Peršić, MD	Global Protocol Amendment 1, Protocol v.4.0	21SEP2015

CROATIA				
IRB or IEC (name/address)	IRB or IEC Chairperson	Centre number (5 digit number) / Investigator(s)	Protocol and/or Amendment number(s)	Date of Final Approval (DD/MMM/YYYY)
medical rehabilitation of heart and lung diseases and rheumatisim			Local Amendment v.3.0	
Drugs committee and transfusiology<br>Magdalena clinic for cardiovascular disease, Medicals faculty of Univerity J.J. Strossmayera in Osijek, Ljudevita Gaja 2, 49000 Krapinske Toplic, Croatia	Mr.sc. Davorka Prajdić Predrijavac, MD	04002 / PI (Prof. Robert Bernat, MD)	Initial approval<br><br>Protocol v.3.0 with Local Amendment 1 and Local Amendment 2	20Feb2013
Ethic Committee/ Magdalena,<br>Magdalena clinic for cardiovascular disease, Medicals faculty of Univerity J.J. Strossmayera in Osijek, Ljudevita Gaja 2, 49000 Krapinske Toplice, Croatia	Notification by MD Janko S. Nossan	04002 / PI (Janko Szavits Nossan, MD)	Notification-Protocol v.3.0 with Local Amendment 1 and Local Amendment 2	20 Mar 2014

CROATIA				
IRB or IEC (name/address)	IRB or IEC Chairperson	Centre number (5 digit number) / Investigator(s)	Protocol and/or Amendment number(s)	Date of Final Approval (DD/MMM/YYYY)
Drugs committee and transfusiology<br>Magdalena clinic for cardiovascular disease, Medicals faculty of Univerity J.J. Strossmayera in Osijek, Ljudevita Gaja 2, 49000 Krapinske Toplic, Croatia	Mr.sc. Davorka Prajdić Predrijavac, MD	04002 / PI (Prof. Robert Bernat, MD)	Global Protocol Amendment 1, Protocol v.4.0<br>Local Amendment v.3.0	Approval not applicable, EC notified about Protocol amendment on 30 Sep 2015
Drug Comitee of<br>CHC Zagreb,<br>Kišpatićeva 12, Zagreb	Igor Francetić, MD, PhD	04003/PI Prof. Davor Miličić, MD	Protocol v.3.0 with Local Amendment 1	NA (notification 30 Spe 2015)
Drug Comitee of<br>CHC Zagreb,<br>Kišpatićeva 12, Zagreb	Igor Francetić, MD, PhD	04003/PI Prof. Davor Miličić, MD	Local Amendment 2	12Sep2013
Drug Comitee of<br>CHC Zagreb,<br>Kišpatićeva 12, Zagreb	Notification by Davor Miličić, PI	04003/Pi Prof. Davor Miličić, MD	Global Protocol Amendment 1, Protocol v.4.0<br>Local Amendment v.3.0	30Sep2015
Drug Commitee<br>University hospital centre	Dina Vukičević Baudoin, MD	04004, PI Vanja Bašić Kes	Initial Approval<br>Protocol v.3.0 with Local	20May2013

CROATIA				
IRB or IEC (name/address)	IRB or IEC Chairperson	Centre number (5 digit number) / Investigator(s)	Protocol and/or Amendment number(s)	Date of Final Approval (DD/MMM/YYYY)
Sestre Milosrdnice, Vinogradska 29, 10000 Zagreb, Croatia			Amendment 1 and Local Amendment 2	
Drug Commitee<br>University hospital centre Sestre Milosrdnice, Vinogradska 29, 10000 Zagreb, Croatia	Notification by Bašić Kes, PI	04004. PI Vanja Bašić Kes, MD	Global Protocol Amendment 1, Protocol v.4.0<br>Local Amendment v.3.0	21Sep2015
Drug Commitee<br>Clinical Hospital Merkur, Zajčeva 19, 10 000 Zagreb	Anna Mrzljak, MD, PhD	04005, PI Darko Počanić	Initial approval<br>Protocol v.3.0 with Local Amendment 1 and Local Amendment 2	13Mar2013
Drug Commitee<br>Clinical Hospital Merkur, Zajčeva 19, 10 000 Zagreb		04005, PI Darko Počanić	Global Protocol Amendment 1, Protocol v.4.0<br>Local Amendment v.3.0	Not applicable (only initial notification about NIS is done)
University hospital centre Sestre Milosrdnice, Vinogradska 29, 10000 Zagreb, Croatia	Dina Vukičević Baudoin, MD	04006, PI Diana Delić Brkljačić, PI	Initial Approval<br>Protocol v.3.0 with Local Amendment 1 and Local Amendment 2	20May2013

CROATIA				
IRB or IEC (name/address)	IRB or IEC Chairperson	Centre number (5 digit number) / Investigator(s)	Protocol and/or Amendment number(s)	Date of Final Approval (DD/MMM/YYYY)
Ethic Commitee<br>University hospital centre Sestre Milosrdnice, Vinogradska 29, 10000 Zagreb, Croatia	Notification by Diana Delić Brkljačić, PI	04006, PI Diana Delić Brkljačić, PI	Global Protocol Amendment 1, Protocol v.4.0<br>Local Amendment v.3.0	21Sep2015
Drug Commitee<br>General hospital Zadar, Bože Peričića 5, 23 000 Zadar	Unknown	04009, PI Aleksandar Knežević	Initial Approval<br>Approval -Protocol v.3.0 with Local Amendment 1 and Local Amendment 2	28 Feb 2013
Drug Commitee<br>General hospital Zadar, Bože Peričića 5, 23 000 Zadar	Notification by Aleksandar Knežević, PI	04009, PI Aleksandar Knežević	Global Protocol Amendment 1, Protocol v.4.0<br>Local Amendment v.3.0	23Sep2015
Drug Committee<br>University hospital Centre Zagreb, Kišpatićeva 12, 10000 Zagreb, Croatia	Prof. Igor Francetić, PhD, MD	04010, PI Prof. Zdravka Poljaković, MD	Initial Approval<br>Approval -Protocol v.3.0 with Local Amendment 1 and Local Amendment 2	16Sep2013
Drug Committee<br>University hospital Centre Zagreb, Kišpatićeva 12,	Notification by Zdravka Poljaković, PI	04010, PI Prof. Zdravka Poljaković, MD	Global Protocol Amendment 1, Protocol v.4.0	30Sep2015

CROATIA				
IRB or IEC (name/address)	IRB or IEC Chairperson	Centre number (5 digit number) / Investigator(s)	Protocol and/or Amendment number(s)	Date of Final Approval (DD/MMM/YYYY)
10000 Zagreb, Croatia			Local Amendment v.3.0	
Drug Committee<br>Clinical Hospital Centre Split, Spiničićeva 1, 21000 Split	Prof. Jugoslav Bagatin, MD	04011, PI Nediljko Pivac,MD	Initial Approval<br>Approval -Protocol v.3.0 with Local Amendment 1	30Oct2012
Drug Committee<br>Clinical Hospital Centre Split, Spiničićeva 1, 21000 Split	Notification by Nediljko Pivac, PI	04011, PI Nediljko Pivac,MD	Notification- Local Amend 2	23Oct2013
Drug Committee<br>Clinical Hospital Centre Split, Spiničićeva 1, 21000 Split	Notification by Nediljko Pivac, PI	04011, PI Nediljko Pivac, MD	Global Protocol Amendment 1, Protocol v.4.0<br>Local Amendment v.3.0	22 Sep 2015
Agency for Medicinal Products and Medicla Devices – Central Ethics Committee – Ksaverska cesta 4, 10000 Zagreb, Croatia		CEC/All Sites	Global Protocol Amendment 1, Protocol v.4.0<br>Local Amendment v.3.0	22 Sep 2015
Central Ethics Committee		**CEC/All Sites**	Global Protocol	22 Sep 2015

CROATIA				
IRB or IEC (name/address)	IRB or IEC Chairperson	Centre number (5 digit number) / Investigator(s)	Protocol and/or Amendment number(s)	Date of Final Approval (DD/MMM/YYYY)
– Ksaverska cesta 4, 10000 Zagreb, Croatia			Amendment 1, Protocol v.4.0<br>Local Amendment v.3.0	

Czech Republic				
IRB or IEC (name/address)	IRB or IEC Chairperson	Centre number (5 digit number) / Investigator(s)	Protocol and/or Amendment number(s)	Date of Final Approval (DD/MMM/YYYY)
Multicentrics ethics committee and local ethics committee for all CZ sites, FN Brno – Bohunice, Jihlavská 20, 625 00 Brno	PharmDr. Šárka Kozáková, MBA	41001 / Prof. MUDr. Jindřich Špinar, Csc.;<br>41006 / MUDr. Petra Mašková, Ph.D.;<br>41009 /MUDr. Libor Nechvátal;<br>41010 / MUDr. Miroslav Rubáček;<br>41011 / MUDr. Milan Mikuš	Protocol version 3.0 23/MAR/2012<br>as part of the core submission<br>Protocol Local Amendment 1 Czech Republic dated 29-May-2013<br>Protocol Local Amendment 2 Czech Republic dated 01-Jun-2015<br>Global protocol amendment 1 version 4.0 dated 22-Oct-2014	14/NOV/2012<br>24/JUL/2013<br>16/SEP/2015<br>09/DEC2015

DENMARK				
IRB or IEC (name/address)	IRB or IEC Chairperson	Centre number (5 digit number) / Investigator(s)	Protocol and/or Amendment number(s)	Date of Approval (DD/MMM/YYYY)
Den Nationale Videnskabsetiske Komité Old address: Finsensvej 15 2000 Frederiksberg Danmark	NA	18001/ Brandes, Dr. Axel<br>18004 / Larsen, Dr. Bjerregard, Torben<br>18005 / Gadsbøll, Dr. Niels<br>18006 / Zeuthen, Dr. Elisabeth Louise<br>18007 / Nyvad, Dr. Ole<br>18008 / Kümler, Dr. Thomas	Protocol version 3.0 23 March 2012<br><br>Protocol version 4.0 22 Oct 2014	On 29 MAY 2012, a letter was sent to the EC to request if there was a need to submit the NIS study. It was confirmed no submissions were needed. (no study documents have been sent)<br><br>not submitted as not required per EC response

ECUADOR				
IRB or IEC (name/address)	IRB or IEC Chairperson	Centre number (5 digit number) / Investigator(s)	Protocol and/or Amendment number(s)	Date of Final Approval (DD/MMM/YYYY)
Comite de Bioetica (COBI) / Av. Universitaria, Quito 170129, Ecuador	Estevez Edmundo, MD, MSc	25001/ Duarte Vera, Yan Carlos	Local Amendment - version 1.0 - dated 15-Oct-2014	19/Dec/2014
Comite de Bioetica (COBI) / Av. Universitaria, Quito 170129, Ecuador	Estevez Edmundo, MD, MSc	25007 / Pezo Lopez, Luis Felipe	Local Amendment - version 1.0 - dated 15-Oct-2014	19/Dec/2014
Comite de Etica de Investigacion en Seres Humanos. Universidad San Francisco de Quito / Campus Cumbayá - Diego de Robles y Vía Interoceánica<br>Casa Corona of. CC 103, Quito, Ecuador	William F. Waters	25010 / Davalos Cordero, Vicente	Protocol - version 4.0 - dated 07-Jun-2013<br>Local Amendment - version 1.0 - dated 15-Oct-2014	26/Jun/2015

ESTONIA				
IRB or IEC (name/address)	IRB or IEC Chairperson	Centre number (5 digit number) / Investigator(s)	Protocol and/or Amendment number(s)	Date of Final Approval (DD/MMM/YYYY)
Tallinn Medical Research Ethics Committee (CEC), Hiiu 42, Tallinn 11619, Estonia	Mrs Kristi Rüütel	38001 / dr Kai Sules<br>38002 / dr Priit Kampus	Protocol version 3.0<br>23/Mar/2012	13/SEP/2012
Tallinn Medical Research Ethics Committee (CEC), Hiiu 42, Tallinn 11619, Estonia	Mrs Kristi Rüütel	38001 / dr Kai Sules<br>38002 / dr Priit Kampus	Protocol version 4.0: Global Amendment 1<br>22/OCT/2014	15/JAN/2015

FRANCE				
IRB or IEC (name/address)	IRB or IEC Chairperson	Centre number (5 digit number) / Investigator(s)	Protocol and/or Amendment number(s)	Date of Final Approval (DD/MMM/YYYY)
**Comité Consultatif sur le Traitement de l'Information en matière de Recherche dans le domaine de la Santé**<br>Ministère de l'Enseignement Supérieur et de la Recherche<br>Direction de la Recherche et de l'Innovation<br>1 rue Descartes<br>75231 PARIS Cedex 05	NA	All sites<br>13008/ Mihail Chreih<br>13014/ C. Brunschwig<br>13017/ M. Brunehaut<br>13018/ Y. Jamon<br>13019/ J. Dillinger<br>13021/ T. Schaupp<br>13022/ S. Destrac<br>13023/ M. Galinier<br>13026/ P. Dary<br>13032/ M. Amelot<br>13035/ El Jabali<br>13037/ F. Georger<br>13039/ Guignier<br>13040/ N. Breton<br>13052/ O. Gartenlaub<br>13053/ De Labriolle<br>13055/ Ferrier<br>13057/ P. Goube<br>13058/ H. Gorka	Protocol version 3.0 23/MAR/2012<br>Protocol version 4.0 7/JUN/2013	06/SEP/2012<br><br>NA

		13064/ Buhl<br>13065/ O. Dibon<br>13069/ J. Quedillac<br>13072/ J. Davy<br>13073/ Y. Cottin<br>13074/ D. Magnin<br>13075/ H. Vial<br>13081/ D. Irles<br>13084/ J. Georges<br>13085/ G. Cayla<br>13093/ P. Scala<br>13101/ D. Smadja<br>13102/ Jouve<br>13103/ M. Fayard<br>13105/ P.Audouin<br>13110/ N. Ley<br>13111/ S. Di Legge<br>13116/ Angoulvant<br>13121/ Cracan<br>13123/ T. Mirault<br>13125/ Benhalima<br>13130/ N. Duvilla<br>13135/ J. Pineau		

		13140/ S. Timsit<br>13141/ L. Chartier<br>13142/ Salem<br>13143/ J. Berneau<br>13144/ X. Ducrocq<br>13146/ S. Cohen<br>13147/ Touze<br>13148/ H. Buatier<br>13152/ O. Dascotte<br>13154/ S. Armero<br>13155/ K. Lavandier<br>13158/ N. Lellouche<br>13159/ Neykova		

Germany				
IRB or IEC (name/address)	IRB or IEC Chairperson	Centre number (5 digit number) / Investigator(s)	Protocol and/or Amendment number(s)	Date of Final Approval (DD/MMM/YYYY)
Landesärztekammer Rheinland-Pfalz<br>Ethik-Kommission<br>Deutschhausplatz 3<br>55116 Mainz	Prof. Dr. med. Dipl.-Ing. St. Letzel	02001 Altmann<br>02002 Baar<br>02003 Beermann<br>02004 Gmehling<br>02005 Haerer<br>02006 Mibach<br>02007 Schön<br>02008 Bosch<br>02009 Frickel<br>02010 Goss<br>02011 Tamm<br>02012 Taggeselle<br>02013 Utech<br>02014 Schmitz<br>02015 Theleman<br>02016 Weinrich<br>02017 Rybak<br>02018 Perings<br>02020 Ince<br>02021 Rieker	- Amendment 01 to protocol final version 3.0 from 23 Mar 2012<br>Germany<br>Date: 30 August 2012<br><br>- Final Protocol<br>Global Amendment 1<br>Version 4.0, 22 Oct 2014<br>(Amendment 02 to protocol final version 4.0 from 22 Oct 2014<br>Germany<br>Date: 19 NOV 2014)<br><br>- Amendment 03 to protocol final version 4.0 from 22 Oct 2014<br>Germany<br>Date: 30 July 2015	17 SEP 2012<br><br>18 DEC 2014<br><br>28 OCT 2015

Germany				
IRB or IEC (name/address)	IRB or IEC Chairperson	Centre number (5 digit number) / Investigator(s)	Protocol and/or Amendment number(s)	Date of Final Approval (DD/MMM/YYYY)
		02022 Jaeger<br>02023 Genz<br>02024 Strasser<br>02025 Leschke<br>02026 Schwimmbeck<br>02027 Bollmann<br>02028 Heidenreich<br>02029 Weritz<br>02030 Gabelmann<br>02031 Wild<br>02032 Weimar<br>02033 Lodde<br>02034 Karolyi<br>02035 Henschel<br>02036 Behrens<br>02037 Jung<br>02038 Michalski<br>02039 Schmitz<br>02040 Muegge<br>02041 Tebbe		

Germany				
IRB or IEC (name/address)	IRB or IEC Chairperson	Centre number (5 digit number) / Investigator(s)	Protocol and/or Amendment number(s)	Date of Final Approval (DD/MMM/YYYY)
		02042 Kastrup<br>02043 Rauch-Kroehnert<br>02044 Kallmuenzer<br>02045 Gremmler<br>02046 Groenefeld<br>02047 Kraft<br>02048 Goldmann<br>02049 Dorsel<br>02050 Kuck<br>02051 Seidl<br>02052 Willems<br>02053 Regner<br>02054 Schuster<br>02055 May<br>02056 Darius<br>02057 Claus<br>02058 Poppert<br>02059 Grond<br>02060 Vester<br>02061 Schnupp		

Germany				
IRB or IEC (name/address)	IRB or IEC Chairperson	Centre number (5 digit number) / Investigator(s)	Protocol and/or Amendment number(s)	Date of Final Approval (DD/MMM/YYYY)
		02062 Schrickel<br>02063 Schellinger<br>02064 Schlueter<br>02065 Guenther<br>02066 Lichy<br>02067 Behrens<br>02068 Von Mering<br>02069 Dziewas<br>02070 Wiemer<br>02071 Thomas<br>02072 Kuniss<br>02073 Lenz<br>02074 Grueger<br>02075 Schmidt<br>02076 Prondzinski<br>02077 Herbst<br>02078 Beyer-Westendorf<br>02079 Treichel<br>02080 Schoeniger<br>02081 Geisler		

Germany				
IRB or IEC (name/address)	IRB or IEC Chairperson	Centre number (5 digit number) / Investigator(s)	Protocol and/or Amendment number(s)	Date of Final Approval (DD/MMM/YYYY)
		02082 Wilke<br>02083 Walter<br>02084 Zahn<br>02085 Schneider<br>02086 Killermann<br>02087 Gonska<br>02088 Witzenbichler<br>02089 Dr. Sause<br>02090 Lemke<br>02091 Bott<br>02092 Klein<br>02093 Laske<br>02094 Vom Dahl<br>02095 Zimmermann<br>02096 Krause<br>02097 Soda<br>02098 Molitor<br>02099 Goette<br>02100 Sek<br>02101 Al-Zoebi		

Germany				
IRB or IEC (name/address)	IRB or IEC Chairperson	Centre number (5 digit number) / Investigator(s)	Protocol and/or Amendment number(s)	Date of Final Approval (DD/MMM/YYYY)
		02102 Seebass<br>02103 Sotiriou<br>02104 Kueppers		
Ethik-Kommission bei der Landesärztekammer Baden-Württemberg<br>Jahnstr. 40<br>70597 Stuttgart	Dr. med. Georg Hook	02025 Leschke<br>02037 Jung	Protocol Version 3.0,<br>23 MAR 2012	02 APR 2013
Ethik-Kommission<br>der Medizinischen Fakultät der Ruhr-Universität Bochum<br>Bürkle-de-la-Camp-Platz 1<br>44789 Bochum	Christine Schnell	02040 Muegge	Protocol Version 3.0,<br>23 MAR 2012<br>Amendment 01 to protocol final version 3.0 from 23 Mar 2012<br>Germany<br>Date: 30 August 2012	16 MAY 2013
Sächsische Landesärztekammer<br>Ethik-Kommission<br>Schützenhöhe 16<br>01099 Dresden	Prof. Dr. med. habil. Haupt	02034 Karolyi	Protocol Version 3.0,<br>23 MAR 2012<br><br>Amendment 01 to protocol final version 3.0	24 MAY 2013

Germany				
IRB or IEC (name/address)	IRB or IEC Chairperson	Centre number (5 digit number) / Investigator(s)	Protocol and/or Amendment number(s)	Date of Final Approval (DD/MMM/YYYY)
			from 23 Mar 2012<br>Germany<br>Date: 30 August 2012	
Ärztekammer Nordrhein<br>Ethik-Kommission<br>Tersteegenstr. 9<br>40474 Düsseldorf	Caroline Schulz	02026 Schwimmbeck<br>02054 Schuster<br>02060 Vester	Amendment 01 to protocol final version 3.0 from 23 Mar 2012<br>Germany<br>Date: 30 August 2012	12 MAR 2013
Ethik-Kommission an der Medizinischen Fakultät der Universität Leipzig<br>Käthe-Kollwitz-Str. 82<br>04109 Leipzig	Prof. Dr. R. Preiß	02027 Bollmann<br>02038 Hobohm	Protocol Version 3.0,<br>23 MAR 2012<br><br>Amendment 01 to protocol final version 3.0 from 23 Mar 2012<br>Germany<br>Date: 30 August 2012	07 MAY 2013
Ärztekammer Bremen<br>Ethik-Kommission<br>Schwachhauser Heerstr. 30<br>28209 Bremen	Prof. Dr. med. H. Rasche	02042 Kastrup	Protocol Version 3.0,<br>23 MAR 2012<br><br>Amendment 01 to	22 MAR 2013

Germany				
IRB or IEC (name/address)	IRB or IEC Chairperson	Centre number (5 digit number) / Investigator(s)	Protocol and/or Amendment number(s)	Date of Final Approval (DD/MMM/YYYY)
			protocol final version 3.0 from 23 Mar 2012<br>Germany<br>Date: 30 August 2012	
Ärztekammer Hamburg<br>Ethik-Kommission<br>Humboldtstr. 67a<br>22083 Hamburg	Prof. Dr. med. Th. Weber	02048 Goldmann (Park)	Protocol Version 3.0,<br>23 MAR 2012<br><br>Amendment 01 to protocol final version 3.0 from 23 Mar 2012<br>Germany<br>Date: 30 August 2012	25 APR 2013
Ethik-Kommission der Ärztekammer Westfalen-Lippe und der Medizinischen Fakultät der Westfälischen Wilhelms-Universität<br>Gartenstr. 201-214<br>48147 Münster	Univ.-Prof. Dr. med. Hans-Werner Bothe	02018 Perings	Protocol Version 3.0,<br>23 MAR 2012<br><br>Amendment 01 to protocol final version 3.0 from 23 Mar 2012<br>Germany<br>Date: 30 August 2012	14 FEB 2013

Germany				
IRB or IEC (name/address)	IRB or IEC Chairperson	Centre number (5 digit number) / Investigator(s)	Protocol and/or Amendment number(s)	Date of Final Approval (DD/MMM/YYYY)
Medizinische Fakultät der Universität Duisburg-Essen<br>Ethik-Kommission<br>Robert-Koch-Str. 9-11<br>45147 Essen	Prof. Dr. W. Havers	02032 Weimar	Protocol Version 3.0,<br>23 MAR 2012<br><br>Amendment 01 to protocol final version 3.0 from 23 Mar 2012<br>Germany<br>Date: 30 August 2012	16 APR 2013

GREECE				
IRB or IEC (name/address)	IRB or IEC Chairperson	Centre number (5 digit number) / Investigator(s)	Protocol and/or Amendment number(s)	Date of Approval (DD/MMM/YYYY)
University General Hospital of Heraklion<br>Stavrakia & Voutes<br>71110 Herakleion, Crete	Prof. Dimitris Georgopoulos	47001 / Parthenakis, Prof. Fragkiskos (new PI due to retirement of Vardas, Prof. Panagiotis)	Protocol version 3.0<br>Protocol version 4.0	04/JUL/2013<br>16/JUN/2015
General Panarkadiko Hospital of Tripolis,<br>Erithros Stavros Str.<br>22100 Tripoli	Dr. Venetsanos Ponirakos	47002 / Katranis, Dr. Alexandros	Protocol version 3.0<br>Protocol version 4.0	22/NOV/2013<br>27/FEB/2015
University Hospital of Alexandroupolis<br>Dragana / 6th km National Road Alexandroupoli/Makri<br>68100 Alexandroupoli	Mr. Efstratios Maltezos	47003 / Stakos, Dr. Dimitrios	Protocol version 3.0<br>Protocol version 4.0	12/JUN/2013<br>18/MAR/2015
Univ. Gen. Hosp. of Ioannina	Prof. Vasiliki Malamou-Mitsi	47004 / Goudevenos, Prof. Ioannis	Protocol version 3.0	17/APR/2013

GREECE				
IRB or IEC (name/address)	IRB or IEC Chairperson	Centre number (5 digit number) / Investigator(s)	Protocol and/or Amendment number(s)	Date of Approval (DD/MMM/YYYY)
Stavrou Niarchou Avenue<br>45500 Ioannina			Protocol version 4.0	17/FEB/2015
University Hospital of Patras<br>Rio<br>26504 Patras	Prof. Dimitrios Goumenos	47005 / Hahalis, Prof. Georgios	Protocol version 3.0<br>Protocol version 4.0	29/MAY/2013<br>27/MAY/2015
Gen. Hosp. of Thessaloniki "Hippokation",<br>49 Constantinoupoleos str<br>54642 Thessaloniki	Mrs. Maria Sdouga	47006 / Vassilikos, Dr. Vassilios	Protocol version 3.0<br>Protocol version 4.0	20/JUN/2013<br>17/FEB/2015
General Hospital of Voula "Asklipieio"<br>Voula<br>1 Vas. Pavlou str<br>16673 Athens	Mr. Kiriakos Sarafis	47007 / Manolis, Dr. Athanasios	Protocol version 3.0<br>Protocol version 4.0	12/JUN/2013<br>11/FEB/2015

GREECE				
IRB or IEC (name/address)	IRB or IEC Chairperson	Centre number (5 digit number) / Investigator(s)	Protocol and/or Amendment number(s)	Date of Approval (DD/MMM/YYYY)
General Hospital of Komotini "Sismanogleio"<br>69100 Komotini	Mr. Heraclis Chatzinestoros	47008 / Gkotsis, Dr. Alexandros	Protocol version 3.0	25/APR/2013
General Hospital of Athens "Korgialeneio-Benakio"<br>1, Erythrou Stavrou Str.<br>11526 Athens	Mr. I. Poulias	47009 / Katsivas, Dr. Apostolos	Protocol version 3.0<br>Protocol version 4.0	13/MAY/2013<br>02/FEB/2015
General Hospital of Edessa<br>Egnatia<br>58200 Edessa	Mr. Pantelis Makridis	47010/ Oikonomou, Dr. Kostas	Protocol version 3.0<br>Protocol version 4.0	08/APR/2013<br>12/FEB/2015
General Hospital of Chalkida<br>48 Gazepi str<br>34100 Chalkida	Dr. Ioannis Mantas	47011 / Mantas, Dr. Ioannis	Protocol version 3.0<br>Protocol version 4.0	27/MAR/2013<br>03/FEB/2015

GREECE				
IRB or IEC (name/address)	IRB or IEC Chairperson	Centre number (5 digit number) / Investigator(s)	Protocol and/or Amendment number(s)	Date of Approval (DD/MMM/YYYY)
General Hospital of Elefsina "Thriassio"<br>G. Gennimatas str.<br>19600 Elefsina	Mr. Sokratis Gavriil	47012 / Olympios, Dr. Christoforos	Protocol version 3.0<br>Protocol version 4.0	27/MAR/2013<br>16/FEB/2015
General Hospital of Chania<br>Mournies<br>73100 Chania	Dr. Chariton Papadakis	47013 / Kafkala, Dr. Krystallenia	Protocol version 3.0<br>Protocol version 4.0	22/APR/2013<br>25/FEB/2015
"Euroclinic" Hospital of Athens<br>9 Athanasiadou str<br>11521 Athens	Prof. Eftichios Voridis	47014 / Richter, Dr. Dimitrios	Protocol version 3.0<br>Protocol version 4.0	03/APR/2013<br>03/APR2015
Sotiria Hosp.,<br>152 Mesogion Avenue<br>11527 Athens	Dr. Ioannis Danos	47015 / Stergiou, Dr. Georgios	Protocol version 3.0<br>Protocol version 4.0	29/APR/2013<br>27/FEB/2015

GREECE				
IRB or IEC (name/address)	IRB or IEC Chairperson	Centre number (5 digit number) / Investigator(s)	Protocol and/or Amendment number(s)	Date of Approval (DD/MMM/YYYY)
University General Hospital of Larissa<br>Mezourlo<br>41110 Larissa	Prof. I. Fezoulidis	47016 / Makaritsis, Dr. Konstantinos	Protocol version 3.0<br><br>Protocol version 4.0	12/JUN/2014<br><br>12/FEB/2015
General Hospital of Kavala,<br>Ring Road, Nea Efkarpia<br>56429 Thessaloniki	Prof. Dimitrios Sotiriadis	47017 / Styliadis, Dr. Ioannis	Protocol version 3.0<br><br>Protocol version 4.0	10/JUN/2014<br><br>16/FEB/2015
General Hospital of Rhodes,<br>Agioi Apostoloi 1<br>85100 Rhodes	Mr. Stylianos Karatapanis	47019 / Moschos, Dr. Nikitas	Protocol version 4.0	02/APR/2015

Hong Kong				
IRB or IEC (name/address)	IRB or IEC Chairperson	Centre number (5 digit number) / Investigator(s)	Protocol and/or Amendment number(s)	Date of Final Approval (DD/MMM/YYYY)
Institutional Review Board of the University of Hong Kong / Hospital Authority Hong Kong West Cluster (HKU/HA HKW IRB)<br>Address: Room 901, Administration Block, Queen Mary Hospital, 102 Pokfulam Road, Hong Kong	Review Panel of meeting held on 08 Jan 2013 – Chairman: Prof. Kent-man CHU	35001/ H.F. Tse	Protocol version 3.0 23/MAR/2012	17/JAN/2013
Institutional Review Board of the University of Hong Kong / Hospital Authority Hong Kong West Cluster (HKU/HA HKW IRB)<br>Address: Room 901, Administration Block, Queen Mary Hospital, 102 Pokfulam Road, Hong Kong	IRB Reviewer: Prof. Kent-man CHU	35001/ H.F. Tse	Protocol version 4.0 7/JUN/2013	25/SEP/2013
Research Ethics Committee Kowloon West Cluster (KWC-REC)	Dr. Ashely CHENG	35002 / Danny Chow	Protocol version 4.0 7/JUN/2013	10/JAN/2014

Hong Kong				
IRB or IEC (name/address)	IRB or IEC Chairperson	Centre number (5 digit number) / Investigator(s)	Protocol and/or Amendment number(s)	Date of Final Approval (DD/MMM/YYYY)
Address: Room 533, 5/F, Block J, Princess of Margaret Hospital, Lai Chi Kok, Kowloon, Hong Kong				
Joint Chinese University of Hong Kong-New Territories East Cluster Clinical Research Ethics Committee (Joint CUHK-NTEC)<br>Address: 8/F, Lui Che Woo Clinical Sciences Building Quarters, Prince of Wales Hospital, Shatin, HK	Prof. Benny C.Y. ZEE	35003 / Bryan Ping Yen Yan	Protocol version 4.0 7/JUN/2013	06/DEC/2013
Joint Chinese University of Hong Kong-New Territories East Cluster Clinical Research Ethics Committee (Joint CUHK-NTEC)<br>Address: 8/F, Lui Che Woo Clinical Sciences Building Quarters, Prince of Wales Hospital, Shatin, HK	Prof. Benny C.Y. ZEE	35004 / Ka-Sing Wong	Protocol version 4.0 7/JUN/2013	01/APR/2014

Ireland (Republic of)				
IRB or IEC (name/address)	IRB or IEC Chairperson	Centre number (5 digit number) / Investigator(s)	Protocol and/or Amendment number(s)	Date of Final Approval (DD/MMM/YYYY)
Tallaght University Hospital/St. James's Hospital Joint Research Ethics Committee	Prof. Richard Deane	14001, 14008	Protocol version 3.0 23/MAR/2012Protocol version 4.0 7/JUN/2013	18Oct2012<br>11May2015
Mallow Primary Healthcare Centre Research Ethics Committee	Dr. Gargan	14002	Protocol version 3.0 23/MAR/2012Protocol version 4.0 7/JUN/2013	24Sep2013<br>N/A
Clinical Research Ethics Committee, Merlin Park Hospital, Galway	Dr. B Gerard Loftus	14005, 14009	Protocol version 3.0 23/MAR/2012Protocol version 4.0 7/JUN/2013	11Dec2012<br>21Apr2015

Italy				
IRB or IEC (name/address)	IRB or IEC Chairperson	Centre number (5 digit number) / Investigator(s)	Protocol and/or Amendment number(s)	Date of Final Approval (DD/MMM/YYYY)
Provinciale di Varese<br>c/o Azienda Ospedaliera "Ospedale di Circolo e Fondazione Macchi"<br>Viale Borri, 57<br>21100 VARESE	Prof Carlo Capella	Ageno - 15015	Protocol Version 3.0<br><br>Protocol Version 4.0	26/11/2013<br><br>24/03/2015
COMITATO ETICO INTERAZIENDALE<br>- l'Azienda Ospedaliera<br>SS. Antonio e Biagio e<br>Cesare Arrigo di Alessandria<br>Via Venezia, 16<br>15121 – Alessandria	Prof. Roberto Russo	Agosti - 15077	Protocol version 3.0<br><br>Protocol version 4.0	22/01/2014<br><br>06/03/2015
COMITATO ETICO<br>LAZIO 2<br>Via Primo Carnera, 1<br>00144 ROMA	Dr. Alfonso Mele	Ammirati - 15030	Protocol version 3.0<br><br>Protocol version 4.0	19/11/2013<br><br>19/02/2015
COMITATO ETICO<br>- la Provincia Monza	Prof Vittorio Locatelli	Annoni - 15017	Protocol version 3.0	5/12/2013

Italy				
IRB or IEC (name/address)	IRB or IEC Chairperson	Centre number (5 digit number) / Investigator(s)	Protocol and/or Amendment number(s)	Date of Final Approval (DD/MMM/YYYY)
Brianza<br>A.O. San Gerardo di Monza<br>Via Pergolesi, 33<br>20900 MONZA (MI)			Protocol version 4.0	02/04/2015
Comitato Etico<br>LAZIO 2<br>Via Primo Carnera 1<br>00142 ROMA	Dr. Alfonso Mele	Ansalone - 15040	Protocol version 3.0<br><br>Protocol version 4.0	19/11/2013<br><br>19/02/2015
COMITATO ETICO PROVINCIALE<br>Segreteria Scientifica<br>c/o Presso Azienda Ospedaliero - Universitaria Policlinico di Modena<br>Via - Pozzo, 71<br>41124 MODENA	Prof. Giuseppe Torelli	Bagni (ex Melandri) - 15066	Protocol version 3.0<br><br>Protocol version 4.0	14/01/2014<br><br>16/02/2015
COMITATO ETICO<br>per la Sperimentazione Clinica<br>- la Provincia di Venezia e	Prof. Enzo Raise	Baldi (ex D’Anna) - 15002	Protocol VERSION 3.0<br><br>Protocol VERSION 4.0	25/03/2014<br><br>14/04/2015

Italy				
IRB or IEC (name/address)	IRB or IEC Chairperson	Centre number (5 digit number) / Investigator(s)	Protocol and/or Amendment number(s)	Date of Final Approval (DD/MMM/YYYY)
IRCCS San Camillo (CESC)<br>Azienda U.L.S.S. 12 Veneziana<br>Via Don F. Tosatto, 147<br>30174 VENEZIA				
COMITATO ETICO<br>AREA VASTA NORD OVEST<br>presso l'Azienda Ospedaliera-<br>Universitraria Pisana<br>Via Roma, 67<br>56126 PISA	Dr Romano Danesi	Berti - 15041	Protocol VERSION 3.0<br>Protocol VERSION 4.0	07/11/2013<br>26/02/2015
COMITATO ETICO LAZIO 1<br>Azienda Ospedaliera San Camillo - Forlanini<br>Via Circonvallazione Gianicolense, 87<br>00152 Roma	Prof. Paola Grammatico	Biscione - 15082	Protocol VERSION 3.0<br><br>Protocol VERSION 4.0	08/04/2014<br><br>04/02/2015

Italy				
IRB or IEC (name/address)	IRB or IEC Chairperson	Centre number (5 digit number) / Investigator(s)	Protocol and/or Amendment number(s)	Date of Final Approval (DD/MMM/YYYY)
COMITATO ETICO UNICO REGIONALE<br>SEZIONE AREA NORD<br>c/o Azienda Ospedaliera di Cosenza<br>Via San Martino<br>87100 Cosenza	Prof. Nicola Perrotti	Bisignani - 15093	Protocol VERSION 3.0<br>Protocol VERSION 4.0	27/06/2014<br>21/04/2015
Comitato Etico<br>Azienda Ospedaliera Città - la Salute e - la Scienza di Torino- Azienda Ospedale Ordine Mauriziano di Torino –ASL TO1<br>Corso Bramante, 88/90<br>10126 TORINO	Prof . A. Pieri	Bo - 15019	Protocol VERSION 3.0<br>Protocol VERSION 4.0	18/12/2013<br>23/03/2015
COMITATO ETICO<br>AREA VASTA NORD OVEST<br>Azienda Ospedaliero Universitaria Pisana	Dr. Romano Danesi	Bongiorni - 15079	Protocol VERSION 3.0<br>Protocol VERSION 4.0	7/11/2013<br>26/02/2015

Italy				
IRB or IEC (name/address)	IRB or IEC Chairperson	Centre number (5 digit number) / Investigator(s)	Protocol and/or Amendment number(s)	Date of Final Approval (DD/MMM/YYYY)
Via Roma 67<br>56 126 PISA				
COMITATO ETICO<br>AREA VASTA SUD EST<br>c/o Dipartimento Politiche - Farmaco<br>Ospedale Misericordia<br>Via Senese, 161<br>58100 GROSSETO	Prof. Remo Martini	Bui – 15089	Protocol VERSION 3.0	17/03/2014
COMITATO ETICO LAZIO 2<br>ASL Roma C<br>Via Primo Carnera, 1<br>00144 Roma	Dr. Alfonso Mele	Calo' - 15053	Protocol VERSION 3.0<br><br>Protocol VERSION 4.0	19/11/2013<br><br>19/02/2015
COMITATO ETICO CATANIA 1<br>Azienda Ospedaliera Universitaria<br>Policlinico Vittorio Emanuele di Catania	Prof Filippo Drago	Calvi - 15029	Protocol VERSION 3.0<br><br>Protocol VERSION 4.0	28/01/2014<br><br>16/02/2015

Italy				
IRB or IEC (name/address)	IRB or IEC Chairperson	Centre number (5 digit number) / Investigator(s)	Protocol and/or Amendment number(s)	Date of Final Approval (DD/MMM/YYYY)
Via S. Sofia, 78<br>95123 Catania				
COMITATO ETICO<br>CAMPANIA SUD<br>Piazza S. Giovanni, 7<br>80031 Brusciano (NA)	Prof. Amelia Filippelli	Capuano - 15007	Protocol VERSION 3.0<br><br>Protocol VERSION 4.0	09/12/2014<br><br>05/02/2015
COMITATO ETICO<br>Azienda Sanitaria Unica Regionale<br>- le Marche di Ancona<br>Via Caduti - Lavoro, 40<br>60131 Ancona	Prof Giuseppe Visani	Capucci - 15049	Protocol VERSION 3.0<br><br>Protocol VERSION 4.0	03/04/2014<br><br>22/07/2015
COMITATO ETICO<br>per le Province di L'Aquila eTeramo<br>c/o la ASL di Avezzano/Sulmona/L'Aquila<br>Via Vetoio 67100 Coppito	Dr. Gianlorenzo Piccioli	Carolei - 15051	Protocol VERSION 3.0<br><br>Protocol VERSION 4.0	22/05/2014<br><br>19/03/2015
COMITATO ETICO	Prof. Francesco Di Carlo	Carvalho - 15023	Protocol VERSION 3.0	19/03/2014

Italy				
IRB or IEC (name/address)	IRB or IEC Chairperson	Centre number (5 digit number) / Investigator(s)	Protocol and/or Amendment number(s)	Date of Final Approval (DD/MMM/YYYY)
- l'Azienda Ospedaliera<br>Universitaria S. Luigi<br>Gonzaga di Orbassano<br>Regione Gonzole, 10<br>10043 Orbassano (TO)			Protocol VERSION 4.0	11/03/2015
COMITATO ETICO<br>ASL 1 Sassari<br>Via Monte Grappa, 82<br>07100 Sassari	Dr. Cesarino Onnis	Casu - 15031	Protocol VERSION 3.0<br>Protocol VERSION 4.0	11/02/2014<br>17/02/2015
COMITATO ETICO<br>c.a. Dr.ssa Linda Richieri<br>Aziende Sanitarie Umbria<br>Segreteria Scientifico-Amministrativa<br>Via - la Rivoluzione, 16<br>06070 Ellera di Corciano (PG)	Prof. Giancarlo Agnelli	Cavallini - 15014	Protocol VERSION 3.0<br>ProtocolVERSION 4.0 -	13/11/2013<br>22/01/2015
COMITATO ETICO<br>ASL Napoli 1 CENTRO<br>Via Comunale - Principe	Dr. Giovandomenico Lepore	Bruzzese (ex Celentano) - 15011	Protocol VERSION 3.0<br>Protocol VERSION 4.0	10/07/2014<br>19/02/2015

Italy				
IRB or IEC (name/address)	IRB or IEC Chairperson	Centre number (5 digit number) / Investigator(s)	Protocol and/or Amendment number(s)	Date of Final Approval (DD/MMM/YYYY)
13/A,<br>80145 Napoli				
COMITATO ETICO<br>REGIONE LIGURIA<br>c/o l'IRCCS Azienda Ospedaliera Universitaria "San Martino" – IST Istituto Nazionale per la Ricerca sul Cancro<br>L.go Rosanna Benzi 10<br>16132 GENOVA	Dr. Fulvio Brema	Chiarella - 15065	Protocol VERSION 3.0<br><br>Protocol VERSION 4.0	19/02/2014<br><br>12/02/2015
COMITATO ETICO LAZIO1<br>Azienda Ospedaliera<br>San Camillo - Forlanini<br>Via Circonvallazione Gianicolense, 87<br>00152 Roma	Prof. Paola Grammatico	Colivicchi 15035	Protocol VERSION 3.0<br><br>Protocol VERSION 4.0	06/11/2013<br><br>04/02/2015
COMITATO ETICO<br>Azienda Ospedaliero Universitaria	Dr. Alessio Nitti<br><br>Vice Chair dr.ssa Maria	Colonna - 15091	Protocol VERSION 3.0	12/03/2014

Italy				
IRB or IEC (name/address)	IRB or IEC Chairperson	Centre number (5 digit number) / Investigator(s)	Protocol and/or Amendment number(s)	Date of Final Approval (DD/MMM/YYYY)
Consorziale Policlinico di Bari<br>Piazza Giulio Cesare 11<br>70124 Bari	l' Aera		Protocol VERSION 4.0	25/02/2015
COMITATO ETICO<br>POLICLINICO GEMELLI<br>Università Cattolica - Sacro Cuore<br>Policlinico A. Gemelli<br>Largo A. Gemelli, 8<br>00168 ROMA	Prof. Salvatore Mancuso	Crea - 15086	Protocol VERSION 3.0<br><br>Protocol VERSION 4.0	20/03/2014<br><br>12/02/2015
COMITATO ETICO<br>Azienda USL - la Valle d'Aosta<br>c/o Direzione Area Ospedaliera<br>Ospedale Regionale Umberto Parini<br>Viale Ginevra, 3<br>11100 Aosta	Dr. Carlomaria Garbellotto	D'Alessandro (ex Bottacchi) - 15020	Protocol VERSION 3.0<br><br>Protocol Version 4.0	11/11/2013<br><br>27/2/2015

Italy				
IRB or IEC (name/address)	IRB or IEC Chairperson	Centre number (5 digit number) / Investigator(s)	Protocol and/or Amendment number(s)	Date of Final Approval (DD/MMM/YYYY)
COMITATO ETICO REGIONE LIGURIA<br>c/o l'IRCCS Azienda Ospedaliera Universitaria "San Martino" – IST Istituto Nazionale per la Ricerca sul Cancro<br>L.go Rosanna Benzi 10<br>16132 GENOVA	Prof. Fulvio Brema	De Franceschi - 15068	Protocol VERSION 3.0<br><br>Protocol VERSION 4.0	19/02/2014<br><br>12/02/2015
COMITATO ETICO REGIONALE UNICO (CERU)<br>Azienda Ospedaliero-Universitaria S. Maria - la Misericordia di Udine<br>Piazzale S. Maria - la Misericordia,15<br>33100 UDINE	Dr.ssa Maria Grazia Troncon	De Biasio - 15084	Protocol VERSION 3.0<br><br>Protocol VERSION 4.0	05/06/2014<br><br>24/02/2015
COMITATO ETICO<br>- la Ricerca Biomedica - le Province Di Chieti E Pescara	Dr. Camillo Romandini	Renda (ex De Caterina) - 15047	" Protocol VERSION 3.0<br><br>Protocol VERSION 4.0	17/07/2014<br><br>12/03/2015

Italy				
IRB or IEC (name/address)	IRB or IEC Chairperson	Centre number (5 digit number) / Investigator(s)	Protocol and/or Amendment number(s)	Date of Final Approval (DD/MMM/YYYY)
Via dei Vestini 29B<br>66100 CHIETI				
COMITATO ETICO<br>AREA VASTA SUD EST<br>c/o Dipartimento Politiche - Farmaco<br>Ospedale Misericordia<br>Via Senese, 161<br>58100 GROSSETO	Prof. Remo Martini	De Sensi (ex Severi) - 15092	Protocol VERSION 3.0<br>Protocol VERSION 4.0	20/05/2014<br>16/03/2015
COMITATO ETICO AREA<br>Azienda Ospedaliero-Universitaria Riuniti<br>di Foggia<br>Viale Luigi Pinto n. 1<br>71122 Foggia	Ing. Maria Rosaria Castrignano'	Brunetti (ex Di Biase) - 15050	Protocol VERSION 3.0<br>Protocol VERSION 4.0	07/04/2014<br>13/04/2015
Comitato Etico Indipendente - l'Azienda Ospedaliero Universitaria di Bologna – Policlinico S. Orsola Malpighi	Prof. Nicola Montanaro	Diemberger (ex Boriani) - 15054	Protocol VERSION 3.0<br>Protocol VERSION 4.0	19/11/2013<br>10/03/2015

Italy				
IRB or IEC (name/address)	IRB or IEC Chairperson	Centre number (5 digit number) / Investigator(s)	Protocol and/or Amendment number(s)	Date of Final Approval (DD/MMM/YYYY)
Via Albertoni, 15<br>40138 BOLOGNA				
COMITATO ETICO<br>Azienda Ospedaliero Universitaria<br>Consorziale Policlinico di Bari<br>Piazza Giulio Cesare 11<br>70124 Bari	Dr. Alessio Nitti	Favale - 15012	Protocol VERSION 3.0<br><br>Protocol VERSION 4.0	20/11/2013<br><br>25/02/2015
COMITATO ETICO SAPIENZA<br>Azienda Policlinico UmbertoI<br>Viale - Policlinico, 155<br>00161 Roma	Prof. Aldo Isidori	Fedele - 15033	Protocol VERSION 3.0<br><br>Protocol VERSION 4.0	16/01/2014<br><br>12/02/2015
COMITATO ETICO<br>per le Province di L'Aquila e Teramo<br>c/o la ASL di Avezzano/Sulmona/L'Aquila<br>Via Vetoio	Dr. Gianlorenzo Piccioli	Ferri - 15052	Protocol VERSION 3.0<br><br>Protocol VERSION 4.0	08/05/2014<br><br>19/03/2015

Italy				
IRB or IEC (name/address)	IRB or IEC Chairperson	Centre number (5 digit number) / Investigator(s)	Protocol and/or Amendment number(s)	Date of Final Approval (DD/MMM/YYYY)
67100 Coppito (AQ)				
COMITATO ETICO - l'Azienda Ospedaliera Universitaria "Maggiore - la Carità" di Novara<br>Corso Mazzini, 18<br>28100 Novara	Prof. Roberto Fantozzi	Frediani - 15018	Protocol VERSION 3.0<br><br>Protocol VERSION 4.0	14/02/2014<br><br>13/02/2015
COMITATO ETICO LAZIO2<br>Via Primo Carnera, 1<br>00142 Roma	Dr. Alfonso Mele	Gaspardone - 15090	Protocol VERSION 3.0<br><br>Protocol VERSION 4.0 -	25/03/2014<br><br>19/02/2015
COMITATO ETICO LAZIO2<br>ASL Roma C<br>Via Primo Carnera, 1<br>00144 Roma	Dr. Alfonso Mele	Battagliese (ex Greco) - 15088	Protocol VERSION 3.0<br><br>Protocol VERSION 4.0	19/11/2013<br><br>19/02/2015
Comitato Etico IRCCS Multimedica sezione - comitato Etico Centrale<br>IRCCS Lombardia<br>Via Milanese,300	Prof. Emilio Trabucchi	De Servi (ex Baronio – exGronda) - 15021	Protocol VERSION 3.0<br><br>Protocol VERSION 4.0	23/07/2013<br><br>18/12/2014

Italy				
IRB or IEC (name/address)	IRB or IEC Chairperson	Centre number (5 digit number) / Investigator(s)	Protocol and/or Amendment number(s)	Date of Final Approval (DD/MMM/YYYY)
20099 Sesto San Giovanni				
COMITATO ETICO<br>- la Provincia di Ferrara<br>c/o Ufficio Ricerca e Innovazione<br>Azienda Ospedaliero<br>Universitaria Sant'Anna di Ferrara<br>Via Aldo Moro, 8<br>44124 CONA - FERRARA	Dr.ssa Graziella Filippini	Guardigli - 15055	Protocol VERSION 3.0<br>Protocol VERSION 4.0	15/05/2014<br>26/02/2015
COMITATO ETICO CATANIA 2<br>Azienda Ospedaliera di Rilievo Nazionale e di Alta Specializzazione Garibaldi di Catania<br>P.zza S.M. Gesu, 7<br>95125 Catania	Prof. Renato Bernardini	Gulizia - 15024	Protocol VERSION 3.0<br>ProtocolVERSION 4.0	30/01/2014<br>11/02/2015
COMITATO ETICO<br>- l'Azienda Unità Sanitaria	Prof Fabrizio De Ponti	Imberti - 15060	Protocol VERSION 3.0	13/12/2013

Italy				
IRB or IEC (name/address)	IRB or IEC Chairperson	Centre number (5 digit number) / Investigator(s)	Protocol and/or Amendment number(s)	Date of Final Approval (DD/MMM/YYYY)
Locale<br>di Piacenza<br>Via Antonio Anguissola, 15<br>29121 PIACENZA			Protocol VERSION 4.0	27/03/2015
COMITATO ETICO REGIONALE SEZIONE AREA CENTRO<br>Azienda Ospedaliera Universitaria<br>Mater Domini<br>Via Vinicio Cortese<br>88100 Catanzaro	Prof. Pitrantonio Ricci	Indolfi - 15027	Protocol VERSION 3.0<br><br>Protocol VERSION 4.0	20/12/2013<br><br>13/03/2015
COMITATO ETICO<br>AREA VASTA CENTRO<br>Azienda Ospedaliero<br>Universitaria Careggi<br>Largo Brambilla 3<br>50134 FIRENZE	Prof. Pierangelo Geppetti	Landini - 15038	Protocol VERSION 3.0<br><br>Protocol VERSION 4.0	11/11/2013<br><br>23/02/2015
COMITATO ETICO<br>AREA VASTA CENTRO	Prof. Pierangelo Geppetti	Marchionni - 15044	Protocol VERSION 3.0	16/12/2013

Italy				
IRB or IEC (name/address)	IRB or IEC Chairperson	Centre number (5 digit number) / Investigator(s)	Protocol and/or Amendment number(s)	Date of Final Approval (DD/MMM/YYYY)
Azienda Ospedaliero<br>Universitaria Careggi<br>Largo Brambilla 3<br>50134 FIRENZE			ProtocolVERSION 4.0	09/02/2015
COMITATO ETICO<br>Azienda Ospedaliera<br>Antonio Cardarelli di Napoli<br>Via A. Cardarelli, 9<br>80131 Napoli	Dr. V. Galgano	Mauro - 15008	Protocol VERSION 3.0<br><br>Protocol VERSION 4.0	09/07/2014<br><br>20/04/2015
COMITATO ETICO<br>REGIONE LIGURIA<br>c/o l'IRCCS Azienda Ospedaliera Universitaria "San Martino" – IST Istituto Nazionale per la Ricerca sul Cancro<br>L.go Rosanna Benzi, 10<br>16132 GENOVA	Prof. Fulvio Brema	Mazzotta – 15067	Protocol VERSION 3.0<br><br>Protocol VERSION 4.0	19/02/2014<br><br>12/02/2015
COMITATO ETICO<br>per la Sperimentazione	– Dr. Roberto Leone	Molon (ex Barbieri) - 15001	Protocol VERSION 3.0	12/03/2014

Italy				
IRB or IEC (name/address)	IRB or IEC Chairperson	Centre number (5 digit number) / Investigator(s)	Protocol and/or Amendment number(s)	Date of Final Approval (DD/MMM/YYYY)
Clinica<br>- le Province di Verona e Rovigo<br>P.le A. Stefani, 1<br>37126 VERONA			Protocol VERSION 4.0	08/04/2015
COMITATO ETICO<br>AREA VASTA SUD EST<br>c/o Farmacia AOUS<br>Viale Bracci<br>53100 SIENA	Prof. Remo Martini	Mondillo - 15042	Protocol VERSION 3.0<br><br>Protocol VERSION 4.0	19/11/2013<br><br>16/02/2015
COMITATO ETICO<br>- l'Azienda Sanitaria Unica Regionale - le Marche di Ancona<br>Via Caduti - Lavoro, 40<br>60131 Ancona	Dr. Giuseppe visani	Moretti - 15081	Protocol VERSION 3.0<br><br>Protocol VERSION 4.0	02/07/2014<br><br>30/09/2015
Spett.le<br>COMITATO ETICO<br>per le Sperimentazioni<br>Cliniche - la Provincia	Dr. Paolo Pallini	Muriago (ex Vescovo) - 15005	Protocol VERSION 3.0<br><br>Protocol VERSION 4.0	24/03/2014<br><br>23/02/2015

Italy				
IRB or IEC (name/address)	IRB or IEC Chairperson	Centre number (5 digit number) / Investigator(s)	Protocol and/or Amendment number(s)	Date of Final Approval (DD/MMM/YYYY)
di Vicenza (CESC)<br>Viale Rodolfi, 37<br>36100 VICENZA				
COMITATO ETICO LAZIO 1<br>Azienda Ospedaliera<br>San Camillo - Forlanini<br>Via Circonvallazione Gianicolense, 87<br>00152 Roma	Prof. Paola Grammatico	Musumesci - 15078	Protocol VERSION 3.0<br><br>Protocol VERSION 4.0	13/11/2013<br><br>04/02/2015
COMITATO ETICO<br>A.O. Città - la Salute e - la Scienza di Torino- Azienda Ospedale Ordine Mauriziano di Torino –ASL TO1<br>Corso Bramante, 88/90<br>10126 TORINO	Prof. Alessandro Pileri	Norbiato - 15074	Protocol VERSION 3.0<br><br>Protocol VERSION 4.0	16/12/2013<br><br>22/06/2015
COMITATO ETICO PALERMO 1<br>c/o Azienda Ospedaliera Universitaria Policlinico	Prof. Salvatore Leone	Novo - 15025	Protocol VERSION 3.0<br><br>Protocol VERSION 4.0	12/02/2014<br><br>11/02/2015

Italy				
IRB or IEC (name/address)	IRB or IEC Chairperson	Centre number (5 digit number) / Investigator(s)	Protocol and/or Amendment number(s)	Date of Final Approval (DD/MMM/YYYY)
P.Giaccone<br>Via - Vespro, 127<br>90129 Palermo				
COMITATO ETICO<br>CESC per la sperimentazione clinica - le province di Treviso e Belluno<br>Azienda ULSS9 di Treviso<br>Borgo Cavalli 42<br>31100 TREVISO	Dr. Paolo Tottolo	De Leo (Ex Olivari) - 15006	Protocol VERSION 3.0<br>Protocol VERSION 4.0	23/01/2014<br>26/02/2015
COMITATO ETICO<br>- l'Azienda Sanitaria Regionale<br>- Molise di Campobasso<br>Via Ugo Petrella, 1<br>86100 Campobasso	Prof. Giovanni De Gaetano	Olivieri (ex Versaci) - 15075	Protocol VERSION 3.0<br>Protocol VERSION 4.0	19/11/2013<br>24/03/2015
COMITATO ETICO LAZIO 2<br>ASL Roma C	Dr. Alfonso Mele	Pajes - 15094	Protocol VERSION 3.0<br>Protocol VERSION 4.0	24/06/2014<br>19/02/2015

Italy				
IRB or IEC (name/address)	IRB or IEC Chairperson	Centre number (5 digit number) / Investigator(s)	Protocol and/or Amendment number(s)	Date of Final Approval (DD/MMM/YYYY)
Via Primo Carnera, 1<br>00142 Roma				
COMITATO ETICO AREA CREMONA-MANTOVA-LODI<br>- l'Azienda Ospedaliera Istituti Ospitalieri di Cremona<br>Viale Concordia, 1<br>26100 CREMONA	Prof. Giovanni Corao	Passamonti (ex Pirelli) - 15071	Protocol VERSION 3.0<br><br>Protocol VERSION 4.0	21/03/2014<br><br>27/02/2015

Italy				
IRB or IEC (name/address)	IRB or IEC Chairperson	Centre number (5 digit number) / Investigator(s)	Protocol and/or Amendment number(s)	Date of Final Approval (DD/MMM/YYYY)
COMITATO ETICO<br>per la sperimentazione clinica - la provincia di Padova<br>Azienda Ospedaliera di Padova<br>Via Giustiniani, 1<br>35128 PADOVA	Dr.ssa Anna Chiara Frigo	Pengo - 15057	Protocol VERSION 3.0<br>Protocol VERSION 4.0	10/04/2014<br>12/02/2015
COMITATO ETICO REGIONALE SEZIONE AREA CENTRO<br>Azienda Ospedaliera Universitaria<br>Mater Domini<br>Via Vinicio Cortese<br>88100 Catanzaro	Prof. Pierantonio Ricci	Perticone - 15028	Protocol VERSION 3.0<br>Protocol VERSION 4.0	20/12/2013<br>13/03/2015
COMITATO ETICO<br>AREA VASTA CENTRO<br>Azienda Ospedaliero<br>Universitaria Careggi	Prof Pierangelo Geppetti	Pini (ex Conti) – 15087	Protocol VERSION 3.0<br>Protocol VERSION 4.0	03/03/2014<br>09/02/2015

Italy				
IRB or IEC (name/address)	IRB or IEC Chairperson	Centre number (5 digit number) / Investigator(s)	Protocol and/or Amendment number(s)	Date of Final Approval (DD/MMM/YYYY)
Largo Brambilla 3<br>50134 FIRENZE				
COMITATO ETICO PROVINCIALE<br>Azienda Ospedaliera Arcispedale Santa Maria Nuova I.R.C.C.S.<br>Viale Risorgimento, 57<br>42123 REGGIO EMILIA	Dr.Roberto Satolli	Leone (ex Pizzini ex iori) - 15063	Protocol VERSION 3.0<br>Protocol VERSION 4.0	24/02/2014<br>25/03/2015
COMITATO ETICO<br>Azienda Ospedaliero-Universitaria<br>di Cagliari<br>Via Ospedale, 54<br>09134 Cagliari	Prof. Renesto d'Aloja	Porcu - 15037	Protocol VERSION 3.0<br>Protocol VERSION 4.0	05/02/2014<br>25/03/2015
COMITATO ETICO AREA<br>CREMONA-MANTOVA-LODI<br>- l'Azienda Ospedaliera<br>Istituti Ospitalieri di	Prof. Giovanni Corrao	Prelle (ex Bertolazzi) - 15056	Protocol VERSION 3.0<br>Protocol VERSION 4.0	21/03/2014<br>27/02/2014

Italy				
IRB or IEC (name/address)	IRB or IEC Chairperson	Centre number (5 digit number) / Investigator(s)	Protocol and/or Amendment number(s)	Date of Final Approval (DD/MMM/YYYY)
Cremona<br>Viale Concordia, 1<br>26100 CREMONA				
Comitato Etico Referente Area di Pavia<br>Fondazione I.R.C.C.S. Policlinico "San Matteo"<br>Viale Camillo Golgi, 19<br>27100 PAVIA	Dr. Mario Regazzi	Raisaro - 15039	Protocol VERSION 3.0<br><br>Protocol VERSION 4.0	16/12/2013<br><br>09/02/2015
COMITATO ETICO<br>- la ASL LE di Lecce<br>Via Miglietta n°5<br>73100 Lecce	Prof. Diana Conte	Ria (ex Mussardo) - 15048	Protocol VERSION 3.0<br><br>Protocol VERSION 4.0	17/12/2013<br><br>10/02/2015
COMITATO ETICO<br>per la Sperimentazione Clinica<br>- la Provincia di Venezia e IRCCS San Camillo (CESC)<br>Azienda U.L.S.S. 12 Veneziana	Prof. Enzo Raise	Rigo - 15003	Protocol VERSION 3.0<br><br>Protocol VERSION 4.0	25/02/2014<br><br>19/05/2015

Italy				
IRB or IEC (name/address)	IRB or IEC Chairperson	Centre number (5 digit number) / Investigator(s)	Protocol and/or Amendment number(s)	Date of Final Approval (DD/MMM/YYYY)
Via Don F. Tosatto, 147<br>30174 VENEZIA				
COMITATO ETICO INDIPENDENTE<br>Fondazione Policlinico Tor Vergata<br>Viale Oxford, 81<br>00133 Roma	Prof. Maria Grazia Marciani	Romeo - 15032	Protocol VERSION 3.0<br><br>Protocol VERSION 4.0	18/03/2014<br><br>27/03/2015
Comitato etico degli IRCCS Istituto Europeo di Oncologia e Centro Cardiologico Monzino<br>C/O Istituto Europeo di Oncologia<br>Via Ripamonti, 435<br>20141 MILANO	Dr. Giovanni Apolone	Agostoni (ex Salvioni) - 15022	Protocol VERSION 3.0<br><br>Protocol VERSION 4.0	20/11/2013<br><br>18/02/2015
COMITATO ETICO	Prof. Fulvio Brenna	Serrati - 15069	Protocol VERSION 3.0	19/02/2014

Italy				
IRB or IEC (name/address)	IRB or IEC Chairperson	Centre number (5 digit number) / Investigator(s)	Protocol and/or Amendment number(s)	Date of Final Approval (DD/MMM/YYYY)
REGIONE LIGURIA<br>c/o l'IRCCS Azienda Ospedaliera Universitaria "San Martino" – IST Istituto Nazionale per la Ricerca sul Cancro<br>L.go Rosanna Benzi 10<br>16132 GENOVA			Protocol VERSION 4.0	12/02/2015
COMITATO ETICO<br>CAMPANIA CENTRO<br>ASL Napoli 1<br>Via Comunale - Principe 13/a<br>80145 Napoli	Dr. Giovandomenico LEPORE	Sibilio - 15010	Protocol VERSION 3.0<br><br>Protocol VERSION 4.0	09/10/2014<br><br>19/02/2015
Comitato Etico<br>- l'azienda ospedaliera<br>Ospedali Riuniti Marche Nord<br>p.zzle Cinelli,4<br>61121 Pesaro	Dr. Giuseppe Visani	Tarsi - 15046	Protocol VERSION 3.0<br><br>Protocol version 4;	30/06/2014<br><br>09/09/2015

Italy				
IRB or IEC (name/address)	IRB or IEC Chairperson	Centre number (5 digit number) / Investigator(s)	Protocol and/or Amendment number(s)	Date of Final Approval (DD/MMM/YYYY)
COMITATO ETICO<br>REGIONE LIGURIA<br>c/o l'IRCCS Azienda Ospedaliera Universitaria "San Martino" – IST Istituto Nazionale per la Ricerca sul Cancro<br>L.go Rosanna Benzi 10<br>16132 GENOVA	Prof. Fulvio Brema	Tassinari - 15061	Protocol VERSION 3.0<br><br>Protocol VERSION 4.0<br>"	19/02/2014<br><br>12/02/2015
COMITATO ETICO<br>c.a. Dr.ssa Linda Richieri<br>Aziende Sanitarie Umbria<br>Segreteria Scientifico-Amministrativa<br>Via - la Rivoluzione, 16<br>06070 Ellera di Corciano (PG)	Prof. Giancarlo Agnelli	Molini (ex Verdecchia) - 15045	Protocol VERSION 3.0<br><br>Protocol VERSION 4.0	13/11/2013<br><br>22/01/2015
COMITATO ETICO AREA CREMONA-MANTOVA-LODI<br>presso Azienda Ospedaliera	Prof. Giovanni Corrao	Reggiani (ex Zanini) - 15073	Protocol VERSION 3.0<br><br>Protocol VERSION 4.0	21/03/2014<br><br>27/02/2015

Italy				
IRB or IEC (name/address)	IRB or IEC Chairperson	Centre number (5 digit number) / Investigator(s)	Protocol and/or Amendment number(s)	Date of Final Approval (DD/MMM/YYYY)
Istituti Ospitalieri di Cremona<br>Viale Concordia, 1<br>26100 CREMONA				
COMITATO ETICO PROVINCIALE<br>Segreteria Scientifica<br>c/o Presso Azienda Ospedaliero - Universitaria Policlinico di Modena<br>Via - Pozzo, 71<br>41124 MODENA	Prof. Giuseppe Torelli	Bigliardi (ex Zini) - 15062	Protocol VERSION 3.0<br>Protocol VERSION 4.0	04/03/2014<br>17/02/2015

Japan				
IRB or IEC (name/address)	IRB or IEC Chairperson	Centre number (5 digit number) / Investigator(s)	Protocol and/or Amendment number(s)	Date of Final Approval (DD/MMM/YYYY)
The IRB of Tokyo Chidori Hospital<br><br>2-39-10, Chidori,<br>Tokyo, Ota<br>146-0083, Japan	Dr. Uehara, Toyohiko	60001 / Matsuda, Katsuhiro	Protocol version 4.0 7/JUN/2013<br>Proctocal local amendment version 1 22/APR/2014	06/JUN/2014<br><br>06/JUN/2014
The IRB of Tomisaka Clinic<br><br>2-5-7, Koishikawa,<br>Tokyo, Bunkyo<br>113-0033, Japan	Dr. Kondo, Kazuo	60002 / Miyamoto, Naomasa	Protocol version 4.0 7/JUN/2013<br>Proctocal local amendment version 1 22/APR/2014	02/JUL/2014<br><br>02/JUL/2014
The IRB of New Tokyo Hospital<br><br>1271, Wanagaya,<br>Chiba, Matsudo<br>270-2232, Japan	Dr. Ishiguro, Hisaaki	60003 / Fujino, Yusuke	Protocol version 4.0 7/JUN/2013<br>Proctocal local amendment version 1 22/APR/2014	15/JUL/2014<br><br>15/JUL/2014
The IRB of Tokyo	Dr. Takase, Atsushi	60004 / Tanaka, Katsumi	Protocol version 4.0	10/JUL/2014

Japan				
IRB or IEC (name/address)	IRB or IEC Chairperson	Centre number (5 digit number) / Investigator(s)	Protocol and/or Amendment number(s)	Date of Final Approval (DD/MMM/YYYY)
Midtown Medical Center<br><br>9-7-1, Akasaka,<br>Tokyo,Minato<br>107-6206, Japan			7/JUN/2013<br>Proctocal local amendment version 1 22/APR/2014	10/JUL/2014
The IRB of Tokyo Chidori Hospital<br><br>2-39-10, Chidori,<br>Tokyo, Ota<br>146-0083, Japan	Dr. Uehara, Toyohiko	60005 / Shimizu, Yutaka	Protocol version 4.0 7/JUN/2013<br>Proctocal local amendment version 1 22/APR/2014	06/JUN/2014<br>06/JUN/2014
The IRB of Clinical Research Tokyo Hospital<br><br>3-87-4, Haramachi,<br>Tokyo, Shinjuku<br>162-0053, Japan	Dr. Tabei, Takashi	60006 / Hata, Yoshiki	Protocol version 4.0 7/JUN/2013<br>Proctocal local amendment version 1 22/APR/2014	27/JUN/2014<br>27/JUN/2014
The IRB of Medical Corporationkokankai Nippon Kokan Hospital	Mr. Mizusawa, Kiyoshi	60007 / Sakai, Tetsuo	Protocol version 4.0 7/JUN/2013<br>Proctocal local	01/JUL/2014<br>01/JUL/2014

Japan				
IRB or IEC (name/address)	IRB or IEC Chairperson	Centre number (5 digit number) / Investigator(s)	Protocol and/or Amendment number(s)	Date of Final Approval (DD/MMM/YYYY)
1-2-1 Kokandori, Kawasaki-ku, Kawasaki, Kanagawa<br>210-0852, Japan			amendment version 1<br>22/APR/2014	
The IRB of Sagamihara Kyodo Hospital<br><br>2-8-18 Midori-ku, Hashimoto, Sagamihara, Kanagawa<br>252-5188, Japan	Dr. Iseki, Harukazu	60008 / Iseki, Harukazu	Protocol version 4.0 7/JUN/2013<br>Proctocal local amendment version 1<br>22/APR/2014	25/JUN/2014<br><br>25/JUN/2014
The IRB of Review Board of Human Rights and Ethics for Clinical Studies<br><br>13-2, Ichibancho,<br>Tokyo, Chiyoda<br>102-0082,Japan	Dr.Basugi,Norihiko	60009 / Yamamoto, Kazuya	Protocol version 4.0 7/JUN/2013<br>Proctocal local amendment version 1<br>22/APR/2014	20/JUN/2014<br><br>20/JUN/2014
The IRB of Tokusyukai Group Institutional	Dr. Ogawa, Makoto	60010 / Yamanoue, Hiroki	Protocol version 4.0 7/JUN/2013	18/JUN/2014

Japan				
IRB or IEC (name/address)	IRB or IEC Chairperson	Centre number (5 digit number) / Investigator(s)	Protocol and/or Amendment number(s)	Date of Final Approval (DD/MMM/YYYY)
Review Board<br><br>1-8-7, Koji-machi, Tokyo, Chiyoda<br>102-0083, Japan			Proctocal local amendment version 1<br>22/APR/2014	18/JUN/2014
The IRB of Clinical Research Tokyo Hospital<br><br>3-87-4, Haramachi, Tokyo, Shinjuku<br>162-0053, Japan	Dr. Tabei, Takashi	60011 / Kyo, Eisho	Protocol version 4.0 7/JUN/2013<br>Proctocal local amendment version 1<br>22/APR/2014	16/JUN/2014<br>16/JUN/2014
The IRB of Tokusyukai Group Institutional Review Board<br><br>1-8-7, Koji-machi, Tokyo, Chiyoda<br>102-0083, Japan	Dr. Ogawa, Makoto	60012 / Sueyoshi, Atsushi	Protocol version 4.0 7/JUN/2013<br>Proctocal local amendment version 1<br>22/APR/2014	21/JUN/2014<br>21/JUN/2014
The IRB of Tokusyukai Group Institutional Review Board	Dr. Ogawa, Makoto	60013 / Kawajiri, Kenji	Protocol version 4.0 7/JUN/2013<br>Proctocal local	21/JUN/2014

Japan				
IRB or IEC (name/address)	IRB or IEC Chairperson	Centre number (5 digit number) / Investigator(s)	Protocol and/or Amendment number(s)	Date of Final Approval (DD/MMM/YYYY)
1-8-7, Koji-machi, Tokyo, Chiyoda<br>102-0083, Japan			amendment version 1<br>22/APR/2014	21/JUN/2014
The IRB of Osaka Kaisei Hospital<br>1-6-10, Miyahara,<br>Osaka, Yodogawa<br>532-0003, Japan	Dr. Tanimura, Hirohisa	60014 / Tsutsui, Takashi	Protocol version 4.0 7/JUN/2013<br>Proctocal local amendment version 1<br>22/APR/2014	25/JUN/2014<br>25/JUN/2014
The IRB of Tokusyukai Group Institutional Review Board<br>1-8-7, Koji-machi, Tokyo, Chiyoda<br>102-0083, Japan	Dr. Ogawa, Makoto	60015 / Nakagawa, Hidemitsu	Protocol version 4.0 7/JUN/2013<br>Proctocal local amendment version 1<br>22/APR/2014	21/JUN/2014<br>21/JUN/2014
The IRB of Kitano Hospital<br>2-4-20 Kita-ku,	Dr. Saito, Isao	60016 / Haruna, Tetsuya	Protocol version 4.0 7/JUN/2013<br>Proctocal local amendment version 1	01/JUL/2014<br>01/JUL/2014

Japan				
IRB or IEC (name/address)	IRB or IEC Chairperson	Centre number (5 digit number) / Investigator(s)	Protocol and/or Amendment number(s)	Date of Final Approval (DD/MMM/YYYY)
Ogimachi,<br>Osaka, Osaka<br>530-8480, Japan			22/APR/2014	
The IRB of Tomisaka Clinic<br><br>2-5-7, Koishikawa, Tokyo, Bunkyo<br>113-0033, Japan	Dr. Kondo, Kazuo	60017 / Hishida, Eiji	Protocol version 4.0 7/JUN/2013<br>Proctocal local amendment version 1<br>22/APR/2014	02/JUL/2014<br><br>02/JUL/2014
The IRB of Kyokyukai Nishi Hospital<br><br>3-2-18, Bingocho, Nada-ku, Hyogo, Kobe<br>657-0037, Japan	Dr. Yoshikawa, Satoshi	60018 / Higashino, Yorihiko	Protocol version 4.0 7/JUN/2013<br>Proctocal local amendment version 1<br>22/APR/2014	26/MAY/2014<br><br>26/MAY/2014
The IRB of Kobe City Medical Center General Hospital	Dr. Ishihara, Takashi	60019 / Furukawa, Yutaka	Protocol version 4.0 7/JUN/2013<br>Proctocal local amendment version 1	14/JUL/2014<br><br>14/JUL/2014

Japan				
IRB or IEC (name/address)	IRB or IEC Chairperson	Centre number (5 digit number) / Investigator(s)	Protocol and/or Amendment number(s)	Date of Final Approval (DD/MMM/YYYY)
2-1-1 Minatojimaminamimachi, Chuo-ku,<br>Hyogo, Kobe<br>650-0047, Japan			22/APR/2014	
The IRB of Sugiura Clinic<br><br>4-4-16-301, Honmachi,<br>Saitama, Kawaguchi<br>332-0012, Japan	Dr. Hashimoto, Takao	60021 / Bando, Shigenobu	Protocol version 4.0 7/JUN/2013<br>Proctocal local amendment version 1<br>22/APR/2014	20/JUN/2014<br>20/JUN/2014
The IRB of Saiseikai Matsuyama Hospital<br><br>880-2 Yamanishicho, Ehime, Matsuyama<br>791-8026, Japan	Dr. Watanabe, Koki	60022 / Watanabe, Kouki	Protocol version 4.0 7/JUN/2013<br>Proctocal local amendment version 1<br>22/APR/2014	16/JUN/2014<br>16/JUN/2014
The IRB of Social Medical Corporation Chikamorikai Chikamori Hospital	Dr. Yamazaki, Masahiro	60023 / Kawai, Kazuya	Protocol version 4.0 7/JUN/2013<br>Proctocal local	02/JUL/2014<br>02/JUL/2014

Japan				
IRB or IEC (name/address)	IRB or IEC Chairperson	Centre number (5 digit number) / Investigator(s)	Protocol and/or Amendment number(s)	Date of Final Approval (DD/MMM/YYYY)
1-1-16 Okawasuji, Kochi, Kochi<br>780-8522, Japan			amendment version 1<br>22/APR/2014	
The IRB of Sugiura Clinic<br><br>4-4-16-301, Honmachi,<br>Saitama, Kawaguchi<br>332-0012, Japan	Dr. Hashimoto, Takao	60024 / Nakayama, Toru	Protocol version 4.0 7/JUN/2013<br>Proctocal local amendment version 1<br>22/APR/2014	05/JUN/2014<br>05/JUN/2014
The IRB of Iizuka Hospital / 3-83 Yoshiomachi, Iizuka, Fukuoka, 820-8505, Japan	Dr. Yufu, Yuji	60025 / Inoue, Shujiro	Protocol version 4.0 7/JUN/2013<br>Proctocal local amendment version 1<br>22/APR/2014	02/JUN/2014<br>02/JUN/2014
The IRB of Tokusyukai Group Institutional Review Board	Dr. Ogawa, Makoto	60026 / Shimomura, Hideki	Protocol version 4.0 7/JUN/2013<br>Proctocal local amendment version 1	18/JUN/2014<br>18/JUN/2014

Japan				
IRB or IEC (name/address)	IRB or IEC Chairperson	Centre number (5 digit number) / Investigator(s)	Protocol and/or Amendment number(s)	Date of Final Approval (DD/MMM/YYYY)
1-8-7, Koji-machi,<br>Tokyo, Chiyoda<br>102-0083, Japan			22/APR/2014	
The IRB of Tokyo Midtown Medical Center<br><br>9-7-1, Akasaka,<br>Tokyo,Minato<br>107-6206, Japan	Dr. Takase, Atsushi	60027 / Hisadome, Tetsuo	Protocol version 4.0 7/JUN/2013<br>Proctocal local amendment version 1 22/APR/2014	10/JUL/2014<br><br>10/JUL/2014
The IRB of Tokyo Midtown Medical Center<br><br>9-7-1, Akasaka,<br>Tokyo,Minato<br>107-6206, Japan	Dr. Takase, Atsushi	60028 / Fujii, Shigeru	Protocol version 4.0 7/JUN/2013<br>Proctocal local amendment version 1 22/APR/2014	10/JUL/2014<br><br>10/JUL/2014
The IRB of Tokyo Midtown Medical Center<br><br>9-7-1, Akasaka,<br>Tokyo,Minato	Dr. Takase, Atsushi	60029 / Nakamura, Yuichiro	Protocol version 4.0 7/JUN/2013<br>Proctocal local amendment version 1 22/APR/2014	10/JUL/2014<br><br>10/JUL/2014

Japan				
IRB or IEC (name/address)	IRB or IEC Chairperson	Centre number (5 digit number) / Investigator(s)	Protocol and/or Amendment number(s)	Date of Final Approval (DD/MMM/YYYY)
107-6206, Japan				
The IRB of Asahi General Hospital<br><br>1326 I,<br>Chiba, Asahi<br>289-2511, Japan	Dr. Watanabe, Saburo	60030 / Kanda, Junji	Protocol version 4.0 7/JUN/2013<br>Proctocal local amendment version 1<br>22/APR/2014	03/JUL/2014<br><br>03/JUL/2014
The IRB of Japanese Red Cross Fukuoka Hospital<br><br>3-1-1 Ogusu, Minami-ku, Fukuoka, Fukuoka<br>815-8555, Japan	Dr. Ino, Tetsuji	60031 / Meno, Hiroshi	Protocol version 4.0 7/JUN/2013<br>Proctocal local amendment version 1<br>22/APR/2014	09/JUL/2014<br><br>09/JUL/2014
The IRB of KKR Takamatsu Hospital<br><br>4-18 Tenjinmae,<br>Kagawa, Takamatsu<br>760-0018, Japan	Dr. Ehara, Kazuo	60032 / Takagi, Yuichiro	Protocol version 4.0 7/JUN/2013<br>Proctocal local amendment version 1<br>22/APR/2014	20/JUN/2014<br><br>20/JUN/2014
The IRB of The	Dr. Adachi, Isao	60033 / Sakamoto, Tamotsu	Protocol version 4.0	18/JUL/2014

Japan				
IRB or IEC (name/address)	IRB or IEC Chairperson	Centre number (5 digit number) / Investigator(s)	Protocol and/or Amendment number(s)	Date of Final Approval (DD/MMM/YYYY)
University of Toyama Hospital<br><br>2630 Sugitani, Toyama, Toyama<br>930-0194, Japan			7/JUN/2013<br>Proctocal local amendment version 1 22/APR/2014	18/JUL/2014
The IRB of Suwa Red Cross Hospital<br><br>5-11-50 Kogandori,<br>Nagano, Suwa<br>392-8510, Japan	Dr. Ohashi, Masahiko	60034 / Tsutsui, Hiroshi	Protocol version 4.0 7/JUN/2013<br>Proctocal local amendment version 1 22/APR/2014	02/JUL/2014<br>02/JUL/2014
The IRB of The Cardiovascular Institute Hospital<br><br>3-2-19 Nishiazabu,<br>Tokyo, Minato<br>106-0031, Japan	Dr. Kirigaya, Hajime	60035 / Yamashita, Takeshi	Protocol version 4.0 7/JUN/2013<br>Proctocal local amendment version 1 22/APR/2014	26/JUN/2014<br>26/JUN/2014
The IRB of Tokyo	Dr. Uehara, Toyohiko	60036 / Horiuchi, Masataka	Protocol version 4.0	10/JUL/2014

Japan				
IRB or IEC (name/address)	IRB or IEC Chairperson	Centre number (5 digit number) / Investigator(s)	Protocol and/or Amendment number(s)	Date of Final Approval (DD/MMM/YYYY)
Chidori Hospital<br><br>2-39-10, Chidori,<br>Tokyo, Ota<br>146-0083, Japan			7/JUN/2013<br>Proctocal local amendment version 1 22/APR/2014	 10/JUL/2014
The IRB of Tachikawa General Hospital<br><br>3-2-11 Kandamachi,<br>Niigata, Nagaoka<br>940-8621, Japan	Dr. Ikeda, Yoshio	60037 / Fujita, Satoru	Protocol version 4.0 7/JUN/2013<br>Proctocal local amendment version 1 22/APR/2014	03/JUN/2014<br>03/JUN/2014
The IRB of The Institute for Adult Diseases,Asahi Life Foundation<br><br>2-2-6 Nihonbashi-Bakurocho,<br>Tokyo, Chuo<br>103-0002 , Japan	Dr. Seko, Yoshinori	60038 / Kamo, Takehiro	Protocol version 4.0 7/JUN/2013<br>Proctocal local amendment version 1 22/APR/2014	14/JUL/2014<br>14/JUL/2014
The IRB of Fukuiken	Dr. Ushiogi, Yasuyuki	60039 / Maeno, Koji	Protocol version 4.0	22/JUL/2014

Japan				
IRB or IEC (name/address)	IRB or IEC Chairperson	Centre number (5 digit number) / Investigator(s)	Protocol and/or Amendment number(s)	Date of Final Approval (DD/MMM/YYYY)
Saiseikai Hospital<br><br>7-1 Funabashi, Wadanaka-cho,<br>Fukui, Fukui<br>918-8503, Japan			7/JUN/2013<br>Proctocal local amendment version 1<br>22/APR/2014	22/JUL/2014
The IRB of Tokyo Chidori Hospital<br><br>2-39-10, Chidori,<br>Tokyo, Ota<br>146-0083, Japan	Dr. Uehara, Toyohiko	60040 / Mori, Kiyoo	Protocol version 4.0 7/JUN/2013<br>Proctocal local amendment version 1<br>22/APR/2014	10/JUL/2014<br>10/JUL/2014
The IRB of Tokyo Midtown Medical Center<br><br>9-7-1, Akasaka,<br>Tokyo,Minato<br>107-6206, Japan	Dr. Takase, Atsushi	60041 / Shimizu, Naruhito	Protocol version 4.0 7/JUN/2013<br>Proctocal local amendment version 1<br>22/APR/2014	10/JUL/2014<br>10/JUL/2014
The IRB of Japan Community Health care	Dr. Inoue, Takeaki	60042 / Tayama, Shinji	Protocol version 4.0 7/JUN/2013	18/JUL/2014

Japan				
IRB or IEC (name/address)	IRB or IEC Chairperson	Centre number (5 digit number) / Investigator(s)	Protocol and/or Amendment number(s)	Date of Final Approval (DD/MMM/YYYY)
Organization Kumamoto General Hospital<br><br>10-10 Toricho,<br>Kumamoto, Yatsushiro<br>866-8660, Japan			Proctocal local amendment version 1<br>22/APR/2014	18/JUL/2014
The IRB of Sugiura Clinic<br><br>4-4-16-301, Honmachi,<br>Saitama, Kawaguchi<br>332-0012, Japan	Dr. Hashimoto, Takao	60043 / Fukunaga, Hiroshi	Protocol version 4.0 7/JUN/2013<br>Proctocal local amendment version 1<br>22/APR/2014	19/JUN/2014<br><br>19/JUN/2014
The IRB of Tokyo Chidori Hospital<br><br>2-39-10, Chidori,<br>Tokyo, Ota<br>146-0083, Japan	Dr. Uehara, Toyohiko	60044 / Sasaoka, Taishi	Protocol version 4.0 7/JUN/2013<br>Proctocal local amendment version 1<br>22/APR/2014	10/JUL/2014<br><br>10/JUL/2014
The IRB of Osaka saiseikai nakatsu hospital	Dr. Nishimura, Haruo	60045 / Shite, Junya	Protocol version 4.0 7/JUN/2013	02/JUL/2014

Japan				
IRB or IEC (name/address)	IRB or IEC Chairperson	Centre number (5 digit number) / Investigator(s)	Protocol and/or Amendment number(s)	Date of Final Approval (DD/MMM/YYYY)
2-10-39 Kita-ku, Shibata, Osaka, Osaka 530-0012, Japan			Proctocal local amendment version 1 22/APR/2014	02/JUL/2014

LATVIA				
IRB or IEC (name/address)	IRB or IEC Chairperson	Centre number (5 digit number) / Investigator(s)	Protocol and/or Amendment number(s)	Date of Final Approval (DD/MMM/YYYY)
Ethics Committee for clinical research at Pauls Stradins Clinical University Hospital development society	Prof. Stradins	37001 / PI Iveta Mintale<br>37002 / PI Iveta Sime<br>37003 / PI Nadezda Rozkova	Protocol Version 3.0	09/NOV/2012
Ethics Committee for clinical research at Pauls Stradins Clinical University Hospital development societyPilosnu Str. 13, LV-1002 Riga<br>Latvia	Prof. Stradins	37001 / PI Iveta Mintale<br>37002 / Pi Iveta Sime<br>37003 / PI Nadezda Rozkova	Global Amendment version 1.0<br>Protocol version 4.0	30/JAN/2015

Lebanon				
IRB or IEC (name/address)	IRB or IEC Chairperson	Centre number (5 digit number) / Investigator(s)	Protocol and/or Amendment number(s)	Date of Final Approval (DD/MMM/YYYY)
American University of Beirut Institutional Review Board, Beirut – Lebanon	Dr Ibrahim Salti	07001/ M Khoury	Protocol Version 3 dated 19 July 2011	08/MAY/2012
Comité d'Ethique de l'Hotel Dieu de France, Damascus Road, Beirut – Lebanon	Dr Georges Halaby	07002/	Protocol Version 3 dated 19 July 2011<br>Protocol Version 4 dated 7 June 2013 (amendment 1)	23/MAR/2012<br>7/JUL/2014
Medical Ethics Committee, Saint Georges Hospital University Medical Center, Beirut – Lebanon	Prof Chawki Cortbawi	07004/P Charbel	Protocol Version 4 dated 7 June 2013 (amendment 1)	10/FEB/2014
Centre Hospitalier Universitaire Notre Dame des Secours Ethics Committee, Jbeil – Lebanon	Father Michel Eliane	07005/H Bayeh	Protocol Version 4 dated 7 June 2013 (amendment 1)	06/NOV/2014
Hammoud Hospital Institutional Review Board, Dr Ghassan Hammoud Street, Saida –	Dr Ahmad Zaatari	07006/S Chaaban	Protocol Version 4 dated 7 June 2013 (amendment 1)	26/JUN/2014

Lebanon				
IRB or IEC (name/address)	IRB or IEC Chairperson	Centre number (5 digit number) / Investigator(s)	Protocol and/or Amendment number(s)	Date of Final Approval (DD/MMM/YYYY)
Lebanon				
Makassed General Hospital Institutional Review Board, Beirut – Lebanon	Dr Mariam Rajab	07007/I Kreidieh	Protocol Version 3 dated 19 July 2011<br>Protocol Version 4 dated 7 June 2013 (amendment 1)	01/AUG/2013<br>09/MAY/2014
Bellevue Medical Center Institutional Review Board, Mansourieh – Lebanon	Dr Ghassan Maalouf	07008/G Kiwan	Protocol Version 4 dated 7 June 2013 (amendment 1)	30/OCT/2013
Rafik Hariri University Hospital Institutional Review Board, Bir Hassan, Jnah – Beirut – Lebanon	Dr Iyad Issa	07009/S Kabbani	Protocol Version 4 dated 7 June 2013 (amendment 1)	30/OCT/2013
Rafik Hariri University Hospital Institutional Review Board, Bir Hassan, Jnah – Beirut – Lebanon	Dr Iyad Issa	07010/R Osman	Protocol Version 4 dated 7 June 2013 (amendment 1)	30/OCT/2013
Commission d'Ethique de la Recherche (CER),	Dr Loubna Tarabey	07011/N Mousallem	Protocol Version 4 dated 7 June 2013 (amendment 1)	25/NOV/2013

Lebanon				
IRB or IEC (name/address)	IRB or IEC Chairperson	Centre number (5 digit number) / Investigator(s)	Protocol and/or Amendment number(s)	Date of Final Approval (DD/MMM/YYYY)
Hôpital Saint Charles, Baabda – Lebanon				
Al Rassoul Al Aazam Hospital Institutional Review Board, Airport Avenue, Beirut – Lebanon	Dr Mahmoud Younes	07012/H Mansour	Protocol Version 4 dated 7 June 2013 (amendment 1)	20/FEB/2014
Makassed General Hospital Institutional Review Board, Beirut – Lebanon	Dr Mariam Rajab	07014/C Abdallah	Protocol Version 4 dated 7 June 2013 (amendment 1)	08/MAY/2014

MEXICO				
IRB or IEC (name/address)	IRB or IEC Chairperson	Centre number (5 digit number) / Investigator(s)	Protocol and/or Amendment number(s)	Date of Final Approval (DD/MMM/YYYY)
Comite de Etica en Investigacion de Sociedad Medica del Hospital Angeles de Culiacan, S.C / Blvd. Alfonso G. Calderon No. 2193, Desarrollo Urbano Tres Rios, Culiacan, Sinaloa. Mexico	Jorge Alverto Zamudio Lerma	26002 / Odin de los Rios Ibarra, Manuel	Protocol - version 3.0 - dated 19-Jul-2011	27/APR/2012
Comite de Etica en Investigacion de Sociedad Medica del Hospital Angeles de Culiacan, S.C / Blvd. Alfonso G. Calderon No. 2193, Desarrollo Urbano Tres Rios, Culiacan, Sinaloa. Mexico	Jorge Alverto Zamudio Lerma	26002 / Odin de los Rios Ibarra, Manuel	Protocol - version 4.0 - dated 07-Jun-2013	29/NOV/2013
Comite de Etica del Hospital General de Durango / Norman Fuentes y 5 de Febrero sin numero, Zona Centro, Durango Mexico	Jesus Armando Flores Alvarez	26004 / Núñez Fragoso, Juan Carlos	Protocol - version 3.0 - dated 19-Jul-2011	28/JAN/2013

MEXICO				
IRB or IEC (name/address)	IRB or IEC Chairperson	Centre number (5 digit number) / Investigator(s)	Protocol and/or Amendment number(s)	Date of Final Approval (DD/MMM/YYYY)
Comite de Etica del Hospital General de Durango / Norman Fuentes y 5 de Febrero sin numero, Zona Centro, Durango Mexico	Jaime Ayala Barragan	26004 / Núñez Fragoso, Juan Carlos	Protocol - Local Amendment - version 1.0 - dated 15-Oct-2014	27/MAY/2015
Comite de Etica en Investigacion del Instituto Nacional de Cardiologia /Juan Badino No 1. Col, Seccion XVI Del Tlalpan. Ciudad de Mexico	Angel Buenaventura Romero Cardenas	26008 / Hermosillo, Gonzalez	Protocol - version 3.0 - dated 19-Jul-2011	22/MAY/2012
Comite de Etica en Investigacion del Instituto Nacional de Cardiologia /Juan Badino No 1. Col, Seccion XVI Del Tlalpan. Ciudad de Mexico	Angel Buenaventura Romero Cardenas	26008 / Hermosillo, Gonzalez	Protocol - version 4.0 - dated 07-Jun-2013	15/OCT/2013

MEXICO				
IRB or IEC (name/address)	IRB or IEC Chairperson	Centre number (5 digit number) / Investigator(s)	Protocol and/or Amendment number(s)	Date of Final Approval (DD/MMM/YYYY)
Comite de Etica en Investigacion del Instituto Nacional de Cardiologia /Juan Badino No 1. Col, Seccion XVI Del Tlalpan. Ciudad de Mexico	Angel Buenaventura Romero Cardenas	26008 / Hermosillo, Gonzalez	Protocol - Local Amendment - version 1.0 - dated 15-Oct-2014	26/JAN/2016
Comite de Etica en Investigacion del Hospital General de Acapulco / Av. Ruiz Cortines No. 128. Col. Alta Progreso, Acapulco Mexico	Maria Iveth Bazan Gutierrez	26011 / Matadamas Hernandez, Norberto	Protocol - version 3.0 - dated 19-Jul-2011	16/APR/2012
Comite de Etica en Investigacion del Hospital General de Acapulco / Av. Ruiz Cortines No. 128. Col. Alta Progreso, Acapulco Mexico	Maria Iveth Bazan Gutierrez	26011 / Matadamas Hernandez, Norberto	Protocol - version 4.0 - dated 07-Jun-2013	27/NOV/2013
CLINBA<br>Calle Valenciana # 7, Colonia Paxtitlán, C.P. 36090, Guanajuato, Gto.MEXICO	Alejandra Medina Navarrete	26011 / Matadamas Hernandez, Norberto	Protocol - version 4.0 - dated 07-Jun-2013	16/JUL/2018

MEXICO				
IRB or IEC (name/address)	IRB or IEC Chairperson	Centre number (5 digit number) / Investigator(s)	Protocol and/or Amendment number(s)	Date of Final Approval (DD/MMM/YYYY)
Comite de Etica del Hospital Lomas de San Luis Internacional / av. Palmira No. 600. Col. Villas del Pedregal. San Luis Potosi	Alejandro Quesada Sanchez	26013 / Briones, Ignacio Rodriguez	Protocol - version 3.0 - dated 19-Jul-2011	17/APR/2012
Comite de Etica del Lospital Lomas de San Luis Internacional / av. Palmira No. 600. Col. Villas del Pedregal. San Luis Potosi	Ismael Padilla Ayala	26013 / Briones, Ignacio Rodriguez	Protocol - Local Amendment - version 1.0 - dated 15-Oct-2014	28/MAR/2015
Comite de Etica del Hospital Lomas de San Luis Internacional / av. Palmira No. 600. Col. Villas del Pedregal. San Luis Potosi	Ismael Padilla Ayala	26013 / Briones, Ignacio Rodriguez	Protocol - version 4.0 - dated 07-Jun-2013	04/SEP/2014
Comite de Etica en Investigacion de la Clinica Bajio Clinba / Valenciana Numero 7, col. Paxtitlan, Guanajuato Mexico	Dolores Davalos	26015 / Linares, Julio Alberto Aguilar	Protocol - version 3.0 - dated 19-Jul-2011	10/JUN/2013
Comite de Etica en Investigacion de la Clinica Bajio Clinba / Valenciana Numero 7, col. Paxtitlan, Guanajuato Mexico	Dolores Davalos	26015 / Linares, Julio Alberto Aguilar	Protocol - version 4.0 - dated 07-Jun-2013	24/OCT/2013

MEXICO				
IRB or IEC (name/address)	IRB or IEC Chairperson	Centre number (5 digit number) / Investigator(s)	Protocol and/or Amendment number(s)	Date of Final Approval (DD/MMM/YYYY)
Comite de Etica en Investigacion de la Clinica Bajio Clinba / Valenciana Numero 7, col. Paxtitlan, Guanajuato Mexico	Dolores Davalos	26015 / Linares, Julio Alberto Aguilar	Protocol - Local Amendment - version 1.0 - dated 15-Oct-2014	31/MAR/2015
Comite de Etica en Investigacion de la Clinica Bajio Clinba / Valenciana Numero 7, col. Paxtitlan, Guanajuato Mexico	Dolores Davalos	26016 / Limon Rodriguez, Ramon Horacio	Protocol - version 3.0 - dated 19-Jul-2011	18/MAY/2012
Comite de Etica en Investigacion de la Clinica Bajio Clinba / Valenciana Numero 7, col. Paxtitlan, Guanajuato Mexico	Dolores Davalos	26016 / Limon Rodriguez, Ramon Horacio	Protocol - version 4.0 - dated 07-Jun-2013	24/OCT/2013
Comite de Etica en Investigacion de la Clinica Bajio Clinba / Valenciana Numero 7, col. Paxtitlan, Guanajuato Mexico	Dolores Davalos	26016 / Limon Rodriguez, Ramon Horacio	Protocol - Local Amendment - version 1.0 - dated 15-Oct-2014	10/JUN/2015
Comite de Etica en Investigacion Christus Muguerza del Parque s.a de c.v/Calle Dr. Pedro leal Rodriguez No. 1802. Col Centro. Chihuahua Mexico	Francisco Javier Mar Arevalo	26017 / Consteras Buenrostro, Gabriel	Protocol - version 3.0 - dated 19-Jul-2011	16/MAY/2012

MEXICO				
IRB or IEC (name/address)	IRB or IEC Chairperson	Centre number (5 digit number) / Investigator(s)	Protocol and/or Amendment number(s)	Date of Final Approval (DD/MMM/YYYY)
Comite de Etica e Invesetigacion. Christus Muguerza del Parque s.a de c.v/Calle Dr. Pedro leal Rodriguez No. 1802. Col Centro. Chihuahua Mexico	Francisco Javier Mar Arevalo	26017 / Consteras Buenrostro, Gabriel	Protocol - version 4.0 - dated 07-Jun-2013	01/APR/2014
Comite de Etica e Invesetigacion. Christus Muguerza del Parque s.a de c.v/Calle Dr. Pedro leal Rodriguez No. 1802. Col Centro. Chihuahua Mexico	Francisco Javier Mar Arevalo	26017 / Consteras Buenrostro, Gabriel	Protocol - Local Amendment - version 1.0 - dated 15-Oct-2014	02/JUN/2015
Comite de Etica Medica e Investigacion de Promotora Medica Aguascalientes S.A de C.V / Republica de ecuadro numero 200, fracc. Las americas. Aguascalientes, Mexico	Fatima Alicia Delgado Salcedo	26033 / Guillermo Llamas Esperon	Protocol - version 3.0 - dated 19-Jul-2011	02/SEP/2013
Comite de Etica Medica e Investigacion de Promotora Medica Aguascalientes S.A de C.V / Republica de ecuadro numero 200, fracc. Las americas. Aguascalientes, Mexico	Fatima Alicia Delgado Salcedo	26033 / Guillermo Llamas Esperon	Protocol - version 4.0 - dated 07-Jun-2013	28/OCT/2013

MEXICO				
IRB or IEC (name/address)	IRB or IEC Chairperson	Centre number (5 digit number) / Investigator(s)	Protocol and/or Amendment number(s)	Date of Final Approval (DD/MMM/YYYY)
Comite de Etica Medica e Investigacion de Promotora Medica Aguascalientes S.A de C.V / Republica de ecuadro numero 200, fracc. Las americas. Aguascalientes, Mexico	Fatima Alicia Delgado Salcedo	26033 / Guillermo Llamas Esperon	Protocol - version 4.0 - dated 07-Jun-2013<br>Local Amendment - version 1.0 - dated 15-Oct-2014	30/JAN/2015
Comite de Etica en Investigacion del Instituto Jaliscience de Investigacion Clinica S.A de C.V / Penitenciaria numero 20. Col. Centro. Guadalajara, Mexico	Maria Gabriela Avelar Bribiesca	26036 / de la Peña Topete, Gilberto de Jesùs	Protocol - version 4.0 - dated 07-Jun-2013<br>Local Amendment - version 1.0 - dated 15-Oct-2014	30/JUN/2015
Comite de Etica en Investigacion del Centro de Investigacion Medica Integral S.C / Calle Escorza numero 492 col. Americana, Guadalajara Mexico	Ricardo Arias Torres	26037 / Padilla Padilla, Francisco Gerardo	Protocol - version 4.0 - dated 07-Jun-2013<br>Local Amendment - version 1.0 - dated 15-Oct-2014	29/JUL/2015

MEXICO				
IRB or IEC (name/address)	IRB or IEC Chairperson	Centre number (5 digit number) / Investigator(s)	Protocol and/or Amendment number(s)	Date of Final Approval (DD/MMM/YYYY)
Comite de Etica en Investigacion del Centro de Investigacion Farmaceutica Especializada de Occidente S.C / Calle Pedro Moreno Numero 934 Col Centro Guadalajara, Mexico	Carlos Ernesto Gonzalez Enriquez	26038 / Hernandez del Rio, Jorge Eduardo	Protocol - version 4.0 - dated 07-Jun-2013<br>Local Amendment - version 1.0 - dated 15-Oct-2014	13/AUG/2015
Comite de Etica en Investigacion Biomedica para el Desarrollo de Farmacos S.A de C.V / Calzada club atlas sur, numero 16, El salto, Jalisco Mexico	Blanca Ivette Venegas Lopez	26039 / Padilla Ríos, Victoria del Carmen	Protocol - version 4.0 - dated 07-Jun-2013<br>Local Amendment - version 1.0 - dated 15-Oct-2014	22/JUN/2015
Comite de Etica en Investigacion Biomedica para el Desarrollo de Farmacos S.A de C.V / Calzada club atlas sur, numero 16, El salto, Jalisco Mexico	Blanca Ivette Venegas Lopez	26040 / Camarillo Sánchez, Mauricio	Protocol - version 4.0 - dated 07-Jun-2013<br>Local Amendment - version 1.0 - dated 15-Oct-2014	29/JUL/2015

MEXICO				
IRB or IEC (name/address)	IRB or IEC Chairperson	Centre number (5 digit number) / Investigator(s)	Protocol and/or Amendment number(s)	Date of Final Approval (DD/MMM/YYYY)
Comite de Etica en Investigacion del Instituto de Corazon de Queretaro S.A de C.V /Prolongacion Privada Ignacio Zaragoza 16-B, segundo piso. Col. Centro, Queretaro Mexico	Miguel angel Alexandro Rangel Alvarado	26041 / León González, Salvador	Protocol - version 4.0 - dated 07-Jun-2013<br>Local Amendment - version 1.0 - dated 15-Oct-2014	10/FEB/2016
Comite de Etica en Investigacion de Medico Centre for Clinical Research S.A de C.V /Calle Amores 709 Col. Del valle, Ciudad de Mexico	Celia Ovadia Savariego	26042 / Aldrete Velasco, Jorge A	Protocol - version 4.0 - dated 07-Jun-2013<br>Local Amendment - version 1.0 - dated 15-Oct-2014	06/OCT/2015
Comite de Etica en Investigacion del Hospital General de Cualiacan / Calle Juan Aldama S/n esquina Estado de Nayarit, Culiacan Mexico	Elmer Guillermo Lopez Meza	26043 / González López, Victor Manuel	Protocol - version 4.0 - dated 07-Jun-2013<br>Local Amendment - version 1.0 - dated 15-Oct-2014	07/JUL/2015

MEXICO				
IRB or IEC (name/address)	IRB or IEC Chairperson	Centre number (5 digit number) / Investigator(s)	Protocol and/or Amendment number(s)	Date of Final Approval (DD/MMM/YYYY)
Comite de Etica en Investigacion de la Unidad de Investigacion Clinica en Medicina S.C / Av de la clinica 2520 - 520 Monterrey Nuevo Leon Mexico	Salvador Bruno Valdovinos Chavez	26044 / Jerjes Sanchez Diaz, Carlos	Protocol - version 4.0 - dated 07-Jun-2013<br>Local Amendment - version 1.0 - dated 15-Oct-2014	18/JUL/2015
Comite de Etica en Investigacion del Centro de Investigacion Farmaceutica Especializada de occidente S.C / Calle Pedro Moreno Numero 934 Col Centro Guadalajara, Mexico	Carlos Ernesto Gonzalez Enriquez	26045 / Perez Rios, Alma Minerva	Protocol - version 4.0 - dated 07-Jun-2013<br>Local Amendment - version 1.0 - dated 15-Oct-2014	20/AUG/2015
Comite de Etica en Investigacion de la Clinica Bajio Clinba / Valenciana Numero 7, col. Paxtitlan, Guanajuato Mexico	Dolores Davalos	26046 / Olvera Ruiz, Rafael	Protocol - version 4.0 - dated 07-Jun-2013<br>Local Amendment - version 1.0 - dated 15-Oct-2014	22/JAN/2016

NETHERLANDS				
IRB or IEC (name/address)	IRB or IEC Chairperson	Centre number (5 digit number) / Investigator(s)	Protocol and/or Amendment number(s)	Date of Approval (DD/MMM/YYYY)
Medisch Ethische Toetsingscommisie<br>E2 170/172<br>Academisch Medisch Centrum<br>Meibergdreef 9<br>1105 AZ Amsterdam	Not applicable	01001/ Brouwers, Dr. P.J.A.M<br>01002/ Tuininga, Dr. Ype<br>01003/ Crijns, Dr. H.J.G.M.<br>01004/ Van Eck, Dr. M.<br>01005/ Aydin, Dr. S.<br>01006/ The, Dr. S.H.K.<br>01007/ Ronner, Dr. E.<br>01008/ Huizenga, Dr. A. (stopdate 12-Jun-2017), Heymeriks, Dr. J. (startdate 12-Jun-2017)<br>01009 / Kragten, Dr.<br>01010 / Swart, Dr. H.<br>01011/ de Groot, Dr. J.R.<br>01012/ Rooyer, Dr. F.A.<br>01013/ Van der Zwaan, Dr. C.<br>01014/ Boswijk, Dr. D.J.	Protocol version 3.0 23 March 2012<br>Protocol version 4.0 22 Oct 2014	13/APR/2012 (Non-WMO statement). The CTP and Draft CRF was send to EC in order to analyse if this study was to be submitted and it was confirmed that this study is not required to be submitted.<br>Not required as per Non-WMO statement

NETHERLANDS				
IRB or IEC (name/address)	IRB or IEC Chairperson	Centre number (5 digit number) / Investigator(s)	Protocol and/or Amendment number(s)	Date of Approval (DD/MMM/YYYY)
		01015/ Krenning, Dr. B.J.<br>01016 L Simmers, Dr. Tim<br>01017/ van Bergen, Dr. P.F.M.M.<br>01018/ Hemels, Dr. Maarten<br>01019/ Elvan, Dr. A.<br>01020 / van den Berg, Dr. B.J.<br>01021 / ten Berg, Dr. J.M.		

NORWAY				
IRB or IEC (name/address)	IRB or IEC Chairperson	Centre number (5 digit number) / Investigator(s)	Protocol and/or Amendment number(s)	Date of Approval (DD/MMM/YYYY)
**Regional Etisk Komite**<br>Postboks 1130<br>Blindern<br>0318 Oslo, Norway.<br>Visit adr:<br>Gullhaugveien 1-3<br>0484 Oslo, Norway	NA	16001 / Berz, Dr. Andrea<br>16002 / Bratland, Dr. Bjørn<br>16003 / Tveit, Dr. Arnljot<br>16004 / Sparby, Dr. Jon Arne<br>16005 / Bøhmer, Dr. Ellen<br>16006 / Heggelund, Dr. Geir<br>16007 / Finsen, Dr. Alexandra<br>16008 / Hall, Dr. Christian	Protocol version 3.0 23 March 2012<br><br>Protocol version 4.0 22 Oct 2014	01/JUN/2012<br><br>13/FEB/2015
**Regional Etisk Komite**<br>Postboks 1130<br>Blindern<br>0318 Oslo, Norway.<br>Visit adr:<br>Gullhaugveien 1-3<br>0484 Oslo, Norway	NA	16006 / Heggelund, Dr. Geir	Protocol version 3.0 23 March 2012<br><br>Protocol version 4.0 22 Oct 2014	01/JUN/2012<br><br>03/JUL/2014

PERU				
IRB or IEC (name/address)	IRB or IEC Chairperson	Centre number (5 digit number) / Investigator(s)	Protocol and/or Amendment number(s)	Date of Final Approval (DD/MMM/YYYY)
Comité de Bioética Red asistencia Sabogal / Jirón Colina 1081, Bellavista 07011, Peru	Edgar Ruecas Enriquez	27011 / Escudero, Aldo Edwin Rodriguez	Protocol - version 4.0 - dated 07-Jun-2013<br>Local Amendment - version 1.0 - dated 15-Oct-2014	15/Apr/2016
Comité de Ética para investigación Asociación Benéfica Prisma / Av. Santo Toribio 115, 5to piso, San Isidro, Peru	Salomón Zavala Sarrio	27012 / Llerena Navarro, Nassip Carlo Justo	Protocol - version 4.0 - dated 07-Jun-2013<br>Local Amendment - version 1.0 - dated 15-Oct-2014	20/Aug/2015
Comité de Ética para investigación Asociación Benéfica Prisma / Av. Santo Toribio 115, 5to piso, San Isidro, Peru	Salomón Zavala Sarrio	27013 / Chavez Ayala, Carlos	Protocol - version 4.0 - dated 07-Jun-2013<br>Local Amendment - version 1.0 - dated 15-Oct-2014	24/Jun/2015

PERU				
IRB or IEC (name/address)	IRB or IEC Chairperson	Centre number (5 digit number) / Investigator(s)	Protocol and/or Amendment number(s)	Date of Final Approval (DD/MMM/YYYY)
Comité de Ética para investigación Asociación Benéfica Prisma / Av. Santo Toribio 115, 5to piso, San Isidro, Peru	Salomón Zavala Sarrio	27016 / Manrique Hurtado, Herald Andres	Protocol - version 4.0 - dated 07-Jun-2013<br>Local Amendment - version 1.0 - dated 15-Oct-2014	24/Jun/2015
Comité de Ética para investigación Universidad San Martin de Porres / Av. Alameda del Corregidor 1531, Urb. Los Sirius, Las Viñas, La Molina, Lima 12	Amador Vargas Guerra	27018 / Negrón Miguel, Sandra Adela	Protocol - version 4.0 - dated 07-Jun-2013<br>Local Amendment - version 1.0 - dated 15-Oct-2014	08/Mar/2016
Comité de Ética para investigación Universidad San Martin de Porres / Av. Alameda del Corregidor 1531, Urb. Los Sirius, Las Viñas, La Molina, Lima 12	Amador Vargas Guerra	27002 / Cabrera, Jose Walter	Protocol - version 4.0 - dated 07-Jun-2013<br><br>Local Amendment - version 1.0 - dated 15-Oct-2014	19/Nov/2013<br><br>13/Jan/2015

PERU				
IRB or IEC (name/address)	IRB or IEC Chairperson	Centre number (5 digit number) / Investigator(s)	Protocol and/or Amendment number(s)	Date of Final Approval (DD/MMM/YYYY)
Comité de Ética para investigación Universidad San Martin de Porres / Av. Alameda del Corregidor 1531, Urb. Los Sirius, Las Viñas, La Molina, Lima 12	Amador Vargas Guerra	27003 / Medina Palomino, Feliz Alvaro	Protocol - version 4.0 - dated 07-Jun-2013<br><br>Local Amendment - version 1.0 - dated 15-Oct-2014	26/Nov/2013<br><br>05/May/2015
Comité de Ética para investigación Universidad San Martin de Porres / Av. Alameda del Corregidor 1531, Urb. Los Sirius, Las Viñas, La Molina, Lima 12	Amador Vargas Guerra	27007 / Berrospi, Percy	Protocol - version 4.0 - dated 07-Jun-2013<br><br>Local Amendment - version 1.0 - dated 15-Oct-2014	26/Nov/2013<br><br>23/Dec/2014
Comité de Ética para investigación Universidad San Martin de Porres / Av. Alameda del Corregidor 1531, Urb. Los Sirius, Las Viñas, La Molina, Lima 12	Amador Vargas Guerra	27009 / Guevara, Carolina	Protocol - version 4.0 - dated 07-Jun-2013<br><br>Local Amendment - version 1.0 - dated 15-Oct-2014	26/Nov/2013<br><br>23/Dec/2014

Poland				
IRB or IEC (name/address)	IRB or IEC Chairperson	Centre number (5 digit number) / Investigator(s)	Protocol and/or Amendment number(s)	Date of Final Approval (DD/MMM/YYYY)
Komisja Bioetyczna<br>Slaskiego Uniwersytetu Medycznego w Katowicach<br>Ul. Poniatowskiego 15<br>40-055<br><br>(not official name of EC in English)<br><br>[Ethic Committee of Medical Univesity of Silesia in Katowice<br>Poniatowskiego 15 street<br>40-055 post code]	Chairperson: Prof. MD, PhD Maria Trusz-Gluza	62001 – Wozakowska-kaplon<br>62002 – Streb<br>62003 – Morka<br>62004 – Nowalany-Kozielska<br>62005 – Miarka<br>62006 – Lenarczyk<br>62007 – Sokal<br>62008 – Gierba<br>62009 – Krysiak<br>62010 – Gniot<br>62011 – Musial<br>62013 – Lelonek<br>62014 – Kowalczyk<br>62015 – Sudnik	Protocol version 3.0	NA<br><br>CEC is NA for NIS in Poland
Komisja Bioetyczna<br>Slaskiego Uniwersytetu Medycznego w	Chairperson: Prof. MD, PhD Maria Trusz-Gluza	62001 – Wozakowska-kaplon<br>62002 – Streb	Final Protocol<br>Global Amendment 1,	NA

Katowicach<br>Ul. Poniatowskiego 15<br>40-055<br><br>(not official name of EC in English)<br><br>[Ethic Committee of Medical Univesity of Silesia in Katowice<br>Poniatowskiego 15 street<br>40-055 post code]		62003 – Morka<br>62004 – Nowalany-Kozielska<br>62005 – Miarka<br>62006 – Lenarczyk<br>62007 – Sokal<br>62008 – Gierba<br>62009 – Krysiak<br>62010 – Gniot<br>62011 – Musial<br>62013 – Lelonek<br>62014 – Kowalczyk<br>62015 – Sudnik	Protocol version 4.0	CEC is NA for NIS in Poland

PORTUGAL				
IRB or IEC (name/address)	IRB or IEC Chairperson	Centre number (5 digit number) / Investigator(s)	Protocol and/or Amendment number(s)	Date of Approval (DD/MMM/YYYY)
Centro Hospitalar Lisboa Ocidental, EPE – Hospital de Egas Moniz<br>Rua da Junqueira, 126<br>1349-019 Lisboa	NA	17001 / Adragão, Dr. Pedro	Protocol version 3.0 23 March 2012<br><br>Protocol version 4.0 22 Oct 2014	16/JAN/2013<br><br>06/ JAN /2016
Centro Hospitalar Lisboa Ocidental, EPE – Hospital de Egas Moniz<br>Rua da Junqueira, 126<br>1349-019 Lisboa	NA	17002 / Leitão, Dr. Ana	Protocol version 3.0 23 March 2012<br><br>Protocol version 4.0 22 Oct 2014	16/JAN/2013<br><br>18/NOV/2015
Centro Hospitalar de Lisboa Norte, EPE<br>Av.Prof.Egas Moniz<br>1649-035 Lisboa	NA	17003 / Pinto, Prof. Fausto	Protocol version 3.0 23 March 2012<br>Protocol version 4.0 22 Oct 2014	04/APR/2013<br><br>09/SEP/2015
Hospital Garcia de Orta, EPE<br>Av. Torrado da Silva,	NA	17005 / Valadão, Dr. Catarina	Protocol version 3.0 23 March 2012	09/JUL/2012

PORTUGAL				
IRB or IEC (name/address)	IRB or IEC Chairperson	Centre number (5 digit number) / Investigator(s)	Protocol and/or Amendment number(s)	Date of Approval (DD/MMM/YYYY)
Pragal<br>2801-951 Almada			Protocol version 4.0 22 Oct 2014	05/NOV/2015
Centro Hospitalar Cova da Beira, EPE<br>Quinta do Alvito<br>6200-251 Covilhã	NA	17006 / Martinez, Dr. Jorge	Protocol version 3.0 23 March 2012<br>Protocol version 4.0 22 Oct 2014	18/JUN/2012<br>14/OCT/2015
Centro Hospitalar de São João, E.P.E<br>Alameda Prof. Hernâni Monteiro<br>4200-319 Porto	NA	17009 / Fonseca Almeida, Dr. Luísa	Protocol version 3.0 23 March 2012<br>Protocol version 4.0 22 Oct 2014	11/OCT/2012<br>22/JAN/2016
Hospital da Luz<br>Avenida Lusíada, nº 100<br>1500-650 Lisboa	NA	17010 / Ferreira, Dr. Daniel	Protocol version 3.0 23 March 2012<br>Protocol version 4.0 22 Oct 2014	14/SEP/2012<br>08/OCT/2015

PORTUGAL				
IRB or IEC (name/address)	IRB or IEC Chairperson	Centre number (5 digit number) / Investigator(s)	Protocol and/or Amendment number(s)	Date of Approval (DD/MMM/YYYY)
Hospital de Santiago<br>Estrada Nacional 10, km 37,<br>2900-722 Setúbal	NA	17011 / Ferreira dos Santos, Dr. José	Protocol version 3.0 23 March 2012<br><br>Protocol version 4.0 22 Oct 2014	23/NOV/2012<br><br>12/NOV/2015
Hospital do Espírito Santo – de Évora, EPE<br>Largo Senhor da Pobreza<br>7000-811 Évora	NA	17013 / Dionísio, Dr. Pedro	Protocol version 3.0 23 March 2012<br><br>Protocol version 4.0 22 Oct 2014	14/MAR/2013<br><br>29/OCT/2015
Hospital de Faro, EPE<br>Rua Leão Penedo<br>8000-386 Faro	NA	17016 / Candeias, Dr. Rui	Protocol version 3.0 23 March 2012<br><br>Protocol version 4.0 22 Oct 2014	20/NOV/2012<br><br>16/FEB/2016

PORTUGAL				
IRB or IEC (name/address)	IRB or IEC Chairperson	Centre number (5 digit number) / Investigator(s)	Protocol and/or Amendment number(s)	Date of Approval (DD/MMM/YYYY)
Centro Hospitalar de Lisboa Norte, EPE<br>Av.Prof.Egas Moniz<br>1649-035 Lisboa	NA	17021 / Fonseca, Dr. Catarina	Protocol version 3.0 23 March 2012<br><br>Protocol version 4.0 22 Oct 2014	17/OCT/2013<br><br>09/SEP/2015

ROMANIA				
IRB or IEC (name/address)	IRB or IEC Chairperson	Centre number (5 digit number) / Investigator(s)	Protocol and/or Amendment number(s)	Date of Final Approval (DD/MMM/YYYY)
National Bioethics Commission of Medicine and Medical Devices, Şos. Ştefan cel Mare nr. 19-21, sector 2, Bucuresti, Romania	Prof. Dr. Dinu Antonescu	40001/Militaru<br>40002/Miu<br>40003/Muresan<br>40004/Mut-Vitcu<br>40005/Podoleanu<br>40006/Pop<br>40007/Popa<br>40008/Tesloianu<br>40009/Vinereanu<br>40010/Nechita<br>40011/Octavian<br>40012/Cindea-Nica<br>40013/Minescu<br>40014/Furntelata<br>40015/Sorin Antonescu	Protocol version 3.0 / 23 Mar 2012	28/NOV/2012
National Bioethics Commission of Medicine and Medical Devices, Şos. Ştefan cel Mare nr. 19-21, sector 2, Bucuresti,	Prof. Dr. Dinu Antonescu	40001/Militaru<br>40002/Miu<br>40003/Muresan<br>40004/Mut-Vitcu<br>40005/Podoleanu	Protocol version 4.0 / 22 Oct 2014	26/JAN/2015

ROMANIA				
IRB or IEC (name/address)	IRB or IEC Chairperson	Centre number (5 digit number) / Investigator(s)	Protocol and/or Amendment number(s)	Date of Final Approval (DD/MMM/YYYY)
Romania		40006/Pop<br>40007/Popa<br>40008/Tesloianu<br>40009/Vinereanu<br>40010/Nechita<br>40011/Octavian<br>40012/Cindea-Nica<br>40013/Minescu<br>40014/Furntelata<br>40015/Sorin Antonescu		

RUSSIA				
IRB or IEC (name/address)	IRB or IEC Chairperson	Centre number (5 digit number) / Investigator(s)	Protocol and/or Amendment number(s)	Date of Final Approval (DD/MMM/YYYY)
Ethics Committee of "Perm regional clinical hospital for war veterans" / 6 Podlesnaya street614097, Perm, Russian Federation	T.V. Odegova	33001/Natalya Koziolova	Final .Protocol version 4.0<br>Global Amendment 1 dated 07 Jun 2013	19/NOV/2013
Ethics Committee of "Perm regional clinical hospital for war veterans" / 6 Podlesnaya street614097, Perm, Russian Federation	T.VERSION Odegova	33001/Natalya Koziolova	Clinical trial protocol version 3.0 dated 19 Jul 2011	16/OCT/2012
Local Ethics Committee of<br>SBFI "Novosibirsk Regional<br>Clinical Cardiological Dispensary"/630047, Russia, Novosibirsk, Zalesskogo Str., 6, bid. 8,	I.A. Kebbel	33002/Sergei Zenin	Final Protocol version 4.0<br>Global Amendment 1 dated 07 Jun 2013	3/OCT/2013
Local Ethics Committee of<br>SBFI "Novosibirsk Regional<br>Clinical Cardiological Dispensary"/630047, Russian Federation, Novosibirsk, Zalesskogo	I.A. Kebbel	33002/Sergei Zenin	Clinical trial protocol version 3.0 dated 19 Jul 2011	30/OCT/2012

RUSSIA				
IRB or IEC (name/address)	IRB or IEC Chairperson	Centre number (5 digit number) / Investigator(s)	Protocol and/or Amendment number(s)	Date of Final Approval (DD/MMM/YYYY)
Str., 6, bid. 8,				
Local Ethics Committee of the Saint-Petersburg State Budget Institution of Healthcare "Pokrovskaya City Hospital"/ 199106, Russian Federation, Saint-Petersburg,Bolshoy prosp.V.O., 85	A.A. Balyabin	33003/Tatiana Novikova	Final Protocol version 4.0 Global Amendment 1 dated 07 Jun 2013	07/NOV/2013
Local Ethics Committee of the Saint-Petersburg State Budget Institution of Healthcare "Pokrovskaya City Hospital"/ 199106, Russian Federation, Saint-Petersburg,Bolshoy prosp.V.O., 85	A.A. Balyabin	33003/Tatiana Novikova	Clinical trial protocol version 3.0 dated 19 Jul 2011	26/DEC/2012
Intercollegiate Committee of Ethics/build.2, 37, per Gagarinsky, Moscow, 119002 Russian Federation	E.A. Volskaya	33006/Andrey Ezhov	Final Protocol version 4.0 Global Amendment 1 dated 07 Jun 2013	26/SEP/2013
Intercollegiate Committee of Ethics/build.2, 37, per Gagarinsky, Moscow, 119002 Russian Federation	E.A. Volskaya	33006/Andrey Ezhov	Clinical trial protocol version 3.0 dated 19 Jul 2011	13/JUL/2012

RUSSIA				
IRB or IEC (name/address)	IRB or IEC Chairperson	Centre number (5 digit number) / Investigator(s)	Protocol and/or Amendment number(s)	Date of Final Approval (DD/MMM/YYYY)
.Local Ethics Committee of MBPHI"Kemerovo Cardiology Dispensary" and Scientific-Research Institute for complex issues of cardiovascular diseases, Siberian branch of the Russian Academy of Medical Sciences"/ 650002, Russian Federation, Kemerovo, Sosnovy boulevard, 6	E.V. Grigoriev	33009/Olga Barbarash	Final Protocol version 4.0<br>Global Amendment 1 dated 07 Jun 2013	11/NOV/2013
Local Ethics Committee of MBPHI"Kemerovo Cardiology Dispensary" and Scientific-Research Institute for complex issues of cardiovascular diseases, Siberian branch of the Russian Academy of Medical Sciences"/ 650002, Russian Federation, Kemerovo, Sosnovy boulevard, 6	E.V. Grigoriev	33009/Olga Barbarash	Clinical trial protocol version 3.0 dated 19 Jul 2011	01/OCT/2012
Local Ethics Committee at State budgetary Healthcare Institution "Samara<br>regional clinical cardiology dispenser"/ 443070, Russian	A.A.Tukhbatova	33015/Dmitry Duplyakov	Final Protocol version 4.0<br>Global Amendment 1 dated 07 Jun 2013	31/JAN/2014

RUSSIA				
IRB or IEC (name/address)	IRB or IEC Chairperson	Centre number (5 digit number) / Investigator(s)	Protocol and/or Amendment number(s)	Date of Final Approval (DD/MMM/YYYY)
Federation, Samara, Aerodromnaya str, 43				
Local Ethics Committee at State budgetary Healthcare Institution "Samara regional clinical cardiology dispenser"/443070, Russian Federation, Samara, Aerodromnaya str, 43	A.A.Tukhbatova	33015/Dmitry Duplyakov	Clinical trial protocol version 3.0 dated 19 Jul 2011	02/NOV/2012
Local Ethics Committee of IGMAPO/ 100, Yubileyny district, Irkutsk, 664079, Russian Federation	T.L. Moroz	33016/Konstantin Protasov	Final Protocol version 4.0 Global Amendment 1 dated 07 Jun 2013	07/NOV/2013
Local Ethics Committee of IGMAPO/ 100, Yubileyny district, Irkutsk, 664079, Russian Federation	T.L. Moroz	33016/Konstantin Protasov	Clinical trial protocol version 3.0 dated 19 Jul 2011	18/OCT/2012
Local Ethics Committee of Altaiskyi Regional Cardiology Dispensary/656055, Russia Federation, Barnaul, Malakhova Str" 46,	E.V.Strubovshchikova	33024/Galina Chumakova	Final Protocol version 4.0 Global Amendment 1 dated 07 Jun 2013	28/FEB/2014

RUSSIA				
IRB or IEC (name/address)	IRB or IEC Chairperson	Centre number (5 digit number) / Investigator(s)	Protocol and/or Amendment number(s)	Date of Final Approval (DD/MMM/YYYY)
Local Ethics Committee of Altaiskyi Regional Cardiology Dispensary/656055, Russian Federation, Barnaul, Malakhova Str" 46,	E.V.Strubovshchikova	33024/Galina Chumakova	Clinical trial protocol version 3.0 dated 19 Jul 2011	11/DEC/2012
Ethics Committee at SBHI "Krai Clinical Hospital # 2"/350012, Russian Federation, Krasnodar, Krasnykh Partizan Str" 6, bld.2	M.I.Veselenko	33026/Galina Ivanchura	Final Protocol version 4.0<br>Global Amendment 1 dated 07 Jun 2013	13/NOV/2013
Ethics Committee at SBHI "Krai Clinical Hospital # 2"/350012, Russian Federation, Krasnodar, Krasnykh Partizan Str" 6, bld.2	M.I.Veselenko	33026/Galina Ivanchura	Clinical trial protocol version 3.0 dated 19 Jul 2011	25/JAN/2013
Local Ethics Committee at State healthcare institution “Ryazan regional clinical hospital"/390039, Russian Federation, Ryazan, Internationalnaya Str., 3a,	V.N.Abrosimov	33028/Alexey Nizov	Final Protocol version 4.0<br>Global Amendment 1 dated 07 Jun 2013	13/DEC/2013
Local Ethics Committee at State healthcare institution “Ryazan regional clinical hospital"/390039, Russian Federation, Ryazan, Internationalnaya	V.N.Abrosimov	33028/Alexey Nizov	Clinical trial protocol version 3.0 dated 19 Jul 2011	24/JAN/2013

RUSSIA				
IRB or IEC (name/address)	IRB or IEC Chairperson	Centre number (5 digit number) / Investigator(s)	Protocol and/or Amendment number(s)	Date of Final Approval (DD/MMM/YYYY)
Str., 3a,				
Local Ethics CommiHee at LLC "Clinic of new technologies in Medicine"/Russian Federation, 140094, Dzerzhinsky, Ugreshskaya Str., 20.	L.Kalinina	33029/Larisa Kalinina	Final Protocol version 4.0<br>Global Amendment 1 dated 07 Jun 2013	08/NOV/2013
Local Ethics CommiHee at LLC "Clinic of new technologies in Medicine"/Russian Federation, 140094, Dzerzhinsky, Ugreshskaya Str., 20.	L.Kalinina	33029/Larisa Kalinina	Clinical trial protocol version 3.0 dated 19 Jul 2011	25/JAN/2013
Local Ethics Committee at federal state budget institution Russian cardiology research and production complex of the Ministry of healthcare of the Russian Federation /Russian Federation, Moscow, 121552, 3rd Cherepkovskaya str., 15A	M.Ya.Ruda	33030/Sergey Golitsyn	Final Protocol version 4.0<br>Global Amendment 1 dated 07 Jun 2013	25/NOV/2013
Local Ethics Committee at federal state budget institution Russian cardiology research and production complex of the Ministry of healthcare of the Russian Federation /Russian Federation, Moscow, 121552, 3rd	M.Ya.Ruda	33030/Sergey Golitsyn	Clinical trial protocol version 3.0 dated 19 Jul 2011	26/NOV/2012

RUSSIA				
IRB or IEC (name/address)	IRB or IEC Chairperson	Centre number (5 digit number) / Investigator(s)	Protocol and/or Amendment number(s)	Date of Final Approval (DD/MMM/YYYY)
Cherepkovskaya str., 15A				

Saudi Arabia				
**IRB or IEC (name/address)**	**IRB or IEC Chairperson**	**Centre number (5 digit number) / Investigator(s)**	**Protocol and/or Amendment number(s)**	**Date of Final Approval (DD/MMM/YYYY)**
Institutional Review Board, King Fahad Medical City, Riyadh Saudi Arabia	Prof Omar Hasan K Kasule Sr	30001/ O Mutwali (EC approval received and this site was initiated but no patients were recruited at this site)	Protocol version 3.0 23/MAR/2012 Protocol version 4.0 7/JUN/2013	15/Mar/2014
Research Ethics Committee, Armed Forces Hospital, Khamis Almushayat, SaudiArabia.	Dr. Ezzadien M. Rabie	30002/ M Alshehri		08/Dec/2013
Institutional Review Board, King Fahad Medical City, Riyadh Saudi Arabia	Prof Omar Hasan K Kasule Sr	30003/ G Hussein		14/Feb/2014
Institutional Review Board, King Fahad Medical City, Riyadh Saudi Arabia	Prof Omar Hasan K Kasule Sr	30004/ H El-Sayed		01/Mar/2014

Saudi Arabia				
**IRB or IEC (name/address)**	**IRB or IEC Chairperson**	**Centre number (5 digit number) / Investigator(s)**	**Protocol and/or Amendment number(s)**	**Date of Final Approval (DD/MMM/YYYY)**
Institutional Review Board, King Fahad Medical City, Riyadh Saudi Arabia	Prof Omar Hasan K Kasule Sr	30005/ A Alasfar		05/Nov/2013
Institutional Review Board, King Fahad Medical City, Riyadh Saudi Arabia	Prof Omar Hasan K Kasule Sr	30006/ M Sirajuddin		01/Mar/2014
Institutional Review Board, King Fahad Medical City, Riyadh Saudi Arabia	Prof Omar Hasan K Kasule Sr	30007/ A Ali		01/Mar/2014
Research Ethics committee, King Abdulaziz University, Jeddah, KSA.	Prof Hasan Alzahrani	30008/ K Al Ghalayini		27/Feb/2014

Saudi Arabia				
**IRB or IEC (name/address)**	**IRB or IEC Chairperson**	**Centre number (5 digit number) / Investigator(s)**	**Protocol and/or Amendment number(s)**	**Date of Final Approval (DD/MMM/YYYY)**
Institutional Review Board,<br>King Fahad Medical City,<br>Riyadh Saudi Arabia	Prof Omar Hasan K Kasule Sr	30009/ F Qaddoura		14/Feb/2014

Singapore				
IRB or IEC (name/address)	IRB or IEC Chairperson	Centre number (5 digit number) / Investigator(s)	Protocol and/or Amendment number(s)	Date of Final Approval (DD/MMM/YYYY)
Centralised Institutional Review Board (CIRB)<br>168 Jalan Bukit Merah,<br>#06-08 Tower<br>3 Connection One<br>Singapore 150168	NA	45001/ Angela Koh<br><br>45003/ Dr. Tu Tian Ming	Protocol version # 3<br>19/ JUL/ 2011<br>Protocol version #4<br>07/JUN/2013	05/JUN/2013<br><br>13/AUG/2013
Domain Specific Review Board<br>Nexus at One-North (South Tower), No. 3 Fusionopolis Link, #03-08, Singapore 138543	NA	45002/ Vijay Kumar Sharma<br><br>45003/ Dr. Tu Tian Ming	Protocol version #4<br>07/JUN/2013	16/AUG/2013
Parkway Independent Ethics Committee (PIEC)<br>ParkwayHealth Day Surgery and Medical Centre (PDSMC)<br>363 Balestier Road, #05-03, Singapore 329784	NA	45004/ Hsu Li Fern	Protocol version #4<br>07/JUN/2013	16/OCT/2013

SLOVENIA				
IRB or IEC (name/address)	IRB or IEC Chairperson	Centre number (5 digit number) / Investigator(s)	Protocol and/or Amendment number(s)	Date of Final Approval (DD/MMM/YYYY)
Komisija Republike Slovenije za medicinsko etiko<br>Štefanova 5<br>1000 Ljubljana<br>Slovenia	Not available	42001/Kovačic, Dragan; 42002/Mavri, Alenka	Protocol version 3.0 23/MAR/2012<br>Protocol version 4.0 22/OCT/2014	18/DEC2012<br>12/FEB/2015

South Africa				
IRB or IEC (name/address)	IRB or IEC Chairperson	Centre number (5 digit number) / Investigator(s)	Protocol and/or Amendment number(s)	Date of Final Approval (DD/MMM/YYYY)
Pharma Ethics<br>123 Amcor Road<br>LYTTELTON MANOR 0157	Dr. C. S. J. Duvenhage	48002/ Abelson, M J<br>48006/ Ranjith, N<br>48007/ Adams, L<br>48008/ Duki, M<br>48010/ Moodley, R	Protocol Version 3.0	04/APR/2013

South Korea				
IRB or IEC (name/address)	IRB or IEC Chairperson	Centre number (5 digit number) / Investigator(s)	Protocol and/or Amendment number(s)	Date of Final Approval (DD/MMM/YYYY)
Korea University Anam Hospital Institutional Review Board /126-1, Anam-dong 5-ga, Seongbuk-gu Seoul, Korea	Byung Soo Kim	11001/ Young Hoon Kim	Protocol version 3.0 23/MAR/2012	22/OCT/2012
	Tae Jin Yoon	11001/ Young Hoon Kim	Protocol version 4.0 7/JUN/2013	31/OCT/2013
Asan Medical Center Institutional Review Board /88, Olympic-ro 43-gil, Songpa-gu, Seoul 138-736, Korea	Jong Woo Chung	11002/ Nam	Protocol version 3.0 23/MAR/2012	02/NOV/2012
	Jong Woo Chung	11002/ Nam	Protocol version 4.0 7/JUN/2013	14/NOV/2013
Eulji University Hospital’s Institutional Review Board/ 1306 Dunsan 2-dong, Seo-gu, Daejeon 302-799, Korea	Soo-Joo Lee	11003/ Kyung Tae Jung	Protocol version 3.0 23/MAR/2012	12/MAR/2013

South Korea				
IRB or IEC (name/address)	IRB or IEC Chairperson	Centre number (5 digit number) / Investigator(s)	Protocol and/or Amendment number(s)	Date of Final Approval (DD/MMM/YYYY)
	Soo-Joo Lee	11003/ Kyung Tae Jung	Protocol version 4.0 7/JUN/2013	25/OCT/2013
Seoul National University Hospital Institutional Review Board / 101 Daehak-ro, Jongno-gu, Seoul 110-744, Korea	Byung Joo, Park	11004/ Oh	Protocol version 3.0 23/MAR/2012	07/DEC/2012
	Byung Joo, Park	11004/ Oh	Protocol version 4.0 7/JUN/2013	13/Nov/2013
Ajou University hospital Institutional Review Board/164 World cup-ro, Yeongtong-gu, Suwon-si, Gyeonggi-do 443-721, Korea	Mi-Son Chun	11005/ Gyo-Seung Hwang	Protocol version 3.0 23/MAR/2012	15 Jan 2013
	Jin Hyuk Choi	11005/ Gyo-Seung Hwang	Protocol version 4.0 7/JUN/2013	25/Nov/2013

South Korea				
IRB or IEC (name/address)	IRB or IEC Chairperson	Centre number (5 digit number) / Investigator(s)	Protocol and/or Amendment number(s)	Date of Final Approval (DD/MMM/YYYY)
Wonkwang University Hospital Institutional Review Board/ 895 Muwang-Ro, Iksan, Jeonlabuk-do, 570-711, Korea	Jeon Cheol Hong	11006/ Nam Ho Kim	Protocol version 3.0 23/MAR/2012	11/Dec/2012
	Moo-Rim Park	11006/ Nam Ho Kim	Protocol version 4.0 7/JUN/2013	12/Nov/2013
Institutional Review Board of Inje University Busan Paik Hospital /Bokji-ro 75, Busanjin-gu, Busan, Korea	Jae Il, Jeong	11007/ Dae Kyeong Kim	Protocol version 3.0 23/MAR/2012	29/Nov/2012
	Jae Il, Jeong	11007/ Dae Kyeong Kim	Protocol version 4.0 7/JUN/2013	13/Nov/2013
Kosin University Gospel Hospital Institutional Review Board/ 262, Gamcheon-ro, Seo-gu, Busan, 602-702, Korea	Tae Won Jang	11008/ Tae Joon Cha	Protocol version 3.0 23/MAR/2012	11/Dec/2012

South Korea				
IRB or IEC (name/address)	IRB or IEC Chairperson	Centre number (5 digit number) / Investigator(s)	Protocol and/or Amendment number(s)	Date of Final Approval (DD/MMM/YYYY)
	Tae Won Jang	11008/ Tae Joon Cha	Protocol version 4.0 7/JUN/2013	14/Nov/2013
Institutional Review Board, Seoul St.Mary's Hospital 222 Banpo-daero, Seocho-gu, Seoul 137-701, Korea	Sang-hong, Baek	11009/ Yong Seog Oh	Protocol version 3.0 23/MAR/2012	10 Jan 2013
	Myung A, Lee	11009/ Yong Seog Oh	Protocol version 4.0 7/JUN/2013	28 Nov 2013
Institutional Review Board Seoul National University Bundang Hospital/82 Gumi-ro, 173 Beon-gil, Bundang-gu, Seongnam-si, Gyeonggi-do 463-707, Korea	Chae-Seo Rhee	11010/ Dong Ju Choi	Protocol version 3.0 23/MAR/2012	19 Jan 2013
	Chae-Seo Rhee	11010/ Dong Ju Choi	Protocol version 4.0 7/JUN/2013	16 Nov 2013

South Korea				
IRB or IEC (name/address)	IRB or IEC Chairperson	Centre number (5 digit number) / Investigator(s)	Protocol and/or Amendment number(s)	Date of Final Approval (DD/MMM/YYYY)
Pusan National University Hospital Instituional Review Board / 179 Gudeok-ro, Seo-gu, Busan 602-739, Korea	Cho, Byung Mann	11011/ Cha	Protocol version 3.0 23/MAR/2012	10 Dec 2012
	Cho, Byung Mann	11011/ Cha	Protocol version 4.0 7/JUN/2013	25 Oct 2013
Severance Hospital, Institutional Review Board	Hee Cheol Kang	11012/ Lee	Protocol version 3.0 23/MAR/2012	16/NOV/2012
	Hee Cheol Kang	11012/ Lee	Protocol version 4.0 7/JUN/2013	22/NOV/2013
Yeungnam University Hospital Institutional Review Board / 317-1 Daemyeong 5-dong, Nam-gu, Daegu, Korea	Yong Jin, Kim	11013/ Shin	Protocol version 3.0 23/MAR/2012	06/DEC/2012
	Yong Jin, Kim	11013/ Shin	Protocol version 4.0 7/JUN/2013	28/Nov/2013

South Korea				
IRB or IEC (name/address)	IRB or IEC Chairperson	Centre number (5 digit number) / Investigator(s)	Protocol and/or Amendment number(s)	Date of Final Approval (DD/MMM/YYYY)
Keimyung Medical Center Institutional Review Board / 56 Dalseong-ro, Jung-gu, Daegu 700-712, Korea	Jeong-Ho Rhee	11014/ Yoon Nyun Kim	Protocol version 3.0 23/MAR/2012	01/Feb/2013
	Jeong-Ho Rhee	11014/ Yoon Nyun Kim	Protocol version 4.0 7/JUN/2013	17/Dec/2013
Pusan National University Yangsan Hospital Institutional Review Board / Beomeo-ri, Mulgeum-eup, Yangsan-si, Gyeong Nam, 626-770, Korea	Sang-Yeoup, Lee	11015/ Jun Kim	Protocol version 3.0 23/MAR/2012	03/DEC/2012
	Hwang, Tae Ho	11015/ Jun Kim	Protocol version 4.0 7/JUN/2013	30/OCT/2013
Chonnam National University Hospital Institutional Review Board/ 42 Jebong-ro, Donggu, Gwangju 501-757, Korea	Young-Ho Won	11016/ Hyung Wook Park	Protocol version 3.0 23/MAR/2012	20/DEC/2012

South Korea				
IRB or IEC (name/address)	IRB or IEC Chairperson	Centre number (5 digit number) / Investigator(s)	Protocol and/or Amendment number(s)	Date of Final Approval (DD/MMM/YYYY)
	Young-Ho Won	11016/ Hyung Wook Park	Protocol version 4.0 7/JUN/2013	14/NOV/2013
Inje University Haeundae Paik Hospital Institutional Review Board / 875 Haeun-daero, Haendae-gu, Busan 612-862, Korea	Min-Seob Song	11017/ Doo II Kim	Protocol version 3.0 23/MAR/2012	02/JAN/2013
	Min-Seob Song	11017/ Doo II Kim	Protocol version 4.0 7/JUN/2013	30/OCT/2013
Samsung Medical Center Institutional Review Board / 50 Irwon-dong, Gangnam gu, Seoul 135-710, Korea	Lee, Jung-Il	11018/ June Soo Kim	Protocol version 3.0 23/MAR/2012	05/DEC/2012
	Chung, Sung Soo	11018/ June Soo Kim	Protocol version 4.0 7/JUN/2013	30/OCT/2013

South Korea				
IRB or IEC (name/address)	IRB or IEC Chairperson	Centre number (5 digit number) / Investigator(s)	Protocol and/or Amendment number(s)	Date of Final Approval (DD/MMM/YYYY)
Kyungpook National University Hospital Institutional Review Board, 130 Dongdeok-ro, Jung-gu, Daegu 700-721, Korea	In-Taek, Kim	11019/ Yong Keun Cho	Protocol version 3.0 23/MAR/2012	04/DEC/2012
	In-Taek, Kim	11019/ Yong Keun Cho	Protocol version 4.0 7/JUN/2013	28/OCT/2013
Jeju National University Hospital Institutional Review Board / Aran 13gil, Jeju-si, Jeju Special Self-Governing Province 690-767, Korea	Chang-Sub Lee	11020/ Ki Seok Kim	Protocol version 3.0 23/MAR/2012	21/JAN/2013
	Chang-Sub Lee	11020/ Ki Seok Kim	Protocol version 4.0 7/JUN/2013	05/NOV/2013
Gyeongsan National University Hospital Institutional Review Board / 79 Gangnam-ro, Jinju-si, Gyeongsangnam-do 660-702, Korea	Hee-Suk Shin	11021/ Choong Hwan Kwak	Protocol version 3.0 23/MAR/2012	04/FEB/2013

South Korea				
IRB or IEC (name/address)	IRB or IEC Chairperson	Centre number (5 digit number) / Investigator(s)	Protocol and/or Amendment number(s)	Date of Final Approval (DD/MMM/YYYY)
	Hee-Suk Shin	11021/ Choong Hwan Kwak	Protocol version 4.0 7/JUN/2013	04/NOV/2013
Chonbuk National University Hospital Institutional Review Board, 634-18 Geuman-dong, Dukjin-gu, Chonju, Chonbuk Chonbuk 562-712, Korea	Sung Kwang, Park	11022/ Jei Keon Chae	Protocol version 3.0 23/MAR/2012	22/JAN/2013
	Sung Kwang, Park	11022/ Jei Keon Chae	Protocol version 4.0 7/JUN/2013	07/NOV/2013
St. Paul's Hospital Institutional Review Board / Jeonnong 1-dong, Dongdaemun-gu, Seoul 130-709, Korea	Sang-hong, Baek	11023/	Protocol version 3.0 23/MAR/2012	10/JAN/2013
	Hyun-Suk, Song	11023/ Sung Won Jang	Protocol version 4.0 7/JUN/2013	08/NOV/2013

South Korea				
IRB or IEC (name/address)	IRB or IEC Chairperson	Centre number (5 digit number) / Investigator(s)	Protocol and/or Amendment number(s)	Date of Final Approval (DD/MMM/YYYY)
Daegu Fatima Hospital Institutional Review Board / 183 Ayang-ro, Dong-gu, Daegu, Korea	Do Yeong, Song	11024/ Jung	Protocol version 3.0 23/MAR/2012	12/DEC/2012
	Do Yeong, Song	11024/ Jung	Protocol version 4.0 7/JUN/2013	27/NOV/2013
Kyung Hee University Hospital Institutional Review Board / 23 Kyungheedae-ro, Dondaemun-gu, 130-872, Seoul, Korea	Seong Ho, Cha	11025/ Jin Bae Kim	Protocol version 3.0 23/MAR/2012	13/DEC/2012
	Seong Ho, Cha	11025/ Jin Bae Kim	Protocol version 4.0 7/JUN/2013	18/NOV/2013

South Korea				
IRB or IEC (name/address)	IRB or IEC Chairperson	Centre number (5 digit number) / Investigator(s)	Protocol and/or Amendment number(s)	Date of Final Approval (DD/MMM/YYYY)
Sejong General Hospital Institutional Review Board/ 91-121 Sosabon-dong, Sosagu Bucheon-si, Gyeonggi-do, Korea	Gi Seok, Lee	11026/ Suk Keun Hong	Protocol version 3.0 23/MAR/2012	27/MAY/2013
	Gi Seok, Lee	11026/ Suk Keun Hong	Protocol version 4.0 7/JUN/2013	09/Dec/2013
Ulsan University Hospital Institutional Review Board / 877 Bangeojinsunhwan-doro, Dong-gu, Ulsan 682-714, Korea	Joon Ho, Ahn	11027/	Protocol version 3.0 23/MAR/2012	03/JAN/2013
	Joon Ho, Ahn	11027/	Protocol version 4.0 7/JUN/2013	14/NOV/2013
Kangdong Sacred Heart Hospital Institutional Review Board / 150 Seongan-ro, Gangdong-gu, Seoul 134-814 , Korea	Soo-Young Kim	11028/	Protocol version 3.0 23/MAR/2012	18/DEC/2012

South Korea				
IRB or IEC (name/address)	IRB or IEC Chairperson	Centre number (5 digit number) / Investigator(s)	Protocol and/or Amendment number(s)	Date of Final Approval (DD/MMM/YYYY)
	Soo-Young Kim	11028/	Protocol version 4.0 7/JUN/2013	22/NOV/2013
Dong-A University Hospital Institutional Review Board / 26 Daesingongwon-ro, Seo-gu, Busan 602-715, Korea	Lee, Soo-il	11029/	Protocol version 3.0 23/MAR/2012	04/JAN/2013
	Lee, Ki-Nam	11029/	Protocol version 4.0 7/JUN/2013	20/NOV/2013
The Catholic University of Korea Daejeon ST. Mary;s Hospital Institutional Review Board / 520-2, Daeheung-dong, Joong-gu, Daejeon, Korea	Hyeong Jin Lee	11030/	Protocol version 3.0 23/MAR/2012	18/MAR/2013
	Hyeong Jin Lee	11030/	Protocol version 4.0 7/JUN/2013	05/DEC/2013

South Korea				
IRB or IEC (name/address)	IRB or IEC Chairperson	Centre number (5 digit number) / Investigator(s)	Protocol and/or Amendment number(s)	Date of Final Approval (DD/MMM/YYYY)
VHS Medical Center Institutional Review Board / 61-53 Jinhwangdo-ro Gangdong-gu, Seoul 05368, Korea	Jeong Young Jin	11031/		14/APR/2016

SPAIN				
IRB or IEC (name/address)	IRB or IEC Chairperson	Centre number (5 digit number) / Investigator(s)	Protocol and/or Amendment number(s)	Date of Final Approval (DD/MMM/YYYY)
CEIm provincial de Sevilla<br>Hospital Universitario Virgen Macarena Avda. Dr. Fedriani, 3 – 41009 Sevilla	Victor Sánchez Margalef	03001 / Dr. Gonzalo Barón Esquivias	Protocol version 3.0 23/MAR/2012<br><br>Local Amendment 1 (22/AUG/2012)<br><br>Protocol version 4.0 22/OCT/2014	07/JUN/2012<br><br>25/OCT/2012<br><br>27/JAN/2015
CEIm Hospital Universitario La Paz.<br>Paseo de la Castellana, 261;<br>Planta 8ª Hospital General<br>28046 MADRID	Almudena Castro Conde	03002 / Dr. José Luis López-Sendón	Protocol version 3.0 23/MAR/2012<br><br>Local Amendment 1 (22/AUG/2012)<br><br>Protocol version 4.0 22/OCT/2014	07/JUN/2012<br><br>25/OCT/2012<br><br>27/JAN/2015

SPAIN				
IRB or IEC (name/address)	IRB or IEC Chairperson	Centre number (5 digit number) / Investigator(s)	Protocol and/or Amendment number(s)	Date of Final Approval (DD/MMM/YYYY)
CEIm provincial de Málaga<br>Hospital Regional Universitario de Málaga<br>U.G. de Ensayos Clínicos - Pabellón A - Planta 7ª<br>Avda. Carlos Haya, s/n<br>29010 - Málaga	Manuel Herrera Gutiérrez	03003 / Dr. Eduardo de Teresa Galvan	Protocol version 3.0 23/MAR/2012<br><br>Local Amendment 1 (22/AUG/2012)<br><br>Protocol version 4.0 22/OCT/2014	07/JUN/2012<br><br>25/OCT/2012<br><br>27/JAN/2015
CEIm Hospital Universitario Virgen de la Arrixaca.<br>Ctra Madrid-Cartagena s/n<br>30120 Murcia	Antonio Piñero Madrona	03005 / Dr. Francisco Marín Ortuño	Protocol version 3.0 23/MAR/2012<br><br>Local Amendment 1 (22/AUG/2012)<br><br>Protocol version 4.0 22/OCT/2014	07/JUN/2012<br><br>25/OCT/2012<br><br>27/JAN/2015

SPAIN				
IRB or IEC (name/address)	IRB or IEC Chairperson	Centre number (5 digit number) / Investigator(s)	Protocol and/or Amendment number(s)	Date of Final Approval (DD/MMM/YYYY)
CEIm Hospital Clínic de Barcelona<br>Plta 0, porta 6B<br>C/ Villarroel, 170 / 08036 Barcelona	Alicia Bernal	03009 / Dr. Lluis Mont Girbau	Protocol version 3.0 23/MAR/2012<br><br>Local Amendment 1 (22/AUG/2012)<br><br>Protocol version 4.0 22/OCT/2014	07/JUN/2012<br><br>25/OCT/2012<br><br>27/JAN/2015
CEIm Hospital Universitario Doctor Peset<br>C/ San Lázaro s/n. Edificio de Riesgos Laborales 1º Piso. 46017 Valencia.	Pilar Codoñer Franch	03010 / Dr. José Luis Llisterri Caro	Protocol version 3.0 23/MAR/2012<br><br>Local Amendment 1 (22/AUG/2012)<br><br>Protocol version 4.0 22/OCT/2014	07/JUN/2012<br><br>25/OCT/2012<br><br>27/JAN/2015

SPAIN				
IRB or IEC (name/address)	IRB or IEC Chairperson	Centre number (5 digit number) / Investigator(s)	Protocol and/or Amendment number(s)	Date of Final Approval (DD/MMM/YYYY)
CEIm Hospital Univ. 12 Octubre<br>Área de Gestión de Proyectos — Gestión de la Investigación<br>Instituto de Investigación Hospital 12 de Octubre (i+12)<br>Centro de Actividades Ambulatorias (CAA), Bloque D - Planta 6ª<br>Avda. de Cordoba s/n<br>28041 MADRID	Luis Manuel Manso Sánchez	03011 / Dr. Rafael Salguero Bodes	Protocol version 3.0 23/MAR/2012<br>Local Amendment 1 (22/AUG/2012)<br>Protocol version 4.0 22/OCT/2014	07/JUN/2012<br>25/OCT/2012<br>27/JAN/2015

SPAIN				
IRB or IEC (name/address)	IRB or IEC Chairperson	Centre number (5 digit number) / Investigator(s)	Protocol and/or Amendment number(s)	Date of Final Approval (DD/MMM/YYYY)
Comité Etico Investigación Clínica de Galicia<br>División de Farmacia y Productos Sanitarios<br>Consellería de Sanidade<br>C/San Lázaro, s/n –Edif. Administrativo<br>14703 Santiago Compostela	Beatriz Gil González	03012 / Dr.Pilar Mazon Ramos	Protocol version 3.0 23/MAR/2012<br>Local Amendment 1 (22/AUG/2012)<br>Protocol version 4.0 22/OCT/2014	07/JUN/2012<br>25/OCT/2012<br>27/JAN/2015
CEIm H. Univ. Vall d'Hebrón<br>Comité Ético de Investigación- Edificio Materno-Infantil, planta 13<br>Passeig Vall d'Hebron, 119-129<br>08035 Barcelona<br>Spain	Soledad Gallego Melcón	03013 / Dr. Angel Moya	Protocol version 3.0 23/MAR/2012<br>Local Amendment 1 (22/AUG/2012)<br>Protocol version 4.0 22/OCT/2014	07/JUN/2012<br>25/OCT/2012<br>27/JAN/2015

SPAIN				
IRB or IEC (name/address)	IRB or IEC Chairperson	Centre number (5 digit number) / Investigator(s)	Protocol and/or Amendment number(s)	Date of Final Approval (DD/MMM/YYYY)
CEIm Hospital Fundación Jiménez Díaz-FJD. Area Gestión de Investigación<br>Instituto de Investigación Sanitaria-FJD<br>Avda. Reyes Católicos, 2, Entreplanta<br>28040-Madrid	Javier Bécares Martínez	03016 / Dr. Isabel Egocheaga Cabello	Protocol version 3.0 23/MAR/2012<br>Local Amendment 1 (22/AUG/2012)<br>Protocol version 4.0 22/OCT/2014	07/JUN/2012<br>25/OCT/2012<br>27/JAN/2015
Comité Ético Investigación Clínica de Galicia<br>División de Farmacia y Productos Sanitarios<br>Consellería de Sanidade<br>C/San Lázaro, s/n –Edif. Administrativo<br>14703 Santiago Compostela	Beatriz Gil González	03018/ Dr. Carlos González Juanatey	Protocol version 3.0 23/MAR/2012<br>Local Amendment 1 (22/AUG/2012)<br>Protocol version 4.0 22/OCT/2014	07/JUN/2012<br>25/OCT/2012<br>27/JAN/2015

SPAIN				
IRB or IEC (name/address)	IRB or IEC Chairperson	Centre number (5 digit number) / Investigator(s)	Protocol and/or Amendment number(s)	Date of Final Approval (DD/MMM/YYYY)
Comité Ético Investigación Clínica de Galicia<br>División de Farmacia y Productos Sanitarios<br>Consellería de Sanidade<br>C/San Lázaro, s/n –Edif. Administrativo<br>14703 Santiago Compostela	Beatriz Gil González	03019 / Dr. Antonio Pose Reino	Protocol version 3.0 23/MAR/2012<br><br>Local Amendment 1 (22/AUG/2012)<br><br>Protocol version 4.0 22/OCT/2014	07/JUN/2012<br><br>25/OCT/2012<br><br>27/JAN/2015
CEIm H. U. de G.C. Dr. Negrín<br>At. Magüi-Administrativa.<br>Nivel -1<br>Barranco de la Ballena S/N<br>35010 - Las Palmas de Gran Canaria	Vicente Olmo Quintana	03020 / Dr. Antonio García Quintana	Protocol version 3.0 23/MAR/2012<br><br>Local Amendment 1 (22/AUG/2012)<br><br>Protocol version 4.0 22/OCT/2014	07/JUN/2012<br><br>25/OCT/2012<br><br>27/JAN/2015

SPAIN				
IRB or IEC (name/address)	IRB or IEC Chairperson	Centre number (5 digit number) / Investigator(s)	Protocol and/or Amendment number(s)	Date of Final Approval (DD/MMM/YYYY)
CEIm H. Univ. y Politécnico La Fe<br>Avda de Fernando Abril Martorell, n.106, Hospital U. i P. La Fe, Torre A, Planta 7, despacho 7.12,<br>46026 Valencia	Adela Cañete Nieto	03021 / Dr. Joaquin Oscà Asensi	Protocol version 3.0 23/MAR/2012<br><br>Local Amendment 1 (22/AUG/2012)<br><br>Protocol version 4.0 22/OCT/2014	07/JUN/2012<br><br>25/OCT/2012<br><br>27/JAN/2015
CEIm provincial de Málaga<br>Hospital Regional Universitario de Málaga<br>U.G. de Ensayos Clínicos - Pabellón A - Planta 7ª<br>Avda. Carlos Haya, s/n<br>29010 - Málaga	MANUEL E. HERRERA GUTIÉRREZ	03024 / Dr. Emilio Gonzalez Cocina	Protocol version 3.0 23/MAR/2012<br><br>Local Amendment 1 (22/AUG/2012)<br><br>Protocol version 4.0 22/OCT/2014	07/JUN/2012<br><br>25/OCT/2012<br><br>27/JAN/2015

SPAIN				
IRB or IEC (name/address)	IRB or IEC Chairperson	Centre number (5 digit number) / Investigator(s)	Protocol and/or Amendment number(s)	Date of Final Approval (DD/MMM/YYYY)
CEIm H. Univ. Ramón y Cajal<br>Ctra. de Colmenar Viejo km. 9,100<br>28034 Madrid	Sonsoles Sancho García	03026 / Dr. José María Lobos Bejarano	Protocol version 3.0 23/MAR/2012<br><br>Local Amendment 1 (22/AUG/2012)<br><br>Protocol version 4.0 22/OCT/2014	07/JUN/2012<br><br>25/OCT/2012<br><br>27/JAN/2015
CEIm de Cáceres<br>Hospital Ntra. Sra. De La Montaña<br>Secretaría CEIM –<br>Avda. de España, 2<br>10004 Cáceres	Luis Palomo Cobos	03027 /Dr. José Polo Garcéa	Protocol version 3.0 23/MAR/2012<br><br>Local Amendment 1 (22/AUG/2012)<br><br>Protocol version 4.0 22/OCT/2014	07/JUN/2012<br><br>25/OCT/2012<br><br>27/JAN/2015

SPAIN				
IRB or IEC (name/address)	IRB or IEC Chairperson	Centre number (5 digit number) / Investigator(s)	Protocol and/or Amendment number(s)	Date of Final Approval (DD/MMM/YYYY)
CEIm H. General de Alicante<br>Hospital General Universitario de Alicante<br>C/ Pintor Baeza, número 12, Planta 5ª Centro de Diagnósticos<br>03010 – Alicante	Caridad Tapia Collados	03030 / Dr. Vicente Bertomeu Gonzalez	Protocol version 3.0 23/MAR/2012<br>Local Amendment 1 (22/AUG/2012)<br>Protocol version 4.0 22/OCT/2014	07/JUN/2012<br>25/OCT/2012<br>27/JAN/2015
CEIm Provincial de Granada<br>Complejo Hospitalario Universitario de Granada<br>Avda. del Doctor Oloriz, nº 16<br>18012 - GRANADA	Fidel Fernandez Quesada	03031 / Dr. Miguel Angel Ulecia	Protocol version 3.0 23/MAR/2012<br>Local Amendment 1 (22/AUG/2012)<br>Protocol version 4.0 22/OCT/2014	07/JUN/2012<br>25/OCT/2012<br>27/JAN/2015

SPAIN				
IRB or IEC (name/address)	IRB or IEC Chairperson	Centre number (5 digit number) / Investigator(s)	Protocol and/or Amendment number(s)	Date of Final Approval (DD/MMM/YYYY)
Comité Ético Investigación Clínica de Galicia<br>División de Farmacia y Productos Sanitarios<br>Consellería de Sanidade<br>C/San Lázaro, s/n –Edif. Administrativo<br>14703 Santiago Compostela	Beatriz Gil González	03032 / Dr. Carlos Rodriguez Pascual & Dr. Ivett Guadalupe Torres	Protocol version 3.0 23/MAR/2012<br>Local Amendment 1 (22/AUG/2012)<br>Protocol version 4.0 22/OCT/2014	07/JUN/2012<br>25/OCT/2012<br>27/JAN/2015
CEIm H. Univ. Ramón y Cajal<br>Carretera de Colmenar Viejo, Km. 9,1<br>Planta -2 Dcha.<br>28034 Madrid	Sonsoles Sancho García	03033 / Prof. Jose Luis Zamorano	Protocol version 3.0 23/MAR/2012<br>Local Amendment 1 (22/AUG/2012)<br>Protocol version 4.0 22/OCT/2014	07/JUN/2012<br>25/OCT/2012<br>27/JAN/2015

SPAIN				
IRB or IEC (name/address)	IRB or IEC Chairperson	Centre number (5 digit number) / Investigator(s)	Protocol and/or Amendment number(s)	Date of Final Approval (DD/MMM/YYYY)
CEIm Hospital Universitario Doctor Peset<br>Hospital Universitario Dr. Peset<br>C/ San Lázaro s/n 1º<br>46017 VALENCIA	Pilar Codoñer Franch	03034 / Dr. Begoña Sevilla Toran	Protocol version 3.0 23/MAR/2012<br>Local Amendment 1 (22/AUG/2012)<br>Protocol version 4.0 22/OCT/2014	07/JUN/2012<br>25/OCT/2012<br>27/JAN/2015
CEIm Provincial Cádiz Hospital Puerta del Mar.<br>Ana de Viya, 21<br>11009 - CADIZ	Encarnación Benítez Rodríguez	03035 / Dr. Antonio Martin Santana	Protocol version 3.0 23/MAR/2012<br>Local Amendment 1 (22/AUG/2012)<br>Protocol version 4.0 22/OCT/2014	07/JUN/2012<br>25/OCT/2012<br>27/JAN/2015

SPAIN				
IRB or IEC (name/address)	IRB or IEC Chairperson	Centre number (5 digit number) / Investigator(s)	Protocol and/or Amendment number(s)	Date of Final Approval (DD/MMM/YYYY)
Comité de Ética de la Investigación de las Illes Balears<br>Consejería de Salud<br>Dirección: C/ Jesús, 38A<br>Palma 07010	Francisco Campoamor Landín	03036 / Dr. Tomas Ripoll Vera	Protocol version 3.0 23/MAR/2012<br><br>Local Amendment 1 (22/AUG/2012)<br><br>Protocol version 4.0 22/OCT/2014	07/JUN/2012<br><br>25/OCT/2012<br><br>27/JAN/2015
Comité Ético Investigación Clínica de Galicia<br>División de Farmacia y Productos Sanitarios<br>Consellería de Sanidade<br>C/San Lázaro, s/n –Edif. Administrativo<br>14703 Santiago Compostela	Beatriz Gil González	03037 / Dr. Juan Carlos Arias	Protocol version 3.0 23/MAR/2012<br><br>Local Amendment 1 (22/AUG/2012)<br><br>Protocol version 4.0 22/OCT/2014	07/JUN/2012<br><br>25/OCT/2012<br><br>27/JAN/2015

SPAIN				
IRB or IEC (name/address)	IRB or IEC Chairperson	Centre number (5 digit number) / Investigator(s)	Protocol and/or Amendment number(s)	Date of Final Approval (DD/MMM/YYYY)
CEIm HOSPITAL GENERAL UNIVERSITARIO DE VALENCIA<br>FUNDACIÓN INVESTIGACIÓN CHGUV<br>PABELLÓN B – 4ª PLANTA<br>Avda. Tres Cruces s/n<br>CP: 46014 Valencia	Emilio López Alcina	03038 / Dr. Lorenzo Fácila	Protocol version 3.0 23/MAR/2012<br>Local Amendment 1 (22/AUG/2012)<br>Protocol version 4.0 22/OCT/2014	07/JUN/2012<br>25/OCT/2012<br>27/JAN/2015
CEIm Area de Salud de León y el Bierzo<br>Complejo Asistencial Universitario de León<br>Altos de Nava, s/n<br>24071-León	Armando Pérez de Prado	03039 / Dr. Ignacio Iglesias Garriz	Protocol version 3.0 23/MAR/2012<br>Local Amendment 1 (22/AUG/2012)<br>Protocol version 4.0 22/OCT/2014	07/JUN/2012<br>25/OCT/2012<br>27/JAN/2015

SPAIN				
IRB or IEC (name/address)	IRB or IEC Chairperson	Centre number (5 digit number) / Investigator(s)	Protocol and/or Amendment number(s)	Date of Final Approval (DD/MMM/YYYY)
CEIm Hospital de Bellvitge<br>Antiguo módulo del Banco Santander<br>Feixa Llarga, s/n<br>08907 L'Hospitalet de Llobregat (Barcelona)	Francesc Esteve Urbano	03040 / Dr. Román Freixa Pàmias	Protocol version 3.0 23/MAR/2012<br><br>Local Amendment 1 (22/AUG/2012)<br><br>Protocol version 4.0 22/OCT/2014	07/JUN/2012<br><br>25/OCT/2012<br><br>27/JAN/2015
CEIm de Cantabria<br>Gestión Administrativa-UCICEC<br>Instituto de Investigación Marques de Valdecilla (IDIVAL)<br>Edificio IFIMAV, 3ª Planta<br>Avda. Cardenal Herrera Oria, s/n<br>39011- Santander	Fernando Rodríguez Fernández	03041/ Dr. Juan José Olalla Antolín	Protocol version 3.0 23/MAR/2012<br><br>Local Amendment 1 (22/AUG/2012)<br><br>Protocol version 4.0 22/OCT/2014	07/JUN/2012<br><br>25/OCT/2012<br><br>27/JAN/2015

SPAIN				
IRB or IEC (name/address)	IRB or IEC Chairperson	Centre number (5 digit number) / Investigator(s)	Protocol and/or Amendment number(s)	Date of Final Approval (DD/MMM/YYYY)
CEIm H. de la Santa Creu i Sant Pau<br>Hospital Santa Creu i Sant Pau<br>Institut de Recerca<br>C/ Sant Quintí, 77-79 08041 Barcelona	Antonio López Pousa	03042 / Dr. Amparo Mena	Protocol version 3.0 23/MAR/2012<br><br>Local Amendment 1 (22/AUG/2012)<br><br>Protocol version 4.0 22/OCT/2014	07/JUN/2012<br><br>25/OCT/2012<br><br>27/JAN/2015
CEIm HOSPITAL SEVERO OCHOA<br>Hospital Severo Ochoa<br>Secretaría CEIm. 1ª Planta Zona C.<br>Avda. de Orellana, s/n<br>28911 LEGANÉS (MADRID)	Amparo Lucena Campillo	03043 / Dr. Ángel Grande Ruiz	Protocol version 3.0 23/MAR/2012<br><br>Local Amendment 1 (22/AUG/2012)<br><br>Protocol version 4.0 22/OCT/2014	07/JUN/2012<br><br>25/OCT/2012<br><br>27/JAN/2015

SPAIN				
IRB or IEC (name/address)	IRB or IEC Chairperson	Centre number (5 digit number) / Investigator(s)	Protocol and/or Amendment number(s)	Date of Final Approval (DD/MMM/YYYY)
CEIm Comunidad Foral de Navarra<br>Secretaria del Comité Ético de Investigación Clínica de Navarra (CEIM)<br>Pabellón de Docencia<br>Recinto Hospital de Navarra<br>Irunlarrea, 3<br>31008 Pamplona (Navarra)	Jesús Mª Arteaga Coloma	03045 / Dra. Irene Madariaga	Protocol version 3.0 23/MAR/2012<br><br>Local Amendment 1 (22/AUG/2012)<br><br>Protocol version 4.0 22/OCT/2014	07/JUN/2012<br><br>25/OCT/2012<br><br>27/JAN/2015
CEIm Provincial de Sevilla<br>Hospital Universitario Virgen Macarena Avda. Dr. Fedriani, 3 – 41009 Sevilla, Unidad de Investigación – 2ª Planta	Víctor Sánchez Margalet	03046 / Dr. Mariano Ruiz Borrell	Protocol version 3.0 23/MAR/2012<br><br>Local Amendment 1 (22/AUG/2012)<br><br>Protocol version 4.0 22/OCT/2014	07/JUN/2012<br><br>25/OCT/2012<br><br>27/JAN/2015

SPAIN				
IRB or IEC (name/address)	IRB or IEC Chairperson	Centre number (5 digit number) / Investigator(s)	Protocol and/or Amendment number(s)	Date of Final Approval (DD/MMM/YYYY)
CEIm Hospital Fundación Jiménez Díaz-FJD. Area Gestión de Investigación<br>Instituto de Investigación Sanitaria-FJD<br>Avda. Reyes Católicos, 2, Entreplanta<br>28040-Madrid	Javier Bécares Martínez	03047 / Dr. Juan Benezet Mazuecos	Protocol version 3.0 23/MAR/2012<br><br>Local Amendment 1 (22/AUG/2012)<br><br>Protocol version 4.0 22/OCT/2014	07/JUN/2012<br><br>25/OCT/2012<br><br>27/JAN/2015
CEIm provincial de Málaga<br>Hospital Regional Universitario de Málaga<br>U.G. de Ensayos Clínicos - Pabellón A - Planta 7ª<br>Avda. Carlos Haya, s/n<br>29010 - Málaga	MANUEL E. HERRERA GUTIÉRREZ	03048 / Dr. Manuel de Mora Martín	Protocol version 3.0 23/MAR/2012<br><br>Local Amendment 1 (22/AUG/2012)<br><br>Protocol version 4.0 22/OCT/2014	07/JUN/2012<br><br>25/OCT/2012<br><br>27/JAN/2015

SPAIN				
IRB or IEC (name/address)	IRB or IEC Chairperson	Centre number (5 digit number) / Investigator(s)	Protocol and/or Amendment number(s)	Date of Final Approval (DD/MMM/YYYY)
CEIm-Parc de Salut MAR<br>IMIM - Institut de Recerca Hospital del Mar<br>Parc de Recerca Biomèdica de Barcelona<br>C/ Doctor Aiguader, 88<br>08003 Barcelona	Marta Guix Arnau	03049 / Dr. Jorge Bruguera Cortada	Protocol version 3.0 23/MAR/2012<br><br>Local Amendment 1 (22/AUG/2012)<br><br>Protocol version 4.0 22/OCT/2014	07/JUN/2012<br><br>25/OCT/2012<br><br>27/JAN/2015
CEIm Area de Salud de León y el Bierzo<br>Complejo Asistencial Universitario de León<br>Altos de Nava, s/n<br>24071-León	Armando Pérez de Prado	03050 / Dr. David Alonso	Protocol version 3.0 23/MAR/2012<br><br>Local Amendment 1 (22/AUG/2012)<br><br>Protocol version 4.0 22/OCT/2014	07/JUN/2012<br><br>25/OCT/2012<br><br>27/JAN/2015

SPAIN				
IRB or IEC (name/address)	IRB or IEC Chairperson	Centre number (5 digit number) / Investigator(s)	Protocol and/or Amendment number(s)	Date of Final Approval (DD/MMM/YYYY)
CEIm provincial de Málaga<br>Hospital Regional Universitario de Málaga<br>U.G. de Ensayos Clínicos - Pabellón A - Planta 7ª<br>Avda. Carlos Haya, s/n<br>29010 - Málaga	MANUEL E. HERRERA GUTIÉRREZ	03051 / Dr. Alejandro Pérez-Cabezas	Protocol version 3.0 23/MAR/2012<br><br>Local Amendment 1 (22/AUG/2012)<br><br>Protocol version 4.0 22/OCT/2014	07/JUN/2012<br><br>25/OCT/2012<br><br>27/JAN/2015
CEIm Hospital Universitario La Princesa<br>C/ Diego de León 62, 1ª planta<br>28006.- Madrid	Francisco Abad Santos	03052 / Dr. Fernando Alfonso Manterola	Protocol version 3.0 23/MAR/2012<br><br>Local Amendment 1 (22/AUG/2012)<br><br>Protocol version 4.0 22/OCT/2014	07/JUN/2012<br><br>25/OCT/2012<br><br>27/JAN/2015

SPAIN				
IRB or IEC (name/address)	IRB or IEC Chairperson	Centre number (5 digit number) / Investigator(s)	Protocol and/or Amendment number(s)	Date of Final Approval (DD/MMM/YYYY)
CEIm HOSPITAL GENERAL UNIVERSITARIO DE VALENCIA<br>FUNDACIÓN INVESTIGACIÓN CHGUV<br>PABELLÓN B – 4ª PLANTA<br>Avda. Tres Cruces s/n<br>CP: 46014 Valencia	Emilio López Alcina	03053 / Dr. Vicente Montagud	Protocol version 3.0 23/MAR/2012<br><br>Local Amendment 1 (22/AUG/2012)<br><br>Protocol version 4.0 22/OCT/2014	07/JUN/2012<br><br>25/OCT/2012<br><br>27/JAN/2015
CEIm Hospital Universitario Virgen de la Arrixaca.<br>Ctra Madrid-Cartagena s/n<br>30120 Murcia	Antonio Piñero Madrona	03056 / Dr. Ignacio Gil	Protocol version 3.0 23/MAR/2012<br><br>Local Amendment 1 (22/AUG/2012)<br><br>Protocol version 4.0 22/OCT/2014	07/JUN/2012<br><br>25/OCT/2012<br><br>27/JAN/2015

SPAIN				
IRB or IEC (name/address)	IRB or IEC Chairperson	Centre number (5 digit number) / Investigator(s)	Protocol and/or Amendment number(s)	Date of Final Approval (DD/MMM/YYYY)
CEIm Hospital Universitario Virgen de la Arrixaca.<br>Ctra Madrid-Cartagena s/n<br>30120 Murcia	Antonio Piñero Madrona	03057 / Dr. Beatriz Pérez	Protocol version 3.0 23/MAR/2012<br><br>Local Amendment 1 (22/AUG/2012)<br><br>Protocol version 4.0 22/OCT/2014	07/JUN/2012<br><br>25/OCT/2012<br><br>27/JAN/2015
CEIm DE CORDOBA<br>Hospital Universitario "Reina Sofía"<br>Ed Consultas Externas Pl -1<br>Avda Menéndez Pidal, s/n (14004 Córdoba)	Inmaculada Herrera Arroyo	03058 / Dr. Amador López	Protocol version 3.0 23/MAR/2012<br><br>Local Amendment 1 (22/AUG/2012)<br><br>Protocol version 4.0 22/OCT/2014	07/JUN/2012<br><br>25/OCT/2012<br><br>27/JAN/2015

SPAIN				
IRB or IEC (name/address)	IRB or IEC Chairperson	Centre number (5 digit number) / Investigator(s)	Protocol and/or Amendment number(s)	Date of Final Approval (DD/MMM/YYYY)
CEIm provincial de Sevilla<br>Hospital Universitario Virgen Macarena Avda. Dr. Fedriani, 3 – 41009 Sevilla, Unidad de Investigación – 2ª Planta	Victor Sánchez Margalef	03059 / Dr. Silvia Gómez	Protocol version 3.0 23/MAR/2012<br>Local Amendment 1 (22/AUG/2012)<br>Protocol version 4.0 22/OCT/2014	07/JUN/2012<br>25/OCT/2012<br>27/JAN/2015
CEIm provincial de Sevilla<br>Hospital Universitario Virgen Macarena<br>Unidad de Investigación – 2ª Planta<br>Avda. Dr. Fedriani, 3 – 41009 Sevilla,	Víctor Sánchez Margalef	03060 / Dr. Ricardo Pavón	Protocol version 3.0 23/MAR/2012<br>Local Amendment 1 (22/AUG/2012)<br>Protocol version 4.0 22/OCT/2014	07/JUN/2012<br>25/OCT/2012<br>27/JAN/2015

SPAIN				
IRB or IEC (name/address)	IRB or IEC Chairperson	Centre number (5 digit number) / Investigator(s)	Protocol and/or Amendment number(s)	Date of Final Approval (DD/MMM/YYYY)
CEIm Hospital Arnau de Vilanova.<br>C/ San Clemente, 12, 8ª Planta<br>Valencia 46015	Fernando Sánchez Toril López	03061 / Dr. Juan Cosín	Protocol version 3.0 23/MAR/2012<br><br>Local Amendment 1 (22/AUG/2012)<br><br>Protocol version 4.0 22/OCT/2014	07/JUN/2012<br><br>25/OCT/2012<br><br>27/JAN/2015
Comité Etico Investigación Clínica de Galicia<br>División de Farmacia y Productos Sanitarios<br>Consellería de Sanidade<br>C/San Lázaro, s/n –Edif. Administrativo<br>14703 Santiago Compostela	Beatriz Gil González	03062 / Dr. Ricardo Nicolás	Protocol version 3.0 23/MAR/2012<br><br>Local Amendment 1 (22/AUG/2012)<br><br>Protocol version 4.0 22/OCT/2014	07/JUN/2012<br><br>25/OCT/2012<br><br>27/JAN/2015

SWEDEN				
IRB or IEC (name/address)	IRB or IEC Chairperson	Centre number (5 digit number) / Investigator(s)	Protocol and/or Amendment number(s)	Date of Final Approval (DD/MMM/YYYY)
Regionala Etikprövningsnämnden i Lund<br>Box 133<br>221 00 Lund	NA	19001 – Dr Peter Svensson<br>19002 – Dr Christos Milonas<br>19006 – Dr Magnus Forsgren<br>19009 – Dr Bengt Johansson<br>19011 – Dr Valerie Bockisch<br>19014 – Dr Stellan Bandh<br>19015 – Dr Mika Skeppholm	Protocol version 3.0 23/MAR?2012<br>Protocol version 4.0 22/OCT/2014	07/NOV/2012<br>14/JAN/2015

Switzerland				
IRB or IEC (name/address)	IRB or IEC Chairperson	Centre number (5 digit number) / Investigator(s)	Protocol and/or Amendment number(s)	Date of Final Approval (DD/MMM/YYYY)
Ethics Committee of both Cantons of Basel EKKB (Ethikkommission beider Basel EKKBB)<br>Hebelstr. 53<br>CH-4056 Basel, Switzerland	Prof. Dr. med. Andre P. Perruchoud	44001 / Prof. Dr. med. Philippe Lyrer	Protocol version 4.0 7/JUN/2013	EC: 01/JUL/2013<br>(CA: 19/AUG/2013)
Ethics Committee of both Cantons of Basel EKKB (Ethikkommission beider Basel EKKBB)<br>Hebelstr. 53<br>CH-4056 Basel, Switzerland	Prof. Dr. med. Andre. P. Perruchoud	44001 /Prof. Dr. med. Philippe Lyrer	Global Amendment 1<br>Protocol 2.0 23 Jul 2013	09/NOV/2013
Ethics Committee of Northwestern and Central Switzerland (Ethikkommission Nordwest- und Zentralschweiz)<br>Hebelstr. 53<br>CH-4056 Basel, Switzerland	Prof. Dr. med. Andre. P. Perruchoud	44001 Prof. / Dr. med. Philippe Lyrer<br>44002 / Prof. Dr. med. Tiziano Moccetti<br>44004 / Prof. Dr. med. Hans Rickli<br>44005 / Prof. Dr. med. Beat Meyer<br>44008 / Dr. med. Patrik Michel	Local Protocol Amendment 1<br>dated 28 May 2015	30/MAR/2016

Taiwan				
IRB or IEC (name/address)	IRB or IEC Chairperson	Centre number (5 digit number) / Investigator(s)	Protocol and/or Amendment number(s)	Date of Final Approval (DD/MMM/YYYY)
Institutional Review Board, Taipei Veterans General Hospital/ 201 Shih-Pai Road, Sec. 2, Taipei, Taiwan, 11217 Republic of China	Shung-Tai Ho	46001/ Shih-Ann Chen	Protocol version 3.0 23/MAR/2012<br><br>Protocol version 4.0 7/JUN/2013	19/JUL/2013<br><br>02/OCT/2013
Cheng Hsin General Hospital Institutional Review Board/ No.45,Cheng Hsin St.,Pai-Tou,Taipei, Taiwan, 11220	Shen-Kou Tsai	46002/ Wei-Hsian Yin	Protocol version 3.0 23/MAR/2012<br><br>Protocol version 4.0 7/JUN/2013	04/MAR/2013<br><br>13/SEP/2013
Chang Chung Medical Foundation Institutional Review Board/ 199 TUNG HWA NORTH ROAD, TAIPEI, TAIWAN, 10507	Tsang-Tang Hsieh	46003/ Ming-Shien Wen	Protocol version 3.0 23/MAR/2012<br><br>Protocol version 4.0 7/JUN/2013	24/MAY/2013<br><br>29/APR/2014
Institutional Review Board, MACHAY	Yen-Ta Lu	46004/ Jen-Yuan Kuo	Protocol version 3.0	24/JUN/2013

Taiwan				
IRB or IEC (name/address)	IRB or IEC Chairperson	Centre number (5 digit number) / Investigator(s)	Protocol and/or Amendment number(s)	Date of Final Approval (DD/MMM/YYYY)
MEMORIAL HOSPITAL/92, Sec.2, Chungshan N. Rd., Taipei 10449, Taiwan			23/MAR/2012<br><br>Protocol version 4.0 7/JUN/2013	 <br>30/SEP/2013
Institutional Review Board of the Cathay General Hospital/ No. 280, Sec. 4, Ren-Ai Rd., Taipei, Taiwan 10630 R.O.C	Cheng-Hua Huang	46005/ Chi-Hung Huang	Protocol version 3.0 23/MAR/2012<br><br>Protocol version 4.0 7/JUN/2013	10/JUL/2013<br><br>02/OCT/2013
Research Ethics Committee C National Taiwan University Hospital/ 7, Chung-Shan South Road, Taipei, Taiwan 100, R.O.C.	Hong-Nerng Ho	46006/ Fu-Tien Chiang<br>Lian-Yu Lin	Protocol version 3.0 23/MAR/2012<br><br>Protocol version 4.0 7/JUN/2013	21/JUN/2013<br><br>30/AUG/2013
Institutional Review Board Kaohsiung Medical University, Chung-Ho Memorial Hospital / No, 100, Tzyou 1st Road, Kaohsiung 807, Taiwan	Zu-Yau Lin<br>Li-Tzong Chen	46007/ Wen-Ter Lai	Protocol version 3.0 23/MAR/2012<br><br>Protocol version 4.0 7/JUN/2013	22/AUG/2013<br><br>26/NOV/2013

Taiwan				
IRB or IEC (name/address)	IRB or IEC Chairperson	Centre number (5 digit number) / Investigator(s)	Protocol and/or Amendment number(s)	Date of Final Approval (DD/MMM/YYYY)
Institutional Review Board<br>National Cheng Kung University Hospital<br>/138 Sheng-Li Rd, Tainan 704., Taiwan R.O.C.	Thy-Sheng Lin	46008/Jyh-Hong Chen<br>Chih-Chan Lin	Protocol version 3.0 23/MAR/2012<br><br>Protocol version 4.0 7/JUN/2013	15/AUG/2013<br><br>08/NOV/2013
Chang Chung Medical Foundation Institutional Review Board/<br>199 TUNG HWA NORTH ROAD, TAIPEI, TAIWAN, 10507	Tsang-Tang Hsieh	46009/ Kuo-Ho Yeh	Protocol version 3.0 23/MAR/2012<br><br>Protocol version 4.0 7/JUN/2013	20/AUG/2013<br><br>29/APR/2014
The institutional Review Board of Taichung Veterans General Hospital/	Jeng-Yuan Hsu	46010/Kuo-Yang Wang	Protocol version 4.0 7/JUN/2013	02/SEP/2013

UAE				
IRB or IEC (name/address)	IRB or IEC Chairperson	Centre number (5 digit number) / Investigator(s)	Protocol and/or Amendment number(s)	Date of Final Approval (DD/MMM/YYYY)
Dubai Scientific Research Ethics Committee,<br>Dubai Health Authority,<br>Dubai Hospital AL-BRAHA Area,<br>Al Khaleej Road,<br>Dubai,<br>7272,<br>United Arab. Emirates	Dr Suhail Al Rukun	31001/N Bazargani	Protocol Version 3 dated 19 July 2011<br><br>Protocol Version 4 dated 7 June 2013 (amendment 1)	02/Jun/2013<br><br>27/Apr/2014
Al Qassimi Clinical Research Centre<br>Research Ethics Committee<br>Sharjah,<br>United Arab. Emirates	Dr Ghada AlTajer	31002/G Kerfes	Protocol Version 4 dated 7 June 2013 (amendment 1)	13/Nov/2013
Zayed Military Hospital IRB,<br>Abu Dhabi,<br>United Arab. Emirates	Dr Asma AlNuaimi	31004/S Alkaabi<br>(EC approval received and this site was initiated but no patients were recruited at this site)	Protocol Version 4 dated 7 June 2013 (amendment 1)	05/May/2014
Research Committee	Dr. Jaishen Rajah	31005/O Elhag	Protocol Version 4 dated 7	28/May/2014

UAE				
IRB or IEC (name/address)	IRB or IEC Chairperson	Centre number (5 digit number) / Investigator(s)	Protocol and/or Amendment number(s)	Date of Final Approval (DD/MMM/YYYY)
Sheikh Khalifa Medical City<br>Al Manhal Street with Al Karamah Street, Bateen Street, Al Manhal,<br>Abu Dhabi,<br>51900,<br>United Arab. Emirates			June 2013 (amendment 1)	
Al Ain Hospital ethic committee,<br>Shakboot Bin Sultan Street,<br>Abu Dhabi,<br>1006,<br>United Arab. Emirates	Gaylord Abad	31006/H Sabbour	Protocol Version 4 dated 7 June 2013 (amendment 1)	29/Dec/2013

United Kingdom				
IRB or IEC (name/address)	IRB or IEC Chairperson	Centre number (5 digit number) / Investigator(s)	Protocol and/or Amendment number(s)	Date of Final Approval (DD/MMM/YYYY)
NRES Committee South Central - Berkshire B<br>Bristol REC Centre<br>Whitefriars<br>Level 3, Block B<br>Lewins Mead<br>Bristol<br>BS1 2NT	Dr John Sheridan	All UK sites<br>20001/Dr. Derek Connolly<br>20003/Dr. Piers Clifford<br>20006/Dr. Andrew Moriarty<br>20008/Dr. Maurice Pye<br>20009/Dr. Manav Sohal<br>20011/Dr. Amrit Takhar<br>20012/Dr. Wayne Turner<br>20013/Dr. Janice Patrick<br>20015/Dr. Shahid Aziz<br>20017/Dr. Terry McCormack<br>20019/Dr. Kausik Chatterjee<br>20020/Dr. Craig Barr<br>20021/Dr. Zakir Mohiuddin<br>20022/Dr. Nick Jones<br>20024/Dr. Hugh McIntyre	Final Protocol<br>Global Amendment 1	07/MAY/2013<br>25/FEB/2015

United Kingdom				
IRB or IEC (name/address)	IRB or IEC Chairperson	Centre number (5 digit number) / Investigator(s)	Protocol and/or Amendment number(s)	Date of Final Approval (DD/MMM/YYYY)
		20028/Dr. Muzahir Taybejee<br>20029/Dr. Harinath Chandrashekar<br>20030/Dr. Lakshmanan Sekaran<br>20031/Dr. Richard Bala<br>20035/Dr. Basel Hanbali<br>20036/Dr. Sasalu Deepak<br>20038/Dr. Frances Adams<br>20040/Dr. Susana Cary<br>20044/Dr. Matthew Hoghton<br>20045/Dr. Muhammad Khalid<br>20047/Dr. Oran Corey<br>20049/Professor Diana Gorog<br>20050/Dr. Ali Ghanbasha<br>20051/Dr. Thuraia Nageh<br>20052/Dr. Drew Bradman		

United Kingdom				
IRB or IEC (name/address)	IRB or IEC Chairperson	Centre number (5 digit number) / Investigator(s)	Protocol and/or Amendment number(s)	Date of Final Approval (DD/MMM/YYYY)
		20053/Mr. Dzifa Bradman<br>20054/Dr. Ewart Jackson-Voyzey<br>20056/Dr. Brian Gordon<br>20061/Dr. Abul Azim<br>20063/Dr. Gershan Davis<br>20064/Dr. Priya Ganeshkumar<br>20068/Dr. Mark Dayer<br>20072/Dr. Paul Ainsworth<br>20073/Dr. Phil Keeling<br>20074/Dr. Rajesh Aggarwal<br>20076/Dr. Shahid Mahmood<br>20077/Dr. Simon Dubrey<br>20081/Dr. Dipankar Dutta<br>20082/Dr. Clare Seamark<br>20083/Dr. Louise Shaw<br>20084/Dr. Hamdi Sati		

United Kingdom				
IRB or IEC (name/address)	IRB or IEC Chairperson	Centre number (5 digit number) / Investigator(s)	Protocol and/or Amendment number(s)	Date of Final Approval (DD/MMM/YYYY)
		20085/Dr. Mohamed Al-Obaidi<br>20088/Dr. David Sharman<br>20089/Dr. Adam Ellery<br>20090/Dr. Martin James<br>20091/Dr. Somnath Kumar<br>20094/Dr. Kneale Metcalf<br>20095/Dr. Abdul Nasser<br>20096/Dr. Richard Oliver<br>20099/Dr. Asok Venkataraman<br>20102/Dr. Tamara Everington<br>20105/Dr. Tim Edwards<br>20107/Dr. Indira Natarajan<br>20108/Dr. Azlisham Mohd Nor<br>20109/Dr. Margaret Ikpoh<br>20110/Dr. Sanjiv Petkar		

United Kingdom				
IRB or IEC (name/address)	IRB or IEC Chairperson	Centre number (5 digit number) / Investigator(s)	Protocol and/or Amendment number(s)	Date of Final Approval (DD/MMM/YYYY)
		20111/Dr. Madjudusan Varma<br>20112/Dr. Priya Nair<br>20113/Dr. David Hargroves<br>20114/Dr. Farazan Kamali<br>20119/Dr. Mehool Patel<br>20121/Dr. Dinesh Mistry<br>20122/Dr. Clare Holmes<br>20123/Dr. Christopher Boos<br>20125/Dr. Andrew Field<br>20127/Dr. Ruth Davies<br>20128/Dr. Neeraj Prasad<br>20130/Dr. Elizabeth Best<br>20132/Dr. Andre Van Wyk<br>20133/Dr. Toby Black<br>20134/Dr. Martin Cooper		

**United States of America**				
IRB or IEC (name/address)	IRB or IEC Chairperson	Centre number (5 digit number) / Investigator(s)	Protocol and/or Amendment number(s)	Date of Final Approval (DD/MMM/YYYY)
Schulman Associates Institutional Review Board, Inc.<br>4445 Lake Forest Drive Suite 300<br>Cincinnati, Ohio 45242<br><br>ADVARRA<br>6940 Columbia Gateway Drive,<br>Suite 110<br>Columbia, MD 21046	Sharon Lynn Nelson, MSN, RN, CNS<br><br>Sara Harnish, J.D	32001 / Graham, Brett	Protocol Version 3.0 dated 19/JUL/2011 and Local Amendment dated 17/FEB/2012<br><br>Protocol Version 4.0 dated 07/JUN/2013 (incorporating Global Amendment 1 dated 21/MAY/2013)	09/MAR/2012<br><br>03/JUL/2013
Schulman Associates Institutional Review Board, Inc.<br>4445 Lake Forest Drive Suite 300<br>Cincinnati, Ohio 45242	Sharon Lynn Nelson, MSN, RN, CNS	32004 / Iyer, Venkat	Protocol Version 3.0 dated 19/JUL/2011 and Local Amendment dated 17/FEB/2012<br><br>Protocol Version 4.0 dated	28/MAR/2012

United States of America				
IRB or IEC (name/address)	IRB or IEC Chairperson	Centre number (5 digit number) / Investigator(s)	Protocol and/or Amendment number(s)	Date of Final Approval (DD/MMM/YYYY)
ADVARRA<br>6940 Columbia Gateway Drive,<br>Suite 110<br>Columbia, MD 21046	Sara Harnish, J.D		07/JUN/2013 (incorporating Global Amendment 1 dated 21/MAY/2013)	03/JUL/2013
Schulman Associates Institutional Review Board, Inc.<br>4445 Lake Forest Drive Suite 300<br>Cincinnati, Ohio 45242<br><br>ADVARRA<br>6940 Columbia Gateway Drive,<br>Suite 110<br>Columbia, MD 21046	Sharon Lynn Nelson, MSN, RN, CNS<br><br>Sara Harnish, J.D	32005 / Lo, Eric	Protocol Version 3.0 dated 19/JUL/2011 and Local Amendment dated 17/FEB/2012<br><br>Protocol Version 4.0 dated 07/JUN/2013 (incorporating Global Amendment 1 dated 21/MAY/2013)	14/MAR/2012<br><br>03/JUL/2013
Schulman Associates Institutional Review Board, Inc.	Sharon Lynn Nelson, MSN, RN, CNS	32006 / El Shahawy, Mafouz	Protocol Version 3.0 dated 19/JUL/2011 and Local Amendment dated	21/MAR/2012

United States of America				
IRB or IEC (name/address)	IRB or IEC Chairperson	Centre number (5 digit number) / Investigator(s)	Protocol and/or Amendment number(s)	Date of Final Approval (DD/MMM/YYYY)
4445 Lake Forest Drive Suite 300<br>Cincinnati, Ohio 45242<br><br>ADVARRA<br>6940 Columbia Gateway Drive,<br>Suite 110<br>Columbia, MD 21046	Sara Harnish, J.D		17/FEB/2012<br><br>Protocol Version 4.0 dated 07/JUN/2013 (incorporating Global Amendment 1 dated 21/MAY/2013)	03/JUL/2013
Schulman Associates Institutional Review Board, Inc.<br>4445 Lake Forest Drive Suite 300<br>Cincinnati, Ohio 45242<br><br>ADVARRA<br>6940 Columbia Gateway Drive,<br>Suite 110	Sharon Lynn Nelson, MSN, RN, CNS<br><br>Sara Harnish, J.D	32007 / Blumberg, Edwin	Protocol Version 3.0 dated 19/JUL/2011 and Local Amendment dated 17/FEB/2012<br><br>Protocol Version 4.0 dated 07/JUN/2013 (incorporating Global Amendment 1 dated 21/MAY/2013)	21/MAR/2012<br><br>03/JUL/2013

United States of America				
IRB or IEC (name/address)	IRB or IEC Chairperson	Centre number (5 digit number) / Investigator(s)	Protocol and/or Amendment number(s)	Date of Final Approval (DD/MMM/YYYY)
Columbia, MD 21046				
Schulman Associates Institutional Review Board, Inc.<br>4445 Lake Forest Drive Suite 300<br>Cincinnati, Ohio 45242<br><br>ADVARRA<br>6940 Columbia Gateway Drive,<br>Suite 110<br>Columbia, MD 21046	Sharon Lynn Nelson, MSN, RN, CNS<br><br>Sara Harnish, J.D	32009 / Greer, Steven G.	Protocol Version 3.0 dated 19/JUL/2011 and Local Amendment dated 17/FEB/2012<br><br>Protocol Version 4.0 dated 07/JUN/2013 (incorporating Global Amendment 1 dated 21/MAY/2013)	28/MAR/2012<br><br>03/JUL/2013
Schulman Associates Institutional Review Board, Inc.<br>4445 Lake Forest Drive Suite 300<br>Cincinnati, Ohio 45242	Sharon Lynn Nelson, MSN, RN, CNS	32014 / Hargrove, Joe	Protocol Version 3.0 dated 19/JUL/2011 and Local Amendment dated 17/FEB/2012	02/APR/2012

United States of America				
IRB or IEC (name/address)	IRB or IEC Chairperson	Centre number (5 digit number) / Investigator(s)	Protocol and/or Amendment number(s)	Date of Final Approval (DD/MMM/YYYY)
ADVARRA<br>6940 Columbia Gateway Drive,<br>Suite 110<br>Columbia, MD 21046	Sara Harnish, J.D		Protocol Version 4.0 dated 07/JUN/2013 (incorporating Global Amendment 1 dated 21/MAY/2013)	03/JUL/2013
Schulman Associates Institutional Review Board, Inc.<br>4445 Lake Forest Drive Suite 300<br>Cincinnati, Ohio 45242<br><br>ADVARRA<br>6940 Columbia Gateway Drive,<br>Suite 110<br>Columbia, MD 21046	Sharon Lynn Nelson, MSN, RN, CNS<br><br>Sara Harnish, J.D	32016 / Roberts, Douglas	Protocol Version 3.0 dated 19/JUL/2011 and Local Amendment dated 17/FEB/2012<br><br>Protocol Version 4.0 dated 07/JUN/2013 (incorporating Global Amendment 1 dated 21/MAY/2013)	04/APR/2012<br><br>03/JUL/2013
Schulman Associates Institutional Review Board, Inc.	Sharon Lynn Nelson, MSN, RN, CNS	32017 / First, Brian	Protocol Version 3.0 dated 19/JUL/2011 and Local Amendment dated	30/MAR/2012

United States of America				
IRB or IEC (name/address)	IRB or IEC Chairperson	Centre number (5 digit number) / Investigator(s)	Protocol and/or Amendment number(s)	Date of Final Approval (DD/MMM/YYYY)
4445 Lake Forest Drive Suite 300<br>Cincinnati, Ohio 45242			17/FEB/2012	
ADVARRA<br>6940 Columbia Gateway Drive,<br>Suite 110<br>Columbia, MD 21046	Sara Harnish, J.D		Protocol Version 4.0 dated 07/JUN/2013 (incorporating Global Amendment 1 dated 21/MAY/2013)	03/JUL/2013
Schulman Associates Institutional Review Board, Inc.<br>4445 Lake Forest Drive Suite 300<br>Cincinnati, Ohio 45242	Sharon Lynn Nelson, MSN, RN, CNS	32019 / Khaira, Ajit	Protocol Version 3.0 dated 19/JUL/2011 and Local Amendment dated 17/FEB/2012	18/APR/2012
ADVARRA<br>6940 Columbia Gateway Drive,<br>Suite 110	Sara Harnish, J.D		Protocol Version 4.0 dated 07/JUN/2013 (incorporating Global Amendment 1 dated 21/MAY/2013)	03/JUL/2013

United States of America				
IRB or IEC (name/address)	IRB or IEC Chairperson	Centre number (5 digit number) / Investigator(s)	Protocol and/or Amendment number(s)	Date of Final Approval (DD/MMM/YYYY)
Columbia, MD 21046				
Schulman Associates Institutional Review Board, Inc.<br>4445 Lake Forest Drive Suite 300<br>Cincinnati, Ohio 45242<br><br>ADVARRA<br>6940 Columbia Gateway Drive,<br>Suite 110<br>Columbia, MD 21046	Sharon Lynn Nelson, MSN, RN, CNS<br><br>Sara Harnish, J.D	32020 / Neiman, James	Protocol Version 3.0 dated 19/JUL/2011 and Local Amendment dated 17/FEB/2012<br><br>Protocol Version 4.0 dated 07/JUN/2013 (incorporating Global Amendment 1 dated 21/MAY/2013)	17/APR/2012<br><br>03/JUL/2013
Schulman Associates Institutional Review Board, Inc.<br>4445 Lake Forest Drive Suite 300<br>Cincinnati, Ohio 45242	Sharon Lynn Nelson, MSN, RN, CNS	32021 / Rosenbaum, David	Protocol Version 3.0 dated 19/JUL/2011 and Local Amendment dated 17/FEB/2012<br><br>Protocol Version 4.0 dated	06/MAR/2012

United States of America				
IRB or IEC (name/address)	IRB or IEC Chairperson	Centre number (5 digit number) / Investigator(s)	Protocol and/or Amendment number(s)	Date of Final Approval (DD/MMM/YYYY)
ADVARRA<br>6940 Columbia Gateway Drive,<br>Suite 110<br>Columbia, MD 21046	Sara Harnish, J.D		07/JUN/2013 (incorporating Global Amendment 1 dated 21/MAY/2013)	03/JUL/2013
Schulman Associates Institutional Review Board, Inc.<br>4445 Lake Forest Drive Suite 300<br>Cincinnati, Ohio 45242<br><br>ADVARRA<br>6940 Columbia Gateway Drive,<br>Suite 110<br>Columbia, MD 21046	Sharon Lynn Nelson, MSN, RN, CNS<br><br>Sara Harnish, J.D	32025 / Challappa, Krishnan	Protocol Version 3.0 dated 19/JUL/2011 and Local Amendment dated 17/FEB/2012<br><br>Protocol Version 4.0 dated 07/JUN/2013 (incorporating Global Amendment 1 dated 21/MAY/2013)	25/APR/2012<br><br>03/JUL/2013
Schulman Associates Institutional Review Board, Inc.	Sharon Lynn Nelson, MSN, RN, CNS	32029 / Bloom, Stephen A.	Protocol Version 3.0 dated 19/JUL/2011 and Local Amendment dated	30/MAR/2012

United States of America				
IRB or IEC (name/address)	IRB or IEC Chairperson	Centre number (5 digit number) / Investigator(s)	Protocol and/or Amendment number(s)	Date of Final Approval (DD/MMM/YYYY)
4445 Lake Forest Drive Suite 300<br>Cincinnati, Ohio 45242<br><br>ADVARRA<br>6940 Columbia Gateway Drive,<br>Suite 110<br>Columbia, MD 21046	 <br><br>Sara Harnish, J.D		17/FEB/2012<br><br>Protocol Version 4.0 dated 07/JUN/2013 (incorporating Global Amendment 1 dated 21/MAY/2013)	 <br><br>03/JUL/2013
Schulman Associates Institutional Review Board, Inc.<br>4445 Lake Forest Drive Suite 300<br>Cincinnati, Ohio 45242<br><br>ADVARRA<br>6940 Columbia Gateway Drive,<br>Suite 110	Sharon Lynn Nelson, MSN, RN, CNS<br><br><br><br>Sara Harnish, J.D	32031 / Cohen, Andrew I.	Protocol Version 3.0 dated 19/JUL/2011 and Local Amendment dated 17/FEB/2012<br><br>Protocol Version 4.0 dated 07/JUN/2013 (incorporating Global Amendment 1 dated 21/MAY/2013)	08/MAR/2012<br><br><br>03/JUL/2013

United States of America				
IRB or IEC (name/address)	IRB or IEC Chairperson	Centre number (5 digit number) / Investigator(s)	Protocol and/or Amendment number(s)	Date of Final Approval (DD/MMM/YYYY)
Columbia, MD 21046				
Schulman Associates Institutional Review Board, Inc.<br>4445 Lake Forest Drive Suite 300<br>Cincinnati, Ohio 45242<br><br>ADVARRA<br>6940 Columbia Gateway Drive,<br>Suite 110<br>Columbia, MD 21046	Sharon Lynn Nelson, MSN, RN, CNS<br><br>Sara Harnish, J.D	32035 / Quigley, James	Protocol Version 3.0 dated 19/JUL/2011 and Local Amendment dated 17/FEB/2012<br><br>Protocol Version 4.0 dated 07/JUN/2013 (incorporating Global Amendment 1 dated 21/MAY/2013)	18/APR/2012<br><br>03/JUL/2013
Schulman Associates Institutional Review Board, Inc.<br>4445 Lake Forest Drive Suite 300<br>Cincinnati, Ohio 45242	Sharon Lynn Nelson, MSN, RN, CNS	32036 / Salacata, Abraham	Protocol Version 3.0 dated 19/JUL/2011 and Local Amendment dated 17/FEB/2012<br><br>Protocol Version 4.0 dated	12/APR/2012<br><br>03/JUL/2013

United States of America				
IRB or IEC (name/address)	IRB or IEC Chairperson	Centre number (5 digit number) / Investigator(s)	Protocol and/or Amendment number(s)	Date of Final Approval (DD/MMM/YYYY)
ADVARRA<br>6940 Columbia Gateway Drive,<br>Suite 110<br>Columbia, MD 21046	Sara Harnish, J.D		07/JUN/2013 (incorporating Global Amendment 1 dated 21/MAY/2013)	
Schulman Associates Institutional Review Board, Inc.<br>4445 Lake Forest Drive Suite 300<br>Cincinnati, Ohio 45242<br><br>ADVARRA<br>6940 Columbia Gateway Drive,<br>Suite 110<br>Columbia, MD 21046	Sharon Lynn Nelson, MSN, RN, CNS<br><br>Sara Harnish, J.D	32037 / Shah, Neerav Suresh	Protocol Version 3.0 dated 19/JUL/2011 and Local Amendment dated 17/FEB/2012<br><br>Protocol Version 4.0 dated 07/JUN/2013 (incorporating Global Amendment 1 dated 21/MAY/2013)	14/MAR/2012<br><br>03/JUL/2013
Schulman Associates Institutional Review Board, Inc.	Sharon Lynn Nelson, MSN, RN, CNS	32038 / Smith, Richard	Protocol Version 3.0 dated 19/JUL/2011 and Local Amendment dated	03/APR/2012

United States of America				
IRB or IEC (name/address)	IRB or IEC Chairperson	Centre number (5 digit number) / Investigator(s)	Protocol and/or Amendment number(s)	Date of Final Approval (DD/MMM/YYYY)
4445 Lake Forest Drive Suite 300<br>Cincinnati, Ohio 45242			17/FEB/2012	
ADVARRA<br>6940 Columbia Gateway Drive,<br>Suite 110<br>Columbia, MD 21046	Sara Harnish, J.D		Protocol Version 4.0 dated 07/JUN/2013 (incorporating Global Amendment 1 dated 21/MAY/2013)	03/JUL/2013
Schulman Associates Institutional Review Board, Inc.<br>4445 Lake Forest Drive Suite 300<br>Cincinnati, Ohio 45242	Sharon Lynn Nelson, MSN, RN, CNS	32039 / Forman, Steven	Protocol Version 3.0 dated 19/JUL/2011 and Local Amendment dated 17/FEB/2012	30/APR/2012
ADVARRA<br>6940 Columbia Gateway Drive,<br>Suite 110	Sara Harnish, J.D		Protocol Version 4.0 dated 07/JUN/2013 (incorporating Global Amendment 1 dated 21/MAY/2013)	03/JUL/2013

United States of America				
IRB or IEC (name/address)	IRB or IEC Chairperson	Centre number (5 digit number) / Investigator(s)	Protocol and/or Amendment number(s)	Date of Final Approval (DD/MMM/YYYY)
Columbia, MD 21046				
Schulman Associates Institutional Review Board, Inc.<br>4445 Lake Forest Drive Suite 300<br>Cincinnati, Ohio 45242<br><br>ADVARRA<br>6940 Columbia Gateway Drive,<br>Suite 110<br>Columbia, MD 21046	Sharon Lynn Nelson, MSN, RN, CNS<br><br>Sara Harnish, J.D	32042 / Dotani, M. Imran	Protocol Version 3.0 dated 19/JUL/2011 and Local Amendment dated 17/FEB/2012<br><br>Protocol Version 4.0 dated 07/JUN/2013 (incorporating Global Amendment 1 dated 21/MAY/2013)	28/MAR/2012<br><br>03/JUL/2013
Cook County Health IRB<br>Research and Regulatory Affairs<br>1950 W. Polk<br>Rm 9303	Linda Brodsky	32044 / Sattar, Payman	Protocol Version 3.0 dated 19/JUL/2011 and Local Amendment dated 17/FEB/2012	07/AUG/2012

United States of America				
IRB or IEC (name/address)	IRB or IEC Chairperson	Centre number (5 digit number) / Investigator(s)	Protocol and/or Amendment number(s)	Date of Final Approval (DD/MMM/YYYY)
Chicago, IL 60612<br><br>Cook County Health & Hospital System IRB<br>627 South Wood Street<br>Rm 333<br>Chicago, IL 60612			Protocol Version 4.0 dated 07/JUN/2013 (incorporating Global Amendment 1 dated 21/MAY/2013)	06/AUG/2013
Schulman Associates Institutional Review Board, Inc.<br>4445 Lake Forest Drive Suite 300<br>Cincinnati, Ohio 45242<br><br>ADVARRA<br>6940 Columbia Gateway Drive,<br>Suite 110<br>Columbia, MD 21046	Sharon Lynn Nelson, MSN, RN, CNS<br><br>Sara Harnish, J.D	32045 / Mikdadi, Ghiath	Protocol Version 3.0 dated 19/JUL/2011 and Local Amendment dated 17/FEB/2012<br><br>Protocol Version 4.0 dated 07/JUN/2013 (incorporating Global Amendment 1 dated 21/MAY/2013)	24/APR/2012<br><br>03/JUL/2013

United States of America				
IRB or IEC (name/address)	IRB or IEC Chairperson	Centre number (5 digit number) / Investigator(s)	Protocol and/or Amendment number(s)	Date of Final Approval (DD/MMM/YYYY)
Schulman Associates Institutional Review Board, Inc.<br>4445 Lake Forest Drive Suite 300<br>Cincinnati, Ohio 45242<br><br>ADVARRA<br>6940 Columbia Gateway Drive,<br>Suite 110<br>Columbia, MD 21046	Sharon Lynn Nelson, MSN, RN, CNS<br><br>Sara Harnish, J.D	32046 / Bhagwat, Ravi	Protocol Version 3.0 dated 19/JUL/2011 and Local Amendment dated 17/FEB/2012<br><br>Protocol Version 4.0 dated 07/JUN/2013 (incorporating Global Amendment 1 dated 21/MAY/2013)	24/APR/2012<br><br>03/JUL/2013
Schulman Associates Institutional Review Board, Inc.<br>4445 Lake Forest Drive Suite 300<br>Cincinnati, Ohio 45242	Sharon Lynn Nelson, MSN, RN, CNS	32048 / Seidman, Barry	Protocol Version 3.0 dated 19/JUL/2011 and Local Amendment dated 17/FEB/2012<br><br>Protocol Version 4.0 dated	11/APR/2012

United States of America				
IRB or IEC (name/address)	IRB or IEC Chairperson	Centre number (5 digit number) / Investigator(s)	Protocol and/or Amendment number(s)	Date of Final Approval (DD/MMM/YYYY)
ADVARRA<br>6940 Columbia Gateway Drive,<br>Suite 110<br>Columbia, MD 21046	Sara Harnish, J.D		07/JUN/2013 (incorporating Global Amendment 1 dated 21/MAY/2013)	03/JUL/2013
Schulman Associates Institutional Review Board, Inc.<br>4445 Lake Forest Drive Suite 300<br>Cincinnati, Ohio 45242<br><br>ADVARRA<br>6940 Columbia Gateway Drive,<br>Suite 110<br>Columbia, MD 21046	Sharon Lynn Nelson, MSN, RN, CNS<br><br>Sara Harnish, J.D	32049 / Harris, Kenneth	Protocol Version 3.0 dated 19/JUL/2011 and Local Amendment dated 17/FEB/2012<br><br>Protocol Version 4.0 dated 07/JUN/2013 (incorporating Global Amendment 1 dated 21/MAY/2013)	16/MAY/2012<br><br>03/JUL/2013
Sisters of Charity Providence Hospitals IRB	Charles S. Bryan, MD, MACP	32050 / Gottipatty, Venkateshwar	Protocol Version 3.0 dated 19/JUL/2011 and Local Amendment dated	19/JUL/2012

United States of America				
IRB or IEC (name/address)	IRB or IEC Chairperson	Centre number (5 digit number) / Investigator(s)	Protocol and/or Amendment number(s)	Date of Final Approval (DD/MMM/YYYY)
2435 Forest Dr.<br>Columbia, SC 29204			17/FEB/2012<br><br>Protocol Version 4.0 dated 07/JUN/2013 (incorporating Global Amendment 1 dated 21/MAY/2013)	 <br>17/OCT/2013
Schulman Associates Institutional Review Board, Inc.<br>4445 Lake Forest Drive Suite 300<br>Cincinnati, Ohio 45242<br><br>ADVARRA<br>6940 Columbia Gateway Drive,<br>Suite 110<br>Columbia, MD 21046	Sharon Lynn Nelson, MSN, RN, CNS<br><br>Sara Harnish, J.D	32054 / Ismail, Younus	Protocol Version 3.0 dated 19/JUL/2011 and Local Amendment dated 17/FEB/2012<br><br>Protocol Version 4.0 dated 07/JUN/2013 (incorporating Global Amendment 1 dated 21/MAY/2013)	04/MAY/2012<br><br>03/JUL/2013

United States of America				
IRB or IEC (name/address)	IRB or IEC Chairperson	Centre number (5 digit number) / Investigator(s)	Protocol and/or Amendment number(s)	Date of Final Approval (DD/MMM/YYYY)
Schulman Associates Institutional Review Board, Inc.<br>4445 Lake Forest Drive Suite 300<br>Cincinnati, Ohio 45242<br><br>ADVARRA<br>6940 Columbia Gateway Drive,<br>Suite 110<br>Columbia, MD 21046	Sharon Lynn Nelson, MSN, RN, CNS<br><br>Sara Harnish, J.D	32057 / Korabathina, Ravikiran	Protocol Version 3.0 dated 19/JUL/2011 and Local Amendment dated 17/FEB/2012<br><br>Protocol Version 4.0 dated 07/JUN/2013 (incorporating Global Amendment 1 dated 21/MAY/2013)	25/APR/2012<br><br>03/JUL/2013
Schulman Associates Institutional Review Board, Inc.<br>4445 Lake Forest Drive Suite 300<br>Cincinnati, Ohio 45242<br><br>ADVARRA	Sharon Lynn Nelson, MSN, RN, CNS	32059 / Vora, Kishor N.	Protocol Version 3.0 dated 19/JUL/2011 and Local Amendment dated 17/FEB/2012<br><br>Protocol Version 4.0 dated 07/JUN/2013 (incorporating Global	27/MAR/2012<br><br>03/JUL/2013

United States of America				
IRB or IEC (name/address)	IRB or IEC Chairperson	Centre number (5 digit number) / Investigator(s)	Protocol and/or Amendment number(s)	Date of Final Approval (DD/MMM/YYYY)
6940 Columbia Gateway Drive,<br>Suite 110<br>Columbia, MD 21046	Sara Harnish, J.D		Amendment 1 dated 21/MAY/2013)	
Schulman Associates Institutional Review Board, Inc.<br>4445 Lake Forest Drive Suite 300<br>Cincinnati, Ohio 45242<br><br>ADVARRA<br>6940 Columbia Gateway Drive,<br>Suite 110<br>Columbia, MD 21046	Sharon Lynn Nelson, MSN, RN, CNS<br><br>Sara Harnish, J.D	32060 / Hayek, Emil	Protocol Version 3.0 dated 19/JUL/2011 and Local Amendment dated 17/FEB/2012<br><br>Protocol Version 4.0 dated 07/JUN/2013 (incorporating Global Amendment 1 dated 21/MAY/2013)	01/MAY/2012<br><br>03/JUL/2013
Schulman Associates Institutional Review Board, Inc.<br>4445 Lake Forest Drive	Sharon Lynn Nelson, MSN, RN, CNS	32061 / Vlastaris, Anthony G.	Protocol Version 3.0 dated 19/JUL/2011 and Local Amendment dated 17/FEB/2012	25/APR/2012

United States of America				
IRB or IEC (name/address)	IRB or IEC Chairperson	Centre number (5 digit number) / Investigator(s)	Protocol and/or Amendment number(s)	Date of Final Approval (DD/MMM/YYYY)
Suite 300<br>Cincinnati, Ohio 45242<br><br>ADVARRA<br>6940 Columbia Gateway Drive,<br>Suite 110<br>Columbia, MD 21046	 <br><br><br>Sara Harnish, J.D		Protocol Version 4.0 dated 07/JUN/2013 (incorporating Global Amendment 1 dated 21/MAY/2013)	03/JUL/2013
IRB at Tallahassee Memorial Health Care<br>1300 Miccosuke Road<br>Tallahassee, FL 32308	Larry C. Deeb, MD	32064 / Smith, David	Protocol Version 3.0 dated 19/JUL/2011 and Local Amendment dated 17/FEB/2012<br><br>Protocol Version 4.0 dated 07/JUN/2013 (incorporating Global Amendment 1 dated 21/MAY/2013)	04/APR/2013<br><br>30/JAN/2014
Schulman Associates Institutional Review	Sharon Lynn Nelson, MSN, RN, CNS	32066 / Clay, Anthony	Protocol Version 3.0 dated 19/JUL/2011 and Local	23/JUL/2012

United States of America				
IRB or IEC (name/address)	IRB or IEC Chairperson	Centre number (5 digit number) / Investigator(s)	Protocol and/or Amendment number(s)	Date of Final Approval (DD/MMM/YYYY)
Board, Inc.<br>4445 Lake Forest Drive Suite 300<br>Cincinnati, Ohio 45242<br><br>ADVARRA<br>6940 Columbia Gateway Drive,<br>Suite 110<br>Columbia, MD 21046	Sara Harnish, J.D		Amendment dated 17/FEB/2012<br><br>Protocol Version 4.0 dated 07/JUN/2013 (incorporating Global Amendment 1 dated 21/MAY/2013)	03/JUL/2013
Schulman Associates Institutional Review Board, Inc.<br>4445 Lake Forest Drive Suite 300<br>Cincinnati, Ohio 45242<br><br>The Methodist Hospital Research Institute IRB,	Sharon Lynn Nelson, MSN, RN, CNS<br><br>Susan Miller, MD	32068 / Tang, Sherman	Protocol Version 3.0 dated 19/JUL/2011 and Local Amendment dated 17/FEB/2012<br><br>Protocol Version 3.0 dated 19/JUL/2011 and Local	18/MAY/2012<br><br>17/MAY/2013

United States of America				
IRB or IEC (name/address)	IRB or IEC Chairperson	Centre number (5 digit number) / Investigator(s)	Protocol and/or Amendment number(s)	Date of Final Approval (DD/MMM/YYYY)
6670 Bertner, Houston, TX 77030			Amendment dated 17/FEB/2012<br><br>Protocol Version 4.0 dated 07/JUN/2013 (incorporating Global Amendment 1 dated 21/MAY/2013)	 <br>14/JAN/2014
Schulman Associates Institutional Review Board, Inc.<br>4445 Lake Forest Drive Suite 300<br>Cincinnati, Ohio 45242<br><br>ADVARRA<br>6940 Columbia Gateway Drive,<br>Suite 110	Sharon Lynn Nelson, MSN, RN, CNS<br><br>Sara Harnish, J.D	32069 / Perlman, Richard L.	Protocol Version 3.0 dated 19/JUL/2011 and Local Amendment dated 17/FEB/2012<br><br>Protocol Version 4.0 dated 07/JUN/2013 (incorporating Global Amendment 1 dated 21/MAY/2013)	08/JUN/2012<br><br>03/JUL/2013

United States of America				
IRB or IEC (name/address)	IRB or IEC Chairperson	Centre number (5 digit number) / Investigator(s)	Protocol and/or Amendment number(s)	Date of Final Approval (DD/MMM/YYYY)
Columbia, MD 21046				
Schulman Associates Institutional Review Board, Inc.<br>4445 Lake Forest Drive Suite 300<br>Cincinnati, Ohio 45242<br><br>ADVARRA<br>6940 Columbia Gateway Drive,<br>Suite 110<br>Columbia, MD 21046	Sharon Lynn Nelson, MSN, RN, CNS<br><br>Sara Harnish, J.D	32071 / Lehman Jr., John J.	Protocol Version 3.0 dated 19/JUL/2011 and Local Amendment dated 17/FEB/2012<br><br>Protocol Version 4.0 dated 07/JUN/2013 (incorporating Global Amendment 1 dated 21/MAY/2013)	07/MAY/2012<br><br>03/JUL/2013
Schulman Associates Institutional Review Board, Inc.<br>4445 Lake Forest Drive Suite 300<br>Cincinnati, Ohio 45242	Sharon Lynn Nelson, MSN, RN, CNS	32073 / Hakas, Joseph	Protocol Version 3.0 dated 19/JUL/2011 and Local Amendment dated 17/FEB/2012	11/MAY/2012

United States of America				
IRB or IEC (name/address)	IRB or IEC Chairperson	Centre number (5 digit number) / Investigator(s)	Protocol and/or Amendment number(s)	Date of Final Approval (DD/MMM/YYYY)
ADVARRA<br>6940 Columbia Gateway Drive,<br>Suite 110<br>Columbia, MD 21046	Sara Harnish, J.D		Protocol Version 4.0 dated 07/JUN/2013 (incorporating Global Amendment 1 dated 21/MAY/2013)	03/JUL/2013
Schulman Associates Institutional Review Board, Inc.<br>4445 Lake Forest Drive Suite 300<br>Cincinnati, Ohio 45242<br><br>ADVARRA<br>6940 Columbia Gateway Drive,<br>Suite 110<br>Columbia, MD 21046	Sharon Lynn Nelson, MSN, RN, CNS<br><br>Sara Harnish, J.D	32075 / McClure II, John Miles	Protocol Version 3.0 dated 19/JUL/2011 and Local Amendment dated 17/FEB/2012<br><br>Protocol Version 4.0 dated 07/JUN/2013 (incorporating Global Amendment 1 dated 21/MAY/2013)	05/JUL/2012<br><br>03/JUL/2013
Schulman Associates Institutional Review Board, Inc.	Sharon Lynn Nelson, MSN, RN, CNS	32079 / Browne, Jr., Kevin F.	Protocol Version 3.0 dated 19/JUL/2011 and Local Amendment dated	09/MAY/2012

United States of America				
IRB or IEC (name/address)	IRB or IEC Chairperson	Centre number (5 digit number) / Investigator(s)	Protocol and/or Amendment number(s)	Date of Final Approval (DD/MMM/YYYY)
4445 Lake Forest Drive Suite 300<br>Cincinnati, Ohio 45242<br><br>ADVARRA<br>6940 Columbia Gateway Drive,<br>Suite 110<br>Columbia, MD 21046	 <br><br>Sara Harnish, J.D		17/FEB/2012<br><br>Protocol Version 4.0 dated 07/JUN/2013 (incorporating Global Amendment 1 dated 21/MAY/2013)	 <br>03/JUL/2013
Schulman Associates Institutional Review Board, Inc.<br>4445 Lake Forest Drive Suite 300<br>Cincinnati, Ohio 45242<br><br>ADVARRA<br>6940 Columbia Gateway Drive,<br>Suite 110	Sharon Lynn Nelson, MSN, RN, CNS<br><br><br>Sara Harnish, J.D	32080 / Wakefield, Paul H.	Protocol Version 3.0 dated 19/JUL/2011 and Local Amendment dated 17/FEB/2012<br><br>Protocol Version 4.0 dated 07/JUN/2013 (incorporating Global Amendment 1 dated 21/MAY/2013)	29/MAR/2012<br><br><br>03/JUL/2013

United States of America				
IRB or IEC (name/address)	IRB or IEC Chairperson	Centre number (5 digit number) / Investigator(s)	Protocol and/or Amendment number(s)	Date of Final Approval (DD/MMM/YYYY)
Columbia, MD 21046				
Schulman Associates Institutional Review Board, Inc.<br>4445 Lake Forest Drive Suite 300<br>Cincinnati, Ohio 45242<br><br>ADVARRA<br>6940 Columbia Gateway Drive,<br>Suite 110<br>Columbia, MD 21046	Sharon Lynn Nelson, MSN, RN, CNS<br><br>Sara Harnish, J.D	32082 / Manning, Ricky D.	Protocol Version 3.0 dated 19/JUL/2011 and Local Amendment dated 17/FEB/2012<br><br>Protocol Version 4.0 dated 07/JUN/2013 (incorporating Global Amendment 1 dated 21/MAY/2013)	30/MAY/2012<br><br>03/JUL/2013
Schulman Associates Institutional Review Board, Inc.<br>4445 Lake Forest Drive Suite 300<br>Cincinnati, Ohio 45242	Sharon Lynn Nelson, MSN, RN, CNS	32083 / McGarity, Jr., William C.	Protocol Version 3.0 dated 19/JUL/2011 and Local Amendment dated 17/FEB/2012	23/MAY/2012

United States of America				
IRB or IEC (name/address)	IRB or IEC Chairperson	Centre number (5 digit number) / Investigator(s)	Protocol and/or Amendment number(s)	Date of Final Approval (DD/MMM/YYYY)
ADVARRA<br>6940 Columbia Gateway Drive,<br>Suite 110<br>Columbia, MD 21046	Sara Harnish, J.D		Protocol Version 4.0 dated 07/JUN/2013 (incorporating Global Amendment 1 dated 21/MAY/2013)	03/JUL/2013
Schulman Associates Institutional Review Board, Inc.<br>4445 Lake Forest Drive Suite 300<br>Cincinnati, Ohio 45242<br><br>ADVARRA<br>6940 Columbia Gateway Drive,<br>Suite 110<br>Columbia, MD 21046	Sharon Lynn Nelson, MSN, RN, CNS<br><br>Sara Harnish, J.D	32090 / Vogel, Craig D.	Protocol Version 3.0 dated 19/JUL/2011 and Local Amendment dated 17/FEB/2012<br><br>Protocol Version 4.0 dated 07/JUN/2013 (incorporating Global Amendment 1 dated 21/MAY/2013)	16/MAY/2012<br><br>03/JUL/2013
Schulman Associates Institutional Review Board, Inc.	Sharon Lynn Nelson, MSN, RN, CNS	32092 / Karim, Amin Haji	Protocol Version 3.0 dated 19/JUL/2011 and Local Amendment dated	10/MAY/2012

United States of America				
IRB or IEC (name/address)	IRB or IEC Chairperson	Centre number (5 digit number) / Investigator(s)	Protocol and/or Amendment number(s)	Date of Final Approval (DD/MMM/YYYY)
4445 Lake Forest Drive Suite 300<br>Cincinnati, Ohio 45242<br><br>ADVARRA<br>6940 Columbia Gateway Drive,<br>Suite 110<br>Columbia, MD 21046	 <br><br>Sara Harnish, J.D		17/FEB/2012<br><br>Protocol Version 4.0 dated 07/JUN/2013 (incorporating Global Amendment 1 dated 21/MAY/2013)	 <br>03/JUL/2013
Schulman Associates Institutional Review Board, Inc.<br>4445 Lake Forest Drive Suite 300<br>Cincinnati, Ohio 45242<br><br>ADVARRA<br>6940 Columbia Gateway Drive,<br>Suite 110	Sharon Lynn Nelson, MSN, RN, CNS<br><br><br>Sara Harnish, J.D	32095 / Sotolongo, Rodolfo	Protocol Version 3.0 dated 19/JUL/2011 and Local Amendment dated 17/FEB/2012<br><br>Protocol Version 4.0 dated 07/JUN/2013 (incorporating Global Amendment 1 dated 21/MAY/2013)	06/APR/2012<br><br><br>03/JUL/2013

United States of America				
IRB or IEC (name/address)	IRB or IEC Chairperson	Centre number (5 digit number) / Investigator(s)	Protocol and/or Amendment number(s)	Date of Final Approval (DD/MMM/YYYY)
Columbia, MD 21046				
Schulman Associates Institutional Review Board, Inc.<br>4445 Lake Forest Drive Suite 300<br>Cincinnati, Ohio 45242<br><br>ADVARRA<br>6940 Columbia Gateway Drive,<br>Suite 110<br>Columbia, MD 21046	Sharon Lynn Nelson, MSN, RN, CNS<br><br>Sara Harnish, J.D	32096 / Jenkins, Ronald	Protocol Version 3.0 dated 19/JUL/2011 and Local Amendment dated 17/FEB/2012<br><br>Protocol Version 4.0 dated 07/JUN/2013 (incorporating Global Amendment 1 dated 21/MAY/2013)	26/APR/2012<br><br>03/JUL/2013
Schulman Associates Institutional Review Board, Inc.<br>4445 Lake Forest Drive Suite 300<br>Cincinnati, Ohio 45242	Sharon Lynn Nelson, MSN, RN, CNS	32099 / Singal, Dinesh K.	Protocol Version 3.0 dated 19/JUL/2011 and Local Amendment dated 17/FEB/2012	26/APR/2012

United States of America				
IRB or IEC (name/address)	IRB or IEC Chairperson	Centre number (5 digit number) / Investigator(s)	Protocol and/or Amendment number(s)	Date of Final Approval (DD/MMM/YYYY)
ADVARRA<br>6940 Columbia Gateway Drive,<br>Suite 110<br>Columbia, MD 21046	Sara Harnish, J.D		Protocol Version 4.0 dated 07/JUN/2013 (incorporating Global Amendment 1 dated 21/MAY/2013)	03/JUL/2013
Schulman Associates Institutional Review Board, Inc.<br>4445 Lake Forest Drive Suite 300<br>Cincinnati, Ohio 45242<br><br>ADVARRA<br>6940 Columbia Gateway Drive,<br>Suite 110<br>Columbia, MD 21046	Sharon Lynn Nelson, MSN, RN, CNS<br><br>Sara Harnish, J.D	32100 / Troyan, Barry	Protocol Version 3.0 dated 19/JUL/2011 and Local Amendment dated 17/FEB/2012<br><br>Protocol Version 4.0 dated 07/JUN/2013 (incorporating Global Amendment 1 dated 21/MAY/2013)	02/MAY/2012<br><br>03/JUL/2013
Schulman Associates Institutional Review	Sharon Lynn Nelson, MSN, RN, CNS	32101 / Al-Joundi, Tammam	Protocol Version 3.0 dated 19/JUL/2011 and Local	22/MAY/2012

United States of America				
IRB or IEC (name/address)	IRB or IEC Chairperson	Centre number (5 digit number) / Investigator(s)	Protocol and/or Amendment number(s)	Date of Final Approval (DD/MMM/YYYY)
Board, Inc.<br>4445 Lake Forest Drive Suite 300<br>Cincinnati, Ohio 45242<br>ADVARRA<br>6940 Columbia Gateway Drive,<br>Suite 110<br>Columbia, MD 21046	Sara Harnish, J.D		Amendment dated 17/FEB/2012<br><br>Protocol Version 4.0 dated 07/JUN/2013 (incorporating Global Amendment 1 dated 21/MAY/2013)	03/JUL/2013
Northwestern University IRB<br>750 N. Lake Shore Dr.<br>Suite 700<br>Chicago, IL 60611	Darren Gitelman, MD	32103 / Bernstein, Richard	Protocol Version 3.0 dated 19/JUL/2011 and Local Amendment dated 17/FEB/2012<br><br>Protocol Version 4.0 dated 07/JUN/2013 (incorporating Global Amendment 1 dated 21/MAY/2013)	03/MAY/2012<br><br>07/OCT/2013

United States of America				
IRB or IEC (name/address)	IRB or IEC Chairperson	Centre number (5 digit number) / Investigator(s)	Protocol and/or Amendment number(s)	Date of Final Approval (DD/MMM/YYYY)
Schulman Associates Institutional Review Board, Inc.<br>4445 Lake Forest Drive Suite 300<br>Cincinnati, Ohio 45242<br><br>ADVARRA<br>6940 Columbia Gateway Drive,<br>Suite 110<br>Columbia, MD 21046	Sharon Lynn Nelson, MSN, RN, CNS<br><br>Sara Harnish, J.D	32104 / Cislowski, David	Protocol Version 3.0 dated 19/JUL/2011 and Local Amendment dated 17/FEB/2012<br><br>Protocol Version 4.0 dated 07/JUN/2013 (incorporating Global Amendment 1 dated 21/MAY/2013)	22/MAY/2012<br><br>03/JUL/2013
Schulman Associates Institutional Review Board, Inc.<br>4445 Lake Forest Drive Suite 300<br>Cincinnati, Ohio 45242<br><br>ADVARRA	Sharon Lynn Nelson, MSN, RN, CNS	32109 / Topkis, Robert	Protocol Version 3.0 dated 19/JUL/2011 and Local Amendment dated 17/FEB/2012<br><br>Protocol Version 4.0 dated	30/MAY/2012

United States of America				
IRB or IEC (name/address)	IRB or IEC Chairperson	Centre number (5 digit number) / Investigator(s)	Protocol and/or Amendment number(s)	Date of Final Approval (DD/MMM/YYYY)
6940 Columbia Gateway Drive,<br>Suite 110<br>Columbia, MD 21046	Sara Harnish, J.D		07/JUN/2013 (incorporating Global Amendment 1 dated 21/MAY/2013)	03/JUL/2013
Schulman Associates Institutional Review Board, Inc.<br>4445 Lake Forest Drive Suite 300<br>Cincinnati, Ohio 45242<br><br>ADVARRA<br>6940 Columbia Gateway Drive,<br>Suite 110<br>Columbia, MD 21046	Sharon Lynn Nelson, MSN, RN, CNS<br><br>Sara Harnish, J.D	32113 / Lorraine, Richard Scott	Protocol Version 3.0 dated 19/JUL/2011 and Local Amendment dated 17/FEB/2012<br><br>Protocol Version 4.0 dated 07/JUN/2013 (incorporating Global Amendment 1 dated 21/MAY/2013)	08/JUN/2012<br><br>03/JUL/2013
Schulman Associates Institutional Review Board, Inc.<br>4445 Lake Forest Drive	Sharon Lynn Nelson, MSN, RN, CNS	32116 / Baula, Giovanni	Protocol Version 3.0 dated 19/JUL/2011 and Local Amendment dated 17/FEB/2012	14/JUN/2012

United States of America				
IRB or IEC (name/address)	IRB or IEC Chairperson	Centre number (5 digit number) / Investigator(s)	Protocol and/or Amendment number(s)	Date of Final Approval (DD/MMM/YYYY)
Suite 300<br>Cincinnati, Ohio 45242				
ADVARRA<br>6940 Columbia Gateway Drive,<br>Suite 110<br>Columbia, MD 21046	Sara Harnish, J.D		Protocol Version 4.0 dated 07/JUN/2013 (incorporating Global Amendment 1 dated 21/MAY/2013)	03/JUL/2013
Schulman Associates Institutional Review Board, Inc.<br>4445 Lake Forest Drive Suite 300<br>Cincinnati, Ohio 45242	Sharon Lynn Nelson, MSN, RN, CNS	32117 / Aguilar, Francisco	Protocol Version 3.0 dated 19/JUL/2011 and Local Amendment dated 17/FEB/2012 and Protocol Version 4.0 dated 07/JUN/2013 (incorporating Global Amendment 1 dated 21/MAY/2013)	17/OCT/2014
ADVARRA<br>6940 Columbia Gateway Drive,<br>Suite 110<br>Columbia, MD 21046	Sara Harnish, J.D		Protocol Version 3.0 dated 19/JUL/2011 and Local Amendment dated 17/FEB/2012	13/JUN/2012

United States of America				
IRB or IEC (name/address)	IRB or IEC Chairperson	Centre number (5 digit number) / Investigator(s)	Protocol and/or Amendment number(s)	Date of Final Approval (DD/MMM/YYYY)
Adventist Midwest Health, Research Review Board, La Grange Treatment Pavilion, 1325 Memorial Dr., La Grange, Illinois 60525	Rebecca C. Preston, MD		Protocol Version 4.0 dated 07/JUN/2013 (incorporating Global Amendment 1 dated 21/MAY/2013)	11/SEP/2014
Schulman Associates Institutional Review Board, Inc.<br>4445 Lake Forest Drive Suite 300<br>Cincinnati, Ohio 45242<br><br>ADVARRA<br>6940 Columbia Gateway Drive,<br>Suite 110<br>Columbia, MD 21046	Sharon Lynn Nelson, MSN, RN, CNS<br><br>Sara Harnish, J.D	32122 / Reza, Shabbir	Protocol Version 3.0 dated 19/JUL/2011 and Local Amendment dated 17/FEB/2012<br><br>Protocol Version 4.0 dated 07/JUN/2013 (incorporating Global Amendment 1 dated 21/MAY/2013)	09/JUL/2012<br><br>03/JUL/2013

United States of America				
IRB or IEC (name/address)	IRB or IEC Chairperson	Centre number (5 digit number) / Investigator(s)	Protocol and/or Amendment number(s)	Date of Final Approval (DD/MMM/YYYY)
Sacred Heart Health System IRB<br>5151 N. Ninth Ave.<br>P.O. Box 2700<br>Pensacola, FL 32513	Mary Foscue, MD	32125 / Phlaum, Stephen	Protocol Version 3.0 dated 19/JUL/2011 and Local Amendment dated 17/FEB/2012<br>Protocol Version 4.0 dated 07/JUN/2013 (incorporating Global Amendment 1 dated 21/MAY/2013)	10/JUL/2012<br>17/SEP/2013
Schulman Associates Institutional Review Board, Inc.<br>4445 Lake Forest Drive Suite 300<br>Cincinnati, Ohio 45242<br>ADVARRA	Sharon Lynn Nelson, MSN, RN, CNS	32126 / Pham, David N.	Protocol Version 3.0 dated 19/JUL/2011 and Local Amendment dated 17/FEB/2012<br>Protocol Version 4.0 dated 07/JUN/2013	07/JUN/2012<br>03/JUL/2013

United States of America				
IRB or IEC (name/address)	IRB or IEC Chairperson	Centre number (5 digit number) / Investigator(s)	Protocol and/or Amendment number(s)	Date of Final Approval (DD/MMM/YYYY)
6940 Columbia Gateway Drive,<br>Suite 110<br>Columbia, MD 21046	Sara Harnish, J.D		(incorporating Global Amendment 1 dated 21/MAY/2013)	
Schulman Associates Institutional Review Board, Inc.<br>4445 Lake Forest Drive Suite 300<br>Cincinnati, Ohio 45242<br><br>ADVARRA<br>6940 Columbia Gateway Drive,<br>Suite 110<br>Columbia, MD 21046	Sharon Lynn Nelson, MSN, RN, CNS<br><br>Sara Harnish, J.D	32127 / Patel, Rajesh J.	Protocol Version 3.0 dated 19/JUL/2011 and Local Amendment dated 17/FEB/2012<br><br>Protocol Version 4.0 dated 07/JUN/2013 (incorporating Global Amendment 1 dated 21/MAY/2013)	08/JUN/2012<br><br>03/JUL/2013
Schulman Associates Institutional Review Board, Inc.<br>4445 Lake Forest Drive	Sharon Lynn Nelson, MSN, RN, CNS	32132 / Koons, Jay	Protocol Version 3.0 dated 19/JUL/2011 and Local Amendment dated 17/FEB/2012	14/JUN/2012

United States of America				
IRB or IEC (name/address)	IRB or IEC Chairperson	Centre number (5 digit number) / Investigator(s)	Protocol and/or Amendment number(s)	Date of Final Approval (DD/MMM/YYYY)
Suite 300<br>Cincinnati, Ohio 45242			Protocol Version 4.0 dated 07/JUN/2013 (incorporating Global Amendment 1 dated 21/MAY/2013)	03/JUL/2013
ADVARRA<br>6940 Columbia Gateway Drive,<br>Suite 110<br>Columbia, MD 21046	Sara Harnish, J.D			
Schulman Associates Institutional Review Board, Inc.<br>4445 Lake Forest Drive Suite 300<br>Cincinnati, Ohio 45242	Sharon Lynn Nelson, MSN, RN, CNS	32133 / Koch, Stanley W.	Protocol Version 3.0 dated 19/JUL/2011 and Local Amendment dated 17/FEB/2012	09/JUL/2012
ADVARRA<br>6940 Columbia Gateway Drive,<br>Suite 110	Sara Harnish, J.D		Protocol Version 4.0 dated 07/JUN/2013 (incorporating Global Amendment 1 dated	03/JUL/2013

United States of America				
IRB or IEC (name/address)	IRB or IEC Chairperson	Centre number (5 digit number) / Investigator(s)	Protocol and/or Amendment number(s)	Date of Final Approval (DD/MMM/YYYY)
Columbia, MD 21046			21/MAY/2013)	
Henry Ford Health System<br>2799 West Grand Blvd.<br>CFP-bsmt, Rm 46<br>Detroit, MI 48202	Jonathon Ehrman, PhD<br>Timothy Roehrs, PhD	32135 / Shah, Vinay	Protocol Version 3.0 dated 19/JUL/2011 and Local Amendment dated 17/FEB/2012<br><br>Protocol Version 4.0 dated 07/JUN/2013 (incorporating Global Amendment 1 dated 21/MAY/2013)	23/MAY/2012<br><br>06/SEP/2013
Schulman Associates Institutional Review Board, Inc.<br>4445 Lake Forest Drive Suite 300<br>Cincinnati, Ohio 45242<br><br>ADVARRA	Sharon Lynn Nelson, MSN, RN, CNS<br><br>Sara Harnish, J.D	32137 / Grena, Paul Gregory	Protocol Version 3.0 dated 19/JUL/2011 and Local Amendment dated 17/FEB/2012<br><br>Protocol Version 4.0 dated 07/JUN/2013 (incorporating Global	29/JUN/2012<br><br>03/JUL/2013

United States of America				
IRB or IEC (name/address)	IRB or IEC Chairperson	Centre number (5 digit number) / Investigator(s)	Protocol and/or Amendment number(s)	Date of Final Approval (DD/MMM/YYYY)
6940 Columbia Gateway Drive,<br>Suite 110<br>Columbia, MD 21046			Amendment 1 dated 21/MAY/2013)	
Schulman Associates Institutional Review Board, Inc.<br>4445 Lake Forest Drive Suite 300<br>Cincinnati, Ohio 45242<br><br>ADVARRA<br>6940 Columbia Gateway Drive,<br>Suite 110<br>Columbia, MD 21046	Sharon Lynn Nelson, MSN, RN, CNS<br><br>Sara Harnish, J.D	32139 / Gabris, Michael	Protocol Version 3.0 dated 19/JUL/2011 and Local Amendment dated 17/FEB/2012<br><br>Protocol Version 4.0 dated 07/JUN/2013 (incorporating Global Amendment 1 dated 21/MAY/2013)	15/JUN/2012<br><br>03/JUL/2013
Schulman Associates Institutional Review Board, Inc.<br>4445 Lake Forest Drive	Sharon Lynn Nelson, MSN, RN, CNS	32140 / Frais, Michael	Protocol Version 3.0 dated 19/JUL/2011 and Local Amendment dated 17/FEB/2012	18/JUN/2012

United States of America				
IRB or IEC (name/address)	IRB or IEC Chairperson	Centre number (5 digit number) / Investigator(s)	Protocol and/or Amendment number(s)	Date of Final Approval (DD/MMM/YYYY)
Suite 300<br>Cincinnati, Ohio 45242<br><br>ADVARRA<br>6940 Columbia Gateway Drive,<br>Suite 110<br>Columbia, MD 21046	Sara Harnish, J.D		Protocol Version 4.0 dated 07/JUN/2013 (incorporating Global Amendment 1 dated 21/MAY/2013)	03/JUL/2013
Schulman Associates Institutional Review Board, Inc.<br>4445 Lake Forest Drive Suite 300<br>Cincinnati, Ohio 45242<br><br>ADVARRA<br>6940 Columbia Gateway Drive,<br>Suite 110	Sharon Lynn Nelson, MSN, RN, CNS<br><br>Sara Harnish, J.D	32142 / Evonich III, Rudolph F.	Protocol Version 3.0 dated 19/JUL/2011 and Local Amendment dated 17/FEB/2012<br><br>Protocol Version 4.0 dated 07/JUN/2013 (incorporating Global Amendment 1 dated 21/MAY/2013)	20/JUN/2012<br><br>03/JUL/2013

United States of America				
IRB or IEC (name/address)	IRB or IEC Chairperson	Centre number (5 digit number) / Investigator(s)	Protocol and/or Amendment number(s)	Date of Final Approval (DD/MMM/YYYY)
Columbia, MD 21046				
Schulman Associates Institutional Review Board, Inc.<br>4445 Lake Forest Drive Suite 300<br>Cincinnati, Ohio 45242<br><br>ADVARRA<br>6940 Columbia Gateway Drive,<br>Suite 110<br>Columbia, MD 21046	Sharon Lynn Nelson, MSN, RN, CNS<br><br>Sara Harnish, J.D	32144 / Betzu, Robert M.	Protocol Version 3.0 dated 19/JUL/2011 and Local Amendment dated 17/FEB/2012<br><br>Protocol Version 4.0 dated 07/JUN/2013 (incorporating Global Amendment 1 dated 21/MAY/2013)	28/JUN/2012<br><br>03/JUL/2013
Schulman Associates Institutional Review Board, Inc.<br>4445 Lake Forest Drive Suite 300<br>Cincinnati, Ohio 45242	Sharon Lynn Nelson, MSN, RN, CNS	32145 / Amjadi, Nima	Protocol Version 3.0 dated 19/JUL/2011 and Local Amendment dated 17/FEB/2012<br><br>Protocol Version 4.0 dated	06/JUN2012

United States of America				
IRB or IEC (name/address)	IRB or IEC Chairperson	Centre number (5 digit number) / Investigator(s)	Protocol and/or Amendment number(s)	Date of Final Approval (DD/MMM/YYYY)
ADVARRA<br>6940 Columbia Gateway Drive,<br>Suite 110<br>Columbia, MD 21046	Sara Harnish, J.D		07/JUN/2013 (incorporating Global Amendment 1 dated 21/MAY/2013)	03/JUL/2013
Schulman Associates Institutional Review Board, Inc.<br>4445 Lake Forest Drive Suite 300<br>Cincinnati, Ohio 45242<br><br>ADVARRA<br>6940 Columbia Gateway Drive,<br>Suite 110<br>Columbia, MD 21046	Sharon Lynn Nelson, MSN, RN, CNS<br><br>Sara Harnish, J.D	32146 / Rashid, Haroon	Protocol Version 3.0 dated 19/JUL/2011 and Local Amendment dated 17/FEB/2012<br><br>Protocol Version 4.0 dated 07/JUN/2013 (incorporating Global Amendment 1 dated 21/MAY/2013)	21/MAR/2012<br><br>03/JUL/2013

United States of America				
IRB or IEC (name/address)	IRB or IEC Chairperson	Centre number (5 digit number) / Investigator(s)	Protocol and/or Amendment number(s)	Date of Final Approval (DD/MMM/YYYY)
Schulman Associates Institutional Review Board, Inc.<br>4445 Lake Forest Drive Suite 300<br>Cincinnati, Ohio 45242<br>ADVARRA<br>6940 Columbia Gateway Drive,<br>Suite 110<br>Columbia, MD 21046	Sharon Lynn Nelson, MSN, RN, CNS<br>Sara Harnish, J.D	32148 / Nsah, Emmanuel N.	Protocol Version 3.0 dated 19/JUL/2011 and Local Amendment dated 17/FEB/2012<br>Protocol Version 4.0 dated 07/JUN/2013 (incorporating Global Amendment 1 dated 21/MAY/2013)	16/JUL/2012<br>03/JUL/2013
Schulman Associates Institutional Review Board, Inc.<br>4445 Lake Forest Drive Suite 300<br>Cincinnati, Ohio 45242<br>ADVARRA<br>6940 Columbia Gateway	Sharon Lynn Nelson, MSN, RN, CNS<br>Sara Harnish, J.D	32151 / Aliyar, Pareed	Protocol Version 3.0 dated 19/JUL/2011 and Local Amendment dated 17/FEB/2012<br>Protocol Version 4.0 dated 07/JUN/2013	27/JUN/2012<br>03/JUL/2013

United States of America				
IRB or IEC (name/address)	IRB or IEC Chairperson	Centre number (5 digit number) / Investigator(s)	Protocol and/or Amendment number(s)	Date of Final Approval (DD/MMM/YYYY)
Drive,<br>Suite 110<br>Columbia, MD 21046			(incorporating Global Amendment 1 dated 21/MAY/2013)	
Schulman Associates Institutional Review Board, Inc.<br>4445 Lake Forest Drive Suite 300<br>Cincinnati, Ohio 45242<br><br>ADVARRA<br>6940 Columbia Gateway Drive,<br>Suite 110<br>Columbia, MD 21046	Sharon Lynn Nelson, MSN, RN, CNS<br><br>Sara Harnish, J.D	32152 / D'Souza, Anthony W.	Protocol Version 3.0 dated 19/JUL/2011 and Local Amendment dated 17/FEB/2012<br><br>Protocol Version 4.0 dated 07/JUN/2013 (incorporating Global Amendment 1 dated 21/MAY/2013)	03/JUL/2012<br><br>03/JUL/2013
Schulman Associates Institutional Review Board, Inc.<br>4445 Lake Forest Drive Suite 300	Sharon Lynn Nelson, MSN, RN, CNS	32153 / Ehrlich, Clifford L.	Protocol Version 3.0 dated 19/JUL/2011 and Local Amendment dated 17/FEB/2012	09/JUL/2012

United States of America				
IRB or IEC (name/address)	IRB or IEC Chairperson	Centre number (5 digit number) / Investigator(s)	Protocol and/or Amendment number(s)	Date of Final Approval (DD/MMM/YYYY)
Cincinnati, Ohio 45242<br><br>ADVARRA<br>6940 Columbia Gateway Drive,<br>Suite 110<br>Columbia, MD 21046	Sara Harnish, J.D		Protocol Version 4.0 dated 07/JUN/2013 (incorporating Global Amendment 1 dated 21/MAY/2013)	03/JUL/2013
Schulman Associates Institutional Review Board, Inc.<br>4445 Lake Forest Drive Suite 300<br>Cincinnati, Ohio 45242<br><br>ADVARRA<br>6940 Columbia Gateway Drive,<br>Suite 110<br>Columbia, MD 21046	Sharon Lynn Nelson, MSN, RN, CNS<br><br>Sara Harnish, J.D	32154 / Georgeson, Steven	Protocol Version 3.0 dated 19/JUL/2011 and Local Amendment dated 17/FEB/2012<br><br>Protocol Version 4.0 dated 07/JUN/2013 (incorporating Global Amendment 1 dated 21/MAY/2013)	06/JUL/2012<br><br>03/JUL/2013

United States of America				
IRB or IEC (name/address)	IRB or IEC Chairperson	Centre number (5 digit number) / Investigator(s)	Protocol and/or Amendment number(s)	Date of Final Approval (DD/MMM/YYYY)
Schulman Associates Institutional Review Board, Inc.<br>4445 Lake Forest Drive Suite 300<br>Cincinnati, Ohio 45242<br><br>ADVARRA<br>6940 Columbia Gateway Drive,<br>Suite 110<br>Columbia, MD 21046	Sharon Lynn Nelson, MSN, RN, CNS<br><br>Sara Harnish, J.D	32155 / Hearne, Steven E.	Protocol Version 3.0 dated 19/JUL/2011 and Local Amendment dated 17/FEB/2012<br><br>Protocol Version 4.0 dated 07/JUN/2013 (incorporating Global Amendment 1 dated 21/MAY/2013)	16/JUL/2012<br><br>03/JUL/2013
Schulman Associates Institutional Review Board, Inc.<br>4445 Lake Forest Drive Suite 300<br>Cincinnati, Ohio 45242<br><br>ADVARRA	Sharon Lynn Nelson, MSN, RN, CNS	32156 / Igbokidi, Oyidie	Protocol Version 3.0 dated 19/JUL/2011 and Local Amendment dated 17/FEB/2012<br><br>Protocol Version 4.0 dated 07/JUN/2013	28/JUN/2012<br><br>03/JUL/2013

United States of America				
IRB or IEC (name/address)	IRB or IEC Chairperson	Centre number (5 digit number) / Investigator(s)	Protocol and/or Amendment number(s)	Date of Final Approval (DD/MMM/YYYY)
6940 Columbia Gateway Drive,<br>Suite 110<br>Columbia, MD 21046	Sara Harnish, J.D		(incorporating Global Amendment 1 dated 21/MAY/2013)	
Schulman Associates Institutional Review Board, Inc.<br>4445 Lake Forest Drive Suite 300<br>Cincinnati, Ohio 45242<br><br>ADVARRA<br>6940 Columbia Gateway Drive,<br>Suite 110<br>Columbia, MD 21046	Sharon Lynn Nelson, MSN, RN, CNS<br><br>Sara Harnish, J.D	32157 / Kmetzo, James J.	Protocol Version 3.0 dated 19/JUL/2011 and Local Amendment dated 17/FEB/2012<br><br>Protocol Version 4.0 dated 07/JUN/2013 (incorporating Global Amendment 1 dated 21/MAY/2013)	09/JUL/2012<br><br>03/JUL/2013
Schulman Associates Institutional Review Board, Inc.<br>4445 Lake Forest Drive	Sharon Lynn Nelson, MSN, RN, CNS	32158 / Rama, Bhola N.	Protocol Version 3.0 dated 19/JUL/2011 and Local Amendment dated 17/FEB/2012	05/JUL/2012

United States of America				
IRB or IEC (name/address)	IRB or IEC Chairperson	Centre number (5 digit number) / Investigator(s)	Protocol and/or Amendment number(s)	Date of Final Approval (DD/MMM/YYYY)
Suite 300<br>Cincinnati, Ohio 45242<br><br>ADVARRA<br>6940 Columbia Gateway Drive,<br>Suite 110<br>Columbia, MD 21046	Sara Harnish, J.D		Protocol Version 4.0 dated 07/JUN/2013 (incorporating Global Amendment 1 dated 21/MAY/2013)	03/JUL/2013
Schulman Associates Institutional Review Board, Inc.<br>4445 Lake Forest Drive Suite 300<br>Cincinnati, Ohio 45242<br><br>ADVARRA<br>6940 Columbia Gateway Drive,<br>Suite 110	Sharon Lynn Nelson, MSN, RN, CNS<br><br>Sara Harnish, J.D	32160 / Sofley Jr., C. Wilson	Protocol Version 3.0 dated 19/JUL/2011 and Local Amendment dated 17/FEB/2012<br><br>Protocol Version 4.0 dated 07/JUN/2013 (incorporating Global Amendment 1 dated 21/MAY/2013)	20/JUL/2012<br><br>03/JUL/2013

United States of America				
IRB or IEC (name/address)	IRB or IEC Chairperson	Centre number (5 digit number) / Investigator(s)	Protocol and/or Amendment number(s)	Date of Final Approval (DD/MMM/YYYY)
Columbia, MD 21046				
Schulman Associates Institutional Review Board, Inc.<br>4445 Lake Forest Drive Suite 300<br>Cincinnati, Ohio 45242<br>ADVARRA<br>6940 Columbia Gateway Drive,<br>Suite 110<br>Columbia, MD 21046	Sharon Lynn Nelson, MSN, RN, CNS<br>Sara Harnish, J.D	32162 / Jobe, R. Lee	Protocol Version 3.0 dated 19/JUL/2011 and Local Amendment dated 17/FEB/2012<br>Protocol Version 4.0 dated 07/JUN/2013 (incorporating Global Amendment 1 dated 21/MAY/2013)	11/JUL/2012<br>03/JUL/2013
Schulman Associates Institutional Review Board, Inc.<br>4445 Lake Forest Drive Suite 300<br>Cincinnati, Ohio 45242	Sharon Lynn Nelson, MSN, RN, CNS	32163 / Coodley, Greg O.	Protocol Version 3.0 dated 19/JUL/2011 and Local Amendment dated 17/FEB/2012<br>Protocol Version 4.0 dated	20/JUL/2012

United States of America				
IRB or IEC (name/address)	IRB or IEC Chairperson	Centre number (5 digit number) / Investigator(s)	Protocol and/or Amendment number(s)	Date of Final Approval (DD/MMM/YYYY)
ADVARRA<br>6940 Columbia Gateway Drive,<br>Suite 110<br>Columbia, MD 21046	Sara Harnish, J.D		07/JUN/2013 (incorporating Global Amendment 1 dated 21/MAY/2013)	03/JUL/2013
Schulman Associates Institutional Review Board, Inc.<br>4445 Lake Forest Drive Suite 300<br>Cincinnati, Ohio 45242<br><br>ADVARRA<br>6940 Columbia Gateway Drive,<br>Suite 110<br>Columbia, MD 21046	Sharon Lynn Nelson, MSN, RN, CNS<br><br>Sara Harnish, J.D	32164 / Iteld, Bruce	Protocol Version 3.0 dated 19/JUL/2011 and Local Amendment dated 17/FEB/2012<br><br>Protocol Version 4.0 dated 07/JUN/2013 (incorporating Global Amendment 1 dated 21/MAY/2013)	23/JUL/2012<br><br>03/JUL/2013
Schulman Associates Institutional Review Board, Inc.	Sharon Lynn Nelson, MSN, RN, CNS	32165 / Gillespie, Eve L.	Protocol Version 3.0 dated 19/JUL/2011 and Local Amendment dated	10/JUL/2012

United States of America				
IRB or IEC (name/address)	IRB or IEC Chairperson	Centre number (5 digit number) / Investigator(s)	Protocol and/or Amendment number(s)	Date of Final Approval (DD/MMM/YYYY)
4445 Lake Forest Drive Suite 300<br>Cincinnati, Ohio 45242<br><br>ADVARRA<br>6940 Columbia Gateway Drive,<br>Suite 110<br>Columbia, MD 21046	 <br><br>Sara Harnish, J.D		17/FEB/2012<br><br>Protocol Version 4.0 dated 07/JUN/2013 (incorporating Global Amendment 1 dated 21/MAY/2013)	 <br>03/JUL/2013
Schulman Associates Institutional Review Board, Inc.<br>4445 Lake Forest Drive Suite 300<br>Cincinnati, Ohio 45242<br>ADVARRA<br>6940 Columbia Gateway Drive,<br>Suite 110<br>Columbia, MD 21046	Sharon Lynn Nelson, MSN, RN, CNS<br><br>Sara Harnish, J.D	32166 / Gabra, Nashwa W.	Protocol Version 3.0 dated 19/JUL/2011 and Local Amendment dated 17/FEB/2012<br><br>Protocol Version 4.0 dated 07/JUN/2013 (incorporating Global Amendment 1 dated 21/MAY/2013)	27/JUL/2012<br><br>03/JUL/2013

United States of America				
IRB or IEC (name/address)	IRB or IEC Chairperson	Centre number (5 digit number) / Investigator(s)	Protocol and/or Amendment number(s)	Date of Final Approval (DD/MMM/YYYY)
Schulman Associates Institutional Review Board, Inc.<br>4445 Lake Forest Drive Suite 300<br>Cincinnati, Ohio 45242<br><br>ADVARRA<br>6940 Columbia Gateway Drive,<br>Suite 110<br>Columbia, MD 21046	Sharon Lynn Nelson, MSN, RN, CNS<br><br>Sara Harnish, J.D	32167 / Vargas, Michael	Protocol Version 3.0 dated 19/JUL/2011 and Local Amendment dated 17/FEB/2012<br><br>Protocol Version 4.0 dated 07/JUN/2013 (incorporating Global Amendment 1 dated 21/MAY/2013)	18/JUL/2012<br><br>03/JUL/2013
Genesys Health System<br>One Genesys Parkway<br>Grand Blanc, MI 48439	Mark Vogel, PhD	32168 / Ebinger, Mathew	Protocol Version 3.0 dated 19/JUL/2011 and Local Amendment dated 17/FEB/2012<br><br>Protocol Version 4.0 dated 07/JUN/2013 (incorporating Global	12/JUL/2012<br><br>14/AUG/2013

United States of America				
IRB or IEC (name/address)	IRB or IEC Chairperson	Centre number (5 digit number) / Investigator(s)	Protocol and/or Amendment number(s)	Date of Final Approval (DD/MMM/YYYY)
			Amendment 1 dated 21/MAY/2013)	
Schulman Associates Institutional Review Board, Inc.<br>4445 Lake Forest Drive Suite 300<br>Cincinnati, Ohio 45242<br><br>ADVARRA<br>6940 Columbia Gateway Drive,<br>Suite 110<br>Columbia, MD 21046	Sharon Lynn Nelson, MSN, RN, CNS<br><br>Sara Harnish, J.D	32171 / Suneja, Randeep	Protocol Version 3.0 dated 19/JUL/2011 and Local Amendment dated 17/FEB/2012<br><br>Protocol Version 4.0 dated 07/JUN/2013 (incorporating Global Amendment 1 dated 21/MAY/2013)	25/JUN/2012<br><br>03/JUL/2013
Schulman Associates Institutional Review Board, Inc.<br>4445 Lake Forest Drive Suite 300<br>Cincinnati, Ohio 45242	Sharon Lynn Nelson, MSN, RN, CNS	32173 / Nanna, Michael	Protocol Version 3.0 dated 19/JUL/2011 and Local Amendment dated 17/FEB/2012<br><br>Protocol Version 4.0 dated	25/JUL/2012

United States of America				
IRB or IEC (name/address)	IRB or IEC Chairperson	Centre number (5 digit number) / Investigator(s)	Protocol and/or Amendment number(s)	Date of Final Approval (DD/MMM/YYYY)
ADVARRA<br>6940 Columbia Gateway Drive,<br>Suite 110<br>Columbia, MD 21046	Sara Harnish, J.D		07/JUN/2013 (incorporating Global Amendment 1 dated 21/MAY/2013)	03/JUL/2013
Schulman Associates Institutional Review Board, Inc.<br>4445 Lake Forest Drive Suite 300<br>Cincinnati, Ohio 45242	Sharon Lynn Nelson, MSN, RN, CNS	32174 / Williams, David L.	Protocol Version 3.0 dated 19/JUL/2011 and Local Amendment dated 17/FEB/2012	15/AUG/2012
ADVARRA<br>6940 Columbia Gateway Drive,<br>Suite 110<br>Columbia, MD 21046	Sara Harnish, J.D		Protocol Version 4.0 dated 07/JUN/2013 (incorporating Global Amendment 1 dated 21/MAY/2013)	03/JUL/2013

United States of America				
IRB or IEC (name/address)	IRB or IEC Chairperson	Centre number (5 digit number) / Investigator(s)	Protocol and/or Amendment number(s)	Date of Final Approval (DD/MMM/YYYY)
Schulman Associates Institutional Review Board, Inc.<br>4445 Lake Forest Drive Suite 300<br>Cincinnati, Ohio 45242<br><br>ADVARRA<br>6940 Columbia Gateway Drive,<br>Suite 110<br>Columbia, MD 21046	Sharon Lynn Nelson, MSN, RN, CNS<br><br>Sara Harnish, J.D	32176 / Jaffrani, Naseem	Protocol Version 3.0 dated 19/JUL/2011 and Local Amendment dated 17/FEB/2012<br><br>Protocol Version 4.0 dated 07/JUN/2013 (incorporating Global Amendment 1 dated 21/MAY/2013)	11/JUL/2012<br><br>03/JUL/2013
Schulman Associates Institutional Review Board, Inc.<br>4445 Lake Forest Drive Suite 300<br>Cincinnati, Ohio 45242<br><br>ADVARRA	Sharon Lynn Nelson, MSN, RN, CNS	32179 / Renzi, Michael	Protocol Version 3.0 dated 19/JUL/2011 and Local Amendment dated 17/FEB/2012<br><br>Protocol Version 4.0 dated 07/JUN/2013 (incorporating Global	26/APR/2012<br><br>03/JUL/2013

United States of America				
IRB or IEC (name/address)	IRB or IEC Chairperson	Centre number (5 digit number) / Investigator(s)	Protocol and/or Amendment number(s)	Date of Final Approval (DD/MMM/YYYY)
6940 Columbia Gateway Drive,<br>Suite 110<br>Columbia, MD 21046	Sara Harnish, J.D		Amendment 1 dated 21/MAY/2013)	
University of Nevada, Reno Biomedical IRB<br>Research Integrity Office<br>218 Ross Hall/ 331<br>Reno, NV 89557<br><br>Renown Regional Medical Center IRB<br>1155 Mill Street<br>X-19<br>Reno, NV 89502	Richard Bjur, PhD<br><br>John Watson, MD<br>William McHugh, PHD, MD	32180 / Anderson, Ivan	Protocol Version 3.0 dated 19/JUL/2011 and Local Amendment dated 17/FEB/2012<br><br>Protocol Version 4.0 dated 07/JUN/2013 (incorporating Global Amendment 1 dated 21/MAY/2013)	09/MAY/2012<br><br>20/AUG/2013
Schulman Associates Institutional Review Board, Inc.	Sharon Lynn Nelson, MSN, RN, CNS	32184 / Ahmad, Zia	Protocol Version 3.0 dated 19/JUL/2011 and Local Amendment dated	27/AUG/2012

United States of America				
IRB or IEC (name/address)	IRB or IEC Chairperson	Centre number (5 digit number) / Investigator(s)	Protocol and/or Amendment number(s)	Date of Final Approval (DD/MMM/YYYY)
4445 Lake Forest Drive Suite 300<br>Cincinnati, Ohio 45242			17/FEB/2012	
ADVARRA<br>6940 Columbia Gateway Drive,<br>Suite 110<br>Columbia, MD 21046	Sara Harnish, J.D		Protocol Version 4.0 dated 07/JUN/2013 (incorporating Global Amendment 1 dated 21/MAY/2013)	03/JUL/2013
Schulman Associates Institutional Review Board, Inc.<br>4445 Lake Forest Drive Suite 300<br>Cincinnati, Ohio 45242	Sharon Lynn Nelson, MSN, RN, CNS	32185 / Singh, Narendra	Protocol Version 3.0 dated 19/JUL/2011 and Local Amendment dated 17/FEB/2012	24/AUG/2012
ADVARRA<br>6940 Columbia Gateway Drive,	Sara Harnish, J.D		Protocol Version 4.0 dated 07/JUN/2013 (incorporating Global Amendment 1 dated	03/JUL/2013

United States of America				
IRB or IEC (name/address)	IRB or IEC Chairperson	Centre number (5 digit number) / Investigator(s)	Protocol and/or Amendment number(s)	Date of Final Approval (DD/MMM/YYYY)
Suite 110<br>Columbia, MD 21046			21/MAY/2013)	
Schulman Associates Institutional Review Board, Inc.<br>4445 Lake Forest Drive Suite 300<br>Cincinnati, Ohio 45242<br><br>ADVARRA<br>6940 Columbia Gateway Drive,<br>Suite 110<br>Columbia, MD 21046	Sharon Lynn Nelson, MSN, RN, CNS<br><br>Sara Harnish, J.D	32189 / Rubalcava, Frank John	Protocol Version 3.0 dated 19/JUL/2011 and Local Amendment dated 17/FEB/2012<br><br>Protocol Version 4.0 dated 07/JUN/2013 (incorporating Global Amendment 1 dated 21/MAY/2013)	25/JUN/2012<br><br>03/JUL/2013
St. Cloud Hospital IRB<br>1406 6th Ave N<br>St. Could, MN 56303	Mary Phipps, PharmD	32190 / Erickson, Bernard	Protocol Version 3.0 dated 19/JUL/2011 and Local Amendment dated 17/FEB/2012<br><br>Protocol Version 4.0 dated	16/AUG/2012

United States of America				
IRB or IEC (name/address)	IRB or IEC Chairperson	Centre number (5 digit number) / Investigator(s)	Protocol and/or Amendment number(s)	Date of Final Approval (DD/MMM/YYYY)
			07/JUN/2013 (incorporating Global Amendment 1 dated 21/MAY/2013)	19/SEP/2013
Schulman Associates Institutional Review Board, Inc.<br>4445 Lake Forest Drive Suite 300<br>Cincinnati, Ohio 45242<br><br>ADVARRA<br>6940 Columbia Gateway Drive,<br>Suite 110<br>Columbia, MD 21046	Sharon Lynn Nelson, MSN, RN, CNS<br><br>Sara Harnish, J.D	32192 / Hermany, Paul	Protocol Version 3.0 dated 19/JUL/2011 and Local Amendment dated 17/FEB/2012<br><br>Protocol Version 4.0 dated 07/JUN/2013 (incorporating Global Amendment 1 dated 21/MAY/2013)	30/JUL/2012<br><br>03/JUL/2013
Schulman Associates Institutional Review Board, Inc.<br>4445 Lake Forest Drive	Sharon Lynn Nelson, MSN, RN, CNS	32193 / Kosinski, Edward J.	Protocol Version 3.0 dated 19/JUL/2011 and Local Amendment dated 17/FEB/2012	17/JUL/2012

United States of America				
IRB or IEC (name/address)	IRB or IEC Chairperson	Centre number (5 digit number) / Investigator(s)	Protocol and/or Amendment number(s)	Date of Final Approval (DD/MMM/YYYY)
Suite 300<br>Cincinnati, Ohio 45242<br>ADVARRA<br>6940 Columbia Gateway Drive,<br>Suite 110<br>Columbia, MD 21046	Sara Harnish, J.D		Protocol Version 4.0 dated 07/JUN/2013 (incorporating Global Amendment 1 dated 21/MAY/2013)	03/JUL/2013
Schulman Associates Institutional Review Board, Inc.<br>4445 Lake Forest Drive Suite 300<br>Cincinnati, Ohio 45242<br><br>ADVARRA<br>6940 Columbia Gateway Drive,<br>Suite 110<br>Columbia, MD 21046	Sharon Lynn Nelson, MSN, RN, CNS<br><br>Sara Harnish, J.D	32196 / Mayer, Nolan J.	Protocol Version 3.0 dated 19/JUL/2011 and Local Amendment dated 17/FEB/2012<br><br>Protocol Version 4.0 dated 07/JUN/2013 (incorporating Global Amendment 1 dated 21/MAY/2013)	06/AUG/2012<br><br>03/JUL/2013

United States of America				
IRB or IEC (name/address)	IRB or IEC Chairperson	Centre number (5 digit number) / Investigator(s)	Protocol and/or Amendment number(s)	Date of Final Approval (DD/MMM/YYYY)
Schulman Associates Institutional Review Board, Inc.<br>4445 Lake Forest Drive Suite 300<br>Cincinnati, Ohio 45242<br><br>ADVARRA<br>6940 Columbia Gateway Drive,<br>Suite 110<br>Columbia, MD 21046	Sharon Lynn Nelson, MSN, RN, CNS<br><br>Sara Harnish, J.D	32198 / Ukrainski, Gerald James	Protocol Version 3.0 dated 19/JUL/2011 and Local Amendment dated 17/FEB/2012<br><br>Protocol Version 4.0 dated 07/JUN/2013 (incorporating Global Amendment 1 dated 21/MAY/2013)	30/AUG/2012<br><br>03/JUL/2013
Schulman Associates Institutional Review Board, Inc.<br>4445 Lake Forest Drive Suite 300<br>Cincinnati, Ohio 45242<br><br>ADVARRA	Sharon Lynn Nelson, MSN, RN, CNS	32199 / Van De Graaff, Eric	Protocol Version 3.0 dated 19/JUL/2011 and Local Amendment dated 17/FEB/2012<br>Protocol Version 4.0 dated 07/JUN/2013 (incorporating Global Amendment 1 dated	16/AUG/2012<br><br>03/JUL/2013

United States of America				
IRB or IEC (name/address)	IRB or IEC Chairperson	Centre number (5 digit number) / Investigator(s)	Protocol and/or Amendment number(s)	Date of Final Approval (DD/MMM/YYYY)
6940 Columbia Gateway Drive,<br>Suite 110<br>Columbia, MD 21046	Sara Harnish, J.D		21/MAY/2013)	
Schulman Associates Institutional Review Board, Inc.<br>4445 Lake Forest Drive Suite 300<br>Cincinnati, Ohio 45242<br><br>ADVARRA<br>6940 Columbia Gateway Drive,<br>Suite 110<br>Columbia, MD 21046	Sharon Lynn Nelson, MSN, RN, CNS<br><br>Sara Harnish, J.D	32200 / Andrawis, Nabil S.	Protocol Version 3.0 dated 19/JUL/2011 and Local Amendment dated 17/FEB/2012<br><br>Protocol Version 4.0 dated 07/JUN/2013 (incorporating Global Amendment 1 dated 21/MAY/2013)	17/JUL/2012<br><br>03/JUL/2013
Schulman Associates Institutional Review Board, Inc.<br>4445 Lake Forest Drive	Sharon Lynn Nelson, MSN, RN, CNS	32201 / Eaves, William	Protocol Version 3.0 dated 19/JUL/2011 and Local Amendment dated 17/FEB/2012	03/AUG/2012

United States of America				
IRB or IEC (name/address)	IRB or IEC Chairperson	Centre number (5 digit number) / Investigator(s)	Protocol and/or Amendment number(s)	Date of Final Approval (DD/MMM/YYYY)
Suite 300<br><br>Cincinnati, Ohio 45242<br>ADVARRA<br>6940 Columbia Gateway Drive,<br>Suite 110<br>Columbia, MD 21046	Sara Harnish, J.D		Protocol Version 4.0 dated 07/JUN/2013 (incorporating Global Amendment 1 dated 21/MAY/2013)	03/JUL/2013

United States of America				
**IRB or IEC (name/address)**	**IRB or IEC Chairperson**	**Centre number (5 digit number) / Investigator(s)**	**Protocol and/or Amendment number(s)**	Date of Final Approval (DD/MMM/YYYY)
CIRBI<br>7063 Columbia Gateway Dr.<br>Suite 110<br>Columbia, MD 21046	Joy Cavagnaro, PhD	32202 / Eldadah, Zayd	Protocol Version 3.0 dated 19/JUL/2011 and Local Amendment dated 17/FEB/2012<br><br>Protocol Version 4.0	16/MAY/2012

United States of America				
**IRB or IEC (name/address)**	**IRB or IEC Chairperson**	**Centre number (5 digit number) / Investigator(s)**	**Protocol and/or Amendment number(s)**	Date of Final Approval (DD/MMM/YYYY)
			dated 07/JUN/2013 (incorporating Global Amendment 1 dated 21/MAY/2013)	29/APR/2014
Schulman Associates Institutional Review Board, Inc.<br>4445 Lake Forest Drive Suite 300<br>Cincinnati, Ohio 45242<br><br>ADVARRA<br>6940 Columbia Gateway Drive,<br>Suite 110<br>Columbia, MD 21046	Sharon Lynn Nelson, MSN, RN, CNS<br><br>Sara Harnish, J.D	32203 / Hack, Terrence	Protocol Version 3.0 dated 19/JUL/2011 and Local Amendment dated 17/FEB/2012<br><br>Protocol Version 4.0 dated 07/JUN/2013 (incorporating Global Amendment 1 dated 21/MAY/2013)	29/JUN/2012<br><br>03/JUL/2013
Chambersburg Hospital Summit IRB	Patrick Brannac, MD	32206 / Tang, Aylmer	Protocol Version 3.0 dated 19/JUL/2011 and Local	23/AUG/2012

United States of America				
**IRB or IEC (name/address)**	**IRB or IEC Chairperson**	**Centre number (5 digit number) / Investigator(s)**	**Protocol and/or Amendment number(s)**	Date of Final Approval (DD/MMM/YYYY)
112 North Seventh St.<br>Chambersburg, PA 17201			Amendment dated 17/FEB/2012	
Schulman Associates Institutional Review Board, Inc.<br>4445 Lake Forest Drive Suite 300<br>Cincinnati, Ohio 45242<br><br>ADVARRA<br>6940 Columbia Gateway Drive,<br>Suite 110<br>Columbia, MD 21046	Sharon Lynn Nelson, MSN, RN, CNS<br><br>Sara Harnish, J.D	32211 / Lieber, Ira H.	Protocol Version 3.0 dated 19/JUL/2011 and Local Amendment dated 17/FEB/2012<br><br>Protocol Version 4.0 dated 07/JUN/2013 (incorporating Global Amendment 1 dated 21/MAY/2013)	20/SEP/2012<br><br>03/JUL/2013
Schulman Associates Institutional Review Board, Inc.<br>4445 Lake Forest Drive Suite 300	Sharon Lynn Nelson, MSN, RN, CNS	32215 / Chand, Sunil	Protocol Version 3.0 dated 19/JUL/2011 and Local Amendment dated 17/FEB/2012	27/SEP/2012

United States of America				
**IRB or IEC (name/address)**	**IRB or IEC Chairperson**	**Centre number (5 digit number) / Investigator(s)**	**Protocol and/or Amendment number(s)**	Date of Final Approval (DD/MMM/YYYY)
Cincinnati, Ohio 45242<br><br>ADVARRA<br>6940 Columbia Gateway Drive,<br>Suite 110<br>Columbia, MD 21046	Sara Harnish, J.D		Protocol Version 4.0 dated 07/JUN/2013 (incorporating Global Amendment 1 dated 21/MAY/2013)	03/JUL/2013
Schulman Associates Institutional Review Board, Inc.<br>4445 Lake Forest Drive Suite 300<br>Cincinnati, Ohio 45242<br><br>ADVARRA<br>6940 Columbia Gateway Drive,<br>Suite 110<br>Columbia, MD 21046	Sharon Lynn Nelson, MSN, RN, CNS<br><br>Sara Harnish, J.D	32216 / Goldberg, Ronald	Protocol Version 3.0 dated 19/JUL/2011 and Local Amendment dated 17/FEB/2012<br><br>Protocol Version 4.0 dated 07/JUN/2013 (incorporating Global Amendment 1 dated 21/MAY/2013)	15/APR/2013<br><br>03/JUL/2013

United States of America				
**IRB or IEC (name/address)**	**IRB or IEC Chairperson**	**Centre number (5 digit number) / Investigator(s)**	**Protocol and/or Amendment number(s)**	Date of Final Approval (DD/MMM/YYYY)
Trinity Hospitals IRB<br>One Burdick Expressway West<br>East Minot, ND 58701	Steve Mattson, MD	32220 / Turk, Samir	Protocol Version 3.0 dated 19/JUL/2011 and Local Amendment dated 17/FEB/2012<br><br>Protocol Version 4.0 dated 07/JUN/2013 (incorporating Global Amendment 1 dated 21/MAY/2013)	10/SEP/2012<br><br>11/NOV/2013
The Valley Hospital Health System IRB<br>223 North Van Diem Avenue<br>Ridgewood, NJ 07450	David Montgomery	32221 /Williams, Marcus	Protocol Version 3.0 dated 19/JUL/2011 and Local Amendment dated 17/FEB/2012	22/AUG/2012

United States of America				
**IRB or IEC (name/address)**	**IRB or IEC Chairperson**	**Centre number (5 digit number) / Investigator(s)**	**Protocol and/or Amendment number(s)**	Date of Final Approval (DD/MMM/YYYY)
Western Institutional Review Board<br>1019 39th Avenue SE, Suite 120 Puyallup, WA 98374-2115	Bert Wilkins		Protocol Version 3.0 dated 19/JUL/2011 and Local Amendment dated 17/FEB/2012<br><br>Protocol Version 4.0 dated 07/JUN/2013 (incorporating Global Amendment 1 dated 21/MAY/2013)	07/JAN/2014<br><br>07/JAN/2014
Memorial Hospital Of Rhode Island<br>Institutional Review Board<br>45 Willard Ave, Providence, RI 02905	Patrick Weyer	32224 / Eaton, Charles	Protocol Version 3.0 dated 19/JUL/2011 and Local Amendment dated 17/FEB/2012<br><br>Protocol Version 4.0 dated 07/JUN/2013 (incorporating Global Amendment 1 dated 21/MAY/2013)	04/SEP/2012<br><br>03/SEP/2013

United States of America				
**IRB or IEC (name/address)**	**IRB or IEC Chairperson**	**Centre number (5 digit number) / Investigator(s)**	**Protocol and/or Amendment number(s)**	Date of Final Approval (DD/MMM/YYYY)
Schulman Associates Institutional Review Board, Inc.<br>4445 Lake Forest Drive Suite 300<br>Cincinnati, Ohio 45242<br><br>ADVARRA<br>6940 Columbia Gateway Drive,<br>Suite 110<br>Columbia, MD 21046	Sharon Lynn Nelson, MSN, RN, CNS<br><br>Sara Harnish, J.D	32227 / Frandsen, Brad	Protocol Version 3.0 dated 19/JUL/2011 and Local Amendment dated 17/FEB/2012<br><br>Protocol Version 4.0 dated 07/JUN/2013 (incorporating Global Amendment 1 dated 21/MAY/2013)	28/SEP/2012<br><br>03/JUL/2013
Covenant Medical Center Institutional Review Board<br>1447 North Harrison<br>Saginaw, MI 48602	Dennis Boysen	32231 / Jundi, Mayar	Protocol Version 3.0 dated 19/JUL/2011 and Local Amendment dated 17/FEB/2012<br><br>Protocol Version 4.0 dated 07/JUN/2013 (incorporating Global	19/SEP/2012<br><br>18/SEP/2013

United States of America				
**IRB or IEC (name/address)**	**IRB or IEC Chairperson**	**Centre number (5 digit number) / Investigator(s)**	**Protocol and/or Amendment number(s)**	Date of Final Approval (DD/MMM/YYYY)
			Amendment 1 dated 21/MAY/2013)	
McLaren HealthCare Human Research Protections Program IRB<br>1198 N. Belsay Road<br>Bldg #1<br>Burton, MI 48509	Ammar Hatahet	32232 / Kazmierski, John	Protocol Version 3.0 dated 19/JUL/2011 and Local Amendment dated 17/FEB/2012<br><br>Protocol Version 4.0 dated 07/JUN/2013 (incorporating Global Amendment 1 dated 21/MAY/2013)	15/OCT/2012<br><br>03/SEP/2013
Schulman Associates Institutional Review Board, Inc.<br>4445 Lake Forest Drive Suite 300	Sharon Lynn Nelson, MSN, RN, CNS	32233 / Landau, Charles	Protocol Version 3.0 dated 19/JUL/2011 and Local Amendment dated 17/FEB/2012	16/OCT/2012

United States of America				
**IRB or IEC (name/address)**	**IRB or IEC Chairperson**	**Centre number (5 digit number) / Investigator(s)**	**Protocol and/or Amendment number(s)**	Date of Final Approval (DD/MMM/YYYY)
Cincinnati, Ohio 45242<br><br>ADVARRA<br>6940 Columbia Gateway Drive,<br>Suite 110<br>Columbia, MD 21046	Sara Harnish, J.D		Protocol Version 4.0 dated 07/JUN/2013 (incorporating Global Amendment 1 dated 21/MAY/2013)	03/JUL/2013
Schulman Associates Institutional Review Board, Inc.<br>4445 Lake Forest Drive Suite 300<br>Cincinnati, Ohio 45242<br><br>ADVARRA<br>6940 Columbia Gateway Drive,<br>Suite 110	Sharon Lynn Nelson, MSN, RN, CNS<br><br>Sara Harnish, J.D	32235 / Mulhearn, Thomas	Protocol Version 3.0 dated 19/JUL/2011 and Local Amendment dated 17/FEB/2012<br><br>Protocol Version 4.0 dated 07/JUN/2013 (incorporating Global Amendment 1 dated	30/AUG/2012<br><br>03/JUL/2013

United States of America				
**IRB or IEC (name/address)**	**IRB or IEC Chairperson**	**Centre number (5 digit number) / Investigator(s)**	**Protocol and/or Amendment number(s)**	Date of Final Approval (DD/MMM/YYYY)
Columbia, MD 21046			21/MAY/2013)	
Schulman Associates Institutional Review Board, Inc.<br>4445 Lake Forest Drive Suite 300<br>Cincinnati, Ohio 45242<br>ADVARRA<br>6940 Columbia Gateway Drive,<br>Suite 110<br>Columbia, MD 21046	Sharon Lynn Nelson, MSN, RN, CNS<br><br>Sara Harnish, J.D	32236 / Nadar, Venatesh	Protocol Version 3.0 dated 19/JUL/2011 and Local Amendment dated 17/FEB/2012<br><br>Protocol Version 4.0 dated 07/JUN/2013 (incorporating Global Amendment 1 dated 21/MAY/2013))	27/SEP/2012<br><br>03/JUL/2013
Baptist Hospital IRB<br>1000 West Moreno Street<br>PO Box 17500<br>Pensacola, FL 32522	N/A	32239 / Giedrimas, Evaldas	Protocol Version 3.0 dated 19/JUL/2011 and Local Amendment dated 17/FEB/2012<br><br>Protocol Version 4.0	04/OCT/2012

United States of America				
**IRB or IEC (name/address)**	**IRB or IEC Chairperson**	**Centre number (5 digit number) / Investigator(s)**	**Protocol and/or Amendment number(s)**	Date of Final Approval (DD/MMM/YYYY)
			dated 07/JUN/2013 (incorporating Global Amendment 1 dated 21/MAY/2013)	01/AUG/2013
Schulman Associates Institutional Review Board, Inc.<br>4445 Lake Forest Drive Suite 300<br>Cincinnati, Ohio 45242<br><br>ADVARRA<br>6940 Columbia Gateway Drive,<br>Suite 110<br>Columbia, MD 21046 USA	Sharon Lynn Nelson, MSN, RN, CNS<br><br>Sara Harnish, J.D	32241 / Huehnergarth, Kier	Protocol Version 3.0 dated 19/JUL/2011 and Local Amendment dated 17/FEB/2012<br><br>Protocol Version 4.0 dated 07/JUN/2013 (incorporating Global Amendment 1 dated 21/MAY/2013)	20/SEP/2012<br><br>03/JUL/2013
Schulman Associates Institutional Review	Sharon Lynn Nelson, MSN, RN, CNS	32242 / Zukerman, Steven	Protocol Version 3.0 dated 19/JUL/2011 and Local Amendment dated	13/SEP/2012

United States of America				
**IRB or IEC (name/address)**	**IRB or IEC Chairperson**	**Centre number (5 digit number) / Investigator(s)**	**Protocol and/or Amendment number(s)**	Date of Final Approval (DD/MMM/YYYY)
Board, Inc.<br>4445 Lake Forest Drive Suite 300<br>Cincinnati, Ohio 45242<br><br>ADVARRA<br>6940 Columbia Gateway Drive,<br>Suite 110<br>Columbia, MD 21046	Sara Harnish, J.D		17/FEB/2012<br><br>Protocol Version 4.0 dated 07/JUN/2013 (incorporating Global Amendment 1 dated 21/MAY/2013)	03/JUL/2013
AnMed Health IRB<br>800 North Fant Street<br>Anderson, SC 29621	Elaine Epstein	32243 / McLaurin, Brent	Protocol Version 3.0 dated 19/JUL/2011 and Local Amendment dated 17/FEB/2012<br><br>Protocol Version 4.0 dated 07/JUN/2013	20/SEP/2012<br><br>15/AUG/2013

United States of America				
**IRB or IEC (name/address)**	**IRB or IEC Chairperson**	**Centre number (5 digit number) / Investigator(s)**	**Protocol and/or Amendment number(s)**	Date of Final Approval (DD/MMM/YYYY)
			(incorporating Global Amendment 1 dated 21/MAY/2013)	
Schulman Associates Institutional Review Board, Inc.<br>4445 Lake Forest Drive Suite 300<br>Cincinnati, Ohio 45242<br><br>ADVARRA<br>6940 Columbia Gateway Drive,<br>Suite 110<br>Columbia, MD 21046	Sharon Lynn Nelson, MSN, RN, CNS<br><br>Sara Harnish, J.D	32244 / Mohammed, Akber	Protocol Version 3.0 dated 19/JUL/2011 and Local Amendment dated 17/FEB/2012<br><br>Protocol Version 4.0 dated 07/JUN/2013 (incorporating Global Amendment 1 dated 21/MAY/2013)	24/OCT/2012<br><br>03/JUL/2013
St. Vincent Hospital IRB<br>8402 Harcourt Rd.<br>Suite 806<br>Indianapolis, IN 46260	Tina Noonan	32245 / Padanilam, Benzy	Protocol Version 3.0 dated 19/JUL/2011 and Local Amendment dated 17/FEB/2012	06/SEP/2012

United States of America				
**IRB or IEC (name/address)**	**IRB or IEC Chairperson**	**Centre number (5 digit number) / Investigator(s)**	**Protocol and/or Amendment number(s)**	Date of Final Approval (DD/MMM/YYYY)
			Protocol Version 4.0 dated 07/JUN/2013 (incorporating Global Amendment 1 dated 21/MAY/2013)	18/SEP/2013
New York-Presbyterian Brooklyn Methodist Hospital<br>Institutional Review Committee<br>506 Sixth St.<br>Box 159008<br>Brooklyn, NY 11215-9008	Eric Balmir	32246 / Wong, Brian	Protocol Version 3.0 dated 19/JUL/2011 and Local Amendment dated 17/FEB/2012<br>Protocol Version 4.0 dated 07/JUN/2013 (incorporating Global Amendment 1 dated 21/MAY/2013)	28/AUG/2012<br>21/AUG/2013

United States of America				
**IRB or IEC (name/address)**	**IRB or IEC Chairperson**	**Centre number (5 digit number) / Investigator(s)**	**Protocol and/or Amendment number(s)**	Date of Final Approval (DD/MMM/YYYY)
Schulman Associates Institutional Review Board, Inc.<br>4445 Lake Forest Drive Suite 300<br>Cincinnati, Ohio 45242<br><br>ADVARRA<br>6940 Columbia Gateway Drive,<br>Suite 110<br>Columbia, MD 21046	Sharon Lynn Nelson, MSN, RN, CNS<br><br>Sara Harnish, J.D	32248 / Culp, John	Protocol Version 3.0 dated 19/JUL/2011 and Local Amendment dated 17/FEB/2012<br><br>Protocol Version 4.0 dated 07/JUN/2013 (incorporating Global Amendment 1 dated 21/MAY/2013)	24/OCT/2012<br><br>03/JUL/2013
Carolinas HealthCare System IRB<br>P.O. Box 32861<br>Charlotte, NC 28232	Michael Brennan	32251 / Thomas, Harold	Protocol Version 3.0 dated 19/JUL/2011 and Local Amendment dated 17/FEB/2012	11/JUL/2012

United States of America				
**IRB or IEC (name/address)**	**IRB or IEC Chairperson**	**Centre number (5 digit number) / Investigator(s)**	**Protocol and/or Amendment number(s)**	Date of Final Approval (DD/MMM/YYYY)
UT South Western IRB<br>5323 Harry Hines Blvd.<br>Dallas, TX 75390	David Karp	32252 / Banerjee, Subhas	Protocol Version 3.0 dated 19-Jul-2011 and Local Amendment 1 dated 17-Feb-2012<br><br>Protocol Version 4.0 dated 07/JUN/2013 (incorporating Global Amendment 1 dated 21/MAY/2013)	24/JUL/2012<br><br>18/JAN/2014
St. Joseph's Health Care System IRB<br>Xavier Building<br>Administration 1<br>Room 1406<br>Peterson, NJ 07503	Patrick V. Perin, MD	32255 / Wenchi, Tsai	Protocol Version 3.0 dated 19/JUL/2011 and Local Amendment dated 17/FEB/2012<br><br>Protocol Version 4.0 dated 07/JUN/2013 (incorporating Global	04/OCT/2012<br><br>05/SEP/2013

United States of America				
**IRB or IEC (name/address)**	**IRB or IEC Chairperson**	**Centre number (5 digit number) / Investigator(s)**	**Protocol and/or Amendment number(s)**	Date of Final Approval (DD/MMM/YYYY)
			Amendment 1 dated 21/MAY/2013)	
Schulman Associates Institutional Review Board, Inc.<br>4445 Lake Forest Drive Suite 300<br>Cincinnati, Ohio 45242<br><br>INTEGRIS Health Inc., IRB<br>3400 NW Expressway<br>Building C, Suite 806<br>Oklahoma City, OK 73112	Sharon Lynn Nelson, MSN, RN, CNS<br><br>R.C. Brown, MD	32258 / Friese, Athena	Protocol Version 3.0 dated 19/JUL/2011 and Local Amendment dated 17/FEB/2012<br><br>Protocol Version 4.0 dated 07/JUN/2013 (incorporating Global Amendment 1 dated 21/MAY/2013)	08/JUN/2012<br><br>20/JAN/2015
Schulman Associates Institutional Review Board, Inc.<br>4445 Lake Forest Drive	Sharon Lynn Nelson, MSN, RN, CNS	32259 / Sheikh, Khalid<br>,	Protocol Version 3.0 dated 19/JUL/2011 and Local Amendment dated 17/FEB/2012	13/NOV/2012

United States of America				
**IRB or IEC (name/address)**	**IRB or IEC Chairperson**	**Centre number (5 digit number) / Investigator(s)**	**Protocol and/or Amendment number(s)**	Date of Final Approval (DD/MMM/YYYY)
Suite 300<br>Cincinnati, Ohio 45242<br><br>ADVARRA<br>6940 Columbia Gateway Drive,<br>Suite 110<br>Columbia, MD 21046	 <br><br>Sara Harnish, J.D		Protocol Version 4.0 dated 07/JUN/2013 (incorporating Global Amendment 1 dated 21/MAY/2013)	03/JUL/2013
Schulman Associates Institutional Review Board, Inc.<br>4445 Lake Forest Drive Suite 300<br>Cincinnati, Ohio 45242<br><br>ADVARRA<br>6940 Columbia Gateway Drive,	Sharon Lynn Nelson, MSN, RN, CNS<br><br><br>Sara Harnish, J.D	32263 / Brautigam, Donald	Protocol Version 3.0 dated 19/JUL/2011 and Local Amendment dated 17/FEB/2012<br><br>Protocol Version 4.0 dated 07/JUN/2013 (incorporating Global	30/OCT/2012<br><br><br>03/JUL/2013

United States of America				
**IRB or IEC (name/address)**	**IRB or IEC Chairperson**	**Centre number (5 digit number) / Investigator(s)**	**Protocol and/or Amendment number(s)**	Date of Final Approval (DD/MMM/YYYY)
Suite 110<br>Columbia, MD 21046			Amendment 1 dated 21/MAY/2013)	
Genesys Health System<br>One Genesys Parkway<br>Grand Blanc, MI 48439-8066	Mark Vogel, PhD	32264 / DuThinh, Vuong	Protocol Version 3.0 dated 19/JUL/2011 and Local Amendment dated 17/FEB/2012<br><br>Protocol Version 4.0 dated 07/JUN/2013 (incorporating Global Amendment 1 dated 21/MAY/2013)	12/NOV/2012<br><br>14/AUG/2013
Schulman Associates Institutional Review Board, Inc.<br>4445 Lake Forest Drive Suite 300<br>Cincinnati, Ohio 45242	Sharon Lynn Nelson, MSN, RN, CNS	32265 / Paraschos, Alexander	Protocol Version 3.0 dated 19/JUL/2011 and Local Amendment dated 17/FEB/2012	29/NOV/2012

United States of America				
**IRB or IEC (name/address)**	**IRB or IEC Chairperson**	**Centre number (5 digit number) / Investigator(s)**	**Protocol and/or Amendment number(s)**	Date of Final Approval (DD/MMM/YYYY)
ADVARRA<br>6940 Columbia Gateway Drive,<br>Suite 110<br>Columbia, MD 21046	Sara Harnish, J.D		Protocol Version 4.0 dated 07/JUN/2013 (incorporating Global Amendment 1 dated 21/MAY/2013)	03/JUL/2013
Sparks Health System<br>1001 Towson Ave<br>Fort Smith, AR 72902	Samara Criswell	32266 / Parris, Robert	Protocol Version 3.0 dated 19/JUL/2011 and Local Amendment dated 17/FEB/2012<br><br>Protocol Version 4.0 dated 07/JUN/2013 (incorporating Global Amendment 1 dated 21/MAY/2013)	14/SEP/2012<br><br>09/AUG/2013

United States of America				
**IRB or IEC (name/address)**	**IRB or IEC Chairperson**	**Centre number (5 digit number) / Investigator(s)**	**Protocol and/or Amendment number(s)**	Date of Final Approval (DD/MMM/YYYY)
Schulman Associates Institutional Review Board, Inc.<br>4445 Lake Forest Drive Suite 300<br>Cincinnati, Ohio 45242<br><br>ADVARRA<br>6940 Columbia Gateway Drive,<br>Suite 110<br>Columbia, MD 21046	Sharon Lynn Nelson, MSN, RN, CNS<br><br>Sara Harnish, J.D	32267 / Teixeira, Jose	Protocol Version 3.0 dated 19/JUL/2011 and Local Amendment dated 17/FEB/2012<br><br>Protocol Version 4.0 dated 07/JUN/2013 (incorporating Global Amendment 1 dated 21/MAY/2013)	12/OCT/2012<br><br>03/JUL/2013
Bridgeport Hospital IRB<br>Yale New Haven Health<br>267 Grant Street<br>Bridgeport, CT 06610	Karen A. Hutchinson, MD	32268 / Zarich, Stuart	Protocol Version 3.0 dated 19/JUL/2011 and Local Amendment dated 17/FEB/2012<br>Protocol Version 4.0 dated 07/JUN/2013 (incorporating Global	19/JUL/2012<br><br>05/SEP/2013

United States of America				
**IRB or IEC (name/address)**	**IRB or IEC Chairperson**	**Centre number (5 digit number) / Investigator(s)**	**Protocol and/or Amendment number(s)**	Date of Final Approval (DD/MMM/YYYY)
			Amendment 1 dated 21/MAY/2013)	
Schulman Associates Institutional Review Board, Inc.<br>4445 Lake Forest Drive Suite 300<br>Cincinnati, Ohio 45242<br><br>ADVARRA<br>6940 Columbia Gateway Drive,<br>Suite 110<br>Columbia, MD 21046	Sharon Lynn Nelson, MSN, RN, CNS<br><br>Sara Harnish, J.D	32269 / Roelke, Marc	Protocol Version 3.0 dated 19/JUL/2011 and Local Amendment dated 17/FEB/2012<br><br>Protocol Version 4.0 dated 07/JUN/2013 (incorporating Global Amendment 1 dated 21/MAY/2013)	10/DEC/2012<br><br>03/JUL/2013
Schulman Associates Institutional Review Board, Inc.	Sharon Lynn Nelson, MSN, RN, CNS	32270 / Ackermann, Alan	Protocol Version 3.0 dated 19/JUL/2011 and Local Amendment dated	12/DEC/2012

United States of America				
**IRB or IEC (name/address)**	**IRB or IEC Chairperson**	**Centre number (5 digit number) / Investigator(s)**	**Protocol and/or Amendment number(s)**	Date of Final Approval (DD/MMM/YYYY)
4445 Lake Forest Drive Suite 300<br>Cincinnati, Ohio 45242<br><br>ADVARRA<br>6940 Columbia Gateway Drive,<br>Suite 110<br>Columbia, MD 21046	Sara Harnish, J.D		17/FEB/2012<br><br>Protocol Version 4.0 dated 07/JUN/2013 (incorporating Global Amendment 1 dated 21/MAY/2013)	03/JUL/2013
Schulman Associates Institutional Review Board, Inc.<br>4445 Lake Forest Drive Suite 300<br>Cincinnati, Ohio 45242<br><br>ADVARRA<br>6940 Columbia Gateway Drive,	Sharon Lynn Nelson, MSN, RN, CNS<br><br>Sara Harnish, J.D	32271 / Jeanfreau, Robert	Protocol Version 3.0 dated 19/JUL/2011 and Local Amendment dated 17/FEB/2012<br><br>Protocol Version 4.0 dated 07/JUN/2013 (incorporating Global	26/NOV/2012<br><br>03/JUL/2013

United States of America				
**IRB or IEC (name/address)**	**IRB or IEC Chairperson**	**Centre number (5 digit number) / Investigator(s)**	**Protocol and/or Amendment number(s)**	Date of Final Approval (DD/MMM/YYYY)
Suite 110<br>Columbia, MD 21046			Amendment 1 dated 21/MAY/2013)	
MetroHealth Institutional Review Board<br>2500 MetroHealth Dr.<br>Cleveland, OH 44109	David Kuentz	32273 / Kaufman, Elizabeth	Protocol Version 3.0 dated 19/JUL/2011 and Local Amendment dated 17/FEB/2012<br>Protocol Version 4.0 dated 07/JUN/2013 (incorporating Global Amendment 1 dated 21/MAY/2013)	27/SEP/2012<br>14/AUG/2013
LSU Health Sciences Center IRB<br>433 Bolivar St.<br>Suite 206<br>New Orleans, LA 70112	Richard Tejedor, MD	32274 / Ahmed, Jameel	Protocol Version 3.0 dated 19/JUL/2011 and Local Amendment dated 17/FEB/2012<br>Protocol Version 4.0 dated 07/JUN/2013 (incorporating Global Amendment 1 dated 21/MAY/2013)	21/SEP/2012<br>31/OCT/2013

United States of America				
**IRB or IEC (name/address)**	**IRB or IEC Chairperson**	**Centre number (5 digit number) / Investigator(s)**	**Protocol and/or Amendment number(s)**	Date of Final Approval (DD/MMM/YYYY)
Western Institutional Review Board<br>1019 39th Avenue SE, Suite 120 Puyallup, WA 98374-2115	Bert Wilkins	32277 / Labovitz, Arthur	Protocol Version 3.0 dated 19/JUL/2011 and Local Amendment dated 17/FEB/2012<br><br>Protocol Version 4.0 dated 07/JUN/2013 (incorporating Global Amendment 1 dated 21/MAY/2013)	07/DEC/2012<br><br>03/SEP/2013
United Health Services Hospitals<br>33-57 Harrison St. Johnson City NY<br>United Health Svcs Hospitals IRB<br>33-57 Harrison Street<br>Johnson City, NY 13790	Michael Chisdak, MD	32278 / Kashou, Hisman	Protocol Version 3.0 dated 19/JUL/2011 and Local Amendment dated 17/FEB/2012<br><br>Protocol Version 4.0 dated 07/JUN/2013 (incorporating Global Amendment 1 dated 21/MAY/2013)	19/SEP/2012<br><br>09/SEP/2013

United States of America				
**IRB or IEC (name/address)**	**IRB or IEC Chairperson**	**Centre number (5 digit number) / Investigator(s)**	**Protocol and/or Amendment number(s)**	Date of Final Approval (DD/MMM/YYYY)
Schulman Associates Institutional Review Board, Inc.<br>4445 Lake Forest Drive Suite 300<br>Cincinnati, Ohio 45242<br><br>ADVARRA<br>6940 Columbia Gateway Drive,<br>Suite 110<br>Columbia, MD 21046	Sharon Lynn Nelson, MSN, RN, CNS<br><br>Sara Harnish, J.D	32279 / Kapadia, Shaival	Protocol Version 3.0 dated 19/JUL/2011 and Local Amendment dated 17/FEB/2012<br><br>Protocol Version 4.0 dated 07/JUN/2013 (incorporating Global Amendment 1 dated 21/MAY/2013)	10/OCT/2012<br><br>03/JUL/2013
Schulman Associates Institutional Review Board, Inc.<br>4445 Lake Forest Drive Suite 300<br>Cincinnati, Ohio 45242	Sharon Lynn Nelson, MSN, RN, CNS	32280 / Gornick, Charles	Protocol Version 3.0 dated 19/JUL/2011 and Local Amendment dated 17/FEB/2012	14/OCT/2013

United States of America				
**IRB or IEC (name/address)**	**IRB or IEC Chairperson**	**Centre number (5 digit number) / Investigator(s)**	**Protocol and/or Amendment number(s)**	Date of Final Approval (DD/MMM/YYYY)
ADVARRA<br>6940 Columbia Gateway Drive,<br>Suite 110<br>Columbia, MD 21046	Sara Harnish, J.D		Protocol Version 4.0 dated 07/JUN/2013 (incorporating Global Amendment 1 dated 21/MAY/2013)	14/OCT/2013
Schulman Associates Institutional Review Board, Inc.<br>4445 Lake Forest Drive Suite 300<br>Cincinnati, Ohio 45242<br><br>ADVARRA<br>6940 Columbia Gateway Drive,<br>Suite 110<br>Columbia, MD 21046 USA	Sharon Lynn Nelson, MSN, RN, CNS<br><br>Sara Harnish, J.D	32283 / Lupovitch, Steven	Protocol Version 3.0 dated 19/JUL/2011 and Local Amendment dated 17/FEB/2012<br><br>Protocol Version 4.0 dated 07/JUN/2013 (incorporating Global Amendment 1 dated 21/MAY/2013)	11/DEC/2012<br><br>03/JUL/2013

United States of America				
**IRB or IEC (name/address)**	**IRB or IEC Chairperson**	**Centre number (5 digit number) / Investigator(s)**	**Protocol and/or Amendment number(s)**	Date of Final Approval (DD/MMM/YYYY)
Mercy Health North LLC Adult IRB<br>2200 Jefferson Ave<br>Toledo, OH 43604	Pamela Oatis, MD	32285 / Kabour, Ameer	Protocol Version 3.0 dated 19/JUL/2011 and Local Amendment dated 17/FEB/2012<br><br>Protocol Version 4.0 dated 07/JUN/2013 (incorporating Global Amendment 1 dated 21/MAY/2013)	10/DEC/2012<br><br>09/SEP/2013
Western Institutional Review Board<br>3535 7th Avenue SW,<br>Olympia, WA 98374-2115	Theodore D. Schultz	32286 / Deumite, Joseph	Protocol Version 3.0 dated 19/JUL/2011 and Local Amendment dated 17/FEB/2012<br><br>Protocol Version 4.0 dated 07/JUN/2013 (incorporating Global Amendment 1 dated	02/OCT/2012<br><br>16/AUG/2013

United States of America				
**IRB or IEC (name/address)**	**IRB or IEC Chairperson**	**Centre number (5 digit number) / Investigator(s)**	**Protocol and/or Amendment number(s)**	Date of Final Approval (DD/MMM/YYYY)
			21/MAY/2013)	
Schulman Associates Institutional Review Board, Inc.<br>4445 Lake Forest Drive Suite 300<br>Cincinnati, Ohio 45242<br><br>ADVARRA<br>6940 Columbia Gateway Drive,<br>Suite 110<br>Columbia, MD 21046	Sharon Lynn Nelson, MSN, RN, CNS<br><br>Sara Harnish, J.D	32287 / Foster, Malcom	Protocol Version 3.0 dated 19/JUL/2011 and Local Amendment dated 17/FEB/2012<br><br>Protocol Version 4.0 dated 07/JUN/2013 (incorporating Global Amendment 1 dated 21/MAY/2013)	05/JUL/2012<br><br>03/JUL/2013
Schulman Associates Institutional Review Board, Inc.<br>4445 Lake Forest Drive Suite 300	Sharon Lynn Nelson, MSN, RN, CNS	32288 / Krishnaswamy, Kannappan	Protocol Version 3.0 dated 19/JUL/2011 and Local Amendment dated 17/FEB/2012	23/JAN/2013

United States of America				
**IRB or IEC (name/address)**	**IRB or IEC Chairperson**	**Centre number (5 digit number) / Investigator(s)**	**Protocol and/or Amendment number(s)**	Date of Final Approval (DD/MMM/YYYY)
Cincinnati, Ohio 45242<br><br>ADVARRA<br>6940 Columbia Gateway Drive,<br>Suite 110<br>Columbia, MD 21046	Sara Harnish, J.D		Protocol Version 4.0 dated 07/JUN/2013 (incorporating Global Amendment 1 dated 21/MAY/2013)	03/JUL/2013
Newton Wellesley Hospital IRB<br>214 Washington Street<br>Newton, MA 02462	Michael Jellinek	32289 / Huckins, David	Protocol Version 3.0 dated 19/JUL/2011 and Local Amendment dated 17/FEB/2012<br><br>Protocol Version 4.0 dated 07/JUN/2013 (incorporating Global Amendment 1 dated 21/MAY/2013)	25/OCT/2012<br><br>11/SEP/2013
Edward Hines, JR. VAH/JALFHCC IRB	Yvonne Lucero, MD	32293 / Rohit, Aurora	Protocol Version 3.0 dated 19/JUL/2011 and Local	27/DEC/2012

United States of America				
**IRB or IEC (name/address)**	**IRB or IEC Chairperson**	**Centre number (5 digit number) / Investigator(s)**	**Protocol and/or Amendment number(s)**	Date of Final Approval (DD/MMM/YYYY)
PO Box 5000 (#151)<br>Hines, IL 60141			Amendment dated 17/FEB/2012<br><br>Protocol Version 4.0 dated 07/JUN/2013 (incorporating Global Amendment 1 dated 21/MAY/2013)	 <br>06/SEP/2013
St. Louis University IRB<br>3556 Caroline Street<br>Room C110<br>St. Louis, MO 63104	Oleg Kisselev, PhD	32297 / Alderson, Lisa	Protocol Version 3.0 dated 19/JUL/2011 and Local Amendment dated 17/FEB/2012<br><br>Protocol Version 4.0 dated 07/JUN/2013 (incorporating Global Amendment 1 dated 21/MAY/2013)	06/JUN/2012<br><br>07/AUG/2013

United States of America				
**IRB or IEC (name/address)**	**IRB or IEC Chairperson**	**Centre number (5 digit number) / Investigator(s)**	**Protocol and/or Amendment number(s)**	Date of Final Approval (DD/MMM/YYYY)
University of Chicago IRB<br>Division of Biological Sciences Division<br>5841 S. Maryland Ave.<br>Chicago, IL 60637	Christopher Daugherty, MD	32298 / Nayak, Hemal	Protocol Version 3.0 dated 19/JUL/2011 and Local Amendment dated 17/FEB/2012<br><br>Protocol Version 4.0 dated 07/JUN/2013 (incorporating Global Amendment 1 dated 21/MAY/2013)	26/NOV/2012<br><br>09/JUN/2014
CIRBI<br>7063 Columbia Gateway Dr.<br>Suite 110<br>Columbia, MD 21046	Joy Cavagnaro, PhD	32299 / Rashba, Eric	Protocol Version 3.0 dated 19/JUL/2011 and Local Amendment dated 17/FEB/2012<br><br>Protocol Version 4.0 dated 07/JUN/2013 (incorporating Global Amendment 1 dated 21/MAY/2013)	06/DEC/2012<br><br>12/NOV/2013

United States of America				
**IRB or IEC (name/address)**	**IRB or IEC Chairperson**	**Centre number (5 digit number) / Investigator(s)**	**Protocol and/or Amendment number(s)**	Date of Final Approval (DD/MMM/YYYY)
The Methodist Hospital Research Institute IRB<br>6670 Bertner<br>Houston, TX 77030	Susan Miller, MD	32301 / Kurrelmeyer, Karla	Protocol Version 3.0 dated 19/JUL/2011 and Local Amendment dated 17/FEB/2012<br><br>Protocol Version 4.0 dated 07/JUN/2013 (incorporating Global Amendment 1 dated 21/MAY/2013)	30/JUL/2012<br><br>18/SEP/2013
Emory St. Joseph's Hospital IRB<br>5665 Peachtree Dunwoody Rd.<br>Suite G7<br>Atlanta, GA 30342	Alexander Park, MD	32302 / Eisenberg, Steven	Protocol Version 3.0 dated 19/JUL/2011 and Local Amendment dated 17/FEB/2012<br>Protocol Version 4.0 dated 07/JUN/2013 (incorporating Global Amendment 1 dated 21/MAY/2013)	25/JUL/2012<br><br>25/SEP/2013

United States of America				
**IRB or IEC (name/address)**	**IRB or IEC Chairperson**	**Centre number (5 digit number) / Investigator(s)**	**Protocol and/or Amendment number(s)**	Date of Final Approval (DD/MMM/YYYY)
Schulman Associates Institutional Review Board, Inc.<br>4445 Lake Forest Drive Suite 300<br>Cincinnati, Ohio 45242<br><br>ADVARRA<br>6940 Columbia Gateway Drive,<br>Suite 110<br>Columbia, MD 21046	Sharon Lynn Nelson, MSN, RN, CNS<br><br>Sara Harnish, J.D	32303 / Augenbraun, Charles	Protocol Version 3.0 dated 19/JUL/2011 and Local Amendment dated 17/FEB/2012<br><br>Protocol Version 4.0 dated 07/JUN/2013 (incorporating Global Amendment 1 dated 21/MAY/2013)	06/FEB/2013<br><br>03/JUL/2013
UTHSC at Tyler IRB<br>11937 US HWY 271<br>Tyler, TX 75708	David Pearson, Ph.D.	32306 / Goulden, Dudley	Protocol Version 3.0 dated 19/JUL/2011 and Local Amendment dated 17/FEB/2012	14/NOV/2012

United States of America				
**IRB or IEC (name/address)**	**IRB or IEC Chairperson**	**Centre number (5 digit number) / Investigator(s)**	**Protocol and/or Amendment number(s)**	Date of Final Approval (DD/MMM/YYYY)
			Protocol Version 4.0 dated 07/JUN/2013 (incorporating Global Amendment 1 dated 21/MAY/2013)	22/AUG/2013
Schulman Associates Institutional Review Board, Inc.<br>4445 Lake Forest Drive Suite 300<br>Cincinnati, Ohio 45242<br><br>ADVARRA<br>6940 Columbia Gateway Drive,<br>Suite 110<br>Columbia, MD 21046	Sharon Lynn Nelson, MSN, RN, CNS<br><br>Sara Harnish, J.D	32307 / Bank, Alan	Protocol Version 3.0 dated 19/JUL/2011 and Local Amendment dated 17/FEB/2012<br><br>Protocol Version 4.0 dated 07/JUN/2013 (incorporating Global Amendment 1 dated 21/MAY/2013)	14/OCT/2013<br><br>14/OCT/2013

United States of America				
**IRB or IEC (name/address)**	**IRB or IEC Chairperson**	**Centre number (5 digit number) / Investigator(s)**	**Protocol and/or Amendment number(s)**	Date of Final Approval (DD/MMM/YYYY)
Mayo Clinic Institutional Review Board<br>200 First St SW, Rochester, MN 55905<br>Mayo Clinic IRB<br>700 West Avenue South<br>La Crosse, WI 54601	Joseph Lobl	32308 / Del-Carpio, Munoz, Freddy	Protocol Version 3.0 dated 19/JUL/2011 and Local Amendment dated 17/FEB/2012<br><br>Protocol Version 4.0 dated 07/JUN/2013 (incorporating Global Amendment 1 dated 21/MAY/2013)	18/FEB/2013<br><br>06/DEC/2013
Schulman Associates Institutional Review Board, Inc.<br>4445 Lake Forest Drive Suite 300<br>Cincinnati, Ohio 45242<br>ADVARRA<br>6940 Columbia Gateway Drive,<br>Suite 110	Sharon Lynn Nelson, MSN, RN, CNS<br><br>Sara Harnish, J.D	32312 / Jimenez, Javier	Protocol Version 3.0 dated 19/JUL/2011 and Local Amendment dated 17/FEB/2012<br><br>Protocol Version 4.0 dated 07/JUN/2013 (incorporating Global Amendment 1 dated 21/MAY/2013)	22/JAN/2013<br><br>03/JUL/2013

United States of America				
**IRB or IEC (name/address)**	**IRB or IEC Chairperson**	**Centre number (5 digit number) / Investigator(s)**	**Protocol and/or Amendment number(s)**	Date of Final Approval (DD/MMM/YYYY)
Columbia, MD 21046				
Schulman Associates Institutional Review Board, Inc.<br>4445 Lake Forest Drive Suite 300<br>Cincinnati, Ohio 45242<br><br>ADVARRA<br>6940 Columbia Gateway Drive,<br>Suite 110<br>Columbia, MD 21046	Sharon Lynn Nelson, MSN, RN, CNS<br><br>Sara Harnish, J.D	32313 / Puleo, John	Protocol Version 3.0 dated 19/JUL/2011 and Local Amendment dated 17/FEB/2012<br><br>Protocol Version 4.0 dated 07/JUN/2013 (incorporating Global Amendment 1 dated 21/MAY/2013)	05/FEB/2013<br><br>03/JUL/2013
Western Institutional Review Board<br>1019 39th Avenue SE<br>Suite 120	Bert Wilkins	32314 / Cynthia, Tracy	Protocol Version 3.0 dated 19/JUL/2011 and Local Amendment dated 17/FEB/2012	18/JAN/2013

United States of America				
**IRB or IEC (name/address)**	**IRB or IEC Chairperson**	**Centre number (5 digit number) / Investigator(s)**	**Protocol and/or Amendment number(s)**	Date of Final Approval (DD/MMM/YYYY)
Puyallup, WA 98374-2115			Protocol Version 4.0 dated 07/JUN/2013 (incorporating Global Amendment 1 dated 21/MAY/2013)	26/NOV/2013
Schulman Associates Institutional Review Board, Inc.<br>4445 Lake Forest Drive Suite 300<br>Cincinnati, Ohio 45242<br><br>ADVARRA<br>6940 Columbia Gateway Drive,<br>Suite 110<br>Columbia, MD 21046	Sharon Lynn Nelson, MSN, RN, CNS<br><br>Sara Harnish, J.D	32316 / Arora, Chander	Protocol Version 3.0 dated 19/JUL/2011 and Local Amendment dated 17/FEB/2012<br><br>Protocol Version 4.0 dated 07/JUN/2013 (incorporating Global Amendment 1 dated 21/MAY/2013)	11/MAR/2013<br><br>03/JUL/2013

United States of America				
**IRB or IEC (name/address)**	**IRB or IEC Chairperson**	**Centre number (5 digit number) / Investigator(s)**	**Protocol and/or Amendment number(s)**	Date of Final Approval (DD/MMM/YYYY)
Schulman Associates Institutional Review Board, Inc.<br>4445 Lake Forest Drive Suite 300<br>Cincinnati, Ohio 45242<br><br>ADVARRA<br>6940 Columbia Gateway Drive,<br>Suite 110<br>Columbia, MD 21046	Sharon Lynn Nelson, MSN, RN, CNS<br><br>Sara Harnish, J.D	32317 / Dauber, Ira	Protocol Version 3.0 dated 19/JUL/2011 and Local Amendment dated 17/FEB/2012<br><br>Protocol Version 4.0 dated 07/JUN/2013 (incorporating Global Amendment 1 dated 21/MAY/2013)	27/FEB/2013<br><br>03/JUL/2013
Schulman Associates Institutional Review Board, Inc.<br>4445 Lake Forest Drive	Sharon Lynn Nelson, MSN, RN, CNS	32318 / Tallet, Julio	Protocol Version 3.0 dated 19/JUL/2011 and Local Amendment dated 17/FEB/2012	26/FEB/2013

United States of America				
**IRB or IEC (name/address)**	**IRB or IEC Chairperson**	**Centre number (5 digit number) / Investigator(s)**	**Protocol and/or Amendment number(s)**	Date of Final Approval (DD/MMM/YYYY)
Suite 300<br>Cincinnati, Ohio 45242<br><br>ADVARRA<br>6940 Columbia Gateway Drive,<br>Suite 110<br>Columbia, MD 21046	Sara Harnish, J.D		Protocol Version 4.0 dated 07/JUN/2013 (incorporating Global Amendment 1 dated 21/MAY/2013)	03/JUL/2013
Schulman Associates Institutional Review Board, Inc.<br>4445 Lake Forest Drive Suite 300<br>Cincinnati, Ohio 45242<br><br>ADVARRA<br>6940 Columbia Gateway	Sharon Lynn Nelson, MSN, RN, CNS<br><br>Sara Harnish, J.D	32320 / Kneller, G. Larsen	Protocol Version 3.0 dated 19/JUL/2011 and Local Amendment dated 17/FEB/2012<br><br>Protocol Version 4.0 dated 07/JUN/2013 (incorporating Global Amendment 1 dated	23/JAN/2013<br><br>03/JUL/2013

United States of America				
**IRB or IEC (name/address)**	**IRB or IEC Chairperson**	**Centre number (5 digit number) / Investigator(s)**	**Protocol and/or Amendment number(s)**	Date of Final Approval (DD/MMM/YYYY)
Drive,<br>Suite 110<br>Columbia, MD 21046			21/MAY/2013)	
Schulman Associates Institutional Review Board, Inc. 4445 Lake Forest Drive Suite 300 Cincinnati, Ohio 45242<br><br>ADVARRA<br>6940 Columbia Gateway Drive,<br>Suite 110<br>Columbia, MD 21046	Sharon Lynn Nelson, MSN, RN, CNS<br><br>Sara Harnish, J.D	32321 / Salfity, Marwan	Protocol Version 3.0 dated 19/JUL/2011 and Local Amendment dated 17/FEB/2012<br><br>Protocol Version 4.0 dated 07/JUN/2013 (incorporating Global Amendment 1 dated 21/MAY/2013)	05/NOV/2012<br><br>03/JUL/2013

United States of America				
**IRB or IEC (name/address)**	**IRB or IEC Chairperson**	**Centre number (5 digit number) / Investigator(s)**	**Protocol and/or Amendment number(s)**	Date of Final Approval (DD/MMM/YYYY)
UTSW IRB<br>5323 Harry Hines Blvd.<br>Dallas, TX 75390	Idris Ahamed	32322 / Wu, Richard	Protocol Version 3.0 dated 19/JUL/2011 and Local Amendment dated 17/FEB/2012<br><br>Protocol Version 4.0 dated 07/JUN/2013 (incorporating Global Amendment 1 dated 21/MAY/2013)	11/JAN/2013<br><br>28/OCT/2013
Western Institutional Review Board 1019 39th Avenue SE, Suite 120 Puyallup, WA 98374-2115	Bert Wilkins	32323 / Mainigi, Sumeet	Protocol Version 3.0 dated 19/JUL/2011 and Local Amendment dated 17/FEB/2012	02/JAN/2013

United States of America				
**IRB or IEC (name/address)**	**IRB or IEC Chairperson**	**Centre number (5 digit number) / Investigator(s)**	**Protocol and/or Amendment number(s)**	Date of Final Approval (DD/MMM/YYYY)
			Protocol Version 4.0 dated 07/JUN/2013 (incorporating Global Amendment 1 dated 21/MAY/2013)	12/AUG/2013
Schulman Associates Institutional Review Board, Inc.<br>4445 Lake Forest Drive Suite 300<br>Cincinnati, Ohio 45242<br><br>ADVARRA<br>6940 Columbia Gateway	Sharon Lynn Nelson, MSN, RN, CNS	32326 / Ariani, Mehrdad	Protocol Version 3.0 dated 19/JUL/2011 and Local Amendment dated 17/FEB/2012<br><br>Protocol Version 4.0 dated 07/JUN/2013 (incorporating Global Amendment 1 dated 21/MAY/2013)	08/MAR/2013<br><br>03/JUL/2013

United States of America				
**IRB or IEC (name/address)**	**IRB or IEC Chairperson**	**Centre number (5 digit number) / Investigator(s)**	**Protocol and/or Amendment number(s)**	Date of Final Approval (DD/MMM/YYYY)
Drive,<br>Suite 110<br>Columbia, MD 21046	Sara Harnish, J.D			
Schulman Associates Institutional Review Board, Inc.<br>4445 Lake Forest Drive Suite 300<br>Cincinnati, Ohio 45242<br><br>ADVARRA<br>6940 Columbia Gateway Drive,<br>Suite 110<br>Columbia, MD 21046	Sharon Lynn Nelson, MSN, RN, CNS<br><br>Sara Harnish, J.D	32327 / Aslam, M. Shakil	Protocol Version 3.0 dated 19/JUL/2011 and Local Amendment dated 17/FEB/2012<br><br>Protocol Version 4.0 dated 07/JUN/2013 (incorporating Global Amendment 1 dated 21/MAY/2013)	26/MAR/2013<br><br>03/JUL/2013
Schulman Associates Institutional Review Board, Inc.<br>4445 Lake Forest Drive	Sharon Lynn Nelson, MSN, RN, CNS	32328 / Sandesara, Chirag	Protocol Version 3.0 dated 19/JUL/2011 and Local Amendment dated 17/FEB/2012	28/MAR/2013

United States of America				
**IRB or IEC (name/address)**	**IRB or IEC Chairperson**	**Centre number (5 digit number) / Investigator(s)**	**Protocol and/or Amendment number(s)**	Date of Final Approval (DD/MMM/YYYY)
Suite 300<br>Cincinnati, Ohio 45242<br><br>ADVARRA<br>6940 Columbia Gateway Drive,<br>Suite 110<br>Columbia, MD 21046	Sara Harnish, J.D		Protocol Version 4.0 dated 07/JUN/2013 (incorporating Global Amendment 1 dated 21/MAY/2013)	03/JUL/2013
Stanford University IRB<br>Research Compliance Office<br>3000 El Camino Real<br>Five Palo Alto Square, 4th Floor<br>Palo Alto, CA 94306	David Oaks	32329 / Turakhia, Minang	Protocol Version 3.0 dated 19/JUL/2011 and Local Amendment dated 17/FEB/2012<br><br>Protocol Version 4.0 dated 07/JUN/2013 (incorporating Global Amendment 1 dated 21/MAY/2013)	28/MAR/2013<br><br>13/AUG/2013

United States of America				
**IRB or IEC (name/address)**	**IRB or IEC Chairperson**	**Centre number (5 digit number) / Investigator(s)**	**Protocol and/or Amendment number(s)**	Date of Final Approval (DD/MMM/YYYY)
Western Institutional Review Board<br>1019 39th Avenue SE, Suite 120 Puyallup, WA 98374-2115	Bert Wilkins	32332 / Torosoff, Mikhail	Protocol Version 3.0 dated 19/JUL/2011 and Local Amendment dated 17/FEB/2012<br><br>Protocol Version 4.0 dated 07/JUN/2013 (incorporating Global Amendment 1 dated 21/MAY/2013)	08/MAR/2013<br><br>17/AUG/2013
Schulman Associates Institutional Review Board, Inc.<br>4445 Lake Forest Drive Suite 300<br>Cincinnati, Ohio 45242<br>ADVARRA<br>6940 Columbia Gateway Drive,<br>Suite 110	Sharon Lynn Nelson, MSN, RN, CNS<br><br>Sara Harnish, J.D	32334 / Shoultz, Charles	Protocol Version 3.0 dated 19/JUL/2011 and Local Amendment dated 17/FEB/2012<br><br>Protocol Version 4.0 dated 07/JUN/2013 (incorporating Global Amendment 1 dated 21/MAY/2013)	07/MAR/2013<br><br>03/JUL/2013

United States of America				
**IRB or IEC (name/address)**	**IRB or IEC Chairperson**	**Centre number (5 digit number) / Investigator(s)**	**Protocol and/or Amendment number(s)**	Date of Final Approval (DD/MMM/YYYY)
Columbia, MD 21046				
Schulman Associates Institutional Review Board, Inc.<br>4445 Lake Forest Drive Suite 300<br>Cincinnati, Ohio 45242<br><br>ADVARRA<br>6940 Columbia Gateway Drive,<br>Suite 110<br>Columbia, MD 21046 USA	Sharon Lynn Nelson, MSN, RN, CNS<br><br>Sara Harnish, J.D	32335 / Compton, Steven	Protocol Version 3.0 dated 19/JUL/2011 and Local Amendment dated 17/FEB/2012<br><br>Protocol Version 4.0 dated 07/JUN/2013 (incorporating Global Amendment 1 dated 21/MAY/2013)	01/APR/2013<br><br>03/JUL/2013
University of Pennsylvania Office of	Emma Meagher	32337 Bullinga, John	Protocol Version 3.0 dated 19/JUL/2011 and Local	15/JAN/2013

United States of America				
**IRB or IEC (name/address)**	**IRB or IEC Chairperson**	**Centre number (5 digit number) / Investigator(s)**	**Protocol and/or Amendment number(s)**	Date of Final Approval (DD/MMM/YYYY)
Regulatory Affairs<br>3624 Market St.<br>Suite 301 S<br>Philadelphia, PA 191044445 Lake Forest Drive Suite 300<br>Cincinnati, Ohio 45242			Amendment dated 17/FEB/2012<br><br>Protocol Version 4.0 dated 07/JUN/2013 (incorporating Global Amendment 1 dated 21/MAY/2013)	13/SEP/2013
Schulman Associates Institutional Review Board, Inc.<br>4445 Lake Forest Drive Suite 300<br>Cincinnati, Ohio 45242<br><br>ADVARRA<br>6940 Columbia Gateway	Sharon Lynn Nelson, MSN, RN, CNS<br><br>Sara Harnish, J.D	32338 / Crenshaw, James	Protocol Version 3.0 dated 19/JUL/2011 and Local Amendment dated 17/FEB/2012<br><br>Protocol Version 4.0 dated 07/JUN/2013 (incorporating Global	28/MAR/2013<br><br>03/JUL/2013

United States of America				
**IRB or IEC (name/address)**	**IRB or IEC Chairperson**	**Centre number (5 digit number) / Investigator(s)**	**Protocol and/or Amendment number(s)**	Date of Final Approval (DD/MMM/YYYY)
Drive,<br>Suite 110<br>Columbia, MD 21046			Amendment 1 dated 21/MAY/2013)	
Schulman Associates Institutional Review Board, Inc.<br>4445 Lake Forest Drive Suite 300<br>Cincinnati, Ohio 45242<br><br>ADVARRA	Sharon Lynn Nelson, MSN, RN, CNS<br><br>Sara Harnish, J.D	32339 / Mikhail, Magdy	Protocol Version 3.0 dated 19/JUL/2011 and Local Amendment dated 17/FEB/2012<br><br>Protocol Version 4.0 dated 07/JUN/2013 (incorporating Global Amendment 1 dated 21/MAY/2013)	16/APR/2013<br><br>03/JUL/2013
Schulman Associates Institutional Review Board, Inc.<br>4445 Lake Forest Drive Suite 300<br>Cincinnati, Ohio 45242	Sharon Lynn Nelson, MSN, RN, CNS	32340 / Patel, Bharmendra	Protocol Version 3.0 dated 19/JUL/2011 and Local Amendment dated 17/FEB/2012<br><br>Protocol Version 4.0 dated 07/JUN/2013	10/APR/2013<br><br>03/JUL/2013

United States of America				
**IRB or IEC (name/address)**	**IRB or IEC Chairperson**	**Centre number (5 digit number) / Investigator(s)**	**Protocol and/or Amendment number(s)**	Date of Final Approval (DD/MMM/YYYY)
ADVARRA<br>6940 Columbia Gateway Drive,<br>Suite 110<br>Columbia, MD 21046	Sara Harnish, J.D		(incorporating Global Amendment 1 dated 21/MAY/2013)	
Schulman Associates Institutional Review Board, Inc.<br>4445 Lake Forest Drive Suite 300<br>Cincinnati, Ohio 45242<br><br>ADVARRA<br>6940 Columbia Gateway Drive,	Sharon Lynn Nelson, MSN, RN, CNS<br><br>Sara Harnish, J.D	32342 / Baker, Seth	Protocol Version 3.0 dated 19/JUL/2011 and Local Amendment dated 17/FEB/2012<br><br>Protocol Version 4.0 dated 07/JUN/2013 (incorporating Global Amendment 1 dated 21/MAY/2013)	01/MAY/2013<br><br>03/JUL/2013
Schulman Associates Institutional Review Board, Inc.<br>4445 Lake Forest Drive	Sharon Lynn Nelson, MSN, RN, CNS	32345 / Israel, Noah	Protocol Version 3.0 dated 19/JUL/2011 and Local Amendment dated 17/FEB/2012	25/MAR/2013

United States of America				
**IRB or IEC (name/address)**	**IRB or IEC Chairperson**	**Centre number (5 digit number) / Investigator(s)**	**Protocol and/or Amendment number(s)**	Date of Final Approval (DD/MMM/YYYY)
Suite 300<br>Cincinnati, Ohio 45242<br><br>ADVARRA<br>6940 Columbia Gateway Drive,<br>Suite 110<br>Columbia, MD 21046 2	Sara Harnish, J.D		Protocol Version 4.0 dated 07/JUN/2013 (incorporating Global Amendment 1 dated 21/MAY/2013)	03/JUL/2013
Schulman Associates Institutional Review Board, Inc.<br>4445 Lake Forest Drive Suite 300<br>Cincinnati, Ohio 45242<br><br>ADVARRA<br>6940 Columbia Gateway Drive,<br>Suite 110<br>Columbia, MD 21046	Sharon Lynn Nelson, MSN, RN, CNS<br><br>Sara Harnish, J.D	32349 / Sarikonda, Kesari	Protocol Version 3.0 dated 19/JUL/2011 and Local Amendment dated 17/FEB/2012<br><br>Protocol Version 4.0 dated 07/JUN/2013 (incorporating Global Amendment 1 dated 21/MAY/2013)	18/JAN/2013<br><br>03/JUL/2013

United States of America				
**IRB or IEC (name/address)**	**IRB or IEC Chairperson**	**Centre number (5 digit number) / Investigator(s)**	**Protocol and/or Amendment number(s)**	Date of Final Approval (DD/MMM/YYYY)
Schulman Associates Institutional Review Board, Inc.<br>4445 Lake Forest Drive Suite 300<br>Cincinnati, Ohio 45242	Sharon Lynn Nelson, MSN, RN, CNS	32354 / Lumicao, Benjamin	Protocol Version 3.0 dated 19/JUL/2011 and Local Amendment dated 17/FEB/2012	05/JUN/2013
ADVARRA<br>6940 Columbia Gateway Drive,<br>Suite 110<br>Columbia, MD 21046 USA	Sara Harnish, J.D		Protocol Version 4.0 dated 07/JUN/2013 (incorporating Global Amendment 1 dated 21/MAY/2013)	03/JUL/2013

United States of America				
**IRB or IEC (name/address)**	**IRB or IEC Chairperson**	**Centre number (5 digit number) / Investigator(s)**	**Protocol and/or Amendment number(s)**	Date of Final Approval (DD/MMM/YYYY)
ProHealth Care IRB<br>725 American Ave.<br>Suite 502<br>Waukesha, WI 53188	Michael Whittaker, MD	32355 / Schmitz, Lisa	Protocol Version 3.0 dated 19/JUL/2011 and Local Amendment dated 17/FEB/2012<br>Protocol Version 4.0 dated 07/JUN/2013 (incorporating Global Amendment 1 dated 21/MAY/2013)	24/APR/13<br>17/SEP/2013
UAMS IRB<br>MS 636<br>4301 W. Markham<br>Little Rock, AK 72205	Paul Gubbins	32356 / Wei, Jeanne	Protocol Version 3.0 dated 19/JUL/2011 and Local Amendment dated 17/FEB/2012<br>Protocol Version 4.0 dated 07/JUN/2013 (incorporating Global Amendment 1 dated 21/MAY/2013)	14/MAY/2013<br>22/May/2014

United States of America				
**IRB or IEC (name/address)**	**IRB or IEC Chairperson**	**Centre number (5 digit number) / Investigator(s)**	**Protocol and/or Amendment number(s)**	Date of Final Approval (DD/MMM/YYYY)
Western Institutional Review Board<br>3535 7th AvenueSW<br>Olympia, WA 98502	Bert Wilkins	32357 / Musser, Carl	Protocol Version 3.0 dated 19/JUL/2011 and Local Amendment dated 17/FEB/2012<br>Protocol Version 4.0 dated 07/JUN/2013 (incorporating Global Amendment 1 dated 21/MAY/2013)	20/May/2013<br>17/AUG/2013
Marshall University Office of Research Integrity IRB<br>401 11th St Suite 1300<br>Huntington, WV 25701	Henry K. Driscoll, MD	32359 / Cheema, Aamir	Protocol Version 3.0 dated 19/JUL/2011 and Local Amendment dated 17/FEB/2012<br>Protocol Version 4.0 dated 07/JUN/2013 (incorporating Global Amendment 1 dated 21/MAY/2013)	17/JUN/2013<br>09/SEP/2013

United States of America				
**IRB or IEC (name/address)**	**IRB or IEC Chairperson**	**Centre number (5 digit number) / Investigator(s)**	**Protocol and/or Amendment number(s)**	Date of Final Approval (DD/MMM/YYYY)
St. Luke's- Roosevelt Institute for Health Sciences IRB<br>432 West 58th St.<br>Rm 207<br>New York, NY 10019	Theodore Bania, MD	32361 / Aziz, Emad	Protocol Version 3.0 dated 19/JUL/2011 and Local Amendment dated 17/FEB/2012<br><br>Protocol Version 4.0 dated 07/JUN/2013 (incorporating Global Amendment 1 dated 21/MAY/2013)	20/FEB/2013<br><br>21/AUG/2013
Western Institutional Review Board<br>1019 39th Avenue SE<br>Suite 120<br>Puyallup, WA 98374-2115	Bert Wilkins	32362 / Slobodova, Adriana	Protocol Version 3.0 dated 19/JUL/2011 and Local Amendment dated 17/FEB/2012	05/JUN/2013

United States of America				
**IRB or IEC (name/address)**	**IRB or IEC Chairperson**	**Centre number (5 digit number) / Investigator(s)**	**Protocol and/or Amendment number(s)**	Date of Final Approval (DD/MMM/YYYY)
			Protocol Version 4.0 dated 07/JUN/2013 (incorporating Global Amendment 1 dated 21/MAY/2013)	21/AUG/2013
Sentara Rockingham Memorial Hospital IRB<br>2010 Health Campus Dr.<br>Harrisonburg, VA 22801	Laura Adkins	32363 / Pollock, Stewart	Protocol Version 3.0 dated 19/JUL/2011 and Local Amendment dated 17/FEB/2012<br><br>Protocol Version 4.0 dated 07/JUN/2013 (incorporating Global Amendment 1 dated 21/MAY/2013)	13/AUG/2013<br><br>13/AUG/2013
Peace Health System, IRB<br>1255 Hilyard Street<br>Eugene, OR 97401	Phyllis Brown	32366 / Reddy, Ramakota	Protocol Version 3.0 dated 19/JUL/2011 and Local Amendment dated 17/FEB/2012	17/JUN/2013

United States of America				
**IRB or IEC (name/address)**	**IRB or IEC Chairperson**	**Centre number (5 digit number) / Investigator(s)**	**Protocol and/or Amendment number(s)**	Date of Final Approval (DD/MMM/YYYY)
			Protocol Version 4.0 dated 07/JUN/2013 (incorporating Global Amendment 1 dated 21/MAY/2013)	10/OCT/2013
Salem VA Med Center IRB<br>1970 Roanoke Blvd.<br>Salem, VA 24153	Steven, Lash	32367 / Jarmukli, Nabil	Protocol Version 3.0 dated 19/JUL/2011 and Local Amendment dated 17/FEB/2012<br><br>Protocol Version 4.0 dated 07/JUN/2013 (incorporating Global Amendment 1 dated 21/MAY/2013)	03/JUN/2013<br><br>03/SEP/2013
Schulman Associates Institutional Review Board, Inc.<br>4445 Lake Forest Drive	Sharon Lynn Nelson, MSN, RN, CNS	32369 / O'Donnell, Phillip	Protocol Version 3.0 dated 19/JUL/2011 and Local Amendment dated 17/FEB/2012	27/AUG/2013

United States of America				
**IRB or IEC (name/address)**	**IRB or IEC Chairperson**	**Centre number (5 digit number) / Investigator(s)**	**Protocol and/or Amendment number(s)**	Date of Final Approval (DD/MMM/YYYY)
Suite 300<br>Cincinnati, Ohio 45242<br><br>ADVARRA<br>6940 Columbia Gateway Drive,<br>Suite 110<br>Columbia, MD 21046	Sara Harnish, J.D		Protocol Version 4.0 dated 07/JUN/2013 (incorporating Global Amendment 1 dated 21/MAY/2013)	03/JUL/2013
Schulman Associates Institutional Review Board, Inc.<br>4445 Lake Forest Drive Suite 300<br>Cincinnati, Ohio 45242<br><br>ADVARRA<br>6940 Columbia Gateway Drive,<br>Suite 110<br>Columbia, MD 21046	Sharon Lynn Nelson, MSN, RN, CNS<br><br>Sara Harnish, J.D	32370 / Shah, Anil	Protocol Version 3.0 dated 19/JUL/2011 and Local Amendment dated 17/FEB/2012<br><br>Protocol Version 4.0 dated 07/JUN/2013 (incorporating Global Amendment 1 dated 21/MAY/2013)	06/SEP/2013<br><br>03/JUL/2013

United States of America				
**IRB or IEC (name/address)**	**IRB or IEC Chairperson**	**Centre number (5 digit number) / Investigator(s)**	**Protocol and/or Amendment number(s)**	Date of Final Approval (DD/MMM/YYYY)
USA				
University of Pennsylvania Office of Regulatory Affairs<br>3624 Market St.<br>Suite 301 S<br>Philadelphia, PA 19104	Benjamin Hernberg	32371 / Pentz, William	Protocol Version 3.0 dated 19/JUL/2011 and Local Amendment dated 17/FEB/2012<br><br>Protocol Version 4.0 dated 07/JUN/2013 (incorporating Global Amendment 1 dated 21/MAY/2013)	16/JUL/2013<br><br>30/JUL/2013
Northshore LIJ<br>Human Research Protection Program<br>350 Community Drive<br>Manhasset, NY 11030	Martin Lesser, PhD	32372 / Spyropoulos, Alex	Protocol Version 3.0 dated 19/JUL/2011 and Local Amendment dated 17/FEB/2012<br><br>Protocol Version 4.0 dated 07/JUN/2013	11/JUL/2013

United States of America				
**IRB or IEC (name/address)**	**IRB or IEC Chairperson**	**Centre number (5 digit number) / Investigator(s)**	**Protocol and/or Amendment number(s)**	Date of Final Approval (DD/MMM/YYYY)
			(incorporating Global Amendment 1 dated 21/MAY/2013)<br>1	23/APR/2014
CHI Institute for Research Innovation IRB<br>1717 South J St.<br>P.O. Box 2197<br>Tacoma, WA 98401	Michael Bonck, BS, RPh	32373 / Fahmy, Raed	Protocol Version 3.0 dated 19/JUL/2011 and Local Amendment dated 17/FEB/2012<br>Protocol Version 4.0 dated 07/JUN/2013 (incorporating Global Amendment 1 dated 21/MAY/2013)	26/Mar/2013<br>27/AUG/2013
SHS Regional IRB<br>Office of Human Research Protection	Dan Rackham, PhD	32374 / Hsing, Jeff	Protocol Version 3.0 dated 19/JUL/2011 and Local Amendment dated 17/FEB/2012	26/SEP/2013

United States of America				
**IRB or IEC (name/address)**	**IRB or IEC Chairperson**	**Centre number (5 digit number) / Investigator(s)**	**Protocol and/or Amendment number(s)**	Date of Final Approval (DD/MMM/YYYY)
815 NW 9th St.<br>Suite 136<br>Corvallis, OR 97330			Protocol Version 4.0 dated 07/JUN/2013 (incorporating Global Amendment 1 dated 21/MAY/2013)	26/SEP/2013
Southern Illinois Hospital<br>1239 East Main St.<br>Carbondale, IL 62901	Padmalatha Chandrashekar	32375 / Al-Dallow, Raed	Protocol Version 3.0 dated 19/JUL/2011 and Local Amendment dated 17/FEB/2012<br><br>Protocol Version 4.0 dated 07/JUN/2013 (incorporating Global Amendment 1 dated 21/MAY/2013)	16/OCT/2013<br><br>16/OCT/2013
Parkview Health IRB<br>10501 Corporate Dr.	Dennis Dykhuizen, BS, JD	32376 / Mirro, Michael	Protocol Version 3.0 dated 19/JUL/2011 and Local Amendment dated	30/APR/2013

United States of America				
**IRB or IEC (name/address)**	**IRB or IEC Chairperson**	**Centre number (5 digit number) / Investigator(s)**	**Protocol and/or Amendment number(s)**	Date of Final Approval (DD/MMM/YYYY)
Fort Wayne, IN 46845			17/FEB/2012<br>Protocol Version 4.0 dated 07/JUN/2013 (incorporating Global Amendment 1 dated 21/MAY/2013)	30/APR/2013
University of Virginia IRB for Health Sciences Research<br>One Morton Drive<br>Suite 400, Box 5<br>Charlottesville, VA 22903	Richard Stevenson, MD	32377 /Simpson, Allan	Protocol Version 3.0 dated 19/JUL/2011 and Local Amendment dated 17/FEB/2012<br>Protocol Version 4.0 dated 07/JUN/2013 (incorporating Global Amendment 1 dated 21/MAY/2013)	05/SEP/2012<br>04/NOV/2013
Schulman Associates Institutional Review Board, Inc.<br>4445 Lake Forest Drive Suite 300	Sharon Lynn Nelson, MSN, RN, CNS	32378 / Covalesky, John	Protocol Version 3.0 dated 19/JUL/2011 and Local Amendment dated 17/FEB/2012	09/OCT/2013

United States of America				
**IRB or IEC (name/address)**	**IRB or IEC Chairperson**	**Centre number (5 digit number) / Investigator(s)**	**Protocol and/or Amendment number(s)**	Date of Final Approval (DD/MMM/YYYY)
Cincinnati, Ohio 45242<br><br>ADVARRA<br>6940 Columbia Gateway Drive,<br>Suite 110<br>Columbia, MD 21046 USA	Sara Harnish, J.D		Protocol Version 4.0 dated 07/JUN/2013 (incorporating Global Amendment 1 dated 21/MAY/2013)	09/OCT/2013
Mary Imogene Bassett Hospital (MIBH) IRB<br>One Atwell Road<br>Cooperstown, NY 13326	Linda Keller, PhD	32379 / Menzies, Dhananjai	Protocol Version 3.0 dated 19/JUL/2011 and Local Amendment dated 17/FEB/2012<br><br>Protocol Version 4.0 dated 07/JUN/2013 (incorporating Global Amendment 1 dated 21/MAY/2013)	16/OCT/2013<br><br>16/OCT/2013

United States of America				
**IRB or IEC (name/address)**	**IRB or IEC Chairperson**	**Centre number (5 digit number) / Investigator(s)**	**Protocol and/or Amendment number(s)**	Date of Final Approval (DD/MMM/YYYY)
The Coper Health System IRB<br>401 Haddon Ave.<br>Rooms 128 and 288<br>Camden, NJ 08103	David Warshal, MD	32380 / Russo, Andrea	Protocol Version 3.0 dated 19/JUL/2011 and Local Amendment dated 17/FEB/2012<br><br>Protocol Version 4.0 dated 07/JUN/2013 (incorporating Global Amendment 1 dated 21/MAY/2013)	21/MAR/2013<br><br>12/DEC/2013
Western Institutional Review Board 1019 39th Avenue SE, Suite 120 Puyallup, WA 98374-2115	Bert R. Wilkins	32381 / French, William	Protocol Version 3.0 dated 19/JUL/2011 and Local Amendment dated 17/FEB/2012	09/OCT/2013

United States of America				
**IRB or IEC (name/address)**	**IRB or IEC Chairperson**	**Centre number (5 digit number) / Investigator(s)**	**Protocol and/or Amendment number(s)**	Date of Final Approval (DD/MMM/YYYY)
			Protocol Version 4.0 dated 07/JUN/2013 (incorporating Global Amendment 1 dated 21/MAY/2013)	09/OCT/2013
St. Elizabeth's Medical Center<br>Research/Human Subjects Committee<br>736 Cambridge Street, Boston, Massachusetts 02135	Allan B. Ashare	32382 / Wylie, John	Protocol Version 3.0 dated 19/JUL/2011 and Local Amendment dated 17/FEB/2012<br>Protocol Version 4.0 dated 07/JUN/2013 (incorporating Global Amendment 1 dated 21/MAY/2013)	16/SEP/2013<br>18/DEC/2013
Schulman Associates Institutional Review Board, Inc.	Sharon Lynn Nelson, MSN, RN, CNS	32383 / Trivedi, Ketan	Protocol Version 3.0 dated 19/JUL/2011 and Local Amendment dated	29/OCT/2013

United States of America				
**IRB or IEC (name/address)**	**IRB or IEC Chairperson**	**Centre number (5 digit number) / Investigator(s)**	**Protocol and/or Amendment number(s)**	Date of Final Approval (DD/MMM/YYYY)
4445 Lake Forest Drive Suite 300<br>Cincinnati, Ohio 45242<br><br>ADVARRA<br>6940 Columbia Gateway Drive,<br>Suite 110<br>Columbia, MD 21046 USA	Sara Harnish, J.D		17/FEB/2012<br><br>Protocol Version 4.0 dated 07/JUN/2013 (incorporating Global Amendment 1 dated 21/MAY/2013)	29/OCT/2013
South County Hospital IRB<br>100 Kenyon Ave.<br>Wakefield, RI 02879	Steven Fera	32384 / Fera, Steven	Protocol Version 3.0 dated 19/JUL/2011 and Local Amendment dated 17/FEB/2012<br><br>Protocol Version 4.0 dated 07/JUN/2013 (incorporating Global	21/OCT/2013<br><br>21/OCT/2013

United States of America				
**IRB or IEC (name/address)**	**IRB or IEC Chairperson**	**Centre number (5 digit number) / Investigator(s)**	**Protocol and/or Amendment number(s)**	Date of Final Approval (DD/MMM/YYYY)
			Amendment 1 dated 21/MAY/2013)	
Schulman Associates Institutional Review Board, Inc.<br>4445 Lake Forest Drive Suite 300<br>Cincinnati, Ohio 45242<br><br>ADVARRA<br>6940 Columbia Gateway Drive,<br>Suite 110<br>Columbia, MD 21046 USA	Sharon Lynn Nelson, MSN, RN, CNS<br><br>Sara Harnish, J	32385 / Thakker, Ganpet	Protocol Version 3.0 dated 19/JUL/2011 and Local Amendment dated 17/FEB/2012<br><br>Protocol Version 4.0 dated 07/JUN/2013 (incorporating Global Amendment 1 dated 21/MAY/2013)	24/OCT/2013<br><br>24/OCT/2013
Western Institutional Review Board	Bert Wilkins	32386 / Glotzer, Taya	Protocol Version 3.0 dated 19/JUL/2011 and Local	23/SEP/2013

United States of America				
**IRB or IEC (name/address)**	**IRB or IEC Chairperson**	**Centre number (5 digit number) / Investigator(s)**	**Protocol and/or Amendment number(s)**	Date of Final Approval (DD/MMM/YYYY)
3535 7th Avenue SW, WA 98502			Amendment dated 17/FEB/2012<br><br>Protocol Version 4.0 dated 07/JUN/2013 (incorporating Global Amendment 1 dated 21/MAY/2013)	23/SEP/2013
Western Institutional Review Board<br>1019 39th Avenue SE<br>Suite 120<br>Puyallup, WA 98374-2115	Bert Wilkins	32387 / Goldbarg, Seth	Protocol Version 3.0 dated 19/JUL/2011 and Local Amendment dated 17/FEB/2012<br><br>Protocol Version 4.0 dated 07/JUN/2013 (incorporating Global Amendment 1 dated 21/MAY/2013)	23/SEP/2013<br><br>23/SEP/2013

United States of America				
**IRB or IEC (name/address)**	**IRB or IEC Chairperson**	**Centre number (5 digit number) / Investigator(s)**	**Protocol and/or Amendment number(s)**	Date of Final Approval (DD/MMM/YYYY)
John Hopkins Medicine Office of Subjects Research IRB<br>1620 McElderry St.<br>Reed Hall, Suite B-130<br>Baltimore, MD 21205	Joseph Carrese, MD	32388 / Friedman, Keith	Protocol Version 3.0 dated 19/JUL/2011 and Local Amendment dated 17/FEB/2012<br><br>Protocol Version 4.0 dated 07/JUN/2013 (incorporating Global Amendment 1 dated 21/MAY/2013)	29/APR/2013<br><br>12/AUG/2013
Western Institutional Review Board<br>3535 7th Avenue SW, WA 98502	Bert Wilkins	32389 / Kostis, William	Protocol Version 3.0 dated 19/JUL/2011 and Local Amendment dated 17/FEB/2012	09/JUL/2013

United States of America				
**IRB or IEC (name/address)**	**IRB or IEC Chairperson**	**Centre number (5 digit number) / Investigator(s)**	**Protocol and/or Amendment number(s)**	Date of Final Approval (DD/MMM/YYYY)
			Protocol Version 4.0 dated 07/JUN/2013 (incorporating Global Amendment 1 dated 21/MAY/2013)	23/OCT/2013
Schulman Associates Institutional Review Board, Inc.<br>4445 Lake Forest Drive Suite 300<br>Cincinnati, Ohio 45242<br><br>ADVARRA<br>6940 Columbia Gateway Drive,<br>Suite 110<br>Columbia, MD 21046 USA	Sharon Lynn Nelson, MSN, RN, CNS<br><br>Sara Harnish, J.D	32391 / Nijmeh, George	Protocol Version 3.0 dated 19/JUL/2011 and Local Amendment dated 17/FEB/2012<br><br>Protocol Version 4.0 dated 07/JUN/2013 (incorporating Global Amendment 1 dated 21/MAY/2013))	16/DEC/2013<br><br>16/DEC/2013

United States of America				
**IRB or IEC (name/address)**	**IRB or IEC Chairperson**	**Centre number (5 digit number) / Investigator(s)**	**Protocol and/or Amendment number(s)**	Date of Final Approval (DD/MMM/YYYY)
Robley Rex VAMC IRB<br>800 Zorn Avenue<br>Louisville, KY 40206	Fred Hendler, MD, PhD	32392 / Stoddard, Marcus	Protocol Version 3.0 dated 19/JUL/2011 and Local Amendment dated 17/FEB/2012<br><br>Protocol Version 4.0 dated 07/JUN/2013 (incorporating Global Amendment 1 dated 21/MAY/2013)	17/SEP/2013<br><br>17/SEP/2013
Colorado Multiple IRB, CB F490<br>University of Colorado, Anschutz Medical Campus<br>13001 E. 17th Place<br>Bldg 500, Rm N3214<br>Aurora, CO 80045	Stephen Bartlett, RPh	32393 / Krantz, Mont	Protocol Version 3.0 dated 19/JUL/2011 and Local Amendment dated 17/FEB/2012<br><br>Protocol Version 4.0 dated 07/JUN/2013 (incorporating Global Amendment 1 dated	10/DEC/2013<br><br>10/DEC/2013

United States of America				
**IRB or IEC (name/address)**	**IRB or IEC Chairperson**	**Centre number (5 digit number) / Investigator(s)**	**Protocol and/or Amendment number(s)**	Date of Final Approval (DD/MMM/YYYY)
			21/MAY/2013)	
University of South Alabama IRB<br>CSAB 138<br>Mobile, AL 36688	Hamayun Imran, MD	32394 / Cohen, Michael	Protocol Version 3.0 dated 19/JUL/2011 and Local Amendment dated 17/FEB/2012<br><br>Protocol Version 4.0 dated 07/JUN/2013 (incorporating Global Amendment 1 dated 21/MAY/2013)	18/DEC/2014<br><br>18/DEC/2014
University of Nebraska Medical Center IRB<br>987830 Nebraska Medical Ctr.	Gail Kotulak BS, CIP	32396 / Scherschel, John	Protocol Version 3.0 dated 19/JUL/2011 and Local Amendment dated 17/FEB/2012	18/NOV/2013

United States of America				
**IRB or IEC (name/address)**	**IRB or IEC Chairperson**	**Centre number (5 digit number) / Investigator(s)**	**Protocol and/or Amendment number(s)**	Date of Final Approval (DD/MMM/YYYY)
Omaha, NE 68198			Protocol Version 4.0 dated 07/JUN/2013 (incorporating Global Amendment 1 dated 21/MAY/2013)	11/MAR/2014
Upstate Medical Center SUNY Upstate IRB<br>750 East Adams St Syracuse NY 13210	Stephen Graziano	32397 / Bhatta, Luna	Protocol Version 3.0 dated 19/JUL/2011 and Local Amendment dated 17/FEB/2012	07/JAN/2014
			Protocol Version 4.0 dated 07/JUN/2013 (incorporating Global Amendment 1 dated 21/MAY/2013)	07/JAN/2014

United States of America				
**IRB or IEC (name/address)**	**IRB or IEC Chairperson**	**Centre number (5 digit number) / Investigator(s)**	**Protocol and/or Amendment number(s)**	Date of Final Approval (DD/MMM/YYYY)
Schulman Associates Institutional Review Board, Inc.<br>4445 Lake Forest Drive Suite 300<br>Cincinnati, Ohio 45242<br><br>ADVARRA<br>6940 Columbia Gateway Drive,<br>Suite 110<br>Columbia, MD 21046 USA	Sharon Lynn Nelson, MSN, RN, CNS<br><br>Sara Harnish, J.D	32398 / Canosa,Roddy	Protocol Version 3.0 dated 19/JUL/2011 and Local Amendment dated 17/FEB/2012<br><br>Protocol Version 4.0 dated 07/JUN/2013 (incorporating Global Amendment 1 dated 21/MAY/2013)	14/JAN/2014<br><br>14/JAN/2014
Abington Memorial Hospital<br>1200 Old York Rd.<br>Abington, PA 19001	Chris Christianasen	32400 / Borge Jr, Richard	Protocol Version 3.0 dated 19/JUL/2011 and Local Amendment dated 17/FEB/2012	26/MAR/2014

United States of America				
**IRB or IEC (name/address)**	**IRB or IEC Chairperson**	**Centre number (5 digit number) / Investigator(s)**	**Protocol and/or Amendment number(s)**	Date of Final Approval (DD/MMM/YYYY)
			Protocol Version 4.0 dated 07/JUN/2013 (incorporating Global Amendment 1 dated 21/MAY/2013)	15/MAY/2014
Schulman Associates Institutional Review Board, Inc.<br>4445 Lake Forest Drive Suite 300<br>Cincinnati, Ohio 45242<br>ADVARRA<br>6940 Columbia Gateway Drive,<br>Suite 110<br>Columbia, MD 21046	Sharon Lynn Nelson, MSN, RN, CNS<br><br>Sara Harnish, J.D	32401 / Bartlett, Maria	Protocol Version 3.0 dated 19/JUL/2011 and Local Amendment dated 17/FEB/2012<br><br>Protocol Version 4.0 dated 07/JUN/2013 (incorporating Global Amendment 1 dated 21/MAY/2013)	09/SEP/2013<br><br>09/SEP/2013

United States of America				
**IRB or IEC (name/address)**	**IRB or IEC Chairperson**	**Centre number (5 digit number) / Investigator(s)**	**Protocol and/or Amendment number(s)**	Date of Final Approval (DD/MMM/YYYY)
Schulman Associates Institutional Review Board, Inc.<br>4445 Lake Forest Drive Suite 300<br>Cincinnati, Ohio 45242<br><br>ADVARRA<br>6940 Columbia Gateway Drive,<br>Suite 110<br>Columbia, MD 21046	Sharon Lynn Nelson, MSN, RN, CNS<br><br>Sara Harnish, J.D	32402 / Cardona, Francisco	Protocol Version 3.0 dated 19/JUL/2011 and Local Amendment dated 17/FEB/2012<br><br>Protocol Version 4.0 dated 07/JUN/2013 (incorporating Global Amendment 1 dated 21/MAY/2013)	16/JAN/2014<br><br>16/JAN/2014
Schulman Associates Institutional Review Board, Inc.<br>4445 Lake Forest Drive Suite 300<br>Cincinnati, Ohio 45242<br><br>ADVARRA	Sharon Lynn Nelson, MSN, RN, CNS	32403 / Jain, Rakesh	Protocol Version 3.0 dated 19/JUL/2011 and Local Amendment dated 17/FEB/2012<br><br>Protocol Version 4.0	02/FEB/2014

United States of America				
**IRB or IEC (name/address)**	**IRB or IEC Chairperson**	**Centre number (5 digit number) / Investigator(s)**	**Protocol and/or Amendment number(s)**	Date of Final Approval (DD/MMM/YYYY)
6940 Columbia Gateway Drive,<br>Suite 110<br>Columbia, MD 21046 USA	Sara Harnish, J.D		dated 07/JUN/2013 (incorporating Global Amendment 1 dated 21/MAY/2013)	02/FEB/2014
Heartland Regional Medical Center IRB<br>5325 Faraon<br>St. Joseph, MO 64506	Robert C. Johnson	32404 / Ramos, Ricardo	Protocol Version 3.0 dated 19/JUL/2011 and Local Amendment dated 17/FEB/2012<br><br>Protocol Version 4.0 dated 07/JUN/2013 (incorporating Global Amendment 1 dated 21/MAY/2013)	16/DEC/2013<br><br>27/MAY/2014

United States of America				
**IRB or IEC (name/address)**	**IRB or IEC Chairperson**	**Centre number (5 digit number) / Investigator(s)**	**Protocol and/or Amendment number(s)**	Date of Final Approval (DD/MMM/YYYY)
Essentia Health IRB<br>502 East Second St.<br>Duluth, MN 55805	Catherine A. McCarty	32405 / Mollerus, Michael	Protocol Version 3.0 dated 19/JUL/2011 and Local Amendment dated 17/FEB/2012<br><br>Protocol Version 4.0 dated 07/JUN/2013 (incorporating Global Amendment 1 dated 21/MAY/2013)	04/FEB/2014<br><br>04/FEB/2014
Schulman Associates Institutional Review Board, Inc.<br>4445 Lake Forest Drive Suite 300<br>Cincinnati, Ohio 45242	Sharon Lynn Nelson, MSN, RN, CNS	32406 / Tayal, Ashis	Protocol Version 3.0 dated 19/JUL/2011 and Local Amendment dated 17/FEB/2012	29/JAN/2014

United States of America				
**IRB or IEC (name/address)**	**IRB or IEC Chairperson**	**Centre number (5 digit number) / Investigator(s)**	**Protocol and/or Amendment number(s)**	Date of Final Approval (DD/MMM/YYYY)
ADVARRA<br>6940 Columbia Gateway Drive,<br>Suite 110<br>Columbia, MD 21046 USA	Sara Harnish, J.D		Protocol Version 4.0 dated 07/JUN/2013 (incorporating Global Amendment 1 dated 21/MAY/2013)	29/JAN/2014
University of Nevada, Reno Biomedical IRB<br>Research Integrity Office<br>218 Ross Hall/ 331<br>Reno, NV 89557	Richard Bjur, PhD	32407 / Minaie, Sedi	Protocol Version 4.0 dated 07/JUN/2013 (incorporating Global Amendment 1 dated 21/MAY/2013)	13/NOV/2013
University of Mississippi Medical Center IRB<br>2500 North State Street	N/A	32408 / Tanawuttiwat, Tanyanan	Protocol Version 4.0 dated 07/JUN/2013 (incorporating Global	12/FEB/2014

United States of America				
**IRB or IEC (name/address)**	**IRB or IEC Chairperson**	**Centre number (5 digit number) / Investigator(s)**	**Protocol and/or Amendment number(s)**	Date of Final Approval (DD/MMM/YYYY)
Jackson, MS 39216			Amendment 1 dated 21/MAY/2013)	
Human Subjects Office/IRB<br>105 Hardin Library for the Health Sciences<br>600 Newton Rd.<br>Iowa City, IA 52242	Andrew Bertolatus, MD	32411 / Giudici, Michael	Protocol Version 4.0 dated 07/JUN/2013 (incorporating Global Amendment 1 dated 21/MAY/2013)	25/MAR/2014
University of Illinois at Chicago<br>OPRS MC 672<br>203 Administrative Office Building<br>1737 West Polk St.<br>Chicago, IL 60612	Patricia West-Thielke, PhD	32412 / Gans, Christopher	Protocol Version 3.0 dated 19/JUL/2011 and Local Amendment dated 17/FEB/2012<br><br>Protocol Version 4.0 dated 07/JUN/2013 (incorporating Global Amendment 1 dated 21/MAY/2013)	08/AUG/2014<br><br>08/AUG/2014

United States of America				
**IRB or IEC (name/address)**	**IRB or IEC Chairperson**	**Centre number (5 digit number) / Investigator(s)**	**Protocol and/or Amendment number(s)**	Date of Final Approval (DD/MMM/YYYY)
Howard University IRB<br>Howard University Research Building<br>11840 7th St. NW Suite 309<br>Washington, DC 20001	Robert L. Copeland, Jr. PhD	32414 / Randall, Otelio	Protocol Version 3.0 dated 19/JUL/2011 and Local Amendment dated 17/FEB/2012<br><br>Protocol Version 4.0 dated 07/JUN/2013 (incorporating Global Amendment 1 dated 21/MAY/2013)	05/MAY/2014<br><br>05/MAY/2014

United States of America				
**IRB or IEC (name/address)**	**IRB or IEC Chairperson**	**Centre number (5 digit number) / Investigator(s)**	**Protocol and/or Amendment number(s)**	Date of Final Approval (DD/MMM/YYYY)
Schulman Associates Institutional Review Board, Inc.<br>4445 Lake Forest Drive Suite 300<br>Cincinnati, Ohio 45242<br><br>ADVARRA<br>6940 Columbia Gateway Drive,<br>Suite 110<br>Columbia, MD 21046	Sharon Lynn Nelson, MSN, RN, CNS<br><br>Sara Harnish, J.D	32418 / Weiner, Stan	Protocol Version 3.0 dated 19/JUL/2011 and Local Amendment dated 17/FEB/2012<br><br>Protocol Version 4.0 dated 07/JUN/2013 (incorporating Global Amendment 1 dated 21/MAY/2013)	22/JAN/2014<br><br>22/JAN/2014
Chesapeake IRB<br>7063 Columbia Gateway Drive<br>Suite 110<br>Columbia, MD 21046	Joy Cavagnaro	32421 / Addala, Srinivas	Protocol Version 3.0 dated 19/JUL/2011 and Local Amendment dated 17/FEB/2012<br><br>Protocol Version 4.0 dated 07/JUN/2013 (incorporating Global	03/FEB/2014<br><br>03/FEB/2014

United States of America				
**IRB or IEC (name/address)**	**IRB or IEC Chairperson**	**Centre number (5 digit number) / Investigator(s)**	**Protocol and/or Amendment number(s)**	Date of Final Approval (DD/MMM/YYYY)
			Amendment 1 dated 21/MAY/2013)	
Schulman Associates Institutional Review Board, Inc.<br>4445 Lake Forest Drive Suite 300<br>Cincinnati, Ohio 45242<br><br>ADVARRA<br>6940 Columbia Gateway Drive,<br>Suite 110<br>Columbia, MD 21046	Sharon Lynn Nelson, MSN, RN, CNS<br><br>Sara Harnish, J.D	32422 / Assefa, Getu	Protocol Version 3.0 dated 19/JUL/2011 and Local Amendment dated 17/FEB/2012<br><br>Protocol Version 4.0 dated 07/JUN/2013 (incorporating Global Amendment 1 dated 21/MAY/2013)	27/JUN/2014<br><br>27/JUN/2014
Schulman Associates Institutional Review Board, Inc.<br>4445 Lake Forest Drive	Sharon Lynn Nelson, MSN, RN, CNS	32424 / Kumar, Priya	Protocol Version 3.0 dated 19/JUL/2011 and Local Amendment dated 17/FEB/2012	23/MAY/2014

United States of America				
**IRB or IEC (name/address)**	**IRB or IEC Chairperson**	**Centre number (5 digit number) / Investigator(s)**	**Protocol and/or Amendment number(s)**	Date of Final Approval (DD/MMM/YYYY)
Suite 300<br>Cincinnati, Ohio 45242<br><br>ADVARRA<br>6940 Columbia Gateway Drive,<br>Suite 110<br>Columbia, MD 21046	Sara Harnish, J.D		Protocol Version 4.0 dated 07/JUN/2013 (incorporating Global Amendment 1 dated 21/MAY/2013)	23/MAY/2014
Schulman Associates Institutional Review Board, Inc.<br>4445 Lake Forest Drive Suite 300<br>Cincinnati, Ohio 45242<br><br>ADVARRA<br>6940 Columbia Gateway	Sharon Lynn Nelson, MSN, RN, CNS<br><br>Sara Harnish, J.D	32425 / Shah, Shujahat	Protocol Version 3.0 dated 19/JUL/2011 and Local Amendment dated 17/FEB/2012<br><br>Protocol Version 4.0 dated 07/JUN/2013 (incorporating Global Amendment 1 dated 21/MAY/2013)	18/JUN/2014<br><br>18/JUN/2014

United States of America				
**IRB or IEC (name/address)**	**IRB or IEC Chairperson**	**Centre number (5 digit number) / Investigator(s)**	**Protocol and/or Amendment number(s)**	Date of Final Approval (DD/MMM/YYYY)
Drive,<br>Suite 110<br>Columbia, MD 21046<br>USA				
VA Greater Los Angeles Healthcare System IRB<br>11301 Wilshire Blvd.<br>Bldg. 500 Room 4425<br>Los Angeles, CA 90073	Dean T. Yamaguchi	32426 / Warner, Alberta	Protocol Version 3.0 dated 19/JUL/2011 and Local Amendment dated 17/FEB/2012<br><br>Protocol Version 4.0 dated 07/JUN/2013 (incorporating Global Amendment 1 dated 21/MAY/2013)	26/JAN/2015<br><br>26/JAN/2015
Schulman Associates Institutional Review	Sharon Lynn Nelson, MSN, RN, CNS	32427 / Luke, Robert	Protocol Version 3.0 dated 19/JUL/2011 and Local	20/AUG/2015

United States of America				
**IRB or IEC (name/address)**	**IRB or IEC Chairperson**	**Centre number (5 digit number) / Investigator(s)**	**Protocol and/or Amendment number(s)**	Date of Final Approval (DD/MMM/YYYY)
Board, Inc.<br>4445 Lake Forest Drive Suite 300<br>Cincinnati, Ohio 45242			Amendment dated 17/FEB/2012	
ADVARRA<br>6940 Columbia Gateway Drive,<br>Suite 110<br>Columbia, MD 21046	Sara Harnish, J.D		Protocol Version 4.0 dated 07/JUN/2013 (incorporating Global Amendment 1 dated 21/MAY/2013)	20/AUG/2015
Schulman Associates Institutional Review Board, Inc.<br>4445 Lake Forest Drive Suite 300<br>Cincinnati, Ohio 45242	Sharon Lynn Nelson, MSN, RN, CNS	32428 / Goldstein, Robert	Protocol Version 3.0 dated 19/JUL/2011 and Local Amendment dated 17/FEB/2012	20/JUL/2015
ADVARRA<br>6940 Columbia Gateway Drive,	Sara Harnish, J.D		Protocol Version 4.0 dated 07/JUN/2013 (incorporating Global	20/JUL/2015

United States of America				
**IRB or IEC (name/address)**	**IRB or IEC Chairperson**	**Centre number (5 digit number) / Investigator(s)**	**Protocol and/or Amendment number(s)**	Date of Final Approval (DD/MMM/YYYY)
Suite 110<br>Columbia, MD 21046			Amendment 1 dated 21/MAY/2013)	
Willis-Knishton Health System<br>2600 Greenwood Road<br>Sheverport, LA<br>71103<br><br>Schulman Associates Institutional Review Board, Inc.<br>4445 Lake Forest Drive Suite 300<br>Cincinnati, Ohio 45242<br><br>ADVARRA<br>6940 Columbia Gateway Drive,<br>Suite 110	Louis Sardenga<br><br>Sharon Lynn Nelson, MSN, RN, CNS<br><br>Sara Harnish, J.D	32429 / Veerareddy, Srikar	Protocol Version 4.0 dated 07/JUN/2013 (incorporating Global Amendment 1 dated 21/MAY/2013)<br><br>Protocol Version 4.0 dated 07/JUN/2013 (incorporating Global Amendment 1 dated 21/MAY/2013)	19/OCT/2015<br><br>15/Aug/2017

United States of America				
**IRB or IEC (name/address)**	**IRB or IEC Chairperson**	**Centre number (5 digit number) / Investigator(s)**	**Protocol and/or Amendment number(s)**	Date of Final Approval (DD/MMM/YYYY)
Columbia, MD 21046				
Mary Washington Hospital IRB<br>1300 Hospital Drive,Suite 305<br>Fredericksburg, VA 22401	James R. Daniels	32430 / Vranian,, Robert	Protocol Version 3.0 dated 19/JUL/2011 and Local Amendment dated 17/FEB/2012<br><br>Protocol Version 4.0 dated 07/JUN/2013 (incorporating Global Amendment 1 dated 21/MAY/2013)	08/SEP/2015<br><br>08/SEP/2015
Schulman Associates Institutional Review Board, Inc.<br>4445 Lake Forest Drive Suite 300<br>Cincinnati, Ohio 45242	Sharon Lynn Nelson, MSN, RN, CNS	32432 / Phillips, Roland	Protocol Version 3.0 dated 19/JUL/2011 and Local Amendment dated 17/FEB/2012	02/SEP/2015

United States of America				
**IRB or IEC (name/address)**	**IRB or IEC Chairperson**	**Centre number (5 digit number) / Investigator(s)**	**Protocol and/or Amendment number(s)**	Date of Final Approval (DD/MMM/YYYY)
ADVARRA<br>6940 Columbia Gateway Drive,<br>Suite 110<br>Columbia, MD 21046	Sara Harnish, J.D		Protocol Version 4.0 dated 07/JUN/2013 (incorporating Global Amendment 1 dated 21/MAY/2013)	02/SEP/2015
Schulman Associates Institutional Review Board, Inc.<br>4445 Lake Forest Drive Suite 300<br>Cincinnati, Ohio 45242<br><br>ADVARRA<br>6940 Columbia Gateway Drive,<br>Suite 110<br>Columbia, MD 21046	Sharon Lynn Nelson, MSN, RN, CNS<br><br>Sara Harnish, J.D	32436 / Friedlander, Ira	Protocol Version 3.0 dated 19/JUL/2011 and Local Amendment dated 17/FEB/2012<br><br>Protocol Version 4.0 dated 07/JUN/2013 (incorporating Global Amendment 1 dated 21/MAY/2013)	19/JAN/2016<br><br>19/JAN/2016

Total number of centers: 1

Venezuela		Number of patients enrolled in country: 9			
Centre number	Investigator name and role	Affiliation and address	Facility description	# patients enrolled Phase II/centre	# patients enrolled Phase III/centre
05002	Principal Investigator(s): Mendoza Mujica, Ivan	Instituto de Clinicas y Urologia Tamanaco<br>Anexo D, Consultorio D<br>Calle Chivacoa,<br>San Roman, Caracas<br>Zip Code: 1061<br>Venezuela<br>Phone: 58 212 993 2866<br>Fax: 58 212 993 9157<br>Email: imivanjm(at)gmail.com	Practice	9	0
