# Supplementary material for: Atrial fibrillation and comorbidities: Clinical characteristics and antithrombotic treatment in GLORIA-AF
Source: PLoS One. 2021 Apr 14;16(4):e0249524. doi: 10.1371/journal.pone.0249524 (PMC8046191; doi:10.1371/journal.pone.0249524)
Supplement: Supplementary file 2 [file pone.0249524.s002.odt]

S2 File

Atrial Fibrillation and Comorbidities: Clinical Characteristics and Antithrombotic Treatment in GLORIA-AF

Running Headline: Multimorbidity in GLORIA-AF

Monika Kozieł, MD, PhD; Christine Teutsch, MD; Jonathan L. Halperin, MD; Kenneth J. Rothman, DrPH; Hans-Christoph Diener, MD, PhD; Chang-Sheng Ma, MD; Sabrina Marler MS; Shihai Lu, PhD; Venkatesh K. Gurusamy MSc; Menno V. Huisman, MD, PhD, FESC; and Gregory Y.H. Lip, MD; on behalf of the GLORIA-AF Investigators

From the Liverpool Centre for Cardiovascular Science, University of Liverpool and Liverpool Heart & Chest Hospital, Liverpool, UK (M.K., G.L.); 1st Department of Cardiology and Angiology, Silesian Centre for Heart Diseases, Zabrze, Poland (M.K., G.L.); Department of Clinical Development and Medical Affairs, Therapeutic Area Cardiometabolism, Boehringer Ingelheim International GmbH, Ingelheim, Germany (C.T.); Icahn School of Medicine at Mount Sinai, New York, NY (J.L.H.); RTI Health Solutions, Research Triangle Park, North Carolina (K.R.); fInstitute for Medical Informatics, Biometry and Epidemiology, University of Duisburg-Essen, Essen, Germany (H-C.D.); Cardiology Department, Atrial Fibrillation Center, Beijing AnZhen Hospital, Capital Medical University, Beijing, China (C-S.M); Biostatistics and Data Sciences, Boehringer Ingelheim Pharmaceuticals, Inc., Ridgefield, Connecticut (S.L., S.M.); Global Epidemiology, Boehringer Ingelheim International GmbH, Ingelheim, Germany (V.K.); Department of Thrombosis and Hemostasis, Leiden University Medical Center, Leiden, the Netherlands (M.V.H.); Aalborg Thrombosis Research Unit, Department of Clinical Medicine, Aalborg University, Aalborg, Denmark

S1 Table. Multivariable Log-binomial Analysis For Factors Associated With Baseline Prescription of OAC Therapy (no OAC vs OAC)a,b

| Factor | Relative Risk (95% CI) For Prescription of No OAC Globally |
| --- | --- |
| Age |  |
| <65 | 1.05 (0.95-1.16) |
| 65-74 | 0.90 (0.83- 0.99) |
| ≥75 | 1.0 (ref) |
| BMI class |  |
| <18.5 | 0.98 (0.77-1.24) |
| 18.5-24 | 1.0 (ref) |
| 25-29 | 0.85 (0.79-0.91) |
| 30-34 | 0.77 (0.69-0.87) |
| ≥35 | 0.70 (0.60-0.81) |
| Gender |  |
| Male | 1.0 (ref) |
| Female | 1.05 (0.97-1.13) |
| Alcohol abuse | 0.93 (0.80-1.08) |
| Psychosocial factors | 0.99 (0.85-1.14) |
| Current smoker | 1.14 (1.03-1.25) |
| Past smoker | 0.91 (0.84-0.99) |
| Categorization of AF |  |
| EHRA I | 1.0 (ref) |
| EHRA II | 1.04 (0.96-1.12) |
| EHRA III | 0.99 (0.91-1.07) |
| EHRA IV | 1.07 (0.95- 1.20) |
| Type of AF |  |
| Paroxysmal | 1.67 (1.42-1.97) |
| Persistent | 1.20 (1.02-1.43) |
| Permanent | 1.0 (ref) |
| Hypertension | 0.89 (0.83-0.97) |
| Coronary artery disease | 1.42 (1.31-1.53) |
| Myocardial infarction | 1.18 (1.08-1.28) |
| Congestive heart failure | 1.01 (0.94-1.08) |
| Diabetes mellitus | 0.95 (0.88-1.02) |
| Previous TIA or stroke | 0.81 (0.68-0.97) |
| Bleeding after diagnosis of AF | 1.60 (1.42-1.79) |
| Peripheral artery disease | 1.13 (0.96-1.34) |
| Biological heart valve implant | 0.73 (0.38-1.39) |
| Valve repair | 0.93 (0.50-1.74) |
| Cancer | 1.00 (0.90-1.12) |
|  |  |
| Gastric ulcer | 0.91 (0.69-1.21) |
| Peptic ulcer | 0.86 (0.42-1.76) |
| Gastritis or duodenitis | 0.95 (0.82-1.10) |
| Functional dyspepsia | 0.85 (0.56-1.27) |
| Hepatic disease | 1.05 (0.87-1.27) |
| COPD | 1.03 (0.90-1.19) |
| Dementia | 1.09 (0.76-1.56) |
| Hyperthyroidism | 0.96 (0.89-1.04) |
| AF cardioversion | 0.96 (0.89-1.04) |
| Creatinine clearance (mL/min) |  |
| <30 | 1.09 (0.94-1.26) |
| 30-59 | 0.88 (0.79-0.97) |
| 60-79 | 0.91 (0.83-1.00) |
| ≥80 | 1.0 (ref) |
| AF ablation | 0.30 (0.20-0.45) |
| Region |  |
| Asia | 3.17 (2.88-3.49) |
| Europe | 1.0 (ref) |
| North America | 1.24 (1.11-1.39) |
| Latin America | 1.14 (0.96-1.37) |
| Medical treatment reimbursed by |  |
| Self-pay/no coverage | 0.82 (0.69-0.96) |
| Not self-pay | 1.0 (ref) |
| Type of site |  |
| Specialist office | 1.26 (1.14-1.39) |
| Community hospital | 1.0 (ref) |
| University hospital | 1.28 (1.17-1.40) |

aAF = atrial fibrillation; BMI = body mass index; CI = confidence interval; COPD = chronic obstructive pulmonary disease; EHRA = European Heart Rhythm Association; OAC = oral anticoagulant; ref = reference; TIA = transient ischemic attack.  

S2 Table. Multivariable Log-Binomial Analysis for Factors Associated With Baseline Prescription of Oral Antithrombotic Therapy (no OAC vs OAC)a,b

| Factor | Relative Risk (95% CI) for Prescription of No OAC in Asia | Relative Risk (95% CI) for Prescription of No OAC in Europe | Relative Risk (95% CI) for Prescription of No OAC in North America |
| --- | --- | --- | --- |
| Age |  |  |  |
| <65 | 0.87 (0.77-0.99) | 1.43 (1.15-1.79) | 1.37 (1.07-1.76) |
| 65-74 | 0.90 (0.81-1.00) | 0.89 (0.74-1.07) | 0.95 (0.77-1.16) |
| ≥75 | 1.0 (ref) | 1.0 (ref) | 1.0 (ref) |
| BMI class |  |  |  |
| <18.5 | 1.03 (0.83-1.28) | 0.62 (0.29-1.34) | 1.51 (0.83-2.76) |
| 18.5-24 | 1.0 (ref) | 1.0 (ref) | 1.0 (ref) |
| 25-29 | 0.90 (0.83-0.98) | 0.89 (0.75-1.06) | 0.89 (0.71-1.11) |
| 30-34 | 0.75 (0.61-0.91) | 0.82 (0.66-1.02) | 0.96 (0.75-1.23) |
| ≥ 35 | 0.57 (0.36-0.89) | 0.62 (0.46-0.84) | 0.89 (0.67-1.17) |
| Gender |  |  |  |
| Male | 1.0 (ref) | 1.0 (ref) | 1.0 (ref) |
| Female | 1.00 (0.90-1.11) | 1.02 (0.87-1.20) | 1.05 (0.88-1.24) |
| Current smoker | 1.09 (0.98-1.23) | 1.08 (0.85-1.37) | 1.31 (1.03-1.68) |
| Past smoker | 0.94 (0.82-1.07) | 0.97 (0.82-1.15) | 0.89 (0.75-1.05) |
| Alcohol abuse | 0.86 (0.65-1.13) | 1.03 (0.80-1.31) | 0.86 (0.63-1.18) |
| Psychosocial factors | 0.85 (0.53-1.35) | 1.04 (0.81-1.34) | 0.92 (0.75-1.13) |
| EHRA I | 1.0 (ref) | 1.0 (ref) | 1.0 (ref) |
| EHRA II | 1.02 (0.93-1.11) | 0.83 (0.70-1.00) | 1.10 (0.93-1.32) |
| EHRA III | 1.00 (0.90-1.11) | 1.04 (0.86-1.25) | 1.00 (0.81-1.23) |
| EHRA IV | 0.92 (0.75-1.14) | 1.19 (0.94-1.51) | 1.17 (0.82-1.68) |
| Type of AF |  |  |  |
| Paroxysmal | 1.36 (1.06-1.75) | 1.89 (1.45-2.47) | 1.84 (1.01-3.36) |
| Persistent | 1.20 (0.93-1.56) | 1.18 (0.89-1.57) | 0.90 (0.48-1.68) |
| Permanent | 1.0 (ref) | 1.0 (ref) | 1.0 (ref) |
| Hypertension | 0.94 (0.85-1.03) | 0.86 (0.72-1.02) | 0.94 (0.75-1.18) |
| Coronary artery disease | 1.54 (1.40-1.71) | 1.21 (0.99-1.48) | 1.15 (0.95-1.39) |
| Myocardial infarction | 1.18 (1.07-1.31) | 1.18 (0.94-1.48) | 1.07 (0.84-1.36) |
| Congestive heart failure | 1.11 (1.04-1.19) | 0.72 (0.60-0.86) | 0.87 (0.71-1.05) |
| Diabetes mellitus | 1.00 (0.92-1.08) | 1.00 (0.85-1.17) | 0.82 (0.69-0.97) |
| Previous TIA or stroke | 0.93 (0.70-1.22) | 0.63 (0.45-0.88) | 0.86 (0.62-1.18) |
| Bleeding after diagnosis of AF | 1.41 (1.20-1.66) | 1.83 (1.28-2.62) | 1.97 (1.40-2.77) |
| Peripheral artery disease | 1.13 (0.79-1.62) | 1.25 (0.95-1.65) | 1.33 (0.99-1.79) |
| Biological heart valve implant | NA | 0.93 (0.40-2.16) | 0.76 (0.26-2.21) |
| Valve repair | NA | 0.64 (0.22-1.84) | 2.22 (1.01-4.88) |
| Cancer | 0.67 (0.51-0.88) | 1.15 (0.95-1.40) | 1.29 (1.08-1.54) |
| Functional dyspepsia | 0.53 (0.19-1.46) | 1.19 (0.63-2.25) | 0.97 (0.49-1.93) |
| Gastric ulcer | 0.89 (0.60-1.32) | 0.82 (0.39-1.73) | 1.67 (0.96-2.91) |
| Gastritis or duodenitis | 0.81 (0.65-1.00) | 1.44 (1.05-1.98) | 1.07 (0.70-1.63) |
| COPD | 0.97 (0.78-1.21) | 1.19 (0.94-1.50) | 0.88 (0.68-1.13) |
| Hyperthyroidism | 0.90 (0.64-1.26) | 1.00 (0.70-1.43) | 0.90 (0.61-1.33) |
| Hepatic disease | 0.98 (0.80-1.19) | 1.63 (1.02-2.62) | 1.70 (1.10-2.63) |
| Dementia | 1.14 (0.64-2.01) | 1.17 (0.56-2.42) | 1.85 (0.89-3.82) |
| AF cardioversion | 1.09 (0.99-1.19) | 0.99 (0.83-1.19) | 0.57 (0.45-0.72) |
| Creatinine clearance (mL/min) |  |  |  |
| <30 | 1.05 (0.87-1.27) | 1.53 (1.09-2.17) | 1.31 (0.85-2.02) |
| 30-59 | 0.90 (0.79-1.02) | 1.04 (0.83-1.31) | 0.96 (0.74-1.24) |
| 60-79 | 0.97 (0.87-1.07) | 1.01 (0.81-1.25) | 0.89 (0.70-1.14) |
| ≥80 | 1.0 (ref) | 1.0 (ref) | 1.0 (ref) |
| AF ablation | 0.30 (0.19-0.47) | 0.46 (0.12-1.73) | 0.18 (0.03-1.20) |
| Medical treatment reimbursed by |  |  |  |
| Self-pay/no coverage | 0.74 (0.60-0.91) | 1.30 (0.86-1.97) | 1.05 (0.66-1.66) |
| Not self-pay | 1.0 (ref) | 1.0 (ref) | 1.0 (ref) |
| Type of site |  |  |  |
| Specialist office | 2.25 (1.87-2.70) | 0.47 (0.35-0.62) | 1.19 (0.90 -1.57) |
| Community hospital | 1.0 (ref) | 1.0 (ref) | 1.0 (ref) |
| University hospital | 2.12 (1.77-2.53) | 0.84 (0.72-0.99) | 1.71 (1.25-2.32) |

aThe Latin America region is not included due to the small number of Latin American patients in the No OAC group (N=130).

bAF = atrial fibrillation; BMI = body mass index; CI = confidence interval; COPD = chronic obstructive pulmonary disease; EHRA = European Heart Rhythm Association; NA = not available; NOAC = non-vitamin K antagonist oral anticoagulant; OAC = oral anticoagulant; ref = reference; TIA = transient ischaemic attack; VKA = vitamin K antagonist.  

S3 Table. Multivariable Log-Binomial Analysis For Factors Associated With Baseline Prescription of OAC Therapy (VKA vs NOAC)a

| Factor | Relative Risk (95% CI) For Prescription of VKA Globally |
| --- | --- |
| Age |  |
| <65 | 1.20 (1.11-1.30) |
| 65-74 | 1.09 (1.02-1.17) |
| ≥75 | 1.0 (ref) |
| BMI class |  |
| <18.5 | 0.91 (0.70-1.17) |
| 18.5-24 | 1.0 (ref) |
| 25-29 | 0.99 (0.92-1.06) |
| 30-34 | 1.01 (0.93-1.10) |
| ≥35 | 0.98 (0.89-1.08) |
| Gender |  |
| Male | 1.0 (ref) |
| Female | 1.00 (0.95-1.07) |
| Current smoker | 1.04 (0.94-1.15) |
| Past smoker | 1.01 (0.95-1.07) |
| Alcohol abuse | 0.92 (0.82-1.04) |
| Psychosocial factors | 0.95 (0.87-1.03) |
| EHRA I | 1.0 (ref) |
| EHRA II | 1.12 (1.05-1.20) |
| EHRA III | 1.16 (1.08-1.24) |
| EHRA IV | 1.20 (1.09-1.32) |
| Type of AF |  |
| Paroxysmal | 0.77 (0.71-0.83) |
| Persistent | 0.91 (0.85-0.98) |
| Permanent | 1.0 (ref) |
| Hypertension | 1.02 (0.95-1.11) |
| Coronary artery disease | 1.07 (0.99-1.16) |
| Myocardial infarction | 1.12 (1.03-1.23) |
| Congestive heart failure | 1.24 (1.17-1.32) |
| Diabetes mellitus | 1.08 (1.02-1.14) |
| Previous TIA or stroke | 0.86 (0.75-0.97) |
| Bleeding after diagnosis of AF | 1.01 (0.81-1.26) |
| Peripheral artery disease | 1.09 (0.98-1.22) |
| Biological heart valve implant | 1.33 (1.03-1.72) |
| Valve repair | 1.40 (1.10-1.78) |
| Cancer | 1.05 (0.97-1.13) |
| Functional dyspepsia | 0.84 (0.62-1.13) |
| Gastric ulcer | 0.82 (0.61-1.09) |
| Gastritis or duodenitis | 1.01 (0.91-1.12) |
| COPD | 1.04 (0.94-1.14) |
| Hyperthyroidism | 0.97 (0.82-1.13) |
| Hepatic disease | 1.07 (0.88-1.31) |
| Dementia | 0.80 (0.58-1.11) |
| AF cardioversion | 0.75 (0.70-0.82) |
| Creatinine clearance (mL/min) |  |
| <30 | 1.57 (1.42-1.73) |
| 30-59 | 1.16 (1.06-1.26) |
| 60-79 | 1.05 (0.96-1.15) |
| ≥80 | 1.0 (ref) |
| AF ablation | 0.91 (0.72-1.15) |
| Region |  |
| Asia | 0.92 (0.85-1.00) |
| Europe | 1.0 (ref) |
| North America | 0.67 (0.62-0.73) |
| Latin America | 1.21 (1.11-1.31) |
| Medical treatment reimbursed by |  |
| Self-pay/no coverage | 0.80 (0.70-0.92) |
| Not self-pay | 1.0 (ref) |
| Type of site |  |
| Specialist office | 1.10 (1.01-1.20) |
| Community hospital | 1.0 (ref) |
| University hospital | 1.55 (1.44-1.66) |

aAF = atrial fibrillation; BMI = body mass index; CI = confidence interval; COPD = chronic obstructive pulmonary disease; EHRA = European Heart Rhythm Association; NOAC = nonvitamin K antagonist oral anticoagulant; OAC = oral anticoagulant; ref = reference; TIA = transient ischemic attack; VKA = vitamin K antagonist.

S4 Table. Multivariable Log-Binomial Analysis for Factors Associated With Baseline Prescription of Oral Antithrombotic Therapy (VKA vs NOAC) in Asiaa

| Factor | Relative Risk (95% CI) for Prescription of VKA in Asia |
| --- | --- |
| Age |  |
| <65 | 1.56 (1.25-1.96) |
| 65-74 | 1.38 (1.12-1.69) |
| ≥75 | 1.0 (ref) |
| BMI class |  |
| <18.5 | 1.08 (0.68-1.70) |
| 18.5-24 | 1.0 (ref) |
| 25-29 | 1.03 (0.88-1.20) |
| 30-34 | 0.90 (0.70-1.16) |
| ≥35 | 0.81 (0.52-1.25) |
| Gender |  |
| Male | 1.0 (ref) |
| Female | 0.99 (0.84-1.16) |
| Current smoker | 1.17 (0.96-1.43) |
| Past smoker | 1.06 (0.88-1.28) |
| Alcohol abuse | 0.96 (0.67-1.37) |
| Psychosocial factors | 0.56 (0.25-1.25) |
| EHRA I | 1.0 (ref) |
| EHRA II | 1.08 (0.91-1.28) |
| EHRA III | 1.06 (0.88-1.29) |
| EHRA IV | 0.69 (0.46-1.06) |
| Type of AF |  |
| Paroxysmal | 0.91 (0.70-1.18) |
| Persistent | 0.97 (0.74-1.27) |
| Permanent | 1.0 (ref) |
| Hypertension | 0.93 (0.78-1.10) |
| Coronary artery disease | 1.46 (1.25-1.71) |
| Myocardial infarction | 0.99 (0.78-1.26) |
| Congestive heart failure | 1.23 (1.06-1.43) |
| Diabetes mellitus | 0.93 (0.80-1.07) |
| Previous TIA or stroke | 0.93 (0.66-1.30) |
| Bleeding after diagnosis of AF | 0.74 (0.27-2.00) |
| Peripheral artery disease | 1.14 (0.55-2.36) |
| Cancer | 1.10 (0.84-1.45) |
| Functional dyspepsia | 0.93 (0.43-2.02) |
| Gastric ulcer | 0.47 (0.19-1.17) |
| Gastritis or duodenitis | 0.91 (0.70-1.19) |
| COPD | 0.74 (0.46-1.20) |
| Hyperthyroidism | 1.11 (0.79-1.54) |
| Hepatic disease | 0.75 (0.51-1.11) |
| Dementia | 0.50 (0.09-2.62) |
| AF cardioversion | 1.11 (0.91-1.35) |
| Creatinine clearance (mL/min) |  |
| <30 | 1.47 (0.97-2.20) |
| 30-59 | 1.07 (0.85-1.34) |
| 60-79 | 0.93 (0.77-1.12) |
| ≥80 | 1.0 (ref) |
| AF ablation | 0.75 (0.57-1.00) |
| Medical treatment reimbursed by |  |
| Self-pay/no coverage | 0.90 (0.71-1.14) |
| Not self-pay | 1.0 (ref) |
| Type of site |  |
| Specialist office | 3.98 (2.95-5.37) |
| Community hospital | 1.0 (ref) |
| University hospital | 3.42 (2.58-4.53) |

aAF = atrial fibrillation; BMI = body mass index; CI = confidence interval; COPD = chronic obstructive pulmonary disease; EHRA = European Heart Rhythm Association; NOAC = non-vitamin K antagonist oral anticoagulant; OAC = oral anticoagulant; TIA = transient ischaemic attack; VKA = vitamin K antagonist.

S5 Table. Multivariable Log-Binomial Analysis for Factors Associated With Baseline Prescription of Oral Antithrombotic Therapy (VKA vs NOAC) in Europea

| Factor | Relative Risk (95% CI) for Prescription of VKA in Europe |
| --- | --- |
| Age |  |
| <65 | 1.12 (0.98-1.27) |
| 65-74 | 1.12 (1.03-1.22) |
| ≥75 | 1.0 (ref) |
| BMI class |  |
| <18.5 | 0.82 (0.56-1.22) |
| 18.5-24 | 1.0 (ref) |
| 25-29 | 1.01 (0.92-1.11) |
| 30-34 | 1.11 (1.00-1.24) |
| ≥35 | 0.98 (0.86-1.13) |
| Gender |  |
| Male | 1.0 (ref) |
| Female | 1.00 (0.92-1.08) |
| Alcohol abuse | 0.95 (0.82-1.10) |
| Psychosocial factors | 1.02 (0.93-1.14) |
| Current smoker | 0.95 (0.83-1.09) |
| Past smoker | 1.03 (0.94-1.12) |
| EHRA I | 1.0 (ref) |
| EHRA II | 1.08 (0.99-1.18) |
| EHRA III | 1.07 (0.97-1.18) |
| EHRA IV | 1.20 (1.07-1.35) |
| Type of AF |  |
| Paroxysmal | 0.78 (0.71-0.87) |
| Persistent | 0.97 (0.88-1.06) |
| Permanent | 1.0 (ref) |
| Hypertension | 1.03 (0.93-1.14) |
| Coronary artery disease | 1.00 (0.90-1.12) |
| Myocardial infarction | 1.20 (1.07-1.36) |
| Congestive heart failure | 1.11 (1.02-1.20) |
| Diabetes mellitus | 1.08 (1.00-1.17) |
| Previous TIA or stroke | 0.81 (0.69-0.94) |
| Bleeding after diagnosis of AF | 0.98 (0.82-1.18) |
| Peripheral artery disease | 1.08 (0.93-1.26) |
| Biological heart valve implant | 1.54 (1.19-2.00) |
| Valve repair | 1.23 (0.82-1.86) |
| Cancer | 1.00 (0.91-1.11) |
| Functional dyspepsia | 0.91 (0.62-1.33) |
| Gastric ulcer | 0.96 (0.68-1.35) |
| Gastritis or duodenitis | 1.15 (0.93-1.41) |
| COPD | 1.03 (0.91-1.18) |
| Hyperthyroidism | 0.97 (0.80-1.19) |
| Hepatic disease | 1.25 (0.94-1.66) |
| Dementia | 0.89 (0.62-1.27) |
| AF cardioversion | 0.72 (0.64-0.80) |
| Creatinine clearance (mL/min) |  |
| <30 | 1.77 (1.53-2.04) |
| 30-59 | 1.23 (1.09-1.38) |
| 60-79 | 1.12 (0.99-1.26) |
| ≥80 | 1.0 (ref) |
| AF ablation | 0.42 (0.17-1.06) |
| Medical treatment reimbursed by |  |
| Self-pay/no coverage | 0.87 (0.68-1.12) |
| Not self-pay | 1.0 (ref) |
| Type of site |  |
| Specialist office | 0.97 (0.86-1.09) |
| Community hospital | 1.0 (ref) |
| University hospital | 1.42 (1.31-1.54) |

aAF = atrial fibrillation; BMI = body mass index; CI = confidence interval; COPD = chronic obstructive pulmonary disease; EHRA = European Heart Rhythm Association; NOAC = non-vitamin K antagonist oral anticoagulant; OAC = oral anticoagulant; TIA = transient ischaemic attack; VKA = vitamin K antagonist.

S6 Table. Multivariable Log-Binomial Analysis for Factors Associated With Baseline Prescription of Oral Antithrombotic Therapy (VKA vs NOAC) in North Americaa

| Factor | Relative Risk (95% CI) for Prescription of VKA in North America |
| --- | --- |
| Age |  |
| <65 | 1.09 (0.86-1.37) |
| 65-74 | 1.11 (0.94-1.32) |
| ≥75 | 1.0 (ref) |
| BMI class |  |
| <18.5 | 0.72 (0.35-1.47) |
| 18.5-24 | 1.0 (ref) |
| 25-29 | 0.81 (0.66-1.00) |
| 30-34 | 0.92 (0.74-1.15) |
| ≥35 | 1.02 (0.81-1.29) |
| Gender |  |
| Male | 1.0 (ref) |
| Female | 0.98 (0.84-1.15) |
| Alcohol abuse | 0.83 (0.61-1.13) |
| Current smoker | 1.15 (0.89-1.49) |
| Past smoker | 1.03 (0.89-1.19) |
| Psychosocial factors | 0.96 (0.79-1.16) |
| EHRA I | 1.0 (ref) |
| EHRA II | 1.10 (0.93-1.30) |
| EHRA III | 1.24 (1.04-1.48) |
| EHRA IV | 1.11 (0.84-1.47) |
| Type of AF |  |
| Paroxysmal | 0.87 (0.62-1.22) |
| Persistent | 0.96 (0.67-1.36) |
| Permanent | 1.0 (ref) |
| Hypertension | 0.98 (0.79-1.21) |
| Coronary artery disease | 0.96 (0.80-1.15) |
| Myocardial infarction | 1.17 (0.95-1.45) |
| Congestive heart failure | 1.50 (1.29-1.74) |
| Diabetes mellitus | 1.20 (1.04-1.39) |
| Previous TIA or stroke | 0.76 (0.55-1.05) |
| Bleeding after diagnosis of AF | 1.33 (0.85-2.08) |
| Peripheral artery disease | 1.14 (0.84-1.55) |
| Biological heart valve implant | 0.83 (0.32-2.15) |
| Valve repair | 2.01 (0.87-4.66) |
| Cancer | 1.21 (1.03-1.43) |
| Functional dyspepsia | 0.59 (0.28-1.26) |
| Gastric ulcer | 1.19 (0.66-2.15) |
| Gastritis or duodenitis | 1.37 (0.98-1.91) |
| COPD | 0.99 (0.81-1.22) |
| Hyperthyroidism | 0.89 (0.61-1.31) |
| Hepatic disease | 1.91 (1.19-3.06) |
| Dementia | 0.83 (0.36-1.90) |
| AF cardioversion | 0.74 (0.61-0.90) |
| Creatinine clearance (mL/min) |  |
| <30 | 2.26 (1.70-3.02) |
| 30-59 | 1.44 (1.16-1.79) |
| 60-79 | 1.08 (0.86-1.35) |
| ≥80 | 1.0 (ref) |
| AF ablation | 1.37 (1.01-1.87) |
| Medical treatment reimbursed by |  |
| Self-pay/no coverage | 1.89 (1.38-2.57) |
| Not self-pay | 1.0 (ref) |
| Type of site |  |
| Specialist office | 0.79 (0.63-0.98) |
| Community hospital | 1.0 (ref) |
| University hospital | 1.45 (1.14-1.83) |

aAF = atrial fibrillation; BMI = body mass index; CI = confidence interval; COPD = chronic obstructive pulmonary disease; EHRA = European Heart Rhythm Association; NOAC = non-vitamin K antagonist oral anticoagulant; OAC = oral anticoagulant; TIA = transient ischaemic attack; VKA = vitamin K antagonist.

S7 Table. Multivariable Log-Binomial Analysis for Factors Associated With Baseline Prescription of Oral Antithrombotic Therapy (VKA vs NOAC) in Latin Americaa

| Factor | Relative Risk (95% CI) for Prescription of VKA in Latin America |
| --- | --- |
| Age |  |
| <65 | 1.08 (0.91-1.28) |
| 65-74 | 0.96 (0.83-1.11) |
| ≥75 | 1.0 (ref) |
| BMI class |  |
| <18.5 | 1.21 (0.77-1.92) |
| 18.5-24 | 1.0 (ref) |
| 25-29 | 0.98 (0.85-1.14) |
| 30-34 | 0.89 (0.73-1.09) |
| ≥35 | 0.94 (0.79-1.12) |
| Gender |  |
| Male | 1.0 (ref) |
| Female | 0.96 (0.84-1.11) |
| Alcohol abuse | 1.06 (0.88-1.28) |
| Current smoker | 1.04 (0.87-1.23) |
| Past smoker | 1.04 (0.89-1.20) |
| Psychosocial factors | 0.93 (0.69-1.24) |
| EHRA I | 1.0 (ref) |
| EHRA II | 1.38 (1.13-1.69) |
| EHRA III | 1.58 (1.30-1.92) |
| EHRA IV | 1.51 (1.16-1.95) |
| Type of AF |  |
| Paroxysmal | 0.82 (0.67-0.99) |
| Persistent | 0.88 (0.77-1.01) |
| Permanent | 1.0 (ref) |
| Hypertension | 1.20 (0.91-1.57) |
| Coronary artery disease | 0.86 (0.65-1.15) |
| Myocardial infarction | 1.22 (0.90-1.64) |
| Congestive heart failure | 1.46 (1.25-1.71) |
| Diabetes mellitus | 1.03 (0.91-1.16) |
| Previous TIA or stroke | 1.07 (0.78-1.48) |
| Bleeding after diagnosis of AF | 0.47 (0.14-1.54) |
| Peripheral artery disease | 1.34 (1.00-1.79) |
| Cancer | 0.98 (0.74-1.31) |
| Functional dyspepsia | 0.93 (0.37-2.35) |
| Gastric ulcer | 0.45 (0.08-2.64) |
| Gastritis or duodenitis | 0.98 (0.78-1.24) |
| COPD | 1.11 (0.97-1.28) |
| Hyperthyroidism | 0.76 (0.38-1.53) |
| Hepatic disease | 1.44 (1.05-1.98) |
| Dementia | 0.76 (0.20-2.89) |
| AF cardioversion | 0.77 (0.60-0.99) |
| Creatinine clearance (mL/min) |  |
| <30 | 1.13 (0.95-1.33) |
| 30-59 | 0.90 (0.74-1.11) |
| 60-79 | 0.85 (0.68-1.07) |
| ≥80 | 1.0 (ref) |
| Medical treatment reimbursed by |  |
| Self-pay/no coverage | 0.54 (0.40-0.72) |
| Not self-pay | 1.0 (ref) |
| Type of site |  |
| Specialist office | 1.07 (0.80-1.41) |
| Community hospital | 1.0 (ref) |
| University hospital | 1.50 (1.14-1.96) |

aAF = atrial fibrillation; BMI = body mass index; CI = confidence interval; COPD = chronic obstructive pulmonary disease; EHRA = European Heart Rhythm Association; NOAC = non-vitamin K antagonist oral anticoagulant; OAC = oral anticoagulant; TIA = transient ischaemic attack; VKA = vitamin K antagonist.
