# Supplementary material for: Atrial fibrillation and comorbidities: Clinical characteristics and antithrombotic treatment in GLORIA-AF
Source: PLoS One. 2021 Apr 14;16(4):e0249524. doi: 10.1371/journal.pone.0249524 (PMC8046191; doi:10.1371/journal.pone.0249524)
Supplement: Attachment — Submitted filename: Gloria-AF registry,.pdf [file pone.0249524.s003.pdf]

The authors of the paper “**Atrial Fibrillation and Comorbidities: Clinical Characteristics and Antithrombotic Treatment in GLORIA-AF**" have been focused on the analysis objective, specifically in baseline characteristics and antithrombotic therapy in patients with AF and more than two concomitant and chronic comorbidities. Authors have done, through Gloria-AF registry, an international covered registry, a genuine work in screening of baseline characteristics and medication of an enormous number of patients with AF during a three-year enrolling period. Even though we have the baseline picture, it is of a big interest to know the trend or changes with regards to oral anticoagulation (OAC) therapy during the follow-up (FU), but as mentioned in Limitations the authors have chosen to show the FU data probably later on.

General comments:

The manuscript is well written with the objective and methodology clearly described. The authors make an important point showing that patients with AF and a high-risk profile for stroke, more than 2 chronic comorbidities, were unfortunately undertreated with NOAC, in a period when at least three NOACs were established in the clinical use. According to the authors, around 60% of those patients were on NOAC regimen, but still 16% of those patients were without OAC, despite CHA_2DS_2-VASc ≥ 2. This work points the fact that younger patients with AF and less comorbidities were likely to were on NOAC regimen, at the same time that observations and clinical practice today show something different. In this perspective, I think it is very important the future information from the registry if the trends during the follow-up will show the impact of implementation of the current guidelines for the antithrombotic therapy in patients with AF.

I have some comments I would like to address to the authors:

1. I will appreciate if the authors can explain or argue what was the reason for choosing of standardized differences in order to compare baseline characteristics between different stroke prevention strategies.
2. Again, when considering the trend aspect, it is of interest if a trend of increased NOAC use was observed during the last year of inclusion as compared with the previous year(s).

3. In Discussion, I feel sometimes that authors give some more results rather than discuss the findings and face them with the literature, and specifically in the second paragraph.
4. I miss the comparison and discussion with some more recent findings regarding to NOAC use, where data from Scandinavia has given a different picture than probably other regions in Europe.
5. The tables are informative and presented well, but sometimes the perception of overflow is not avoidable. The possibility to cut some information could be considered.
6. I would like to have in the ordinary tables one of those included in supplements, specifically table S3 and would recommend authors to change with another one. I believe that one of strengths with this analysis is the region-specific patients enrolling and comparisons.
