# Supplementary material for: Atrial fibrillation and comorbidities: Clinical characteristics and antithrombotic treatment in GLORIA-AF
Source: PLoS One. 2021 Apr 14;16(4):e0249524. doi: 10.1371/journal.pone.0249524 (PMC8046191; doi:10.1371/journal.pone.0249524)
Supplement: Attachment — Submitted filename: Rebuttal PLOS one (2)GYHLMK.kjr (1) (3).docx [file pone.0249524.s004.docx]

Reviewer #1

The authors of the paper “Atrial Fibrillation and Comorbidities: Clinical Characteristics and Antithrombotic Treatment in GLORIA-AF" have been focused on the analysis objective, specifically in baseline characteristics and antithrombotic therapy in patients with AF and more than two concomitant and chronic comorbidities. Authors have done, through Gloria-AF registry, an international covered registry, a genuine work in screening of baseline characteristics and medication of an enormous number of patients with AF during a three-year enrolling period. Even though we have the baseline picture, it is of a big interest to know the trend or changes with regards to oral anticoagulation (OAC) therapy during the follow-up (FU), but as mentioned in Limitations the authors have chosen to show the FU data probably later on. General comments: The manuscript is well written with the objective and methodology clearly described. The authors make an important point showing that patients with AF and a high-risk profile for stroke, more than 2 chronic comorbidities, were unfortunately undertreated with NOAC, in a period when at least three NOACs were established in the clinical use. According to the authors, around 60% of those patients were on NOAC regimen, but still 16% of those patients were without OAC, despite CHA2DS2-VASc ≥ 2. This work points the fact that younger patients with AF and less comorbidities were likely to were on NOAC regimen, at the same time that observations and clinical practice today show something different. In this perspective, I think it is very important the future information from the registry if the trends during the follow-up will show the impact of implementation of the current guidelines for the antithrombotic therapy in patients with AF. I have some comments I would like to address to the authors:

1. I will appreciate if the authors can explain or argue what was the reason for choosing of standardized differences in order to compare baseline characteristics between different stroke prevention strategies.

***Authors’ response:***

*Thank you. Standardized differences were used because they are independent of sample size and it allows for the comparison of the relative balance of variables measured in different units.*

2. Again, when considering the trend aspect, it is of interest if a trend of increased NOAC use was observed during the last year of inclusion as compared with the previous year(s).

***Authors’ response:***

*Thank you. Trends of NOAC use during the last years of inclusion as compared with previous years has been addressed by a different manuscript which has been under separate development for submission.*

3. In Discussion, I feel sometimes that authors give some more results rather than discuss the findings and face them with the literature, and specifically in the second paragraph.

***Authors’ response:***

*Thank you. The Discussion has been edited and the findings have been discussed along with other studies (see pages 8-12).*

4. I miss the comparison and discussion with some more recent findings regarding to NOAC use, where data from Scandinavia has given a different picture than probably other regions in Europe.

***Authors’ response:***

*Thank you. Our findings have been compared and discussed with Scandinavian registries regarding NOAC (see pages 11 and 12).*

*‘Of note, younger patients (18-54 years) and ≥75 years were less likely to receive NOAC than those aged 65-74 years in Sweden (31). In Denmark, older age was associated with increased NOAC use (32).’*

*‘In contrast, HF was associated with decreased OAC initiation in Danish dataset (33).’*

*‘In Danish nationwide registries, bleeding was also associated with decreased OAC use (33).’*

*‘In our registry, previous TIA or stroke was the comorbidity associated with decreased VKA use in multimorbid AF patients in Europe. Interestingly, stroke/ thromboembolism or bleeding were associated with increased NOAC initiation in Denmark (32)’.*

5. The tables are informative and presented well, but sometimes the perception of overflow is not avoidable. The possibility to cut some information could be considered.

***Authors’ response:***

*Thank you. The tables have been carefully revised and some excessive information has been deleted (see Tables 2-5).*

6. I would like to have in the ordinary tables one of those included in supplements, specifically table S3 and would recommend authors to change with another one. I believe that one of strengths with this analysis is the region-specific patients enrolling and comparisons.

***Authors’ response:***

*Thank you. The Table S3 have been changed into separate tables for Asia, Europe, North America and South America (see Table S3, S4, S5 and S6).*

Reviewer #2: PONE-D-20-40256: statistical review

SUMMARY. This study describes factors that are associated with oral anticoagulant (OAC) prescription in subjects with atrial fibrillation and comorbidities. I am not sure that the association between patient's characteristics and prescription makes sense as a research question. However, I'm not a medical doctor and I am going to limit my discussion to the statistical methods that are deployed here.
From a statistical viewpoint, the core of the analysis relies on a cross-sectional log-binomial regression model, estimated by likelihood-based multiple imputation methods. These methods could in principle be appropriate, but the paper lacks information about the data structure and the multiple imputation method. I therefore need to ask first some questions (major issues 1 and 2 below) before making a recommendation.

MAJOR ISSUES

1. Little is said about the structure of the response variable: OAC prescriptions. The cross-sectional log-binomial regression model assumes that the response variable is a binary variable. It therefore makes sense if each subject receive only one type of prescription during the whole follow up. In this case, the model chosen provides a correct approach. If instead subjects switch between a type of prescription to another one, then this method is no longer correct and the data must be examined by a longitudinal version of the model that includes subject-specific random effects. Please clarify.

***Authors’ response:***

*Thank you. In this registry, there was no intervention in treatment prescription for patients over time, so patients can have more than one types of treatment prescribed during the study. The cross-sectional association analysis is based on the first prescription of antithrombotic treatment (i.e. index treatment) that was prescribed as long term use at baseline visit. Per inclusion criteria in protocol, the patients enrolled in the study must have been newly diagnosed (<3 months prior to baseline visit) with non-valvular AF. In order to clarify this definition of response variable in the manuscript, we have changed ‘OAC prescription’ to ‘baseline OAC prescription’.*

2. The authors correctly use multiple imputation to handle missing data in a regression framework. However, nothing is said about the model used to generate the imputation sample. If all the incomplete covariates are continuous, a multivariate normal distribution is typically used for imputation. However, in this study, incomplete covariates are of mixed type (some are continuous, others are categorical). I therefore wonder what model has been used at the imputation step.

***Authors’ response:***

*Thank you. The technique called multiple imputation by chained equations or fully conditional specification) was used to impute missing continuous and categorical variables. This method was well described in the reference ‘White IR, Royston P, Wood AM. Multiple imputation using chained equations: issues and guidance for practice. Stat Med. 2011;30(4):377-399.’. It provides the flexibility of handling different types of variables at the same time by employing different types of models accordingly. For example, ordinal linear regression is used for imputing continuous variable; logistic regression or discriminant function is used for (ordinal or nominal) categorical variables. For each of the total 56 covariates analysed, there is one imputation model specified. For this clinically orientated manuscript, the full details of this statistical analysis were not provided. However, we have added one sentence in the ‘statistical analysis’ section to explain what specific multiple imputation is used that can deal with both continuous and categorical variables (see page 6).*

*‘Multiple imputation by chained equations was used to impute both missing categorical and continuous values.’*
